# Molecular Basis of Disease Resistance in Banana Progenitor *Musa balbisiana* against *Xanthomonas campestris* pv. *musacearum*

**DOI:** 10.1038/s41598-019-43421-1

**Published:** 2019-05-07

**Authors:** Leena Tripathi, Jaindra Nath Tripathi, Trushar Shah, Kariuki Samwel Muiruri, Manpreet Katari

**Affiliations:** 1 International Institute of Tropical Agriculture (IITA), P.O. Box 30709-00100, Nairobi, Kenya; 2 Department of Biology, New York University, New York, NY United States

**Keywords:** Plant molecular biology, Biotic

## Abstract

Banana Xanthomonas wilt disease, caused by *Xanthomonas campestris* pv. *musacearum* (Xcm), is a major threat to banana production in east Africa. All cultivated varieties of banana are susceptible to Xcm and only the progenitor species *Musa balbisiana* was found to be resistant. The molecular basis of susceptibility and resistance of banana genotypes to Xcm is currently unknown. Transcriptome analysis of disease resistant genotype *Musa balbisiana* and highly susceptible banana cultivar Pisang Awak challenged with Xcm was performed to understand the disease response. The number of differentially expressed genes (DEGs) was higher in *Musa balbisiana* in comparison to Pisang Awak. Genes associated with response to biotic stress were up-regulated in *Musa balbisiana*. The DEGs were further mapped to the biotic stress pathways. Our results suggested activation of both PAMP-triggered basal defense and disease resistance (R) protein-mediated defense in *Musa balbisiana* as early response to Xcm infection. This study reports the first comparative transcriptome profile of the susceptible and resistant genotype of banana during early infection with Xcm and provide insights on the defense mechanism in *Musa balbisiana*, which can be used for genetic improvement of commonly cultivated banana varieties.

## Introduction

Banana Xanthomonas wilt (BXW), caused by the bacterium *Xanthomonas campestris* pv. *musacearum* (Xcm), is one of the most devastating disease endangering the livelihood of millions of farmers in east Africa, which is the largest banana-producing and -consuming region in Africa^1^. The impacts of BXW disease are both rapid and extreme, unlike those of other diseases, which cause gradually increasing losses over years. The disease has caused estimated economic losses of about $2-8 billion over the decade and significant reductions in production have resulted in major price increases^1^. The disease affects all banana cultivars grown in east Africa^2^. Only diploid *Musa balbisiana*, which is a wild type banana native to Asia and one of the progenitors of modern cultivated bananas, was found to be resistant^2^.

Resistant cultivars could play an important role in controlling the BXW disease in east Africa, where the consumption of bananas is highest in the world at 220 to 460 kg per person annually^3^. There is an ongoing project for developing transgenic bananas resistant to BXW using sweet pepper genes^4^. However, knowledge of resistance mechanism in *Musa balbisiana* against Xcm can be utilized for developing resistant varieties through cis-genesis using *Musa* genes associated with defense, or editing of genes related to susceptibility and/or negative regulation of plant immunity. Currently, there is no understanding about the molecular mechanism for disease resistance or susceptibility in response to Xcm infection. Therefore, to obtain insight into the molecular basis of disease resistance, the transcriptome-wide differential gene expression was investigated between the BXW-resistant genotype *Musa balbisiana* and BXW-susceptible genotype Pisang Awak in response to Xcm. Further the differentially expressed transcripts were mapped to biotic stress pathways using Mapman to identify genes associated with defense mechanism.

## Results

### RNA-Seq and mapping of sequences to reference genome

In this study, RNA-Seq data from Xcm challenged and mock-inoculated BXW-susceptible banana genotype Pisang Awak and BXW-resistant genotype *Musa balbisiana* were analyzed for transcriptome comparison.

In total, about 559 million raw reads were generated from sequencing of 30 libraries using Illumina HiSeq™ 2500. The majority of samples had average of 5–10 million reads that uniquely mapped to the reference *Musa acuminata* genome version 2. The raw reads were deposited in the National Center for Biotechnology Information Sequence Read Archive under project SRPRJNA401071 (https://www.ncbi.nlm.nih.gov/Traces/study/?acc=SRP116676).

### Differentially expressed genes (DEGs)

Readcounts for 35,238 annotated genes were loaded in R program and genes with low expression were filtered out. This filtering step reduced the dataset to 26,676 genes. The DEGs were identified in DESeq2 using filtered readcount data. The data from two time points, 12 h post inoculation (hpi) and 48 hpi, were analyzed and a model was created to identify genes that were responding to the treatment with pathogen and also had an interaction with the genotypes. The design parameter (~genotype + treatment + genotype:treatment) was used for DESeq2. The genes with fold change of greater than 1.5 or <−1.5 were identified.

**Figure 1:**
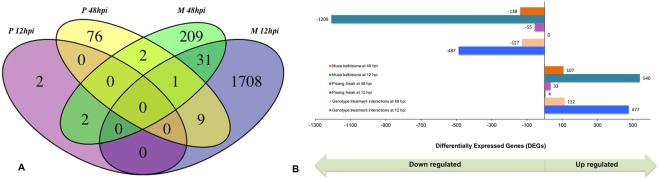
Graphs showing differentially expressed genes (DEGs) in BXW-resistant wild type banana genotype *Musa balbisiana* and BXW-susceptible banana genotype Pisang Awak at 12 and 48 hours post-inoculation with *Xanthomonas campestris* pv. *musacearum*. (**A)** Venn diagram showing comparison of DEGs in two genotypes at 12 hpi and 48 hpi, (**B)** Graphs showing number of up or down-regulated DEGs.

The numbers of DEGs were higher in BXW-resistant genotype *Musa balbisiana* in comparison to BXW-susceptible genotype Pisang Awak at both 12 hpi (1749 vs 4) and 48 hpi (245 vs 88) (Fig. 1A, Supplementary Tables S1–S4). Out of 1749 DEGs in *Musa balbisiana*, only 540 DEGs showed up-regulation and remaining 1209 were down-regulated, whereas all four DEGs in Pisang Awak were found to be up-regulated (Fig. 1B).

The number of DEGs for *Musa balbisiana* was considerably higher at 12 hpi in comparison to 48 hpi (1749 vs 245), in contrast a greater number of genes were differentially expressed at 48 hpi than at 12 hpi in Pisang Awak (88 vs 4) (Fig. 1B). In *Musa balbisiana*, 107/245 DEGs at 48 hpi were up-regulated, whereas only 33/88 DEGs were up-regulated in Pisang Awak at 48 hpi (Fig. 1B).

Several DEGs were observed due to genotype:treatment interactions (Supplementary Tables S5–S6). The difference in genotypes due to differences in treatment i.e. genotype:treatment interaction were observed to be higher at 12 hpi than 48 hpi.

### GO-term analysis

To know the functional categories of DEGs, GO enrichment analysis was performed using GO-terms provided by the Banana Genome Hub and the GOStats. A Gene Set Enrichment dataset was created using Gene-to-GO association in the Banana Genome Hub and used for identifying GO-terms that were over-represented in a given gene list. Out of 26,676 filtered genes set, 13,699 corresponding proteins were associated with at least one GO term. The GO-term analysis was performed for all the DEGs in both banana genotypes at 12 hpi and 48 hpi. At 12 hpi, the GO-terms were mainly due to interaction of bacterial pathogen with host plants (Supplementary Table S7).

No significant GO-term enrichment was seen for Pisang Awak at 12 hpi, however, many GO-terms were enriched for *Musa balbisiana* at 12 hpi (Supplementary Table S7). The majority of GO-terms in *Musa balbisiana* were associated with ‘Defense response’ suggesting that BXW-resistant genotype respond to pathogen attack at very early stage. At 48 hpi, several GO-terms were found in Pisang Awak, whereas only one significant GO-term was obtained for *Musa balbisiana* (Supplementary Table S7).

### Gene annotation and metabolic pathway analysis

The transcripts were annotated based on the genome of *Musa acuminata* version 2 and mapped to the biotic stress pathway in Mapman using the Mercator tool. Table 1 lists number of annotated genes aligned to different functional categories (BINs) in metabolic pathways in Mapman.

**Table 1:** List of number of differentially expressed genes (DEGs) in BXW-resistant banana genotype *Musa balbisiana* and BXW-susceptible banana genotype Pisang Awak mapped to different functional categories (BINs) in MapMan pathways.

Bin	Name	Pisang Awak at 12 hpi		*Musa balbisiana* at 12 hpi		Pisang Awak at 48 hpi		*Musa balbisiana* at 48 hpi	
		Up- regulated	Down- regulated	Up- regulated	Down- regulated	Up- regulated	Down- regulated	Up- regulated	Down- regulated
1	Photosynthesis	—	—	—	—	—	—	—	—
2	Major carbohydrate metabolism	—	—	1	—	—	—	1	—
3	Minor carbohydrate metabolism	1	—	—	—	—	—	—	—
4	Glycolysis	—	—	—	—	—	—	—	—
5	Fermentation	—	—	—	—	—	—	—	—
6	Gluconeogenese/glyoxylate cycle	—	—	—	—	—	—	—	—
7	OPP cycle	—	—	—	—	—	—	—	—
8	TCA/orgin acid transformation	—	—	—	—	—	—	—	—
9	Mitochondrial electron transport/ATP synthesis	—	—	—	—	—	—	—	—
10	Cell wall	—	—	—	—	—	—	—	—
11	Lipid metabolism	—	—	—	—	—	—	—	—
12	N-metabolism	—	—	—	—	—	—	—	—
13	Amino acid metabolism	—	—	5	11	—	—	1	—
14	S-assimilation	—	—	—	—	—	—	—	—
15	Metal handling	—	—	1	11	—	—	1	—
16	Secondary metabolism	—	—	23	32	1	6	4	2
17	Hormone metabolism	—	—	32	78	—	2	4	8
18	Co-factor and vitamin metabolism	—	—	3	1	—	—	1	—
19	Tetrapyrrole synthesis	—	—	1	—	—	—	—	—
20	Stress	1	—	25	49	—	3	4	3
21	Redox regulation	—	—	12	7	—	2	1	1
22	Polyamine metabolism	—	—	1	1	—	—	—	—
23	Nucleotide metabolism	—	—	4	7	—	—	—	1
24	Biodegradation of xenobiotics	—	—	3	1	—	—	—	—
25	C1-metabolism	—	—	1	3	—	—	—	—
26	Miscellaneous	—	—	39	93	1	6	6	3
27	RNA	1	—	57	202	3	4	20	32
28	DNA	—	—	5	25	—	2	3	—
29	Protein	—	—	77	138	8	5	13	19
30	Signaling	—	—	25	125	4	2	10	12
31	Cell	—	—	14	46	1	2	4	4
32	Micro RNA, natural antisense	—	—	—	—	—	—	—	—
33	Development	—	—	21	37	—	3	1	9
34	Transport	—	—	31	48	2	1	6	5
35	Not assigned	—	—	147	301	13	21	31	45
36	C4-photosynthesis	—	—	—	—	—	—	—	—

**Figure 2:**
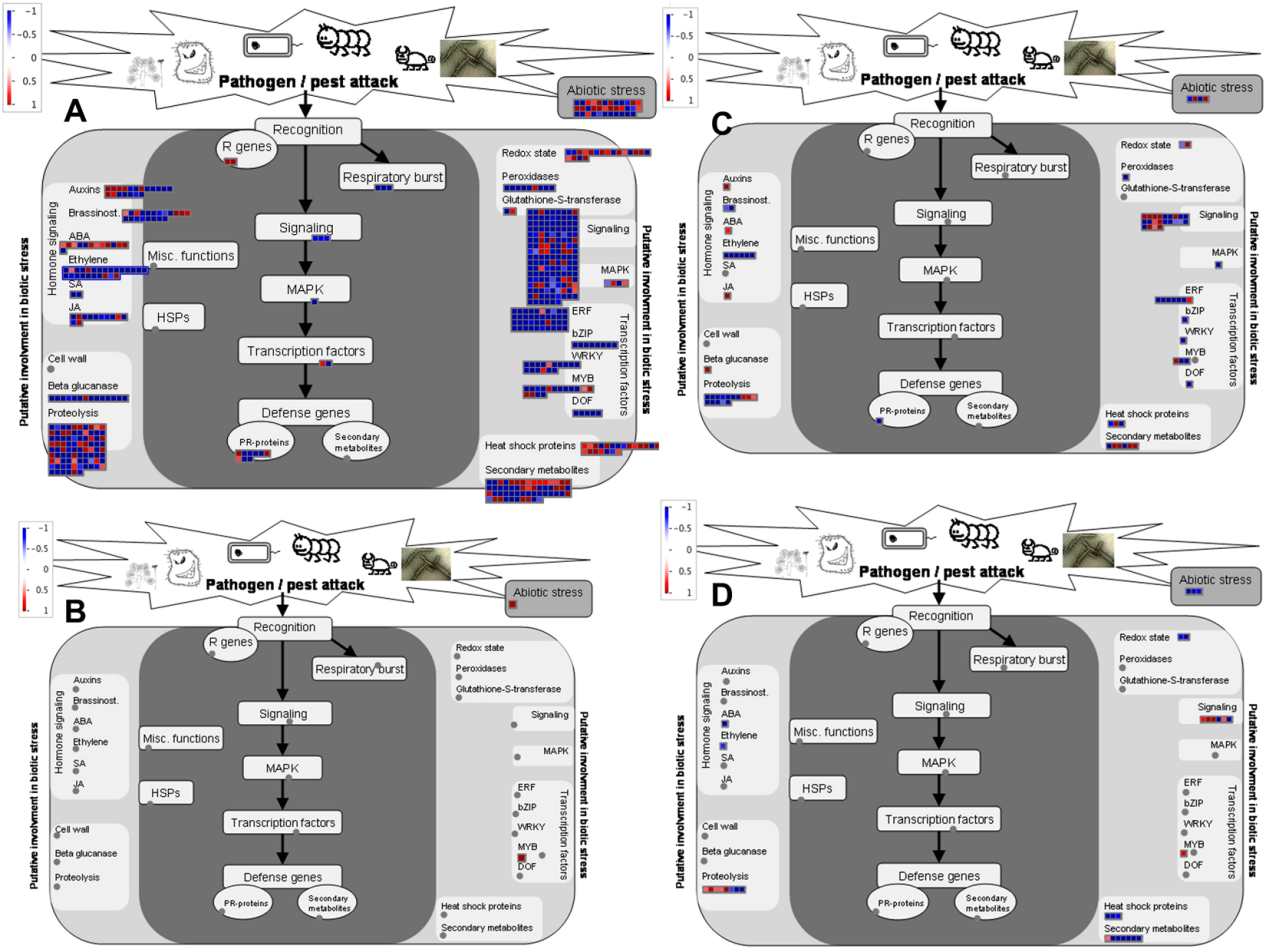
Diagram showing differentially expressed genes in BXW-resistant and BXW-susceptible genotypes of banana after inoculation with *Xanthomonas campestris* pv. *musacearum* mapped onto known biotic stress pathway bins using Mapman. (**A)** BXW–resistant genotype *Musa balbisiana* at 12 hpi, (**B)** BXW-susceptible genotype Pisang Awak at 12 hpi, (**C)** BXW–resistant genotype *Musa balbisiana* at 48 hpi, (**D)** BXW-susceptible genotype Pisang Awak at 48 hpi. Figures were generated from Mapman. The scale is from down-regulated (blue) to up-regulated (red).

More number of genes were differentially expressed in BXW-resistant genotype in comparison to BXW-susceptible genotype due to interaction with Xcm at different time post inoculation (Table 1). The overview of genes mapped onto the biotic stress pathway at 12 hpi and 48 hpi for BXW-resistant and BXW-susceptible genotypes is illustrated in Fig. 2.

Stress associated genes were differentially expressed in both banana genotypes in response to Xcm. About 25 genes associated with stress were up-regulated in *Musa balbisiana* at 12 hpi, whereas only one gene, Germin-like protein (GER1), was activated in Pisang Awak at 12 hpi (Tables 1 and 2). Germin-like protein was also up-regulated in *Musa balbisiana* at 48 hpi. All stress-associated genes were suppressed in Pisang Awak at 48 hpi (Table 1).

**Table 2:** Details of the major differentially expressed genes (DEGs) in BXW-resistant banana genotype *Musa balbisiana* and BXW-susceptible banana genotype Pisang Awak upon pathogen attack.

Gene ID from *Musa acuminata* (DH Pahang)	Gene ID from *Musa balbisiana* (Pisang Klutuk Wulung)	Log2 Fold Change	Description
**Pisang Awak at 12 hpi**			
**Stress-related**			
Ma02_g20530	ITC1587_Bchr2_P04749	17.87	Germin-like protein 8–14, similar to germin 3 of *Arabidopsis thaliana*
**Transcription factors**			
Ma05_g25630	ITC1587_Bchr5_P14199	18.46	MYB family transcription factor
***Musa balbisiana*** **at 12 hpi**			
**Disease Resistance and Pathogenesis-Related Protein**			
Ma09_g22650	ITC1587_Bchr9_P27517*	1.75	Probable leucine-rich repeat (LRR) disease resistance family protein
Ma09_g28690	ITC1587_Bchr9_P28091	1.05	Putative disease resistance protein RPM1, confers resistance to *Pseudomonas syringae* pv. *maculicola* 1
Ma04_g38470	ITC1587_Bchr4_P11526	4.6	Pathogenesis-related protein R major form precursor, involves in defense response to bacterium
Ma09_g05410	ITC1587_Bchr9_P25531*	2.52	Pathogenesis-related family protein
Ma05_g02080	ITC1587_Bchr5_P11835	0.68	NPR1-like protein 3 (NPR3), involves in defense response to bacterium
Ma03_g14510	ITC1587_Bchr3_P06402	−0.83	MLO-like protein 13
**Heat shock protein**			
Ma09_g29150	ITC1587_Bchr9_P28137	3.36	Putative 26.5 kDa heat shock protein, HSP20-like chaperones, involves in response to response to hydrogen peroxide
Ma08_g24440	ITC1587_Bchr8_P24119	1.16	Class III heat shock protein
Ma01_g14540	ITC1587_Bchr1_P01860*	2.02	Class I heat shock protein, HSP20-like chaperones
Ma01_g10350	ITC1587_Bchr1_P01490	1.19	Putative small heat shock protein
Ma06_g13850	ITC1587_Bchr6_P15877	1.2	Stromal 70 kDa heat shock-related protein
Ma10_g14770	ITC1587_Bchr10_P30216	1.42	BAG family molecular chaperone regulator 6-like, involves in apoptosis after pathogen attack
**Transcription factors**			
Ma04_g05410	ITC1587_Bchr4_P08866	3.0	MADS-box transcription factor family protein AGL61
Ma08_g04270	ITC1587_Bchr8_P21825	1.35	MADS-box transcription factor 14 (OsMADS14), APETALA1-like B
Ma09_g08260	ITC1587_Bchr9_P25771	1.31	MyB-related protein 308-like, encodes a R2R3 MYB protein, myb domain protein 4 (MYB4)
Ma10_g16050	ITC1587_Bchr10_P30324*	1.05	Transcription repressor MYB4, encodes a R2R3 MYB
Ma08_g20860	ITC1587_Bchr8_P23775	1.12	Transcription factor MYB1R1-like
Ma02_g23870	ITC1587_Bchr2_P05052	0.95	Putative Myb-related protein 306, encodes a R2R3 type Myb, myb domain protein 96 (MYB96)
Ma01_g01800	ITC1587_Bchr1_P00594	0.60	MYB-related transcription factor family
Ma04_g14250	ITC1587_Bchr4_P09682	0.65	WRKY transcription factor 4
Ma09_g06980	ITC1587_Bchr6_P15345*	0.79	WRKY transcription factor 75
Ma10_g30810	ITC1587_Bchr10_P31614	2.18	C2H2 zinc finger protein 4-like
Ma10_g07810	ITC1587_Bchr10_P29542	1.19	C2H2 zinc finger family
Ma09_g04220	ITC1587_Bchr9_P25437	1.32	Floral homeotic protein APETALA 2-like, AP2.7
Ma03_g05720	ITC1587_Bchr3_P05677	0.64	Putative Ethylene-responsive transcription factor 1
Ma07_g22730	ITC1587_Bchr7_P20906	1.86	Basic Helix-Loop-Helix family
Ma03_g17120	ITC1587_Bchr3_P06850	1.95	Heat shock transcription factor C1 (HSFC1)
Ma10_g27770	ITC1587_Bchr10_P31333	1.67	Heat shock transcription factor C1 (HSFC1)
Ma01_g09550	ITC1587_Bchr1_P01419*	2.16	GRAS transcription factor family
Ma06_g09030	ITC1587_Bchr6_P15445	1.31	Transcription factor, Auxin Response Factor (ARF)
Ma04_g37190	ITC1587_Bchr4_P11429	1.18	Zinc finger protein CONSTANS-LIKE 16-like, transcription factor C2C2(Zn) CO-like
Ma11_g07820	ITC1587_Bchr11_P32446	1.01	Transcription factor C2C2(Zn) CO-like
Ma04_g07150	ITC1587_Bchr4_P09012	2.02	Homeobox transcription factor family
Ma06_g08780	ITC1587_Bchr6_P15417	1.0	Homeobox transcription factor family
Ma09_g14370	ITC1587_Bchr9_P26312	1.19	Homeobox transcription factor family
**Receptor-like kinases**			
Ma02_g12690	ITC1587_Bchr2_P04089*	4.90	Wall-associated kinase 2 (WAK2)
Ma02_g12710	ITC1587_Bchr2_P04072*	4.17	Wall associated kinase 5 (WAK5)
Ma04_g18790	ITC1587_Bchr4_P10336	3.58	Leucine-rich repeat receptor-like protein kinase family
Ma08_g08120	ITC1587_Bchr8_P22166	1.3	Leucine-rich receptor-like protein kinase HSL1
Ma09_g08850	ITC1587_Bchr9_P25823	1.16	LRR receptor-like serine/threonine-protein kinase
Ma10_g09280	ITC1587_Bchr7_P20253*	0.91	Leucine-rich repeat transmembrane protein kinase
Ma03_g30610	ITC1587_Bchr3_P08112*	2.12	Receptor-like protein kinase, wheat LRK10 like
Ma01_g14090	ITC1587_Bchr1_P00623*	0.92	Receptor-like protein kinase DUF 26
**Signaling pathways**			
Ma04_g04500	ITC1587_Bchr4_P08787	0.89	Mitogen-activated protein kinase 2
Ma02_g08740	ITC1587_Bchr2_P03721	2.23	Phosphatidylinositol 4-phosphate 5-kinase 6-like
Ma05_g17450	ITC1587_Bchr5_P13538	0.91	Phosphatidylinositol-4-phosphate 5-kinase
Ma05_g06670	ITC1587_Bchr5_P12277	1.07	G-proteins rho guanine nucleotide exchange factor 8-like
Ma08_g10000	ITC1587_Bchr8_P22338*	0.97	Putative protein pleiotropic regulator PRL2, essential for plant innate immunity
Ma09_g08850	ITC1587_Bchr9_P25823	0.99	Nodulation receptor kinase-like
Ma10_g09280	ITC1587_Bchr7_P20253*	0.91	Leucine-rich repeat transmembrane protein kinase
Ma08_g08120	ITC1587_Bchr8_P22166	1.31	Leucine-rich receptor-like protein kinase family protein HSL1
Ma10_g14170	ITC1587_Bchr10_P30158	1.37	Pseudo histidine-containing phosphotransfer protein 2
**Redox regulation**			
Ma06_g26670	ITC1587_Bchr6_P17425	1.88	Polyamine oxidase-like
Ma01_g07400	ITC1587_Bchr6_P17629*	3.21	Glutaredoxin family GRX480 involved in SA/JA cross-talk
Ma10_g29540	ITC1587_Bchr10_P31495	1.09	WCRKC thioredoxin 1 involved in cell redox homeostasis
Ma01_g02150	ITC1587_Bchr1_P00623*	1.49	Ascorbate-specific transmembrane electron transporter 1
Ma10_g11130	ITC1587_Bchr10_P29903	0.99	GDP-L-galactose phosphorylase 1, defense response to bacterium by callose deposition in cell wall
Ma10_g15710	ITC1587_Bchr10_P30300	1.04	Heme-binding-like protein
Ma05_g15800	ITC1587_Bchr5_P13135	1.23	Catalase isozyme 2, a peroxisomal catalase
Ma11_g19280	ITC1587_Bchr11_P33899	1.68	1-Cys peroxiredoxin (PER1)
Ma06_g19640	ITC1587_Bchr6_P16418	1.31	Glutaredoxin involved in cell redox homeostasis
**Hormone metabolism**			
***Absciscisic acid (ABA)***			
Ma04_g19490	ITC1587_Bchr4_P10415	1.29	Putative probable carotenoid cleavage dioxygenase 4
Ma03_g33240	ITC1587_Bchr3_P08330	0.94	Protein phosphatase 2C 51
Ma03_g18730	ITC1587_BchrUn_random_P38210	1.21	Probable protein phosphatase 2C 9
Ma07_g20270	ITC1587_Bchr7_P20689	0.64	Zeaxanthin epoxidase involved in first step of ABA biosynthesis
Ma04_g10140	ITC1587_Bchr4_P09286	0.61	Cytosolic short-chain dehydrogenase/reductase involved in the conversion of xanthoxin to ABA-aldehyde during ABA biosynthesis
Ma03_g06930	ITC1587_Bchr3_P05785	0.75	Leucine zipper transcription factor that binds to ABA responsive element (ABRE) motif in the promoter region of ABA-inducible genes
Ma04_g39350	ITC1587_Bchr4_P11595	3.36	Ninja-family protein AFP3-like
***Ethylene***			
Ma06_g14430	ITC1587_BchrUn_random_P38187	5.40	1-aminocyclopropane-1-carboxylate oxidase
Ma11_g16580	ITC1587_Bchr11_P33656*	2.05	Ethylene receptor
Ma04_g03160	ITC1587_BchrUn_random_P39147*	1.16	Methylesterase 3
***Auxin***			
Ma01_g11950	ITC1587_Bchr1_P01647	3.06	Auxin-induced protein 15A-like
Ma06_g06990	ITC1587_Bchr6_P15254	1.46	UDP-glucose:indole-3-acetate beta-D-glucosyltransferase
Ma06_g07000	ITC1587_Bchr6_P15254*	1.45	UDP-glucosyltransferase
Ma06_g07010	ITC1587_Bchr6_P15256	1.067	UDP-glucosyltransferase
Ma05_g18810	ITC1587_Bchr5_P13157*	1.34	IN2-2 protein-like
***Jasmonic acid***			
Ma03_g11520	ITC1587_Bchr3_P06219	1.53	Linoleate 13S-lipoxygenase 2-1 required for wound-induced jasmonic acid accumulation
Ma07_g12340	ITC1587_Bchr7_P19705	1.10	Protein TIFY 6b involved in jasmonic acid mediated signaling pathway
**Secondary Metabolites**			
Ma06_g06860	ITC1587_BchrUn_random_P38862	5.46	Geranylgeranyl pyrophosphate synthase involved in isoprenoid biosynthesis
Ma06_g25700	ITC1587_BchrUn_random_P39673	1.39	Terpenoid cyclases, alpha-terpineol synthase
Ma04_g12630	ITC1587_Bchr4_P09530	1.37	Probable homogentisate phytyltransferase 1 involved in tocopherol biosynthesis
Ma04_g33020	ITC1587_Bchr4_P11074	2.30	Cinnamyl alcohol dehydrogenase 1 (CAD1) involved in lignin biosynthetic process
Ma07_g07170	ITC1587_Bchr7_P19196	5.04	Mannitol dehydrogenase
Ma02_g17740	ITC1587_Bchr2_P04501	1.11	UDP-glycosyltransferase 73C2
Ma05_g05930	ITC1587_Bchr5_P12213	1.92	UDP-glycosyltransferase 73C6-like
Ma11_g02650	ITC1587_Bchr11_P31934	1.39	Naringenin, 2-oxoglutarate 3-dioxygenase, involved in response to fungus and bacterium, flavonoid biosynthetic process
Ma04_g33310	ITC1587_Bchr4_P11096	1.60	Malonyl-coenzyme A: anthocyanin 3-O-glucoside-6”-O-malonyltransferase
ma06_g13940	ITC1587_Bchr6_P15886	1.28	Nicotinamidase 1 (NIC1) involved in the pyridine nucleotide salvage pathway which is connected to the de novo NAD biosynthesis through the ABA signaling pathway
**Transporters**			
Ma08_g00630	ITC1587_Bchr8_P21529	−7.31	Bidirectional sugar transporter SWEET14-like
Ma11_g13300	ITC1587_Bchr11_P33322	−5.98	Early nodulin-like protein 3
Ma06_g07960	ITC1587_Bchr6_P15344	−1.91	Early nodulin-like protein 1
**E3 ubiquitin ligase**			
Ma07_g03310	ITC1587_Bchr7_P18846	−2.99	E3 ubiquitin-protein ligase PUB23-like
Ma07_g03320	ITC1587_Bchr7_P18846*	−4.02	E3 ubiquitin-protein ligase PUB22
Ma05_g17000	ITC1587_BchrUn_random_P35437	−0.93	E3 ubiquitin-protein ligase RMA1H1-like
Ma05_g16120	ITC1587_BchrUn_random_P35166	−2.08	E3 ubiquitin-protein ligase RHA1B-like
Ma09_g05070	ITC1587_Bchr9_P25505	−2.73	E3 ubiquitin-protein ligase RHA2B
Ma08_g12080	ITC1587_Bchr8_P22539	−4.23	RING-H2 finger protein ATL1-like
Ma03_g14160	ITC1587_BchrUn_random_P35655	−1.10	RING-H2 finger protein ATL16-like
Ma10_g24780	ITC1587_Bchr10_P31078	−1.26	E3 ubiquitin-protein ligase ATL6-like
Ma07_g16650	ITC1587_Bchr7_P20364	−1.55	RING-H2 finger protein ATL2-like
Ma04_g27580	ITC1587_Bchr5_P13665*	−1.56	E3 ubiquitin-protein ligase ATL4-like
**Miscellaneous**			
Ma04_g03310	ITC1587_Bchr4_P08682	1.23	Glucan endo-1,3-beta-glucosidase
Ma06_g08050	ITC1587_Bchr6_P15353	1.18	Peptidase family M48 involved in proteolysis
Ma07_g03700	ITC1587_Bchr7_P18873	1.24	Allantoate deiminase involved in proteolysis
Ma11_g20890	ITC1587_Bchr11_P34045	1.03	Serine carboxypeptidase-like 18, involved in proteolysis
Ma03_g11210	ITC1587_Bchr3_P06202*	1.46	Subtilisin-like protease involved in proteolysis
Ma04_g35970	ITC1587_Bchr4_P11328	2.26	Subtilisin-like protease involved in proteolysis
Ma11_g20890	ITC1587_Bchr11_P34045	1.03	Serine carboxypeptidase-like 18, involved in proteolysis
Ma08_g04840	ITC1587_Bchr8_P21878	1.45	Zinc metalloprotease EGY3, involved in response to hydrogen peroxide
**Anti-microbial**			
Ma03_g31180	ITC1587_Bchr3_P08153	5.44	Vicilin-like antimicrobial peptides 2–2
**Pisang Awak at 48 hpi**			
**Transcription factors**			
Ma10_g18840	ITC1587_Bchr10_P30560*	0.88	Transcription factor MYB88
**Receptor-like kinases**			
Ma08_g16960	ITC1587_BchrUn_random_P38061*	1.4	LRR receptor-like serine/threonine-protein kinase
Ma07_g16540	ITC1587_BchrUn_random_P38061	1.21	LRR transmembrane protein kinase, SHR5-receptor-like kinase
Ma04_g24750	ITC1587_Bchr1_P00475	0.78	LRR receptor-like serine/threonine-protein kinase
**Signaling pathways**			
Ma03_g07190	ITC1587_Bchr10_P30745*	0.64	G protein, translation factor GUF1 homolog
**E3 ubiquitin ligase**			
Ma04_g13930	ITC1587_Bchr4_P09646	−1.04	Putative E3 ubiquitin ligase BIG BROTHER/
**Miscellaneous**			
Ma03_g26960	ITC1587_Bchr3_P07815	0.61	Serine carboxypeptidase-like 18, involved in proteolysis
Ma03_g29980	ITC1587_Bchr3_P08046*	0.96	Carboxyl-terminal peptidase
Ma11_g15300	ITC1587_Bchr5_P13764*	0.62	Prolyl Oligopeptidase, involved in proteolysis
Ma03_g26960	ITC1587_Bchr3_P07815	0.61	Serine carboxypeptidase-like, involved in proteolysis
Ma09_g06030	ITC1587_Bchr9_P25584	1.40	Ubiquitin-conjugating enzyme E2
***Musa balbisiana*** **at 48 hpi**			
**Stress-related**			
Ma02_g20530	ITC1587_Bchr2_P04749	16.16	Germin-like protein 8–14
**Disease resistance (R) and Pathogenesis-related (PR)**			
Ma06_g14360	ITC1587_Bchr6_P15924	1.94	Lipase-like PAD4
**Heat Shock**			
Ma08_g29620	ITC1587_Bchr8_P24584*	3.03	Class IV heat shock protein-like
**Transcription factors**			
Ma01_g17260	ITC1587_Bchr1_P02266	3.12	Transcription factor MYB86, myb domain protein 61 (MYB61)
Ma01_g18330	ITC1587_Bchr1_P02164	1.92	Trihelix transcription factor GTL1-like
Ma09_g16130	ITC1587_BchrUn_random_P36803	0.84	AP2-like ethylene-responsive transcription factor
Ma07_g04810	ITC1587_Bchr7_P18970	1.33	Transcription factor MYC2, basic helix-loop-helix (bHLH) DNA-binding family protein
Ma06_g05210	ITC1587_Bchr6_P15106	0.92	Putative ZOS4-07 - C2H2 zinc finger protein
**Receptor-like kinases**			
Ma03_g20500	ITC1587_Bchr3_P07270	1.86	LRR receptor-like serine/threonine-protein kinase RPK2
Ma04_g39220	ITC1587_Bchr4_P11583	1.86	Receptor-like protein kinase
Ma09_g12980	ITC1587_Bchr9_P26183	1.39	LRR receptor-like serine/threonine-protein kinase
**Signaling pathways**			
Ma02_g10140	ITC1587_Bchr2_P03841*	2.59	Zinc finger Ran-binding domain-containing protein 2-like
Ma02_g02010	ITC1587_BchrUn_random_P34585		
Ma02_g24350	ITC1587_Bchr2_P05093	1.18	DELLA protein SLR1
Ma09_g24360	ITC1587_Bchr9_P27692	1.0	Glutamate receptor 3.3
**Redox regulation**			
Ma03_g19080	ITC1587_Bchr3_P07100	1.27	Superoxide dismutase, defense response to bacterium
**Hormone**			
***Absciscisic acid (ABA)***			
Ma07_g00090	ITC1587_Bchr7_P18560	0.76	HSI2-like 1 (HSL1), B3 domain-containing protein
***Jasmonic acid***			
Ma03_g11520	ITC1587_Bchr3_P06219	1.34	Linoleate 13S-lipoxygenase 2-1
***Auxin***			
Ma06_g01320	ITC1587_Bchr11_P32703	6.78	Putative auxin efflux carrier component 8
**Secondary Metabolites**			
Ma09_g29570	ITC1587_Bchr9_P28175	1.46	Nicotianamine aminotransferase A involve in tocopherol biosynthesis
Ma08_g04050	ITC1587_Bchr8_P21808	3.76	Putative 3-ketoacyl-CoA synthase 17 involve in wax metabolism
Ma10_g20730	ITC1587_Bchr10_P30723	1.28	Tryptophan aminotransferase-related protein 2 involved in IAA biosynthesis
**E3 ubiquitin ligase**			
Ma06_g35270	ITC1587_Bchr6_P18218	0.93	E3 ubiquitin-protein ligase SDIR1
**Miscellaneous**			
Ma09_g26260	ITC1587_Bchr9_P27875	1.05	Glucan endo-1,3-beta-glucosidase 8

Note: Gene IDs from *Musa balbisiana* are based on the results of the reciprocal best BLAST search. The best BLAST hit is reported for the genes where the reciprocal best BLAST search hit was not available and it is denoted with*.

Several pathogenesis-related (PR) proteins were up-regulated and also many PR genes were suppressed in response to pathogen interaction with *Musa balbisiana* at 12 hpi (Fig. 2, Table 2).

Disease resistance (R) gene in leucine-rich repeat (LRR) family protein and putative disease resistance protein RPM1 were activated in *Musa balbisiana* as early response to pathogen at 12 hpi (Fig. 2, Table 2). Lipase-like PAD4 gene was also activated in *Musa balbisiana* at 48 hpi (Table 2). None of the R or PR genes were differentially expressed in BXW-susceptible genotype Pisang Awak as early response to pathogen infection (Fig. 2).

Transcript aligned with MLO-like protein 13 (MILDEW RESISTANCE LOCUS O 13) was suppressed in BXW-resistant *Musa balbisiana* at 12 hpi (Table 2).

Heat shock related transcripts were differentially expressed in *Musa balbisiana* at 12 hpi (Fig. 2). HSP20-like chaperones superfamily associated genes were up-regulated in *Musa balbisiana* at 12 hpi and as well as at 48 hpi (Table 2). However, genes associated with heat shock proteins were either not differentially expressed or suppressed in Pisang Awak at 12 hpi and 48 hpi respectively (Fig. 2).

Transcript associated with apoptosis regulator, BAG family molecular chaperone regulator 6-like was activated in *Musa balbisiana* upon pathogen attack at 12 hpi (Table 2).

The genes related to transcription factors were differentially expressed in both genotypes at 12 hpi and 48 hpi (Fig. 2, Table 2). The genes associated with transcription factors like MADS-box transcription factor family, MYB, WRKY, C2H2 zinc finger family, AP2/EREBP, heat shock transcription factor C1 (HSFC1), GRAS transcription factor family, auxin response factor (ARF) family, C2C2 family, homeobox transcription factor family and basic helix-loop-helix family (bHLH) were activated in *Musa balbisiana* at 12 hpi. Some of the genes associated with transcription factors like MYB, trihelix, AP2/EREBY), bHLH and C2H2 were also up-regulated in *Musa balbisiana* at 48 hpi. The MYB transcription factor was also activated in Pisang Awak at both 12 hpi and 48 hpi (Fig. 2, Table 2).

**Figure 3:**
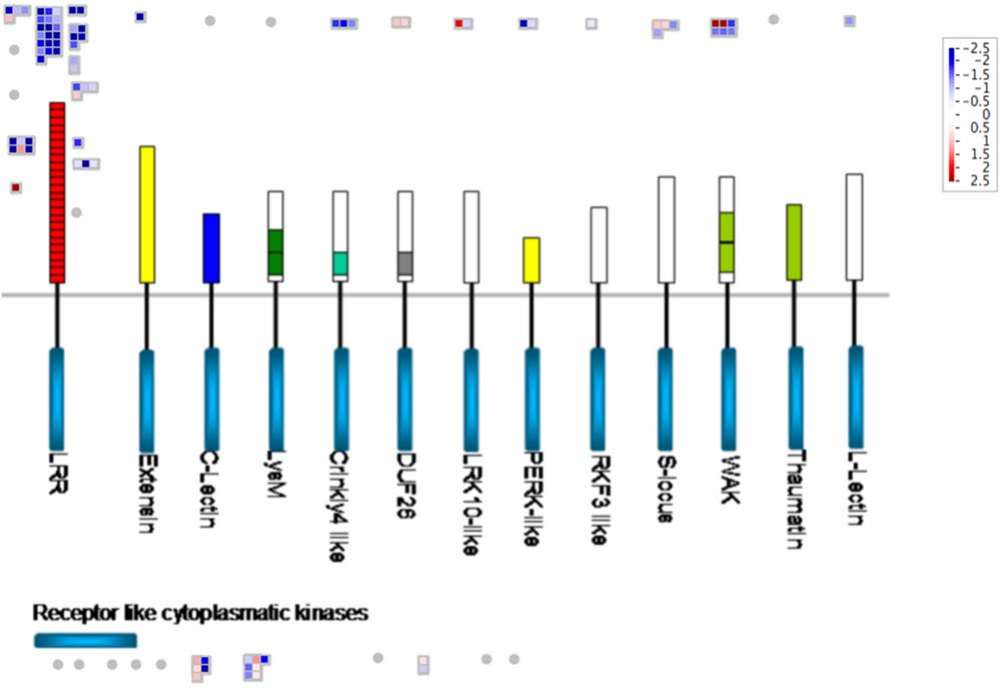
Diagram showing mapping of the transcripts associated with receptor-like-kinases in the BXW-resistant genotype *Musa balbisiana* at 12 hours post-inoculation with *Xanthomonas campestris* pv. *musacearum*. Figure was generated from Mapman. The scale is from down-regulated (blue) to up-regulated (red).

As early response to pathogen interaction in *Musa balbisiana*, several receptor-like kinases such as wall associated receptor kinases, LRR receptor kinases, LRK10 like receptor kinase, receptor kinase DUF26, were up-regulated at 12 hpi and similarly receptor kinases were also activated at 48 hpi (Fig. 3, Table 2). Other genes involved in signaling pathways like mitogen-associated protein kinase (MAPK), phosphatidylinositol-4-phosphate 5-kinase family protein, G protein were also found to be up-regulated in *Musa balbisiana* (Table 2). However, none of signaling pathway-associated gene was differentially expressed in Pisang Awak at 12 hpi, whereas LRR receptor kinases and G protein were up-regulated at 48 hpi (Table 2).

Transcripts involved in redox regulation like glutaredoxins, peroxiredoxin, thioredoxins, ascorbate, glutathione, catalases and heme family were up-regulated in *Musa balbisiana* at 12 hpi (Fig. 2, Table 2). Also superoxide dismutase gene was induced in *Musa balbisiana* as response to pathogen interaction at 48 hpi. However, upon pathogen infection either the redox state was not affected or suppressed in Pisang Awak (Fig. 2). Peroxidases and glutathione S transferases (GSTs) were also differentially expressed in *Musa balbisiana* at 12 hpi. Genes associated with oxidative burst such as polyamine oxidase-like gene was induced in *Musa balbisiana* as early response (12 hpi) to Xcm.

As a result of plant-pathogen interaction, hormone metabolic pathways were activated in *Musa balbisiana*. Abscisic acid (ABA) metabolism was activated in *Musa balbisiana* at both 12 and 48 hpi (Fig. 2, Table 2). However, genes associated with ABA metabolism were not differentially expressed in Pisang Awak at 12 hpi and suppressed at 48 hpi (Fig. 2, Table 2). Genes associated with ninja family protein AFP3 was activated in *Musa balbisiana* at 12 hpi. Genes associated with ethylene synthesis and signal transductions were differentially expressed in *Musa balbisiana* at 12 hpi (Table 2). Ethylene pathway was suppressed in both genotypes at 48 hpi. Several genes associated with jasmonate (JA) metabolism were up-regulated in *Musa balbisiana* at both 12 hpi and 48 hpi (Table 2). The salicylic acid (SA) pathway was repressed in *Musa balbisiana* at 12 hpi (Fig. 2). However, no change was observed in JA and SA metabolism in Pisang Awak. Several genes associated with auxin metabolism were up-regulated in *Musa balbisiana* at both 12 hpi and 48 hpi (Table 2).

Genes associated with beta-glucanases were found to be differentially expressed in *Musa balbisiana*. Only one transcript associated with β*-*1,3-glucan hydrolase was activated in *Musa balbisiana* at 12 hpi and 48 hpi (Table 2). All other β-1,3 glucan hydrolase genes were suppressed in *Musa balbisiana* at 12 hpi. Upon pathogen attack, transcripts associated with proteolysis were also differentially expressed in *Musa balbisiana* at both 12 hpi and 48 hpi and Pisang Awak at 48 hpi.

**Figure 4:**
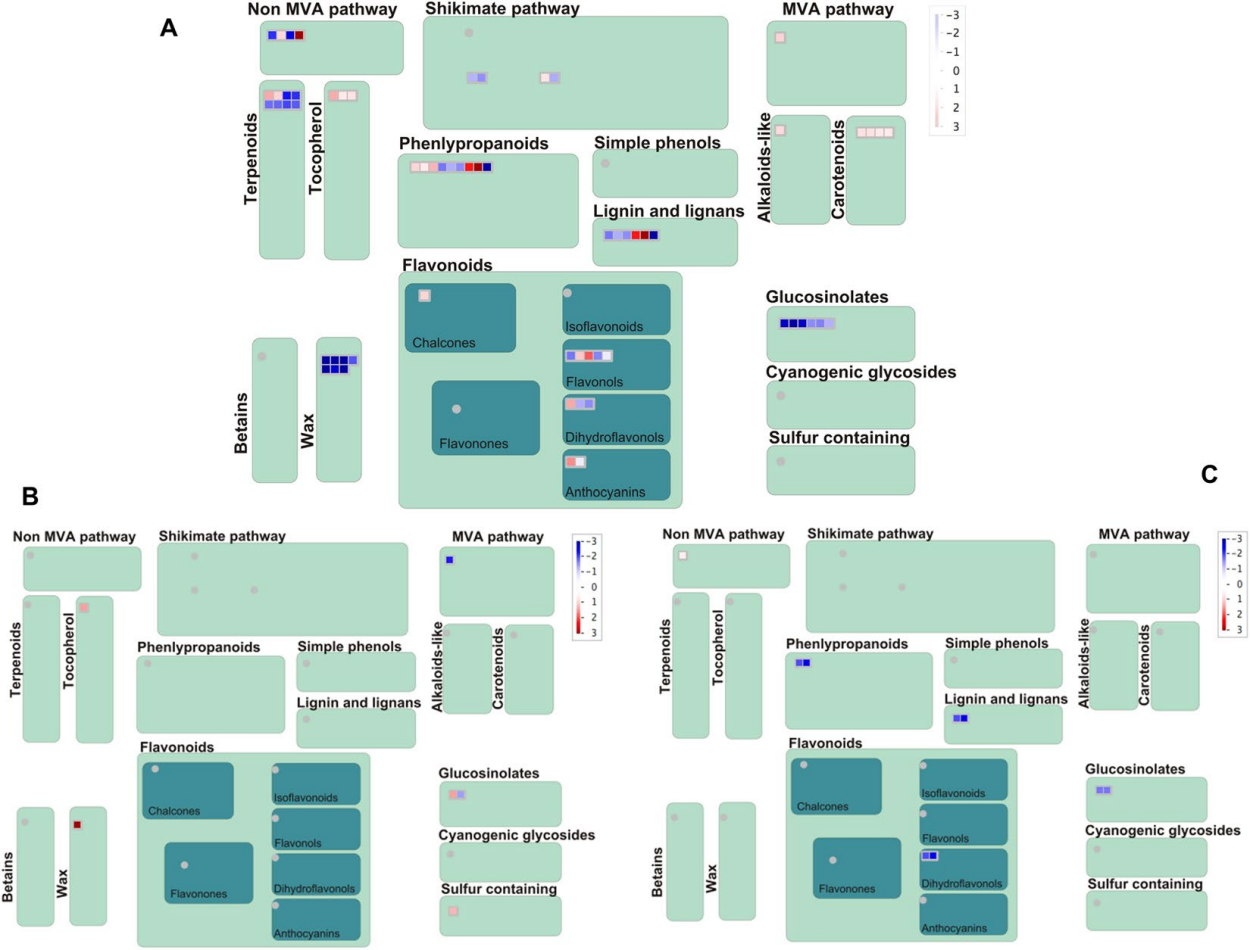
Diagram showing differentially expressed genes mapped to secondary metabolic pathways in BXW-resistant and BXW-susceptible genotypes of banana in response to interaction with *Xanthomonas campestris* pv. *musacearum*. (**A)** BXW–resistant genotype *Musa balbisiana* at 12 hpi, (**B)** BXW–resistant genotype *Musa balbisiana* at 48 hpi, (**C)** BXW-susceptible genotype Pisang Awak at 48 hpi. Figures were generated from Mapman. The scale is from down-regulated (blue) to up-regulated (red).

Also, production of several secondary metabolites was activated in *Musa balbisiana* in response to pathogen infection at both 12 hpi and 48 hpi. Activation of isoprenoids, lignin biosynthesis, phenylpropanoids, flavonoids, terpenoids was observed in *Musa balbisiana* at 12 hpi (Fig. 4, Table 2). The wax synthesis was activated at 48 hpi and suppressed at 12 hpi in *Musa balbisiana*. Lignin biosynthesis, phenylpropanoids, flavonoids pathways were suppressed in Pisang Awak (Fig. 4).

In response to bacterial infection, vicilin-like antimicrobial peptide was activated in *Musa balbisiana* at 12 hpi (Table 2).

Genes associated with transporters of peptides and oligopeptides, proteins, aminoacids, sugars, metals, potassium, metabolites and ABC transporters and multidrug resistance systems were differentially expressed in *Musa balbisiana* at 12 hpi and 48 hpi. Gene associated with bidirectional sugar transporter SWEET14-like was significantly suppressed in *Musa balbisiana* at 12 hpi (Table 2). Transporters for v-ATPases and ABC transporters and multidrug resistance systems were also differentially expressed in Pisang Awak at 48 hpi.

Early nodulin like proteins were suppressed in *Musa balbisiana* at 12 hpi (Table 2).

Genes associated with E3 ubiquitin-protein ligases were differentially expressed in *Musa balbisiana* at 12 hpi and 48 hpi (Table 2). Several of the E3 ubiquitin ligases such as PUB22, PUB23, RMA1H1, Ring 1, ATL-4, RHA1B, RHA2B, were down-regulated in *Musa balbisiana*. E3 ubiquitin ligase BIG BROTHER-like was down-regulated in Pisang Awak at 48 hpi.

Genes involved in metabolic pathways like cytochrome P450 were found to be up-regulated as well as down-regulated in *Musa balbisiana* at both 12 hpi and 48 hpi. However cytochrome P450 pathway was suppressed in Pisang Awak at 48 hpi.

### Validation of RNA-Seq data for selected genes by qRT-PCR analysis

The results of RNA-Seq analysis was validated by quantitative RT- PCR (qRT-PCR) using a set of 30 annotated genes differentially expressed in response to Xcm infection in *Musa balbisiana* and Pisang Awak (Table 3). The differential expression was confirmed by qRT-PCR for some of the genes involved in plant defense such as antimicrobial peptide vicilin, R genes (RPM1, LRR disease resistance family), PR genes, transcription factors (MYB, MADS-box, WRKY), receptor like kinases (wall associated receptor kinases and LRR receptor kinase), redox associated genes (polyamine oxidases), genes involved in signaling pathways (phosphatidylinositol 4-phosphate 5-kinase 6 like), E3 ubiquitin ligase and germin-like protein. The expression of genes involved in hormone metabolism such as ABA pathway (carotenoid cleavage dioxygenase 4), ethylene pathway (1-aminocyclopropane-1-carboxylate oxidase), auxin metabolism (auxin-induced protein 15A-like) and jasmonic acid pathway (linoleate 13S-lipoxygenase 2-1), alpha-galactosidase, sugar transporter gene (SWEET14), early nodulin-like protein, and genes involved in secondary metabolites like lignin biosynthesis (cinnamyl alcohol dehydrogenase 1) and starch biosynthesis (glucose-1-phosphate adenylyltransferase large subunit 1-like) was also corroborated.

**Table 3:** Details of differentially expressed genes (DEGs) used for qRT-PCR analysis for validation of RNA-Seq fold changes in Pisang Awak and *Musa balbisiana* in response to artificial inoculation with *Xanthomonas campestris* pv. *musacearum*.

	Gene ID from *Musa acuminata* (DH Pahang)	Gene ID from *Musa balbisiana* (Pisang Klutuk Wulung)	Description	Genotype and time point
1	Ma02_g20530	ITC1587_Bchr2_P04749	Germin-like protein 8–14	Pisang Awak at 12 hpi
2	Ma05_g25630	ITC1587_Bchr5_P14199	MYB family transcription factor	Pisang Awak at 12 hpi
3	Ma02_g12480	ITC1587_Bchr1_P00003*	Alpha-galactosidase-like	Pisang Awak at 12 hpi
4	Ma06_g26670	ITC1587_Bchr6_P17425	Polyamine oxidase-like	*Musa balbisiana* at 12 hpi
5	Ma03_g31180	ITC1587_Bchr3_P08153	Vicilin-like antimicrobial peptides 2–2	*Musa balbisiana* at 12 hpi
6	Ma09_g22650	ITC1587_Bchr9_P27517*	Leucine-rich repeat (LRR) disease resistance family protein	*Musa balbisiana* at 12 hpi
7	Ma09_g28690	ITC1587_Bchr9_P28091	Putative disease resistance protein RPM1	*Musa balbisiana* at 12 hpi
8	Ma04_g38470	ITC1587_Bchr4_P11526	Pathogenesis-related R protein	*Musa balbisiana* at 12 hpi
9	Ma09_g05410	ITC1587_Bchr9_P25531*	Pathogenesis-related family protein	*Musa balbisiana* at 12 hpi
10	Ma09_g08260	ITC1587_Bchr9_P25771	MyB-related protein 308-like, encodes a R2R3 MYB protein, myb domain protein 4 (MYB4)	*Musa balbisiana* at 12 hpi
11	Ma04_g05410	ITC1587_Bchr4_P08866	MADS-box transcription factor family protein	*Musa balbisiana* at 12 hpi
12	Ma02_g12690	ITC1587_Bchr2_P04089*	Wall-associated kinase 2 (WAK2)	*Musa balbisiana* at 12 hpi
13	Ma02_g12710	ITC1587_Bchr2_P04072*	Wall associated kinase 5 (WAK5)	*Musa balbisiana* at 12 hpi
14	Ma04_g18790	ITC1587_Bchr4_P10336	Leucine-rich repeat receptor-like protein kinase family	*Musa balbisiana* at 12 hpi
15	Ma04_g19490	ITC1587_Bchr4_P10415	Carotenoid cleavage dioxygenase 4	*Musa balbisiana* at 12 hpi
16	Ma06_g14430	ITC1587_BchrUn_random_P38187	1-aminocyclopropane-1-carboxylate oxidase	*Musa balbisiana* at 12 hpi
17	Ma02_g08740	ITC1587_Bchr2_P03721	Phosphatidylinositol 4-phosphate 5-kinase 6-like	*Musa balbisiana* at 12 hpi
18	Ma01_g11950	ITC1587_Bchr1_P01647	Auxin-induced protein 15A-like	*Musa balbisiana* at 12 hpi
19	Ma04_g33020	ITC1587_Bchr4_P11074	Cinnamyl alcohol dehydrogenase 1 (CAD1) involved in lignin biosynthetic process	*Musa balbisiana* at 12 hpi
20	Ma07_g03320	ITC1587_Bchr7_P18846*	E3 ubiquitin-protein ligase PUB22	*Musa balbisiana* at 12 hpi
21	Ma07_g08690	ITC1587_Bchr7_P19375	Putative Probable WRKY transcription factor 72	*Musa balbisiana* at 12 hpi
22	Ma08_g00630	ITC1587_Bchr8_P21529	Bidirectional sugar transporter SWEET14-like	*Musa balbisiana* at 12 hpi
23	Ma11_g13300	ITC1587_Bchr11_P33322	Early nodulin-like protein 3	*Musa balbisiana* at 12 hpi
24	Ma04_g02930	ITC1587_Bchr4_P08659	Glucose-1-phosphate adenylyltransferase large subunit 1-like	*Musa balbisiana* at 12 hpi
25	Ma08_g16700	ITC1587_Bchr6_P15616*	Protein MOTHER of FT and TF 1-like	*Musa balbisiana* at 12 hpi
26	Ma08_g29360	ITC1587_Bchr8_P24561	Uncharacterized LOC103995997	*Musa balbisiana* at 12 hpi
27	Ma10_g12750	ITC1587_Bchr5_P14061*	Uncharacterized LOC104000771	*Musa balbisiana* at 12 hpi
28	Ma01_g17260	ITC1587_Bchr1_P02266	Transcription factor MYB86, myb domain protein 61	*Musa balbisiana* at 48 hpi
29	Ma03_g11520	ITC1587_Bchr3_P06219	Linoleate 13S-lipoxygenase 2-1	*Musa balbisiana* at 48 hpi
30	Ma02_g09860	ITC1587_Bchr2_P03815	Uncharacterized protein C24B11.05-like	*Musa balbisiana* at 48 hpi

Note: Gene IDs from *Musa balbisiana* are based on the results of the reciprocal best BLAST search. The best BLAST hit is reported for the genes where the reciprocal best BLAST search hit was not available and it is denoted with*.

**Figure 5:**
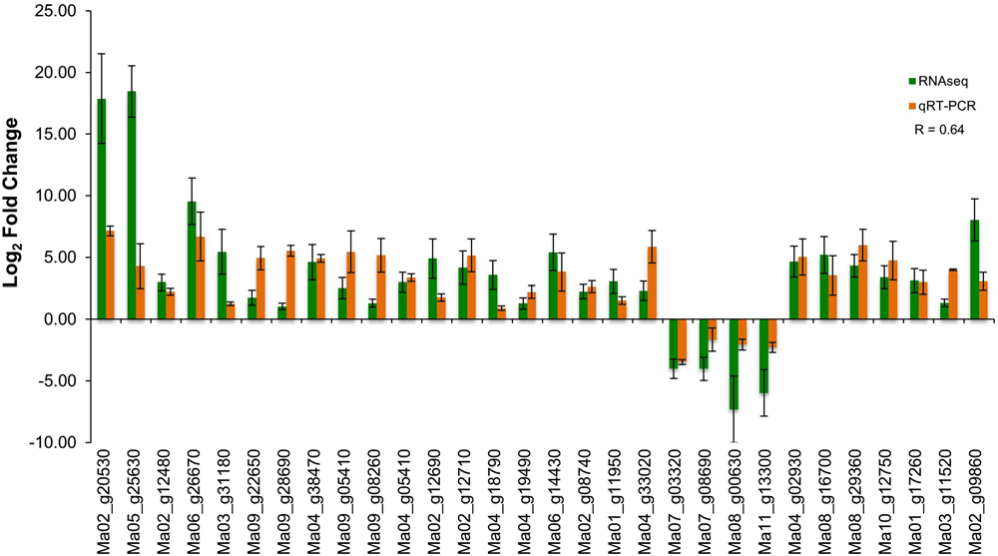
Comparison of RNA-Seq and qRT-PCR analysis for differential expression (log_2_ fold change) of selected genes. Details of selected genes are provided in Table 3.

The comparison of RNA-Seq and qRT-PCR showed similar trends of expression for candidate transcripts but difference in magnitude (Fig. 5). Pearson correlation indicated that the RNA-Seq and qRT-PCR were strongly correlated (r = 0.64, P = 0.00012). The differences in the fold change observed between the RNA-seq and qRT-PCR results might be due to analysis of independent samples and using different algorithms.

## Discussion

BXW is the most important production constraint of banana affecting livelihood of millions of people in east Africa. The wild type banana progenitor *Musa balbisiana* has resistance to BXW, whereas all cultivated banana varieties including commercial Cavendish varieties are susceptible. Currently, molecular basis of resistance and susceptibility of banana to Xcm is unknown. Therefore, transcriptome profiles of BXW-resistant and BXW-susceptible banana genotypes challenged with Xcm were compared to better understand molecular mechanism of resistance or susceptibility of banana upon bacterial infection. In presence of Xcm, resistant genotype showed higher number of DEGs at 12 hpi in comparison to 48 hpi, suggesting that defense pathways were activated by pathogen infection as early as 12 hpi.

Plants defend themselves against pathogens by activating defense mechanisms, including hypersensitive response (HR), induction of genes encoding PR, antimicrobial peptides, generation of reactive oxygen species (ROS), and enforcement of the cell wall.

Up-regulation of vicilin-like AMP in *Musa balbisiana* after artificial inoculation with Xcm suggested its involvement in plant defense and suppression of bacterial population like action of other AMPs. Vicilin-like AMPs are plant derived α-amylase inhibitors having antibacterial and antifungal activity^5^.

Once plants recognize pathogen-associated molecular patterns (PAMPs) through pattern-recognition receptors (PRRs), series of reactions are activated leading to PAMP-triggered immunity (PTI) as first line of plant immunity or basal defense. E3 ubiquitin ligase plays important role in PTI^6^. Suppression of E3 ubiquitin ligases PUB22 and PUB23 in BXW-resistant genotype *Musa balbisiana* in response to Xcm was in agreement with previous report demonstrating role of E3 ubiquitin ligases (PUB22, PUB23, and PUB24) as negative regulators of PTI in Arabidopsis^6^.

Receptor-like kinases act as PRRs, which recognize pathogens as the first layer of inducible defense. Our results showed activation of several receptor like kinases and receptor kinases in BXW-resistant genotype, indicating their role in plant defense (Table 2). WAKs are known to be involved in plant development and defense. AtWAK1 and AtWAK2 are reported to be involved in cell wall expansion^7^. OsWAK14, which is similar to WAK2, was reported to be positive regulator of blast resistance in rice^8^. LRR-RKs recognizes the pathogen effectors and induces innate immunity. Receptor kinase DUF26 also might have been involved in induction of disease resistance as previously reported that cysteine-rich receptor-like kinases (CRKs) containing DUF26 motif in their extracellular domains are involved in stress resistance^9^. The previous report demonstrating that AtLRK10L1.2, Arabidopsis ortholog of wheat LRK10 is involved in ABA-mediated signaling^10^, suggested that up-regulation of LRK10 like receptor kinase in *Musa balbisiana* should have activated the ABA signaling pathway.

PTI involves activation of oxidative burst, MAPK activity and transcription factors, preventing colonization of pathogen in host cells and providing disease resistance. MAPK cascades are major plant plasma membrane-localized PRRs, signaling early basal defense responses against pathogen infection^11^. In this study, activation of MAPK in *Musa balbisiana* indicated that up-regulation of MAPK cascades in the BXW-resistant genotype might have signaled early defense responses, including generation of ROS and HR leading to program cell death.

Upon pathogen attack, plant cell changes its redox state as one of the earliest immune responses and induces HR and programmed cell death^12^. In this study, differential expression of several genes associated with redox state was observed in BXW-resistant genotype *Musa balbisiana* at 12 hpi as early response to Xcm infection, whereas no change in redox state was detected in BXW-susceptible genotype Pisang Awak at 12 hpi (Table 2). In response to pathogen, plants produce ROS including superoxide, hydroxyl radicals and hydrogen peroxide, which trigger HR and also induces lignification and signal transduction pathways and leading to programmed cell death^13^. Several heat shock proteins were also activated in *Musa balbisiana* in response to ROS particularly H_2_O_2_ accumulation.

Our results demonstrated activation of polyamine oxidases and peroxidases involved in ROS^14,15^ in BXW-resistant genotype *Musa balbisiana* as early response to Xcm attack indicating induction of programmed cell death. Similar increase in polyamine enzymatic activity is reported in barley (*Hordeum vulgare*) during HR in response to powdery mildew^16^.

During plant-pathogen interaction, the response starts from the cell wall followed by activation of signaling pathway leading to disease resistance. In this study, lignin biosynthesis was activated in *Musa balbisiana* at 12 hpi, indicating cell wall reinforcement (Fig. 4). The cytochrome P450 involved in the lignin biosynthetic pathway was also up-regulated in the BXW-resistant genotype leading activation of lignin accumulation. Lignin plays important role in plant defense as demonstrated that deposition of lignin provided resistance to *Verticillium dahliae* in *Camelina sativa* and to *Sclerotinia sclerotiorum* in cotton^17,18^. Lignin or lignin-like phenolic polymers are rapidly deposited upon pathogen attack creating physical barrier to pathogen invasion and makes the cell wall more resistant to cell wall degrading enzymes^19^. We also observed up-regulation of phenylpropanoid pathway responsible for lignin biosynthesis in *Musa balbisiana* (Fig. 4).

Our result was similar to the previous reports, in which genes encoding cytochrome P450 were highly induced in disease resistant cauliflower, pepper and grapevines in response to bacterial pathogen infection^20–22^. The cytochrome P450 superfamily is involved in several biochemical pathways leading to the production of primary and secondary metabolites. Our result confirmed induction of cytochrome P450, which might have led to significantly high production of secondary metabolites in *Musa balbisiana* in response to pathogen infection.

We also observed significant up-regulation of peroxidases as early defense response against Xcm in *Musa balbisiana* (Fig. 2). Plant peroxidases play important role in defense mechanism by reinforcing the cell wall in response to pathogen attack, lignin and suberin formation, catalyzing cross-links between phenolic compounds in the secondary walls and polysaccharides, synthesis of phytoalexins, participating in the metabolism of ROS, activating HR at the infection site and restricting the spread of pathogen^23^.

Our results demonstrated up-regulation of several transcription factors in *Musa balbisiana* (Fig. 2, Table 2). Transcription factors respond to biotic stress by altering the expression of a cascade of defense genes. C2H2 zinc-finger transcription factors are known to function as a pathogen-induced early-defense gene in *Capsicum annuum*^24^.

In response to pathogen infection, MYB106 and MYB88 were up-regulated in BXW-susceptible genotype Pisang Awak. MYB106 is reported to be a negative regulator of trichome branching and positive regulator photosynthesis and growth^25,26^. MYB88 is involved in stomatal development as well as regulation in abiotic stress responses and female reproductive development^27^. However, role of MYB88 and MYB106 in plant defense is not known. Up-regulation of MYB4, MYB61 and MYB96 was observed in *Musa balbisiana* after infection with Xcm (Fig. 2). MYB4 is associated with protection against UV and MYB61 regulates several aspects of plant growth and development^28,29^. MYB96 was reported to be involved in regulation of lateral root growth in response to drought stress by activating the ABA-auxin signaling network^30^.

In this study, ERF transcription factor was also activated in the BXW-resistant genotype in response to pathogen infection (Fig. 2). The APETALA2/ethylene responsive factor (AP2/ERF) family is one of the largest transcription factor families involved in defense responses against various pathogens^31^. AP2-mediated disease resistance was demonstrated in Arabidopsis and tomato against *Botrytis cinerea* and *Ralstonia solanacearum*, respectively^32–34^. AP2 mediated defense responses are linked to hormones such as jasmonic acid (JA) and ethylene (ET)^31^, which was also noticed in *Musa balbisiana* at 12 hpi in response to pathogen attack (Fig. 2).

WRKY transcription factor family also plays an important role in regulating genes associated with plant defense responses. WRKY transcription factors are involved in phosphorylation sites for MAP kinases, which is part of PTI^35^. Our results showed up-regulation of WRKY4 and WRKY75 in BXW-resistant genotype in response to Xcm attack. It has been reported that overexpression of WRKY4 have provided resistance to fungal pathogens like *Botrytis cinerea*, but enhanced susceptibility to bacterial pathogens like *Pseudomonas syringae*^36^. WRKY75 transcription factor act as positive regulators of defense during interactions with bacterial pathogens and demonstrated to activate basal and R-mediated resistance in Arabidopsis and strawberry^37^.

In this study, the majority of WRKY (6, 11, 17, 22, 40, 41, 49, 65 and 72) transcription factors were suppressed in the BXW-resistant genotype indicating their involvement as negative regulator (Table 2). Several of WRKY transcription factors (AtWRKY7, 11, 17, 18, 23, 25, 27, 38, 40, 41, 48, 53, 58, 60, and 62) were also reported as negative regulators of defense signaling^35^.

ABA plays important role in plants responding for abiotic as well as biotic stress. Although ABA is known as a negative regulator of defense against biotic stresses, a number of reports have also shown its role in induction of disease resistance^38^. Our results demonstrated activation of ABA metabolism in BXW-resistant genotype in *Musa balbisiana* in response to pathogen attack (Fig. 2). ABA has been reported to play important role in plant defense by callose deposition in response to pathogen attack^39^. ABA suppresses β-1,3-glucanase, an enzyme that degrades callose, ensuring callose accumulation. Our results also showed suppression of the majority of genes associated with β-1,3-glucanases in *Musa balbisiana* after inoculation with Xcm at 12 hpi, suggesting cell wall enforcement by callose deposition.

The phytohormones SA, JA and ET play key roles in defense responses against pathogens^40^. Generally, SA-dependent plant defense system is activated by biotrophic bacterial pathogens, whereas necrotrophic fungal pathogens and chewing insects trigger JA-dependent plant defenses. However, we observed suppression of SA and activation of JA pathway in *Musa balbisiana* in response to interaction with Xcm. Similar to our results, there are reports demonstrating antagonistic interactions between ABA and SA metabolism and ABA production contributing to JA accumulation and activation for resistance against pathogen infection^38,39^. In this study NPR1-like gene was also suppressed confirming no involvement of SA pathway for systemic acquired resistance (SAR) pathway in *Musa balbisiana* at 12 hpi.

A second line of defense is Effector-Triggered Immunity (ETI), which involves R-gene families. The R-genes interactwith virulence factors of pathogens and trigger defense response characterized by rapid calcium fluxes, oxidative burst, transcriptional reprogramming within and around the infection sites and localized programmed cell death, which leads  to suppression of pathogen growth^41^. Resistance proteins protect the plant against pathogens after recognition of virulence factor, however, in the absence of specific recognition of virulence factors, basal defense response occurs through PAMPs. Similar to our studies, it has been reported overlapping of the PAMP-triggered defense with R-protein-mediated defense^42^.

In response to Xcm infection, R-gene in LRR family protein and putative disease resistance protein RPM1 in NBS-LRR domain were up-regulated in *Musa balbisiana* at 12 hpi inducing immune response providing resistance against pathogen (Table 2). LRR family proteins provide the important structural framework required for molecular interactions, and pathogen recognition^42^. Similar to our results, *Arabidopsis thaliana* RPM1 have shown to trigger the defense system against *Pseudomonas syringae*^43^.

Activation of R-gene lipase-like PAD4 gene was also observed in *Musa balbisiana* (Table 2). Lipase like gene is involved in SA signaling and function in R-gene-mediated and basal plant disease resistance^44^. However, activation of SA signaling was not observed in this study.

A typical class of susceptibility (S) genes, MLO-like protein 13 was found to be suppressed in *Musa balbisiana* at 12 hpi. MLO is postulated to act as a negative regulator of plant defenses and resistance to powdery mildew was demonstrated by knocking out susceptibility S-genes at MLO loci^45^.

Induction of PR genes in BXW-resistant genotype *Musa balbisiana* in response to Xcm inoculation indicates their role in innate immune responses like HR and systemic acquired resistance (SAR) in plants against pathogen infection. Osmotin-like protein, a PR5, is reported to be involved in plant defense responses to several pathogens and abiotic stresses^46^. Transgenic sesame overexpressing osmotin-like PR gene demonstrated resistance against *Macrophomena phaseolina* infection by activating JA/ET and SA pathways^46^.

Germin-like protein (GER1 or GLP1) was activated in both BXW-susceptible and BXW-resistant genotypes at 12 hpi and 48 hpi, respectively. Germin-like protein are reported to be increased in several plants after pathogen infection and involved in plant defense^47^. It has been reported that differences between susceptibility and resistance are associated with differences in the timing and magnitude of the induced response rather than just with the expression of various genes^48^. Further investigation is required to understand the function of GER1 in banana.

This study also showed activation of proteolysis in *Musa balbisiana* in response to pathogen at both 12 hpi and 48 hpi, whereas proteolysis was also induced at 48 hpi in Pisang Awak (Fig. 2). Generally, activation of proteolysis enables host plant cells to trigger defense response upon recognition of an invading pathogen^49^.

Our results also showed differential expression of transmembrane transporters in *Musa balbisiana*. Upon attack, pathogens use transporters to send signals to modify host cellular mechanisms promoting virulence and facilitating their own proliferation within the host tissues^50^. This study showed suppression of sugar transporter SWEET14-like protein in *Musa balbisiana* as early response to bacterial attack. However, no differential expression of this transcript was noticed in BXW-susceptible genotype Pisang Awak, indicating that SWEET14 facilitate bacterial colonization in susceptible interaction. Bacterial pathogens manipulate SWEET14 transporter for virulence by direct binding its effector to the SWEET promoter and inducing its expression leading to susceptibility to pathogen^51,52^.

Upon pathogen attack, early nodulin-like proteins were repressed in *Musa balbisiana* at 12 hpi. Normally, nodulin-like proteins are present in legumes and play an important role in symbiosis with *Rhizobium* bacteria^53^. Nodulin-like proteins are involved in transport of nutrients, amino acids, hormones and solutes required for plant development. As nodulin-like proteins assist pathogens to colonize on the host plants^53^, suppression of these proteins in BXW-resistant *Musa balbisiana* suggested restricted colonization of Xcm.

In conclusion, comparative transcriptome of the BXW-resistant and the BXW-susceptible genotypes of banana provided some understanding of molecular basis of response against Xcm. The DEGs mapped to biotic stress pathways allowed us to identify number of candidate genes involved in banana-Xcm interaction. Our results demonstrated activation of both PAMP-triggered defense and R protein-mediated defense in the BXW-resistant genotype *Musa balbisiana* as response to Xcm infection. Upon Xcm attack, pathogen-recognition receptors trigger cascade of responses leading to PTI as first line of defense. Further pathogen effectors were recognized by disease resistant genes inducing R-gene-mediated resistance. In this study transcripts associated with HR and programmed cell death were found to be activated in BXW-resistant genotype as early response to Xcm attack. RNAseq and qRT-PCR results also indicated activation of antimicrobial peptide in the BXW-resistant genotype. The antimicrobial activity of vicilin-like peptide from *Musa balbisiana* needs to be further tested against Xcm.

The differential expressions of several genes involved in plant defense were validated by qRT-PCR (Fig. 5 and Table 3). Further functional genomics need to be performed to understand in-depth molecular mechanism of defense against Xcm. The significant genes should be knocked out or over-expressed to better understand their role in plant defense against Xcm.

To the best of our knowledge, this study is the first transcriptome analysis of the banana genotypes for the response to Xcm pathogen. Our data provide insights on the defense mechanism in the BXW-resistant wild type banana *Musa balbisiana* to the most damaging pathogen Xcm. This information can be used in crop improvement program to transfer the disease resistance trait from wild type banana to farmer-preferred banana cultivars commonly grown in Africa.

## Materials and Methods

### Plant material, inoculation and sampling

The BXW disease resistant genotype *Musa balbisiana* (BB) and highly susceptible banana cultivar Pisang Awak (ABB, commonly known as Kayinja) were obtained from *in vitro* collection at IITA-Kenya. The *in vitro* plantlets were micropropagated in tissue culture.

Pure culture of Ugandan isolate of Xcm isolate collected from Pathology Laboratory, IITA was maintained on YTSA medium (1% yeast extract, 1% tryptone, 1% sucrose and 1.5% agar) at 4 °C. A single bacterial colony was inoculated into 25 ml of YTS medium (1% yeast extract, 1% tryptone and 1% sucrose) and cultured at 28 °C with shaking at 150 rpm for 48 h. The bacterial culture was centrifuged at 5000 rpm for 5 min and pellet was re-suspended in sterile double distilled water. The optical density (OD 600 nm) of the bacterial suspension was checked and bacterial concentration was adjusted to 10^8^ cfu/ml with sterile water. Fresh inoculum was used for all the experiments in order to have high virulent potential of the pathogen.

One month-old *in vitro* rooted plantlets with 3–4 leaves were artificially inoculated with fresh culture of Xcm. About 100 μl of bacterial suspension (10^8^ cfu ml^−1^) was artificially injected using insulin syringe into the midrib of the second fully open leaf. This method of artificial inoculation is similar to natural infection through injury by contaminated cutting tools, commonly practiced by farmers. Previous study in our laboratory showed development of BXW symptoms and complete wilting of Pisang Awak plantlets artificially inoculated with Xcm^54^. The inoculated leaf of *M*. *balbisiana* also showed necrotic and chlorotic patches due to hypersensitive response, but these symptoms did not progress and the plants were subsequently healthy^54^.

Additional plantlets were mock inoculated with sterile water as control in order to nullify the effect of wounding through injection.

The inoculated leaf samples were collected at 0, 12 hpi, 48 hpi. These time points were selected as the expression of defense genes during early infection with Xcm was observed to be high at 12 hpi and then decreasing at 48 hpi^55^. Three biological and three technical replicates were used for each time point.

The samples were:

1. Pisang Awak inoculated with bacterial culture at 0, 12 hpi, 48 hpi
2. *Musa balbisiana* inoculated with bacteria at 0, 12 hpi, 48 hpi
3. Pisang Awak mock inoculated with water at 12 hpi, 48 hpi
4. *Musa balbisiana* mock inoculated with water at 12 hpi, 48 hpi

### RNA extraction and library preparation

Total RNA was extracted from 100 mg leaf samples using the RNeasy plant mini kit (Qiagen, GmbH, Hilden, Germany) and treated with DNase (RNeasy Plant Mini kit, Qiagen). The quality of RNA was assessed using denaturing agarose gel stained with gelred (Biotium, USA) and quantified using a Nanodrop 2000 (Thermo Fisher Scientific, MA, USA).

A total of 30 cDNA libraries were prepared in each of the two treatments [three biological replicates at each of the three time points (0, 12 hpi and 48 hpi) per genotype inoculated with bacterial culture and two time points (12 hpi, 48 hpi) inoculated with sterile water] using Illumina TruSeq RNA Sample Preparation Kit v2. Poly (A) mRNA was obtained from 1 µg of the total RNA using poly-T oligos attached to magnetic beads. The purified mRNA was fragmented and used to synthesize first strand cDNA using SuperScript II Reverse Transcriptase (Thermo Fisher Scientific) and random primers. Second strand cDNA was synthesized from the resultant first strand and this was end-repaired by adding ‘A’ (A-tailing) at the 3′ ends to allow the ligation of indexed adaptors. The adaptor indexes were used in such a way that it allowed for multiplexing in sequencing. The libraries were enriched through twelve cycles of PCR amplifications. Quantitative and qualitative assessments of the libraries were done using QuBit DNA assay (Thermo Fisher Scientific) as well as with Agilent DNA 1000 assay (Agilent technologies, CA, USA) on an Agilent 2100 bioanalyzer. All the assays on quality and quantity were performed according to the respective manufacturer’s protocols.

### RNA sequencing (RNA-Seq) and differential gene expression analysis

The cDNA library was sequenced pair-ends wise using the Illumina HiSeq™ 2500, and 100-bp reads were generated. The sequencing was performed by Center for Genomics and Systems Biology, New York University.

The reads were trimmed for quality using Trimmomatic and aligned against the reference *Musa acuminata* genome using Hisat2^56^. The reference genome for *Musa acuminata* DH Pahang annotation version 2 and annotations were downloaded from Banana Genome Hub^57^ (http://banana-genome-hub.southgreen.fr/download). This genome sequence is considered to be the reference genome for *Musa* species due to its completeness and quality of annotation^58^. The quality of annotation has recently been quantified through a BUSCO analysis, which scores the genome of *Musa acuminata* DH Pahang at 96.5% and the genome of *Musa balbisiana* Pisang Klutuk Wulung at 66.5%^59^. This genome sequence has been extensively used in other transcriptome studies on *Musa* including genotypes having B-genome^60^.

The protein sequences from the *Musa acuminata* DH Pahang and *Musa balbisiana* Pisang Klutuk Wulung genomes were downloaded and the reciprocal best BLAST hits were obtained using a perl script. The gene identifiers (IDs) from *Musa balbisiana* Pisang Klutuk Wulung are reported in all tables based on the results of the reciprocal best BLAST search. The best BLAST hit is reported for the genes where the reciprocal best BLAST search hit was not available and it is denoted with the symbol “*” after the gene identifier.

HTSeq-count was used to count the number of reads that map to the different annotated genes. Readcounts for annotated genes were loaded in R and genes with low expression were filtered out. The genes were removed from the dataset if none of its groups had a median of 10 reads mapping to the genome. The filtered readcount data was used as input for DESeq2 to determine differentially expressed genes. The genes with fold change of greater than 1.5 or <−1.5 and also an adjusted p-value less FDR corrected p-value of 0.1 were identified.

The change in gene expression due to bacterial inoculation was calculated for both genotypes at each time point by comparing them with mock-inoculated plants of the same genotype using the contrasts function in DESeq2. Also the interaction between genotypes and pathogen i.e. genotypes:treatment interaction was recorded.

### Go-term enrichment analysis

A Gene Ontology (GO) Enrichment Analysis of differentially expressed genes was performed using GO-terms provided by the Banana Genome Hub and the GOStats package in R^61^. GOStats has a functionality that allows individuals to create their own GO annotation database for species that are currently not supported in the annotation databases. Using the Banana Genome Hub Gene-to-GO association a Gene Set Enrichment dataset was created and used for identifying GO-terms that were over-represented in a given gene list. The function has been enhanced to provide the FDR corrected p-values. All go-terms were adjusted for p-value of less than 0.05.

### Biotic stress and metabolic pathway analysis

The DEGs were mapped to different pathways using the Mapman^62^ version 3.5.1R2. The mapping to the Mapman functional categories (BINs) was done for all the protein sequences from the *Musa acuminata* version 2 genome. The protein sequences were uploaded to the Mercator tool^63^. The pathway analysis used the overall metabolic pathway and the biotic stress pathways for this study.

### Quantitative RT-PCR validation

To verify the results of RNA-Seq analysis, qRT-PCR analysis was performed with 30 selected DEGs using gene specific primers (Supplementary Table S8). The primers were designed using the PrimerQuest Tool (Integrated DNA Technologies, Iowa, USA) using the 2 primers, intercalating dyes qPCR design type.

The samples for qRT-PCR were independently prepared as described above for RNAseq. One month-old *in vitro* rooted plantlets were artificially inoculated with 100 μl of bacterial culture (10^8^ cfu ml^−1^) of Xcm. The plantlets mock inoculated with sterile water were used as control. The inoculated leaf samples were collected at 0, 12 hpi, 48 hpi and RNA were isolated using the RNeasy plant mini kit (Qiagen, GmbH, Hilden, Germany) and treated with DNase (RNeasy Plant Mini kit, Qiagen). First strand cDNA was synthesized using reverse transcriptase of the Maxima H Minus First Strand cDNA synthesis kit with oligo DT primers (Thermo scientific). The primer specificity for each gene was confirmed by PCR and melting curve in qRT-PCR.

The qRT-PCR was performed using Maxima SYBR green/ROX PCR kit (Thermo Scientific) on 7900 Real Time PCR System (Applied Biosystems, USA). The experiment was set up using three biological and three technical replicates. Relative expression data were normalized using the *Musa25S* ribosomal gene as reference. Mock inoculated control plant was used as calibrator to calculate log_2_ fold change of target gene using the ΔΔCt method^64^.

$${\rm{\Delta }}\mathrm{CT}\,({\rm{Target}})={\rm{CT}}\,(\mathrm{Target})\,-\,{\rm{CT}}\,({\rm{Reference}})$$

$${\rm{\Delta }}{\rm{\Delta }}\mathrm{Ct}={\rm{\Delta }}\mathrm{CT}\,({\rm{Xcm}}\,{\rm{inoculated}}\,{\rm{sample}})\,-\,{\rm{\Delta }}\mathrm{CT}\,({\rm{Mock}}\,{\rm{inoculated}}\,{\rm{sample}})$$

The means and standard error were calculated for three replicates per experiment using Minitab 16 statistical software, 2012.

The log2 fold change by RNA-Seq and qRT-PCR analysis were compared using Pearson correlation analysis in Minitab 16 statistical software.

## Supplementary information

Supplementary Tables
