# Supplementary material for: Molecular Basis of Disease Resistance in Banana Progenitor Musa balbisiana against Xanthomonas campestris pv. musacearum
Source: Sci Rep. 2019 May 7;9:7007. doi: 10.1038/s41598-019-43421-1 (PMC6504851; doi:10.1038/s41598-019-43421-1)
Supplement: Supplementary file 1 — Supplementary Tables [file 41598_2019_43421_MOESM1_ESM.pdf]

Molecular Basis of Disease Resistance in Banana Progenitor *Musa balbisiana* against *Xanthomonas campestris* pv. *musacearum*

Leena Tripathi[1*], Jaindra Nath Tripathi[1], Trushar Shah[1], Kariuki Samwel Muiruri[1], Manpreet Katari[2]

[1]International Institute of Tropical Agriculture (IITA), P.O. Box 30709-00100, Nairobi, Kenya

[2] Department of Biology, New York University, New York, NY, United States

[*]Correspondence author - L.Tripathi@cgiar.org

Supplementary Tables

Table S1: Complete list of genes differentially expressed in BXW-susceptible genotype Pisang Awak in response to *Xanthomonas campestris* pv. *musacearum* at 12 hpi.

Gene ID from *Musa acuminata* (DH Pahang)	Gene ID from *Musa balbisiana* (Pisang Klutuk Wulung)	BaseMean	Log2 Fold Change	lfcSE	Stat	Pvalue	Padj	Description
Ma11_g18240	ITC1587_Bchr11_P33802	82.41	19.11	2.49	7.67	1.79E-14	2.38E-10	non-specific lipid-transfer protein-like
Ma05_g25630	ITC1587_Bchr5_P14199	10.07	18.46	2.08	8.87	7.52E-19	2.01E-14	MYB family transcription factor
Ma02_g20530	ITC1587_Bchr2_P04749	23.31	17.87	3.65	4.90	9.75E-07	8.67E-03	Germin-like protein 8-14
Ma02_g12480	ITC1587_Bchr1_P00003*	15.14	2.98	0.65	4.61	4.10E-06	2.73E-02	alpha-galactosidase-like

Note: Gene IDs from *Musa balbisiana* are based on the results of the reciprocal best BLAST search. The best BLAST hit is reported for the genes where the reciprocal best BLAST search hit was not available and it is denoted with *.

Table S2: Complete list of genes differentially expressed in BXW-resistant wild type banana *Musa balbisiana* in response to *Xanthomonas campestris* pv. *musacearum* at 12 hpi.

Gene ID from *Musa acuminata* (DH Pahang)	Gene ID from *Musa balbisiana* (Pisang Klutuk Wulung)	BaseMean	Log2 Fold Change	lfcSE	Stat	Pvalue	Padj	Description
Ma06_g26670	ITC1587_Bchr6_P17425	65.45	9.54	1.88	5.08	3.68E-07	2.65E-04	polyamine oxidase-like
Ma06_g06860	ITC1587_BchrUn_random_P38862	3.39	5.46	1.5	3.65	2.61E-04	1.45E-02	geranylgeranyl pyrophosphate synthase 7
Ma03_g31180	ITC1587_Bchr3_P08153	3.15	5.44	1.81	3.01	2.62E-03	5.44E-02	vicilin-like antimicrobial peptides 2-2
Ma06_g14430	ITC1587_BchrUn_random_P38187	145.69	5.4	1.49	3.62	2.94E-04	1.55E-02	1-aminocyclopropane-1-carboxylate oxidase-like
Ma08_g16700	ITC1587_Bchr6_P15616*	18.94	5.19	1.5	3.47	5.19E-04	2.17E-02	protein MOTHER of FT and TF 1-like
Ma07_g07170	ITC1587_Bchr7_P19196	291.67	5.04	1.25	4.05	5.15E-05	5.71E-03	8-hydroxygeraniol dehydrogenase-like
Ma06_g32230	ITC1587_Bchr6_P17952	9.83	4.96	1.37	3.63	2.88E-04	1.53E-02	no apical meristem protein
Ma02_g12690	ITC1587_Bchr2_P04089*	12.8	4.9	1.59	3.07	2.12E-03	4.82E-02	wall-associated receptor kinase-like 6
Ma05_g27330	ITC1587_Bchr5_P14359	33.14	4.85	1.64	2.96	3.11E-03	6.01E-02	cytochrome P450 86B1-like

Ma04_g02930	ITC1587_Bchr4_P08659	72.77	4.66	1.25	3.73	1.95E-04	1.23E-02	glucose-1-phosphate adenylyltransferase large subunit 1-like
Ma04_g38470	ITC1587_Bchr4_P11526	324.89	4.6	1.44	3.19	1.44E-03	3.91E-02	protein P21-like
Ma08_g29360	ITC1587_Bchr8_P24561	28.58	4.31	0.91	4.75	2.06E-06	7.95E-04	uncharacterized LOC103995997
Ma02_g12710	ITC1587_Bchr2_P04072*	39.26	4.17	1.35	3.09	2.00E-03	4.68E-02	wall-associated receptor kinase 3-like
Ma10_g12720	ITC1587_Bchr5_P14061*	85.51	3.74	0.86	4.37	1.22E-05	2.44E-03	Hypothetical protein
Ma04_g35880	ITC1587_Bchr4_P11318	7.54	3.69	1.06	3.5	4.74E-04	2.05E-02	actin-depolymerizing factor 1-like
Ma01_g21700	ITC1587_Bchr1_P02746	19.94	3.67	1.14	3.23	1.25E-03	3.61E-02	uncharacterized LOC104000306
Ma02_g24180	ITC1587_Bchr2_P05078	4.69	3.67	1.23	2.98	2.86E-03	5.76E-02	cyclin-D4-1-like
Ma04_g18790	ITC1587_Bchr4_P10336	29.03	3.58	1.16	3.08	2.07E-03	4.74E-02	probable LRR receptor-like serine/threonine-protein kinase At4g08850
Ma08_g04900	ITC1587_Bchr8_P21882*	30.26	3.52	0.65	5.38	7.26E-08	9.52E-05	Fragment
Ma03_g11500	ITC1587_Bchr3_P06218*	19.23	3.47	1.14	3.04	2.35E-03	5.10E-02	3-oxoacyl-[acyl-carrier-protein] reductase
Ma10_g12750	ITC1587_Bchr5_P14061*	146.81	3.39	0.9	3.78	1.60E-04	1.10E-02	uncharacterized LOC104000771
Ma04_g39350	ITC1587_Bchr4_P11595	13.18	3.36	0.9	3.75	1.75E-04	1.17E-02	ninja-family protein AFP3-like
Ma09_g29150	ITC1587_Bchr9_P28137	13.2	3.36	1.15	2.93	3.35E-03	6.25E-02	putative 26.5 kDa heat shock protein
Ma07_g17620	ITC1587_Bchr8_P24644*	14.69	3.29	0.92	3.57	3.57E-04	1.77E-02	uncharacterized LOC103992059
Ma01_g07400	ITC1587_Bchr6_P17629*	70.94	3.21	1.08	2.96	3.03E-03	5.94E-02	monothiol glutaredoxin-S9-like
Ma03_g02250	ITC1587_Bchr3_P05368*	17.82	3.13	1.13	2.78	5.52E-03	8.17E-02	uncharacterized LOC103977124
Ma08_g10610	ITC1587_Bchr8_P22402	54.6	3.07	0.77	4	6.42E-05	6.59E-03	galactinol synthase 1
Ma01_g11950	ITC1587_Bchr1_P01647	6.93	3.06	0.96	3.2	1.37E-03	3.80E-02	auxin-induced protein 15A-like
Ma04_g05410	ITC1587_Bchr4_P08866	14.97	3	0.8	3.74	1.82E-04	1.19E-02	agamous-like MADS-box protein AGL61
Ma11_g16600	ITC1587_Bchr9_P28345*	11.03	2.92	0.86	3.39	7.02E-04	2.57E-02	dual specificity protein phosphatase PHS1-like
Ma08_g08200	ITC1587_Bchr8_P22177	76.11	2.88	0.72	4	6.40E-05	6.59E-03	putative expressed protein
Ma10_g29680	ITC1587_Bchr10_P31511*	76.45	2.85	0.77	3.7	2.17E-04	1.29E-02	ATP-dependent zinc metalloprotease FTSH 6
Ma03_g04050	ITC1587_Bchr3_P05519	88.16	2.79	0.72	3.9	9.55E-05	8.11E-03	BTB/POZ domain-containing protein At1g55760-like
Ma08_g17800	ITC1587_Bchr8_P23541	33.5	2.76	0.78	3.55	3.84E-04	1.85E-02	protein PLASTID MOVEMENT IMPAIRED 2-like
Ma05_g11490	ITC1587_Bchr5_P12707	8.6	2.72	0.8	3.39	6.89E-04	2.54E-02	RING-H2 finger protein ATL8-like

Ma05_g24030	ITC1587_Bchr5_P14049	96.99	2.71	0.72	3.76	1.69E-04	1.14E-02	heparan-alpha-glucosaminide N-acetyltransferase-like
Ma06_g09540	ITC1587_Bchr6_P15490*	7.98	2.71	0.86	3.13	1.72E-03	4.33E-02	protein HVA22-like
Ma08_g22470		377.42	2.69	0.88	3.05	2.31E-03	5.05E-02	Hypothetical protein
Ma06_g34040	ITC1587_Bchr6_P18122	12.23	2.53	0.87	2.9	3.72E-03	6.59E-02	protein LURP-one-related 8-like
Ma09_g05410	ITC1587_Bchr9_P25531	83.15	2.52	0.84	2.99	2.79E-03	5.68E-02	pathogen-related protein
Ma09_g02560	ITC1587_Bchr9_P25297	16.49	2.51	0.86	2.91	3.65E-03	6.56E-02	uncharacterized LOC103996779
Ma10_g21020	ITC1587_Bchr10_P30746	5.58	2.51	0.89	2.83	4.61E-03	7.48E-02	pectate lyase-like
Ma10_g11690	ITC1587_Bchr4_P09673*	7.27	2.45	0.83	2.95	3.16E-03	6.07E-02	protein SYM1-like
Ma02_g05420	ITC1587_Bchr2_P03415	7.88	2.34	0.8	2.94	3.27E-03	6.19E-02	uncharacterized LOC103973692
Ma04_g33020	ITC1587_Bchr4_P11074	542.97	2.3	0.76	3.01	2.62E-03	5.44E-02	cinnamyl alcohol dehydrogenase 1
Ma08_g28170	ITC1587_Bchr8_P24457	146.94	2.29	0.64	3.58	3.38E-04	1.71E-02	probable serine acetyltransferase 1
Ma03_g31600	ITC1587_Bchr3_P08196	38.66	2.28	0.78	2.93	3.37E-03	6.25E-02	ninja-family protein 6-like
Ma04_g35970	ITC1587_Bchr4_P11328	65.11	2.26	0.64	3.54	3.98E-04	1.89E-02	subtilisin-like protease
Ma08_g19300	ITC1587_Bchr8_P22999	89.36	2.26	0.53	4.22	2.48E-05	3.65E-03	putative expressed protein
Ma09_g09290		44.3	2.24	0.81	2.78	5.44E-03	8.12E-02	putative expressed protein
Ma01_g12890	ITC1587_Bchr1_P01723	10.4	2.23	0.64	3.5	4.72E-04	2.05E-02	putative 18 kda seed maturation protein
Ma02_g08740	ITC1587_Bchr2_P03721	16.64	2.23	0.57	3.92	8.96E-05	7.92E-03	phosphatidylinositol 4-phosphate 5-kinase 6-like
Ma09_g28620	ITC1587_Bchr9_P28084	30.1	2.2	0.76	2.89	3.81E-03	6.70E-02	RING-H2 finger protein ATL43-like
Ma10_g30810	ITC1587_Bchr10_P31614	21.47	2.18	0.57	3.86	1.13E-04	8.85E-03	zinc finger protein 4-like
Ma01_g09550	ITC1587_Bchr1_P01419*	7.83	2.16	0.71	3.05	2.30E-03	5.05E-02	scarecrow-like protein 28
Ma10_g25510	ITC1587_Bchr10_P31134	36.03	2.14	0.63	3.38	7.30E-04	2.61E-02	uncharacterized LOC103969916
Ma03_g30610	ITC1587_Bchr3_P08112*	13.63	2.12	0.78	2.71	6.67E-03	9.14E-02	probable receptor-like protein kinase At1g67000
Ma04_g26590	ITC1587_Bchr4_P10494	303.25	2.09	0.44	4.77	1.82E-06	7.60E-04	uncharacterized LOC103983225
Ma06_g27540	ITC1587_Bchr6_P17520	26.97	2.07	0.61	3.39	6.92E-04	2.54E-02	exocyst complex component EXO70B1-like
Ma11_g16580	ITC1587_Bchr11_P33656*	72.69	2.05	0.67	3.05	2.30E-03	5.05E-02	Ethylene receptor
Ma04_g07150	ITC1587_Bchr4_P09012	10.39	2.02	0.69	2.94	3.30E-03	6.20E-02	homeobox-leucine zipper protein HOX12-like
Ma01_g14540	ITC1587_Bchr1_P01860*	2071.61	2.02	0.49	4.16	3.20E-05	4.24E-03	chr1

Ma03_g32480	ITC1587_Bchr3_P08271*	50.53	1.99	0.49	4.09	4.36E-05	5.24E-03	Secologanin synthase
Ma07_g09320	ITC1587_Bchr7_P19437	59.79	1.96	0.59	3.32	9.13E-04	2.99E-02	uncharacterized LOC103991249
Ma03_g17120	ITC1587_Bchr3_P06850	13.4	1.95	0.65	3.01	2.57E-03	5.35E-02	heat stress transcription factor C-1-like
Ma05_g05930	ITC1587_Bchr5_P12213	150.85	1.92	0.52	3.67	2.38E-04	1.35E-02	UDP-glycosyltransferase 73C6-like
Ma04_g26870	ITC1587_Bchr4_P10518	86.18	1.89	0.4	4.78	1.72E-06	7.49E-04	uncharacterized LOC103983203
Ma07_g17570	ITC1587_BchrUn_random _P37718	585.36	1.87	0.44	4.24	2.27E-05	3.46E-03	CBL-interacting protein kinase 1-like
Ma04_g25840	ITC1587_Bchr1_P00570	1846.18	1.86	0.35	5.28	1.26E-07	1.46E-04	granule-bound starch synthase 1
Ma07_g22730	ITC1587_Bchr7_P20906	14.41	1.86	0.65	2.87	4.11E-03	7.01E-02	transcription factor MUTE-like
Ma02_g08780	ITC1587_Bchr2_P03725	363.75	1.85	0.4	4.59	4.41E-06	1.26E-03	alpha carbonic anhydrase 1
Ma11_g09710	ITC1587_Bchr11_P32964	859.02	1.84	0.45	4.07	4.68E-05	5.42E-03	ferric reduction oxidase 7
Ma11_g02070	ITC1587_Bchr11_P31881	97.8	1.83	0.47	3.89	9.96E-05	8.37E-03	probable protein phosphatase 2C 75
Ma01_g21430	ITC1587_Bchr7_P21273*	53.42	1.81	0.65	2.78	5.39E-03	8.08E-02	Hydroxyacylglutathione hydrolase 3
Ma10_g05060	ITC1587_Bchr6_P18098*	115.95	1.79	0.48	3.69	2.26E-04	1.31E-02	uncharacterized LOC103999622
Ma06_g29520	ITC1587_Bchr6_P17697	178.66	1.77	0.55	3.19	1.40E-03	3.87E-02	70 kDa peptidyl-prolyl isomerase-like
Ma09_g22650	ITC1587_Bchr9_P27517*	116.3	1.75	0.6	2.93	3.38E-03	6.25E-02	probable leucine-rich repeat receptor-like protein kinase At1g35710
Ma01_g00980		10.06	1.72	0.58	2.96	3.10E-03	6.01E-02	Hypothetical protein
Ma04_g08840	ITC1587_Bchr4_P09163	32.46	1.72	0.47	3.64	2.68E-04	1.48E-02	protein CHUP1
Ma05_g03580	ITC1587_Bchr5_P11986	289.18	1.68	0.46	3.69	2.22E-04	1.30E-02	ubiquinol oxidase 2
Ma11_g19280	ITC1587_Bchr11_P33899	98.57	1.68	0.51	3.3	9.79E-04	3.12E-02	1-Cys peroxiredoxin
Ma10_g27770	ITC1587_Bchr10_P31333	350.69	1.67	0.46	3.67	2.40E-04	1.35E-02	heat stress transcription factor C-2b-like
Ma02_g08840	ITC1587_Bchr2_P03732	158.14	1.66	0.47	3.54	4.00E-04	1.90E-02	annexin D3-like
Ma06_g05410	ITC1587_Bchr1_P02099*	23.15	1.64	0.53	3.11	1.90E-03	4.54E-02	villin-4-like
Ma09_g06650	ITC1587_Bchr9_P25627	53.65	1.6	0.54	2.99	2.78E-03	5.67E-02	glucose-1-phosphate adenylyltransferase large subunit 1-like
Ma04_g33310	ITC1587_Bchr4_P11096	46.08	1.6	0.41	3.93	8.43E-05	7.59E-03	phenolic glucoside malonyltransferase 1-like
Ma04_g21360	ITC1587_Bchr1_P00031	99.77	1.6	0.52	3.08	2.07E-03	4.74E-02	probable copper-transporting ATPase HMA5
Ma04_g11920	ITC1587_Bchr4_P09465	7.83	1.58	0.53	2.99	2.79E-03	5.67E-02	suppressor of phythchrome A
Ma08_g04910	ITC1587_Bchr1_P02581*	83.93	1.57	0.41	3.82	1.36E-04	1.00E-02	squalene monooxygenase-like

Ma06_g01860	ITC1587_Bchr6_P14797*	237.17	1.56	0.5	3.15	1.64E-03	4.21E-02	uncharacterized LOC103986549
Ma03_g11520	ITC1587_Bchr3_P06219	6097.91	1.53	0.31	4.89	9.90E-07	5.33E-04	linoleate 13S-lipoxygenase 2-1
Ma01_g07170	ITC1587_Bchr1_P01202*	246.67	1.53	0.38	3.99	6.74E-05	6.75E-03	anthocyanidin 5, 3-O-glucosyltransferase-like
Ma02_g24140	ITC1587_Bchr2_P05074	1052.62	1.52	0.32	4.74	2.17E-06	8.04E-04	uncharacterized LOC103976698
Ma02_g17900	ITC1587_Bchr9_P28125*	235.5	1.52	0.53	2.87	4.09E-03	7.00E-02	plant-specific domain TIGR01589 family protein
Ma01_g17110	ITC1587_Bchr1_P02281	397.28	1.51	0.36	4.18	2.93E-05	4.01E-03	uncharacterized LOC103997384
Ma05_g16190	ITC1587_BchrUn_random_P36900*	737.14	1.5	0.3	4.98	6.46E-07	4.20E-04	inositol-3-phosphate synthase-like
Ma02_g22270	ITC1587_Bchr2_P04917	172.35	1.5	0.42	3.54	3.93E-04	1.88E-02	protein FANTASTIC FOUR 1-like
Ma06_g12080	ITC1587_Bchr6_P15721*	19.51	1.5	0.5	2.99	2.77E-03	5.67E-02	uncharacterized LOC103987476
Ma11_g14590	ITC1587_Bchr11_P33465	134.99	1.5	0.45	3.32	9.00E-04	2.98E-02	calmodulin-like
Ma01_g02150	ITC1587_Bchr1_P00623*	653.18	1.49	0.36	4.12	3.82E-05	4.81E-03	probable ascorbate-specific transmembrane electron transporter 1
Ma05_g11350	ITC1587_Bchr11_P34036*	24.06	1.48	0.5	2.99	2.77E-03	5.67E-02	expansin-like A1
Ma05_g18640	ITC1587_Bchr5_P13349	12.03	1.48	0.55	2.7	6.84E-03	9.26E-02	4-alpha-glucan-branching enzyme 2
Ma06_g06990	ITC1587_Bchr6_P15254	1120.43	1.46	0.42	3.52	4.39E-04	1.98E-02	crocetin glucosyltransferase
Ma03_g11210	ITC1587_Bchr3_P06202*	71.09	1.46	0.54	2.69	7.25E-03	9.54E-02	subtilisin-like protease SBT5.3
Ma05_g15570	ITC1587_Bchr5_P13081	27.22	1.46	0.4	3.6	3.18E-04	1.64E-02	uncharacterized LOC103985037
Ma06_g07000	ITC1587_Bchr6_P15254*	475.13	1.45	0.41	3.53	4.12E-04	1.92E-02	crocetin glucosyltransferase
Ma08_g04840	ITC1587_Bchr8_P21878	188.98	1.45	0.45	3.23	1.23E-03	3.58E-02	probable zinc metallopeptidase EGY3
Ma05_g11180	ITC1587_Bchr5_P12673	31.52	1.44	0.47	3.09	2.00E-03	4.68E-02	uncharacterized LOC103984779
Ma03_g32450	ITC1587_Bchr3_P08271*	6369.22	1.43	0.36	3.91	9.05E-05	7.92E-03	cytochrome P450 CYP72A219-like
Ma10_g14770	ITC1587_Bchr10_P30216	2099.25	1.42	0.39	3.64	2.74E-04	1.49E-02	BAG family molecular chaperone regulator 6-like
Ma01_g07150	ITC1587_Bchr1_P01202	526.27	1.42	0.35	4	6.34E-05	6.55E-03	anthocyanidin 5, 3-O-glucosyltransferase-like
Ma04_g10170	ITC1587_Bchr4_P09289	236.55	1.42	0.38	3.76	1.71E-04	1.15E-02	Granule-bound starch synthase 2
Ma03_g32500	ITC1587_Bchr3_P08271	3635.33	1.42	0.38	3.78	1.59E-04	1.10E-02	cytochrome P450 CYP72A219-like
Ma03_g32460	ITC1587_Bchr3_P08271*	2780.58	1.41	0.38	3.68	2.35E-04	1.34E-02	cytochrome P450 CYP72A219-like
Ma01_g10090	ITC1587_Bchr1_P01463	87.31	1.41	0.4	3.56	3.70E-04	1.81E-02	uncharacterized LOC103983795

Ma03_g05910	ITC1587_Bchr3_P05693	1055.48	1.39	0.42	3.34	8.32E-04	2.84E-02	protein NRT1/ PTR FAMILY 6.4
Ma06_g25700	ITC1587_BchrUn_random_P39673	223.77	1.39	0.49	2.83	4.72E-03	7.54E-02	(-)-alpha-terpineol synthase-like
Ma11_g02650	ITC1587_Bchr11_P31934	21.53	1.39	0.5	2.75	5.99E-03	8.54E-02	naringenin, 2-oxoglutarate 3-dioxygenase-like
Ma07_g06690	ITC1587_Bchr7_P19144*	34.17	1.38	0.49	2.83	4.70E-03	7.53E-02	protein O-glucosyltransferase 1
Ma05_g06010	ITC1587_Bchr5_P12219	1440.28	1.38	0.4	3.48	5.05E-04	2.13E-02	dehydrodolichyl diphosphate synthase 2-like
Ma03_g09450	ITC1587_Bchr3_P06039	38.58	1.38	0.46	3	2.66E-03	5.49E-02	protein LURP-one-related 8-like
Ma02_g19870	ITC1587_Bchr2_P04696	35.1	1.37	0.41	3.38	7.27E-04	2.61E-02	E3 ubiquitin-protein ligase Os04g0590900
Ma10_g14170	ITC1587_Bchr10_P30158	141.56	1.37	0.39	3.53	4.11E-04	1.92E-02	histidine-containing phosphotransfer protein 4-like
Ma04_g12630	ITC1587_Bchr4_P09530	312.36	1.36	0.4	3.37	7.52E-04	2.67E-02	probable homogentisate phytyltransferase 1
Ma02_g16590	ITC1587_Bchr2_P04403	334.48	1.35	0.43	3.14	1.68E-03	4.28E-02	uncharacterized glycosyl hydrolase Rv2006/MT2062
Ma02_g01600	ITC1587_Bchr2_P03131	242.26	1.35	0.47	2.9	3.68E-03	6.57E-02	cytokinin riboside 5'-monophosphate phosphoribohydrolase LOG1-like
Ma08_g04270	ITC1587_Bchr8_P21825	28.91	1.35	0.49	2.75	5.99E-03	8.54E-02	truncated transcription factor CAULIFLOWER A-like
Ma05_g18810	ITC1587_Bchr5_P13157*	196.4	1.34	0.33	4.1	4.09E-05	5.00E-03	IN2-2 protein-like
Ma11_g20540	ITC1587_Bchr11_P34014*	92.3	1.34	0.44	3.02	2.50E-03	5.27E-02	Hypothetical protein
Ma04_g20750	ITC1587_Bchr7_P20320*	26.34	1.34	0.36	3.67	2.38E-04	1.35E-02	uncharacterized LOC103979843
Ma10_g15660	ITC1587_Bchr10_P30296	93.48	1.34	0.34	3.91	9.06E-05	7.92E-03	uncharacterized LOC103968354
Ma04_g19580	ITC1587_Bchr5_P12913*	237.32	1.34	0.29	4.61	4.01E-06	1.23E-03	uncharacterized LOC103982051
Ma05_g25720	ITC1587_Bchr5_P14209	214.94	1.33	0.43	3.11	1.85E-03	4.47E-02	Aquaporin NIP2-1
Ma00_g03150	ITC1587_BchrUn_random_P38082	56.62	1.32	0.38	3.48	4.96E-04	2.10E-02	uncharacterized LOC103974549
Ma09_g04220	ITC1587_Bchr9_P25437	137.5	1.32	0.33	3.98	7.02E-05	6.80E-03	floral homeotic protein APETALA 2-like
Ma06_g09030	ITC1587_Bchr6_P15445	291.15	1.32	0.41	3.19	1.43E-03	3.91E-02	auxin response factor 7-like
Ma09_g15890	ITC1587_Bchr9_P26474	626.71	1.32	0.48	2.74	6.08E-03	8.61E-02	acetate/butyrate--CoA ligase AAE7
Ma04_g39080	ITC1587_Bchr2_P04285*	224.15	1.32	0.38	3.49	4.90E-04	2.09E-02	LOB domain-containing protein 37-like
Ma10_g03980	ITC1587_Bchr10_P29258	384.42	1.31	0.43	3.08	2.04E-03	4.73E-02	probable thiol methyltransferase 2
Ma09_g08260	ITC1587_Bchr9_P25771	154.25	1.31	0.33	4.03	5.62E-05	6.09E-03	myb-related protein 308-like

Ma06_g19640	ITC1587_Bchr6_P16418	39.67	1.31	0.49	2.67	7.58E-03	9.75E-02	uncharacterized protein At5g39865-like
Ma08_g08120	ITC1587_Bchr8_P22166	49.15	1.31	0.36	3.66	2.51E-04	1.40E-02	receptor-like protein kinase HSL1
Ma07_g11210	ITC1587_Bchr7_P19592	71.47	1.3	0.46	2.84	4.55E-03	7.41E-02	serine/threonine-protein kinase At5g01020-like
Ma09_g06800	ITC1587_Bchr6_P17663*	4967.08	1.3	0.28	4.68	2.87E-06	9.82E-04	carbonic anhydrase 2
Ma04_g01760	ITC1587_Bchr4_P08548	72.78	1.3	0.44	2.97	3.01E-03	5.93E-02	uncharacterized LOC103980321
Ma04_g19490	ITC1587_Bchr4_P10415	1007.78	1.29	0.46	2.82	4.79E-03	7.58E-02	Putative Probable carotenoid cleavage dioxygenase 4
Ma06_g13940	ITC1587_Bchr6_P15886	297.19	1.28	0.41	3.1	1.92E-03	4.55E-02	uncharacterized LOC103987641
Ma08_g04580	ITC1587_Bchr8_P21853	666.44	1.28	0.32	3.99	6.56E-05	6.68E-03	ABC transporter G family member 22-like
Ma01_g01360	ITC1587_Bchr11_P33828*	607.55	1.28	0.32	3.96	7.63E-05	7.11E-03	uncharacterized LOC103982299
Ma01_g16260	ITC1587_Bchr1_P02022	92.29	1.28	0.25	5.18	2.21E-07	2.11E-04	putative MO25-like protein At5g47540
Ma00_g01630	ITC1587_Bchr1_P00886*	18.7	1.27	0.36	3.51	4.41E-04	1.98E-02	Hypothetical protein
Ma05_g26690	ITC1587_Bchr6_P15148*	73.68	1.27	0.39	3.28	1.03E-03	3.20E-02	uncharacterized LOC103985344
Ma01_g00940	ITC1587_Bchr1_P00935	90.34	1.27	0.3	4.25	2.15E-05	3.35E-03	CBL-interacting serine/threonine-protein kinase 21
Ma02_g11960	ITC1587_Bchr2_P04006	126.29	1.26	0.41	3.04	2.33E-03	5.07E-02	cytochrome P450 86B1-like
Ma08_g25160	ITC1587_Bchr8_P24177	3992.9	1.26	0.3	4.18	2.95E-05	4.02E-03	serine--glyoxylate aminotransferase
Ma07_g03700	ITC1587_Bchr7_P18873	261.96	1.24	0.28	4.43	9.51E-06	2.11E-03	probable allantoate deiminase
Ma04_g11810	ITC1587_Bchr4_P09454	37.86	1.24	0.41	3.04	2.37E-03	5.12E-02	uncharacterized LOC103981226
Ma04_g25620	ITC1587_Bchr1_P00553*	101.5	1.23	0.37	3.35	8.16E-04	2.81E-02	Hypothetical protein
Ma06_g29120	ITC1587_Bchr6_P17663	9226.01	1.23	0.45	2.72	6.57E-03	9.05E-02	carbonic anhydrase 2-like
Ma04_g03310	ITC1587_Bchr4_P08682	136.62	1.23	0.36	3.44	5.89E-04	2.34E-02	glucan endo-1, 3-beta-glucosidase 7-like
Ma05_g15800	ITC1587_Bchr5_P13135	8924.29	1.23	0.29	4.18	2.86E-05	3.95E-03	catalase isozyme 2
Ma05_g12480	ITC1587_BchrUn_random _P35179*	30.24	1.22	0.45	2.69	7.13E-03	9.48E-02	expansin-like B1
Ma09_g19630	ITC1587_Bchr9_P26735	29.69	1.22	0.39	3.11	1.89E-03	4.53E-02	scarecrow-like protein 8
Ma01_g16150	ITC1587_Bchr1_P02012*	286.57	1.22	0.44	2.77	5.65E-03	8.25E-02	polyol transporter 5-like
Ma08_g22300	ITC1587_Bchr8_P23926*	357.8	1.21	0.28	4.34	1.45E-05	2.64E-03	monoacylglycerol lipase abhd6-A
Ma03_g18730	ITC1587_BchrUn_random _P38210	145.06	1.21	0.39	3.08	2.05E-03	4.73E-02	probable protein phosphatase 2C 68
Ma06_g13850	ITC1587_Bchr6_P15877	353.14	1.2	0.34	3.55	3.88E-04	1.86E-02	Stromal 70 kDa heat shock-related protein

Ma01_g10350	ITC1587_Bchr1_P01490	70.2	1.19	0.41	2.91	3.62E-03	6.54E-02	putative small heat shock protein
Ma04_g10370	ITC1587_Bchr4_P09304	1536.51	1.19	0.36	3.31	9.28E-04	3.02E-02	anthocyanidin 5, 3-O-glucosyltransferase-like
Ma10_g07810	ITC1587_Bchr10_P29542	76.81	1.19	0.35	3.43	6.09E-04	2.38E-02	zinc finger protein ZAT5-like
Ma09_g14370	ITC1587_Bchr9_P26312	86.7	1.19	0.34	3.54	4.05E-04	1.90E-02	homeobox-leucine zipper protein HOX4-like
Ma03_g22470	ITC1587_Bchr3_P07437	59.73	1.19	0.42	2.84	4.56E-03	7.43E-02	uncharacterized LOC103979186
Ma01_g00430	ITC1587_Bchr1_P00886	1107.44	1.19	0.43	2.75	6.03E-03	8.58E-02	putative UDP-glucose glucosyltransferase
Ma09_g12900	ITC1587_Bchr9_P26179	267.25	1.18	0.42	2.85	4.36E-03	7.23E-02	CBL-interacting serine/threonine-protein kinase 5-like
Ma10_g13630	ITC1587_Bchr10_P30106	13.09	1.18	0.44	2.67	7.68E-03	9.83E-02	Aquaporin TIP1-3
Ma04_g37190	ITC1587_Bchr4_P11429	181.59	1.18	0.42	2.81	5.03E-03	7.73E-02	zinc finger protein CONSTANS-LIKE 16-like
Ma06_g08050	ITC1587_Bchr6_P15353	605.72	1.18	0.29	4.02	5.83E-05	6.27E-03	uncharacterized LOC103987119
Ma10_g10330	ITC1587_Bchr10_P29766	224.81	1.17	0.42	2.8	5.07E-03	7.77E-02	beta-amylase-like
Ma06_g33490	ITC1587_Bchr9_P25534*	507.49	1.17	0.24	4.86	1.15E-06	5.46E-04	uncharacterized LOC103989609
Ma04_g03160	ITC1587_BchrUn_random_P39147*	27.9	1.16	0.4	2.91	3.65E-03	6.56E-02	methylesterase 3-like
Ma08_g24440	ITC1587_Bchr8_P24119	98.7	1.16	0.4	2.92	3.52E-03	6.44E-02	18.6 kDa class III heat shock protein
Ma10_g02900	ITC1587_Bchr10_P29012	95.43	1.15	0.37	3.1	1.90E-03	4.54E-02	uncharacterized LOC103999850
Ma00_g01310	ITC1587_BchrUn_random_P35751	142.7	1.15	0.4	2.91	3.65E-03	6.56E-02	receptor-like cytosolic serine/threonine-protein kinase RBK2
Ma01_g07300	ITC1587_Bchr4_P09107*	79.63	1.14	0.32	3.52	4.25E-04	1.94E-02	calcineurin B-like protein 4
Ma04_g10380	ITC1587_Bchr4_P09304*	422.5	1.14	0.34	3.32	8.92E-04	2.96E-02	UDP-glycosyltransferase 88A1-like
Ma07_g10070	ITC1587_Bchr7_P19499	72.26	1.13	0.42	2.69	7.19E-03	9.51E-02	uncharacterized LOC103991312
Ma07_g20170	ITC1587_Bchr7_P20677	416.38	1.13	0.2	5.6	2.13E-08	4.36E-05	uncharacterized LOC103992199
Ma09_g05840	ITC1587_Bchr9_P25568	121.77	1.13	0.38	2.94	3.33E-03	6.22E-02	probable glutamate carboxypeptidase 2
Ma11_g08230	ITC1587_Bchr11_P32485	344.4	1.12	0.29	3.87	1.07E-04	8.64E-03	putative quinone-oxidoreductase homolog
Ma08_g00800	ITC1587_Bchr8_P22197*	60.53	1.12	0.35	3.17	1.54E-03	4.09E-02	E3 ubiquitin-protein ligase RNF4-like
Ma08_g20860	ITC1587_Bchr8_P23775	67.06	1.12	0.33	3.4	6.67E-04	2.49E-02	transcription factor MYB1R1-like
Ma06_g08930	ITC1587_Bchr6_P15433	597.57	1.12	0.32	3.53	4.20E-04	1.93E-02	potassium channel AKT2-like
Ma08_g16900	ITC1587_BchrUn_random_P38434	676.49	1.12	0.26	4.23	2.32E-05	3.50E-03	uncharacterized LOC103974734

Ma02_g17740	ITC1587_Bchr2_P04501	250.79	1.11	0.27	4.08	4.48E-05	5.24E-03	UDP-glycosyltransferase 73C6-like
Ma03_g14610	ITC1587_Bchr3_P06604	1546.5	1.1	0.23	4.78	1.77E-06	7.49E-04	uncharacterized LOC103978260
Ma07_g12340	ITC1587_Bchr7_P19705	242.26	1.1	0.27	3.99	6.71E-05	6.75E-03	protein TIFY 6B-like
Ma01_g18220	ITC1587_Bchr1_P02174	4363.77	1.1	0.34	3.21	1.35E-03	3.77E-02	CBS domain-containing protein CBSX1
Ma09_g27300	ITC1587_Bchr9_P27962*	35.1	1.09	0.39	2.83	4.65E-03	7.50E-02	germacrene A oxidase-like
Ma10_g29540	ITC1587_Bchr10_P31495	67.62	1.09	0.27	4.09	4.40E-05	5.24E-03	thioredoxin-like 3-1
Ma08_g05870	ITC1587_Bchr3_P07734*	911.08	1.09	0.27	4.06	4.85E-05	5.57E-03	uncharacterized LOC103993726
Ma11_g13780	ITC1587_Bchr11_P33360	2680.75	1.09	0.28	3.92	8.72E-05	7.78E-03	BTB/POZ and TAZ domain-containing protein 1-like
Ma07_g13760	ITC1587_Bchr7_P19840	74.35	1.08	0.34	3.16	1.56E-03	4.10E-02	uncharacterized LOC103991616
Ma07_g06700	ITC1587_Bchr7_P19145	781.33	1.08	0.38	2.87	4.15E-03	7.03E-02	cytochrome P450 89A2-like
Ma10_g27410	ITC1587_Bchr10_P31308	415.32	1.07	0.24	4.46	8.20E-06	1.95E-03	uncharacterized LOC103969387
Ma07_g11390	ITC1587_Bchr7_P19607	174.9	1.07	0.29	3.69	2.26E-04	1.31E-02	uncharacterized protein OsI_027940-like
Ma05_g06670	ITC1587_Bchr5_P12277	79.51	1.07	0.38	2.79	5.20E-03	7.91E-02	rho guanine nucleotide exchange factor 8-like
Ma06_g07010	ITC1587_Bchr6_P15256	828.47	1.07	0.39	2.73	6.42E-03	8.89E-02	cyanidin 3-O-rutinoside 5-O-glucosyltransferase-like
Ma04_g25580	ITC1587_Bchr1_P00553	1460.37	1.06	0.28	3.81	1.36E-04	1.00E-02	uncharacterized aarF domain-containing protein kinase At1g79600
Ma10_g16050	ITC1587_Bchr10_P30324*	122.1	1.05	0.31	3.43	6.07E-04	2.38E-02	Transcription repressor MYB4
Ma09_g28690	ITC1587_Bchr9_P28091	668.88	1.05	0.27	3.85	1.19E-04	9.13E-03	putative disease resistance protein RPM1
Ma09_g00790	ITC1587_Bchr9_P25133	468.69	1.04	0.33	3.12	1.80E-03	4.43E-02	protein EXECUTER 1
Ma10_g15710	ITC1587_Bchr10_P30300	285.72	1.04	0.31	3.37	7.61E-04	2.69E-02	heme-binding-like protein At3g10130
Ma02_g10490	ITC1587_BchrUn_random_P39260	1008.59	1.03	0.29	3.53	4.19E-04	1.93E-02	uncharacterized LOC103975211
Ma11_g20890	ITC1587_Bchr11_P34045	981.24	1.03	0.31	3.29	1.02E-03	3.18E-02	serine carboxypeptidase-like 18
Ma07_g13740	ITC1587_Bchr7_P19838	1617.49	1.03	0.25	4.06	5.01E-05	5.66E-03	CBL-interacting protein kinase 5-like
Ma06_g29770	ITC1587_Bchr6_P17724	226.15	1.02	0.27	3.8	1.48E-04	1.05E-02	serine/threonine-protein kinase BLUS1
Ma06_g30910	ITC1587_Bchr6_P17825	890.32	1.02	0.26	3.91	9.26E-05	7.97E-03	dnaJ homolog subfamily B member 14
Ma02_g11580	ITC1587_Bchr2_P03976	153.54	1.01	0.29	3.44	5.73E-04	2.30E-02	glycerol-3-phosphate acyltransferase
Ma03_g21160	ITC1587_Bchr3_P07325	94.75	1.01	0.33	3.12	1.84E-03	4.47E-02	3-ketodihydrosphingosine reductase-like
Ma03_g11100	ITC1587_Bchr3_P06188	254.96	1.01	0.31	3.22	1.29E-03	3.68E-02	cation/H( ) antiporter 15-like

Ma11_g07820	ITC1587_Bchr11_P32446	734.2	1.01	0.29	3.43	6.12E-04	2.38E-02	putative zinc finger protein CONSTANS-LIKE 5
Ma03_g15000	ITC1587_Bchr3_P06550	82.36	1.01	0.3	3.4	6.68E-04	2.49E-02	uncharacterized LOC103978232
Ma08_g16890	ITC1587_BchrUn_random_P38830	600.82	1.01	0.24	4.26	2.06E-05	3.27E-03	replication factor C subunit 3-like
Ma06_g08780	ITC1587_Bchr6_P15417	547.4	1.01	0.31	3.21	1.35E-03	3.77E-02	BEL1-like homeodomain protein 1
Ma08_g22980	ITC1587_Bchr8_P23987	141.63	1.01	0.29	3.5	4.73E-04	2.05E-02	uncharacterized LOC103995318
Ma09_g06240	ITC1587_Bchr9_P25599	327.78	1.01	0.23	4.36	1.29E-05	2.49E-03	phosphoenolpyruvate carboxylase 2-like
Ma10_g23450	ITC1587_Bchr10_P30959	970.21	1	0.29	3.49	4.90E-04	2.09E-02	uncharacterized LOC103969061
Ma09_g27710	ITC1587_Bchr9_P28000	661.26	1	0.31	3.29	1.01E-03	3.16E-02	protein WEAK CHLOROPLAST MOVEMENT UNDER BLUE LIGHT 1-like
Ma04_g16190	ITC1587_Bchr4_P09981	3502.21	1	0.37	2.68	7.35E-03	9.62E-02	Sucrose-phosphate synthase
Ma08_g11400	ITC1587_Bchr8_P22477*	108.6	1	0.25	4.03	5.55E-05	6.05E-03	uncharacterized LOC103993238
Ma03_g16540	ITC1587_Bchr3_P06912	287.34	1	0.23	4.38	1.17E-05	2.43E-03	chaperone protein ClpB1-like
Ma03_g26030	ITC1587_Bchr3_P07734	138.29	1	0.32	3.09	2.01E-03	4.70E-02	uncharacterized LOC103978888
Ma07_g24540	ITC1587_Bchr7_P21069*	551.99	0.99	0.25	4.01	6.18E-05	6.49E-03	RING finger protein 141-like
Ma06_g03330	ITC1587_Bchr6_P14920	77.17	0.99	0.35	2.86	4.18E-03	7.06E-02	pentatricopeptide repeat-containing protein At1g15510
Ma10_g11130	ITC1587_Bchr10_P29903	5660.95	0.99	0.34	2.93	3.39E-03	6.27E-02	GDP-L-galactose phosphorylase 1-like
Ma06_g28850	ITC1587_Bchr6_P17637	956.86	0.99	0.24	4.16	3.24E-05	4.26E-03	probable solanesyl-diphosphate synthase 3
Ma09_g08850	ITC1587_Bchr9_P25823	109.49	0.99	0.35	2.82	4.83E-03	7.60E-02	nodulation receptor kinase-like
Ma10_g28290	ITC1587_Bchr10_P31383	537.23	0.99	0.23	4.26	2.05E-05	3.27E-03	crocetin glucosyltransferase
Ma09_g09320	ITC1587_Bchr9_P25865	989.79	0.98	0.19	5.04	4.74E-07	3.17E-04	thioredoxin-like protein HCF164
Ma05_g31110	ITC1587_Bchr5_P14697	137.72	0.97	0.31	3.11	1.89E-03	4.53E-02	aldo-keto reductase family 4 member C9-like
Ma02_g06570	ITC1587_Bchr2_P03523	547.23	0.97	0.24	3.98	7.00E-05	6.80E-03	uncharacterized LOC103972089
Ma01_g19250	ITC1587_Bchr1_P02324	806.69	0.97	0.23	4.14	3.42E-05	4.45E-03	Hypothetical protein
Ma05_g25750	ITC1587_Bchr5_P14213	293.44	0.97	0.32	3	2.70E-03	5.55E-02	aldose 1-epimerase-like
Ma08_g10000	ITC1587_Bchr8_P22338*	32.61	0.97	0.31	3.16	1.56E-03	4.10E-02	putative protein pleiotropic regulator PRL2
Ma04_g17140	ITC1587_Bchr1_P00130*	46.13	0.96	0.31	3.15	1.64E-03	4.22E-02	trans-resveratrol di-O-methyltransferase-like

Ma04_g09240	ITC1587_Bchr4_P09206	1598.2	0.96	0.29	3.35	7.94E-04	2.77E-02	ABC1 kinase
Ma06_g23690	ITC1587_Bchr9_P26054*	41.35	0.96	0.31	3.12	1.80E-03	4.43E-02	uncharacterized LOC103988740
Ma08_g04770	ITC1587_Bchr8_P21871	2992.96	0.96	0.25	3.8	1.44E-04	1.03E-02	4-hydroxy-3-methylbut-2-enyl diphosphate reductase
Ma09_g24060	ITC1587_Bchr9_P27655	650.81	0.96	0.35	2.73	6.37E-03	8.86E-02	No apical meristem protein
Ma11_g03040	ITC1587_Bchr11_P31975	169.56	0.95	0.31	3.08	2.04E-03	4.73E-02	uncharacterized LOC103970289
Ma02_g23870	ITC1587_Bchr2_P05052	68.78	0.95	0.36	2.67	7.64E-03	9.79E-02	putative Myb-related protein 306
Ma07_g00370	ITC1587_Bchr7_P18584	154.37	0.95	0.27	3.48	4.95E-04	2.10E-02	uncharacterized LOC103991016
Ma07_g17820	ITC1587_BchrUn_random _P38528	301.07	0.94	0.27	3.51	4.48E-04	2.00E-02	probable pyridoxal biosynthesis protein PDX1.1
Ma09_g18030	ITC1587_BchrUn_random _P35238*	70.9	0.94	0.32	2.99	2.76E-03	5.66E-02	uncharacterized LOC103973521
Ma09_g03450	ITC1587_Bchr9_P25377	1626.97	0.94	0.33	2.89	3.90E-03	6.79E-02	tropinone reductase homolog At1g07440-like
Ma03_g33240	ITC1587_Bchr3_P08330	511.66	0.94	0.33	2.86	4.21E-03	7.08E-02	probable protein phosphatase 2C 51
Ma10_g25240	ITC1587_Bchr10_P31117*	85.68	0.94	0.28	3.42	6.34E-04	2.44E-02	pheophytinase
Ma06_g17610	ITC1587_Bchr6_P16213	93.15	0.94	0.27	3.47	5.21E-04	2.17E-02	E3 ubiquitin-protein ligase SGR9
Ma04_g22180	ITC1587_Bchr1_P00221	294.65	0.94	0.28	3.4	6.64E-04	2.49E-02	PTI1-like tyrosine-protein kinase 2
Ma11_g09700	ITC1587_Bchr11_P32968	379.24	0.94	0.23	4.05	5.12E-05	5.71E-03	E3 ubiquitin-protein ligase CHIP-like
Ma10_g05590	ITC1587_Bchr10_P28478	1868.73	0.93	0.32	2.95	3.22E-03	6.15E-02	phosphate metabolism protein 8-like
Ma08_g20030	ITC1587_Bchr8_P22917	164.37	0.93	0.25	3.69	2.23E-04	1.30E-02	pheophytinase
Ma07_g09210	ITC1587_Bchr7_P19428	524.03	0.93	0.28	3.31	9.45E-04	3.04E-02	Peroxidase 52
Ma04_g35450	ITC1587_Bchr4_P11284	99.74	0.93	0.34	2.77	5.52E-03	8.17E-02	metalloendoproteinase 1-like
Ma09_g28220	ITC1587_Bchr9_P28047	393.44	0.93	0.2	4.59	4.40E-06	1.26E-03	uncharacterized LOC103999206
Ma01_g14090	ITC1587_Bchr1_P01821*	343.24	0.92	0.31	2.99	2.77E-03	5.67E-02	putative receptor-like protein kinase At4g00960
Ma10_g14740	ITC1587_Bchr10_P30213	1544.31	0.92	0.34	2.74	6.12E-03	8.64E-02	ABC transporter C family member 10
Ma03_g11970	ITC1587_Bchr3_P06263	744.65	0.92	0.21	4.3	1.73E-05	2.91E-03	beta-carotene isomerase D27
Ma05_g22300	ITC1587_Bchr5_P13862	7024.29	0.92	0.32	2.85	4.41E-03	7.27E-02	fructose-bisphosphate aldolase 1
Ma08_g04920	ITC1587_Bchr3_P07822*	290.11	0.91	0.24	3.75	1.79E-04	1.18E-02	squalene monooxygenase-like
Ma05_g17450	ITC1587_Bchr5_P13538	247.93	0.91	0.23	3.97	7.13E-05	6.84E-03	junctophilin-1-like

Ma10_g09280	ITC1587_Bchr7_P20253*	106.36	0.91	0.28	3.29	9.90E-04	3.14E-02	probable LRR receptor-like serine/threonine-protein kinase At1g56130
Ma10_g05910	ITC1587_Bchr10_P28438	1517.11	0.9	0.25	3.61	3.08E-04	1.60E-02	tropinone reductase homolog At1g07440-like
Ma01_g18420	ITC1587_Bchr1_P02154	119.23	0.9	0.3	3.04	2.40E-03	5.15E-02	haloacid dehalogenase-like hydrolase domain-containing protein Sgpp
Ma09_g10470	ITC1587_Bchr9_P25967	3425.08	0.9	0.34	2.67	7.67E-03	9.83E-02	putative horcolin
Ma09_g15590	ITC1587_Bchr9_P26436	39.77	0.89	0.32	2.84	4.57E-03	7.43E-02	target of Myb protein 1-like
Ma04_g04500	ITC1587_Bchr4_P08787	85.86	0.89	0.25	3.51	4.43E-04	1.98E-02	mitogen-activated protein kinase homolog MMK2-like
Ma00_g02750	ITC1587_Bchr9_P27277	1225.26	0.89	0.3	2.98	2.92E-03	5.84E-02	ABC transporter F family member 4-like
Ma09_g17390	ITC1587_Bchr9_P26599	280.02	0.89	0.28	3.16	1.57E-03	4.10E-02	uncharacterized LOC103998278
Ma03_g26110	ITC1587_Bchr3_P07738	3038	0.89	0.31	2.83	4.72E-03	7.54E-02	Pyruvate, phosphate dikinase 1
Ma10_g29920	ITC1587_Bchr10_P31532	261.82	0.89	0.31	2.82	4.83E-03	7.60E-02	zinc finger protein CONSTANS-LIKE 16-like
Ma03_g26080	ITC1587_Bchr2_P03269*	366.37	0.89	0.31	2.89	3.89E-03	6.79E-02	pyruvate, phosphate dikinase
Ma05_g23360	ITC1587_Bchr11_P33897*	387.71	0.89	0.19	4.66	3.23E-06	1.08E-03	shaggy-related protein kinase gamma
Ma04_g16470	ITC1587_BchrUn_random_P36409	118.98	0.88	0.3	2.9	3.68E-03	6.58E-02	uncharacterized LOC103973729
Ma05_g18830	ITC1587_Bchr5_P13157	1205.81	0.88	0.31	2.8	5.16E-03	7.87E-02	IN2-2 protein-like
Ma03_g10090	ITC1587_Bchr3_P06097	219.88	0.87	0.21	4.24	2.22E-05	3.42E-03	uncharacterized LOC103977829
Ma06_g14890	ITC1587_Bchr6_P15968	764.01	0.87	0.24	3.69	2.21E-04	1.30E-02	uncharacterized LOC103987719
Ma01_g14940	ITC1587_BchrUn_random_P36900*	4166.01	0.87	0.22	3.96	7.42E-05	7.04E-03	inositol-3-phosphate synthase-like
Ma01_g07930	ITC1587_Bchr1_P01280	217.97	0.87	0.26	3.34	8.32E-04	2.84E-02	phospholipase A1-II 5
Ma10_g21860	ITC1587_Bchr10_P30820	137.3	0.87	0.3	2.88	3.96E-03	6.85E-02	mitochondrial carrier protein MTM1-like
Ma09_g10410	ITC1587_Bchr9_P25961	4842.76	0.87	0.32	2.74	6.22E-03	8.74E-02	putative horcolin
Ma08_g29220	ITC1587_Bchr8_P24550	690.53	0.86	0.24	3.64	2.70E-04	1.48E-02	uncharacterized LOC103996007
Ma08_g19610	ITC1587_Bchr8_P22966	5426.1	0.86	0.24	3.53	4.15E-04	1.92E-02	Alanine aminotransferase 2
Ma10_g25540	ITC1587_Bchr10_P31137	171.2	0.86	0.25	3.39	7.10E-04	2.58E-02	ultraviolet-B receptor UVR8
Ma11_g00990	ITC1587_Bchr11_P31777	381.79	0.86	0.27	3.17	1.51E-03	4.03E-02	serine carboxypeptidase-like 7
Ma06_g32780	ITC1587_Bchr6_P18002	266.55	0.86	0.3	2.86	4.18E-03	7.06E-02	protein YABBY 2
Ma01_g00570	ITC1587_Bchr1_P00901	1015.07	0.86	0.25	3.45	5.62E-04	2.27E-02	uncharacterized LOC103981509

Ma10_g30250	ITC1587_Bchr10_P31073*	219.82	0.85	0.27	3.18	1.47E-03	3.95E-02	dicarboxylate transporter 2.1
Ma11_g05350	ITC1587_Bchr11_P32196	1668.23	0.85	0.28	3.04	2.40E-03	5.15E-02	beta-glucosidase 22-like
Ma06_g02840	ITC1587_Bchr6_P14880	544.92	0.85	0.24	3.54	4.02E-04	1.90E-02	uncharacterized LOC103986630
Ma09_g01580	ITC1587_Bchr9_P25208	538.98	0.85	0.32	2.67	7.56E-03	9.74E-02	putative G3BP-like protein
Ma01_g05380	ITC1587_Bchr1_P01047	1937.99	0.85	0.26	3.33	8.77E-04	2.93E-02	glucose-1-phosphate adenylyltransferase large subunit 3
Ma07_g24710	ITC1587_Bchr7_P21086	887.03	0.85	0.26	3.23	1.22E-03	3.57E-02	NAC domain-containing protein 48
Ma07_g02560	ITC1587_BchrUn_random_P35408*	246.12	0.85	0.23	3.71	2.05E-04	1.26E-02	uncharacterized LOC103990825
Ma00_g01620	ITC1587_BchrUn_random_P34622*	92.55	0.84	0.28	3.06	2.20E-03	4.91E-02	putative limonoid UDP-glucosyltransferase
Ma08_g27550	ITC1587_Bchr8_P24399	608.46	0.84	0.23	3.69	2.24E-04	1.30E-02	uncharacterized LOC103996158
Ma05_g29280	ITC1587_Bchr5_P14535	88.27	0.84	0.29	2.9	3.71E-03	6.58E-02	squamosa promoter-binding-like protein 9
Ma09_g26520	ITC1587_Bchr9_P27899	2689.47	0.84	0.32	2.66	7.91E-03	9.98E-02	Probable glutathione S-transferase parA
Ma07_g21340	ITC1587_Bchr7_P20795	354.28	0.84	0.3	2.77	5.66E-03	8.27E-02	uncharacterized LOC103992335
Ma06_g18840	ITC1587_Bchr2_P04953*	2037.64	0.84	0.18	4.6	4.28E-06	1.26E-03	putative 12-oxophytodienoate reductase 11
Ma02_g02430	ITC1587_BchrUn_random_P34535*	144.3	0.84	0.26	3.26	1.10E-03	3.33E-02	G-type lectin S-receptor-like serine/threonine-protein kinase At4g03230
Ma06_g33250	ITC1587_Bchr6_P18049	552.46	0.84	0.28	3.03	2.49E-03	5.25E-02	phytoene synthase 2
Ma06_g05630	ITC1587_Bchr6_P15148	210.81	0.83	0.26	3.16	1.57E-03	4.11E-02	uncharacterized LOC103986915
Ma01_g11590	ITC1587_Bchr1_P01610*	193.42	0.83	0.31	2.71	6.64E-03	9.10E-02	uncharacterized protein At5g50100
Ma11_g11780	ITC1587_Bchr11_P33130	433.55	0.83	0.26	3.14	1.66E-03	4.24E-02	uncharacterized LOC103971356
Ma10_g16680	ITC1587_Bchr10_P30381	200.31	0.83	0.29	2.83	4.64E-03	7.50E-02	small heat shock protein
Ma09_g07230	ITC1587_Bchr9_P25690	314.38	0.83	0.19	4.31	1.61E-05	2.82E-03	uncharacterized LOC103997302
Ma03_g17480	ITC1587_Bchr3_P06896	1393.48	0.83	0.27	3.07	2.16E-03	4.85E-02	F-box/kelch-repeat protein At1g67480-like
Ma11_g20830	ITC1587_Bchr11_P34039	205.49	0.83	0.31	2.67	7.55E-03	9.74E-02	15-cis-phytoene desaturase
Ma09_g09060	ITC1587_Bchr9_P25840	1053.08	0.83	0.25	3.28	1.05E-03	3.24E-02	cyclic nucleotide-gated ion channel 2-like
Ma10_g31280	ITC1587_Bchr10_P31664	123.72	0.82	0.23	3.55	3.79E-04	1.83E-02	ubiquitin-conjugating enzyme E2-23 kDa-like
Ma03_g24320	ITC1587_BchrUn_random_P38239	82.64	0.82	0.24	3.47	5.23E-04	2.17E-02	phosphatidate cytidylyltransferase-like

Ma04_g36740	ITC1587_Bchr4_P11394	325.09	0.82	0.2	4.11	3.90E-05	4.86E-03	protein LOW PSII ACCUMULATION 1
Ma07_g01600	ITC1587_Bchr7_P18692	66.35	0.82	0.28	2.91	3.66E-03	6.56E-02	pentatricopeptide repeat-containing protein At2g42920
Ma07_g07720	ITC1587_Bchr7_P19254	300.16	0.82	0.21	3.98	6.91E-05	6.80E-03	folate-biopterin transporter 1
Ma00_g00210	ITC1587_Bchr8_P24432*	98.05	0.82	0.23	3.63	2.85E-04	1.52E-02	2-aminoethanethiol dioxygenase-like
Ma03_g09400	ITC1587_Bchr3_P06033	597.58	0.82	0.2	4.09	4.39E-05	5.24E-03	repressor of RNA polymerase III transcription MAF1 homolog
Ma08_g02940	ITC1587_Bchr8_P21724*	134.58	0.82	0.25	3.32	9.15E-04	2.99E-02	uncharacterized LOC103993976
Ma11_g02550	ITC1587_Bchr11_P31924	132.38	0.82	0.3	2.71	6.71E-03	9.16E-02	uncharacterized calcium-binding protein At1g02270-like
Ma07_g09470	ITC1587_Bchr7_P19453	396.57	0.81	0.26	3.14	1.66E-03	4.24E-02	cytochrome P450 90D2-like
Ma07_g26010	ITC1587_Bchr7_P21209	835.53	0.81	0.26	3.14	1.71E-03	4.33E-02	RNA polymerase sigma factor sigE
Ma10_g27480	ITC1587_Bchr10_P31312*	313.43	0.81	0.23	3.49	4.84E-04	2.08E-02	probable mitochondrial adenine nucleotide transporter BTL3
Ma01_g00790	ITC1587_Bchr1_P00923	1410.77	0.81	0.22	3.62	2.96E-04	1.55E-02	chaperone protein dnaJ 8
Ma11_g06470		198.59	0.81	0.28	2.86	4.21E-03	7.08E-02	uncharacterized LOC103970577
Ma08_g21280	ITC1587_Bchr8_P23742	2189.89	0.81	0.27	3.01	2.63E-03	5.45E-02	probable tocopherol cyclase
Ma07_g25830	ITC1587_Bchr7_P21189	275.12	0.81	0.27	3.02	2.55E-03	5.32E-02	uncharacterized LOC103992727
Ma07_g28490	ITC1587_Bchr7_P21465*	70.06	0.8	0.28	2.86	4.30E-03	7.17E-02	oxidoreductase, aldo/keto reductase family protein
Ma04_g10650	ITC1587_Bchr4_P09337	783.29	0.8	0.25	3.15	1.62E-03	4.20E-02	2-dihydroxy-3-keto-5-methylthiopentene dioxygenase 1-like
Ma03_g01310	ITC1587_Bchr3_P05286	1507.06	0.8	0.21	3.71	2.03E-04	1.26E-02	uncharacterized LOC103977207
Ma06_g28550	ITC1587_Bchr6_P17607	550.79	0.8	0.23	3.44	5.83E-04	2.32E-02	phosphoenolpyruvate carboxylase 2-like
Ma07_g23140	ITC1587_Bchr7_P20939	1201.93	0.8	0.21	3.71	2.06E-04	1.26E-02	protein TRANSPARENT TESTA 12-like
Ma03_g12570	ITC1587_Bchr3_P06313	29.26	0.8	0.28	2.88	4.03E-03	6.93E-02	uncharacterized LOC103978025
Ma01_g15200	ITC1587_Bchr8_P24716*	68.49	0.79	0.28	2.82	4.84E-03	7.61E-02	uncharacterized LOC103991302
Ma09_g17750	ITC1587_Bchr9_P26635	1827.96	0.79	0.2	3.87	1.07E-04	8.64E-03	homeobox-leucine zipper protein HOX16-like
Ma09_g06980	ITC1587_Bchr6_P15345*	228.14	0.79	0.2	3.96	7.38E-05	7.04E-03	probable WRKY transcription factor 75
Ma08_g29400	ITC1587_Bchr8_P24564*	3003.03	0.79	0.23	3.4	6.84E-04	2.54E-02	phosphoglycolate phosphatase 1B
Ma03_g14950	ITC1587_Bchr3_P06554	890.11	0.79	0.19	4.16	3.19E-05	4.24E-03	uncharacterized LOC103978235
Ma03_g23770	ITC1587_Bchr3_P07542	483.46	0.79	0.19	4.24	2.25E-05	3.46E-03	uncharacterized LOC103979087

Ma04_g13320	ITC1587_Bchr4_P09589	751.57	0.79	0.22	3.58	3.43E-04	1.72E-02	protein kinase and PP2C-like domain-containing protein
Ma08_g14830	ITC1587_Bchr8_P23649*	100.83	0.79	0.28	2.76	5.75E-03	8.34E-02	chorismate synthase 1
Ma07_g20160	ITC1587_Bchr5_P14185*	195.23	0.79	0.2	3.97	7.06E-05	6.80E-03	GDT1-like protein 5
Ma01_g00080	ITC1587_Bchr7_P19115*	117.04	0.79	0.23	3.35	8.07E-04	2.79E-02	serine/threonine-protein phosphatase PP-X isozyme 2-like
Ma01_g00620	ITC1587_Bchr1_P00907	732.61	0.79	0.22	3.57	3.63E-04	1.79E-02	probable anion transporter 6
Ma08_g05160	ITC1587_Bchr8_P21903	663.67	0.78	0.25	3.16	1.58E-03	4.12E-02	cellulose synthase-like protein G3
Ma05_g25910	ITC1587_Bchr5_P14229	73.6	0.78	0.22	3.49	4.83E-04	2.08E-02	uncharacterized LOC103985878
Ma09_g27420	ITC1587_Bchr9_P27974	1418.14	0.78	0.23	3.42	6.15E-04	2.39E-02	uncharacterized LOC103999142
Ma03_g12980	ITC1587_Bchr3_P06351	38.36	0.78	0.29	2.7	7.00E-03	9.38E-02	probable protein phosphatase 2C 27
Ma07_g11550	ITC1587_Bchr7_P19621	1956.15	0.78	0.27	2.91	3.64E-03	6.56E-02	protein TRANSPARENT TESTA 12-like
Ma11_g15570	ITC1587_Bchr11_P33560	428.64	0.78	0.18	4.27	2.00E-05	3.25E-03	thylakoid lumenal 15.0 kDa protein 2
Ma10_g20630	ITC1587_Bchr10_P30716	126.67	0.77	0.2	3.93	8.49E-05	7.62E-03	iron-sulfur assembly protein IscA-like 1
Ma09_g19670	ITC1587_Bchr9_P27124	778.38	0.77	0.22	3.58	3.46E-04	1.73E-02	putative zinc finger protein CONSTANS-LIKE 2
Ma09_g07420	ITC1587_Bchr9_P25701	1443.56	0.77	0.27	2.87	4.10E-03	7.00E-02	protein phosphatase 1 regulatory subunit 12A-like
Ma02_g17020	ITC1587_Bchr2_P04440	384.01	0.77	0.21	3.73	1.93E-04	1.22E-02	probable transcription factor GLK1
Ma07_g26030	ITC1587_Bchr7_P21211	560.58	0.77	0.19	4.16	3.25E-05	4.26E-03	mitochondrial adenine nucleotide transporter ADNT1
Ma06_g35910	ITC1587_Bchr6_P18271	373.43	0.77	0.27	2.8	5.04E-03	7.73E-02	folylpolyglutamate synthase {ECO:0000250|UniProtKB:Q05932
Ma07_g19200	ITC1587_Bchr7_P20576	319.48	0.77	0.25	3.05	2.26E-03	5.00E-02	shikimate kinase 3
Ma04_g01020	ITC1587_Bchr4_P08475	234.16	0.77	0.28	2.79	5.26E-03	7.96E-02	RNA polymerase sigma factor sigC
Ma06_g17060		569.3	0.77	0.2	3.8	1.47E-04	1.05E-02	uncharacterized LOC103987907
Ma01_g07000		203.35	0.77	0.22	3.55	3.80E-04	1.83E-02	uncharacterized LOC103986762
Ma02_g14080	ITC1587_Bchr2_P04175	2809.28	0.77	0.27	2.89	3.83E-03	6.71E-02	PGR5-like protein 1B
Ma03_g15750	ITC1587_Bchr3_P06478	378.86	0.77	0.21	3.59	3.35E-04	1.70E-02	protein LURP-one-related 5-like
Ma11_g22640	ITC1587_Bchr11_P34190	38227.75	0.76	0.28	2.76	5.77E-03	8.36E-02	ribulose bisphosphate carboxylase/oxygenase activase 2
Ma07_g08660	ITC1587_Bchr7_P19372	375.74	0.76	0.2	3.73	1.91E-04	1.22E-02	mitochondrial outer membrane protein porin 1-like

Ma02_g04380	ITC1587_Bchr2_P03314*	825.01	0.76	0.22	3.44	5.76E-04	2.30E-02	receptor-like cytosolic serine/threonine-protein kinase RBK1
Ma09_g07910	ITC1587_Bchr9_P25740	959.16	0.76	0.2	3.78	1.58E-04	1.09E-02	probable carboxylesterase 18
Ma08_g15070	ITC1587_Bchr8_P23675	2995.66	0.76	0.22	3.44	5.77E-04	2.30E-02	phosphoglucomutase
Ma10_g22400	ITC1587_Bchr10_P30871	894.61	0.76	0.17	4.48	7.35E-06	1.85E-03	probable anion transporter 1
Ma03_g17570	ITC1587_Bchr3_P07029	315.59	0.76	0.21	3.56	3.75E-04	1.82E-02	chaperone protein dnaJ 20
Ma06_g18140	ITC1587_Bchr6_P16267	724.17	0.76	0.21	3.61	3.10E-04	1.61E-02	diacylglycerol kinase 1
Ma01_g21420		888	0.76	0.22	3.36	7.66E-04	2.69E-02	uncharacterized LOC103999804
Ma04_g07100	ITC1587_Bchr4_P09007	1862.65	0.75	0.18	4.19	2.81E-05	3.90E-03	tryptophan synthase beta chain 1
Ma03_g06930	ITC1587_Bchr3_P05785	218.04	0.75	0.26	2.9	3.72E-03	6.59E-02	putative abscisic acid-insensitive 5-like protein 5
Ma06_g37480	ITC1587_Bchr6_P18408	341.27	0.75	0.22	3.46	5.43E-04	2.22E-02	probable F-box protein At1g60180
Ma06_g29480	ITC1587_Bchr6_P17692	372.11	0.75	0.23	3.25	1.16E-03	3.45E-02	putative quinone-oxidoreductase homolog
Ma06_g18710	ITC1587_Bchr6_P16326	923.38	0.74	0.2	3.7	2.19E-04	1.30E-02	neutral/alkaline invertase
Ma01_g21470	ITC1587_Bchr8_P24425*	289.65	0.74	0.2	3.78	1.56E-04	1.09E-02	protein DEHYDRATION-INDUCED 19-like
Ma05_g10060	ITC1587_Bchr5_P12579	864.97	0.74	0.25	2.96	3.03E-03	5.94E-02	CBL-interacting protein kinase 9
Ma09_g06790	ITC1587_Bchr9_P25639	383.32	0.74	0.19	3.96	7.65E-05	7.11E-03	phosphoglucan phosphatase LSF1
Ma08_g21090	ITC1587_Bchr8_P23757*	349.15	0.74	0.26	2.83	4.70E-03	7.53E-02	uncharacterized LOC103995066
Ma10_g08890	ITC1587_Bchr10_P29637	722.48	0.74	0.21	3.5	4.74E-04	2.05E-02	probable plastid-lipid-associated protein 6
Ma08_g06120	ITC1587_Bchr8_P21978	4318.13	0.74	0.22	3.31	9.19E-04	2.99E-02	chloroplast stem-loop binding protein of 41 kDa b
Ma09_g03980	ITC1587_Bchr9_P25415	435.64	0.74	0.26	2.81	4.91E-03	7.64E-02	inner membrane protein PPF-1
Ma06_g07890	ITC1587_Bchr6_P15337	2170.76	0.74	0.24	3.07	2.15E-03	4.85E-02	uncharacterized LOC103987105
Ma08_g26550	ITC1587_Bchr4_P10458*	337.03	0.74	0.16	4.56	5.08E-06	1.37E-03	nudix hydrolase 2-like
Ma05_g18580	ITC1587_Bchr5_P13359	403.92	0.74	0.24	3.07	2.15E-03	4.85E-02	uncharacterized LOC103985194
Ma10_g29280	ITC1587_Bchr10_P31478	439.4	0.74	0.27	2.69	7.24E-03	9.53E-02	uncharacterized LOC103969532
Ma05_g17940	ITC1587_Bchr5_P13381	206.61	0.74	0.19	3.83	1.27E-04	9.56E-03	putative NAC domain-containing protein 94
Ma03_g25060	ITC1587_Bchr3_P07651*	429.01	0.73	0.26	2.79	5.21E-03	7.92E-02	uncharacterized LOC103978984
Ma11_g01390	ITC1587_Bchr11_P31815	5729.71	0.73	0.23	3.23	1.24E-03	3.59E-02	glyceraldehyde-3-phosphate dehydrogenase B

Ma04_g38310	ITC1587_Bchr4_P11515*	1900.64	0.73	0.2	3.75	1.79E-04	1.18E-02	uncharacterized oxidoreductase At1g06690
Ma11_g13840	ITC1587_Bchr8_P21560*	746.78	0.73	0.26	2.85	4.39E-03	7.26E-02	thioredoxin
Ma06_g27370	ITC1587_Bchr6_P17504*	1588.08	0.73	0.22	3.35	8.13E-04	2.81E-02	chlorophyllide a oxygenase
Ma07_g25470	ITC1587_Bchr7_P21157	414.55	0.73	0.21	3.47	5.25E-04	2.17E-02	uncharacterized LOC103992695
Ma07_g05260	ITC1587_Bchr7_P19015	971.33	0.73	0.18	4.14	3.42E-05	4.45E-03	homeobox protein knotted-1-like 13
Ma03_g00110	ITC1587_Bchr3_P05177	126.15	0.73	0.22	3.26	1.11E-03	3.35E-02	uncharacterized LOC103977307
Ma11_g10660	ITC1587_Bchr11_P32825	105.5	0.72	0.21	3.46	5.32E-04	2.19E-02	beta-carotene isomerase D27
Ma03_g32320	ITC1587_Bchr3_P08254	157.35	0.72	0.26	2.82	4.80E-03	7.59E-02	pentatricopeptide repeat-containing protein At5g66520
Ma03_g27950	ITC1587_Bchr3_P07907	59.43	0.72	0.23	3.12	1.83E-03	4.46E-02	methyltransferase-like protein 23
Ma08_g01610	ITC1587_Bchr5_P14628*	492.1	0.72	0.22	3.26	1.13E-03	3.40E-02	B3 domain-containing protein Os07g0679700-like
Ma02_g14500	ITC1587_Bchr2_P04215	2720.13	0.71	0.16	4.44	9.06E-06	2.05E-03	2-methyl-6-phytyl-1, 4-hydroquinone methyltransferase 1
Ma04_g20350	ITC1587_Bchr1_P00130	675.2	0.71	0.19	3.7	2.15E-04	1.28E-02	trans-resveratrol di-O-methyltransferase-like
Ma09_g16480	ITC1587_Bchr9_P26565	355.4	0.71	0.23	3.04	2.34E-03	5.08E-02	uncharacterized LOC103998234
Ma04_g14870	ITC1587_Bchr4_P09745*	1522.04	0.71	0.26	2.74	6.17E-03	8.69E-02	carbonic anhydrase
Ma05_g03790	ITC1587_Bchr5_P12009	565.72	0.71	0.17	4.14	3.50E-05	4.51E-03	peptidyl-tRNA hydrolase ICT1
Ma05_g04030	ITC1587_Bchr5_P12036	362.42	0.71	0.17	4.11	4.01E-05	4.93E-03	putative zinc finger protein CONSTANS-LIKE 9
Ma09_g27610	ITC1587_Bchr9_P27991	75.21	0.7	0.25	2.81	4.89E-03	7.64E-02	probable mannan synthase 4
Ma08_g15940	ITC1587_Bchr8_P22791	681.59	0.7	0.23	3.1	1.96E-03	4.63E-02	DNA-binding protein SMUBP-2
Ma03_g12650	ITC1587_Bchr3_P06320	3886.29	0.7	0.25	2.76	5.72E-03	8.31E-02	chromoplast-specific carotenoid-associated protein C1
Ma01_g01370	ITC1587_Bchr1_P00967	85.5	0.7	0.24	2.91	3.64E-03	6.56E-02	pentatricopeptide repeat-containing protein At5g42310
Ma06_g25580	ITC1587_Bchr6_P17337	150.81	0.7	0.17	4.01	6.07E-05	6.42E-03	uncharacterized zinc finger protein At4g06634-like
Ma02_g09730	ITC1587_Bchr2_P03805	300.28	0.7	0.21	3.32	9.06E-04	2.99E-02	calcium-dependent protein kinase 8-like
Ma11_g11100	ITC1587_BchrUn_random_P34470	8086.44	0.7	0.18	3.87	1.09E-04	8.71E-03	protein CHUP1
Ma05_g21470	ITC1587_Bchr5_P13774	543.22	0.7	0.14	5.04	4.76E-07	3.17E-04	probable serine acetyltransferase 2

Ma03_g26760	ITC1587_Bchr3_P07798*	895.71	0.7	0.24	2.9	3.72E-03	6.59E-02	uncharacterized LOC103978816
Ma06_g22010	ITC1587_BchrUn_random_P38646	486.26	0.7	0.24	2.95	3.18E-03	6.10E-02	violaxanthin de-epoxidase
Ma09_g14950	ITC1587_Bchr9_P26372*	697.73	0.7	0.22	3.16	1.58E-03	4.12E-02	uncharacterized LOC103998009
Ma06_g31950	ITC1587_Bchr5_P13323*	272.97	0.7	0.22	3.1	1.92E-03	4.55E-02	E3 ubiquitin-protein ligase RLIM-like
Ma05_g30480	ITC1587_Bchr11_P34228*	1666.65	0.7	0.24	2.89	3.91E-03	6.81E-02	6-phosphofructo-2-kinase/fructose-2
Ma11_g15550	ITC1587_Bchr11_P33559*	223.72	0.7	0.25	2.82	4.78E-03	7.58E-02	uncharacterized LOC103971677
Ma06_g05400	ITC1587_Bchr5_P14153*	625.57	0.69	0.19	3.69	2.22E-04	1.30E-02	probable protein phosphatase 2C 59
Ma06_g19830	ITC1587_Bchr6_P16441	2174.71	0.69	0.16	4.33	1.50E-05	2.69E-03	transmembrane protein 64
Ma03_g30980	ITC1587_Bchr3_P08141	3048.39	0.69	0.18	3.89	9.95E-05	8.37E-03	Carotenoid 9
Ma11_g17090	ITC1587_Bchr11_P33698	310.78	0.69	0.22	3.19	1.45E-03	3.93E-02	uncharacterized LOC103971804
Ma04_g26120	ITC1587_Bchr4_P10454	1323.08	0.69	0.23	2.94	3.27E-03	6.19E-02	probable galactinol--sucrose galactosyltransferase 2
Ma06_g36220	ITC1587_Bchr9_P25354*	282.84	0.69	0.21	3.21	1.31E-03	3.70E-02	ras-related protein RABE1c-like
Ma03_g16640	ITC1587_Bchr5_P12284*	361.45	0.69	0.21	3.33	8.79E-04	2.93E-02	E3 ubiquitin-protein ligase SINAT5-like
Ma08_g24540	ITC1587_Bchr7_P20021*	310.27	0.69	0.23	3.03	2.44E-03	5.20E-02	protein TIFY 3B-like
Ma07_g20780	ITC1587_Bchr10_P31604*	220.54	0.69	0.24	2.85	4.38E-03	7.26E-02	neural Wiskott-Aldrich syndrome protein-like
Ma06_g33940	ITC1587_Bchr6_P18111	111.86	0.69	0.24	2.9	3.75E-03	6.61E-02	putative predicted protein
Ma06_g00160	ITC1587_Bchr11_P32604	321.36	0.69	0.17	3.96	7.54E-05	7.08E-03	vacuolar amino acid transporter 1
Ma08_g18080	ITC1587_BchrUn_random_P34651	87.81	0.68	0.25	2.76	5.84E-03	8.44E-02	E3 ubiquitin-protein ligase COP1-like
Ma01_g12150	ITC1587_Bchr1_P01665	336.17	0.68	0.25	2.69	7.16E-03	9.50E-02	histone H1
Ma11_g16990	ITC1587_Bchr11_P33689	417.32	0.68	0.22	3.13	1.72E-03	4.34E-02	auxin response factor 17-like
Ma02_g13970	ITC1587_Bchr11_P32416*	581.35	0.68	0.25	2.78	5.52E-03	8.17E-02	ribose-phosphate pyrophosphokinase 1
Ma06_g38260	ITC1587_Bchr6_P18485	93.52	0.68	0.24	2.79	5.24E-03	7.94E-02	uncharacterized LOC103990231
Ma09_g01310	ITC1587_Bchr9_P25181	52.95	0.68	0.21	3.18	1.46E-03	3.95E-02	CBS domain-containing protein CBSCBSPB5-like
Ma10_g17530	ITC1587_Bchr6_P15816*	141.31	0.68	0.21	3.28	1.02E-03	3.20E-02	probable U6 snRNA-associated Sm-like protein LSm1
Ma04_g22620	ITC1587_Bchr1_P00260*	7943.92	0.68	0.22	3.04	2.40E-03	5.15E-02	ferredoxin--NADP reductase
Ma05_g02080	ITC1587_Bchr5_P11835	824.39	0.68	0.24	2.86	4.23E-03	7.11E-02	regulatory protein NPR3-like

Ma04_g27320	ITC1587_Bchr4_P10561	354.32	0.67	0.21	3.28	1.02E-03	3.19E-02	U-box domain-containing protein 52-like
Ma09_g04080	ITC1587_Bchr5_P14669*	60.43	0.67	0.22	3.04	2.37E-03	5.12E-02	uncharacterized LOC103996917
Ma05_g11500	ITC1587_Bchr5_P12708	123.45	0.67	0.19	3.58	3.44E-04	1.73E-02	probable protein phosphatase 2C 27
Ma07_g23840	ITC1587_Bchr7_P21003	211.37	0.67	0.21	3.14	1.72E-03	4.33E-02	Predicted protein
Ma01_g07230	ITC1587_Bchr1_P01211	1173.32	0.67	0.25	2.71	6.82E-03	9.24E-02	chaperone protein ClpB1
Ma09_g13990	ITC1587_Bchr9_P26277*	283.62	0.67	0.24	2.83	4.64E-03	7.50E-02	uncharacterized LOC103997935
Ma09_g24390	ITC1587_Bchr9_P27697	149.33	0.67	0.2	3.41	6.49E-04	2.47E-02	uncharacterized LOC103998807
Ma04_g18620	ITC1587_Bchr5_P12951*	247.34	0.67	0.2	3.31	9.33E-04	3.02E-02	arginase 1
Ma10_g02560	ITC1587_Bchr8_P23736*	114.39	0.67	0.19	3.44	5.75E-04	2.30E-02	signal recognition particle 19 kDa protein-like
Ma07_g20820	ITC1587_Bchr7_P20734	2383.06	0.67	0.21	3.1	1.92E-03	4.55E-02	NADP-dependent glyceraldehyde-3-phosphate dehydrogenase-like
Ma05_g14660	ITC1587_Bchr5_P12976	3448.97	0.67	0.23	2.89	3.82E-03	6.70E-02	translation factor GUF1 homolog
Ma01_g10850	ITC1587_Bchr1_P01540	445.43	0.66	0.23	2.93	3.36E-03	6.25E-02	cysteine protease ATG4B-like
Ma04_g19840	ITC1587_Bchr3_P07123	1826.32	0.66	0.21	3.22	1.28E-03	3.66E-02	peroxisomal membrane protein 11C-like
Ma10_g27400	ITC1587_Bchr10_P31307	336.96	0.66	0.25	2.69	7.17E-03	9.50E-02	mitogen-activated protein kinase kinase 2-like
Ma07_g24440	ITC1587_Bchr7_P21058	852.92	0.66	0.11	6.15	7.58E-10	5.05E-06	CBS domain-containing protein CBSCBSPB1-like
Ma10_g12850	ITC1587_Bchr10_P30042*	1192.29	0.66	0.18	3.68	2.33E-04	1.34E-02	F-box protein At2g32560-like
Ma04_g30260	ITC1587_Bchr7_P18560*	836.24	0.66	0.24	2.74	6.23E-03	8.74E-02	B3 domain-containing protein Os07g0563300-like
Ma05_g21330	ITC1587_Bchr5_P13760	6602.78	0.66	0.21	3.15	1.65E-03	4.22E-02	glycerate dehydrogenase
Ma11_g01370	ITC1587_Bchr11_P31813	2332.69	0.66	0.24	2.69	7.23E-03	9.53E-02	ferredoxin--NADP reductase
Ma10_g27080	ITC1587_Bchr10_P31270	266.36	0.66	0.19	3.39	6.90E-04	2.54E-02	uncharacterized LOC103969357
Ma01_g03060	ITC1587_Bchr10_P29616*	358.07	0.66	0.22	3.03	2.44E-03	5.20E-02	probable salt tolerance-like protein At1g78600
Ma10_g15170	ITC1587_Bchr10_P30255	1820.48	0.65	0.23	2.88	3.96E-03	6.85E-02	glutaredoxin domain-containing cysteine-rich protein CG12206
Ma08_g00160	ITC1587_Bchr8_P21484	151.72	0.65	0.24	2.68	7.46E-03	9.69E-02	probable indole-3-pyruvate monooxygenase YUCCA11
Ma04_g04050	ITC1587_Bchr4_P08747	172.65	0.65	0.23	2.87	4.05E-03	6.95E-02	peptidyl-prolyl cis-trans isomerase FKBP16-1
Ma11_g14000	ITC1587_Bchr11_P33382	99.45	0.65	0.18	3.52	4.40E-04	1.98E-02	E3 ubiquitin-protein ligase RNF4-like

Ma09_g02300	ITC1587_Bchr9_P25275	4119.04	0.65	0.21	3.06	2.20E-03	4.91E-02	salt tolerance protein
Ma02_g16160	ITC1587_Bchr2_P04361	282.72	0.65	0.22	2.97	2.93E-03	5.86E-02	ribonucleases P/MRP protein subunit POP1-like
Ma03_g02630	ITC1587_Bchr3_P05405*	1893.02	0.65	0.2	3.25	1.16E-03	3.45E-02	uncharacterized LOC103977086
Ma04_g14250	ITC1587_Bchr4_P09682	120.36	0.65	0.24	2.68	7.28E-03	9.57E-02	probable WRKY transcription factor 4
Ma02_g17510	ITC1587_Bchr2_P04481	415.16	0.64	0.22	2.92	3.49E-03	6.41E-02	putative GATA transcription factor 22
Ma06_g32870	ITC1587_Bchr6_P18012	356.63	0.64	0.17	3.76	1.71E-04	1.15E-02	putative oxidoreductase TDA3
Ma10_g30880	ITC1587_Bchr10_P31621	682.9	0.64	0.21	3.11	1.88E-03	4.53E-02	ribulose bisphosphate carboxylase/oxygenase activase
Ma06_g21850	ITC1587_Bchr6_P16669	280.29	0.64	0.18	3.59	3.28E-04	1.68E-02	uncharacterized LOC103988620
Ma06_g01340	ITC1587_Bchr11_P32705*	559.41	0.64	0.14	4.45	8.61E-06	2.00E-03	uncharacterized LOC103971095
Ma05_g03800	ITC1587_Bchr5_P12010	69.91	0.64	0.22	2.94	3.29E-03	6.20E-02	uncharacterized LOC103983884
Ma06_g25750	ITC1587_Bchr7_P18674*	239.61	0.64	0.2	3.26	1.11E-03	3.35E-02	serine/threonine-protein kinase HT1-like
Ma06_g31240	ITC1587_Bchr6_P17860	6786.53	0.64	0.24	2.66	7.85E-03	9.93E-02	glutamate--glyoxylate aminotransferase 2
Ma07_g17090	ITC1587_Bchr11_P33828*	348.39	0.64	0.15	4.26	2.01E-05	3.25E-03	uncharacterized LOC103992068
Ma02_g13200	ITC1587_Bchr2_P04109	341.72	0.64	0.22	2.97	2.98E-03	5.90E-02	amino acid permease 4-like
Ma09_g10620	ITC1587_Bchr8_P22910*	379.59	0.64	0.21	2.99	2.82E-03	5.71E-02	uncharacterized LOC103997597
Ma11_g17790	ITC1587_Bchr11_P33757	365.94	0.64	0.19	3.33	8.77E-04	2.93E-02	pheophytinase
Ma07_g20270	ITC1587_Bchr7_P20689	3234.36	0.64	0.21	3.02	2.51E-03	5.27E-02	zeaxanthin epoxidase
Ma03_g05720	ITC1587_Bchr3_P05677	707.67	0.64	0.23	2.79	5.23E-03	7.93E-02	putative ethylene-responsive transcription factor 1
Ma07_g22030	ITC1587_Bchr7_P20844	112.85	0.63	0.22	2.82	4.78E-03	7.58E-02	uncharacterized LOC103992377
Ma06_g30900	ITC1587_Bchr6_P17824	4953.34	0.63	0.23	2.81	5.00E-03	7.71E-02	uncharacterized aarF domain-containing protein kinase At4g31390
Ma08_g15730	ITC1587_Bchr8_P22821	506.09	0.63	0.22	2.93	3.36E-03	6.25E-02	pentatricopeptide repeat-containing protein At1g31920
Ma07_g28860	ITC1587_Bchr7_P21429	1048.55	0.63	0.17	3.74	1.85E-04	1.19E-02	probable receptor-like protein kinase At5g56460
Ma09_g00470	ITC1587_Bchr9_P25105*	497.32	0.63	0.15	4.08	4.48E-05	5.24E-03	uncharacterized LOC103996580
Ma08_g09290	ITC1587_Bchr8_P22275	263.25	0.62	0.14	4.59	4.43E-06	1.26E-03	uncharacterized LOC103993422
Ma04_g16670	ITC1587_BchrUn_random _P35009*	246.69	0.62	0.18	3.53	4.23E-04	1.94E-02	broad-range acid phosphatase DET1-like
Ma10_g00690	ITC1587_Bchr7_P19823*	118.95	0.62	0.22	2.77	5.52E-03	8.17E-02	monoglyceride lipase-like

Ma07_g09490	ITC1587_Bchr7_P19455	394.62	0.62	0.19	3.32	9.09E-04	2.99E-02	U-box domain-containing protein 34
Ma02_g12300	ITC1587_Bchr2_P04030	298.24	0.62	0.23	2.74	6.09E-03	8.61E-02	zeaxanthin epoxidase
Ma07_g19710	ITC1587_Bchr7_P20625	1925.69	0.62	0.22	2.81	4.90E-03	7.64E-02	uncharacterized LOC103992165
Ma06_g29830	ITC1587_Bchr6_P17730	255.53	0.62	0.2	3.15	1.63E-03	4.20E-02	protein FLX-like 4
Ma08_g15380	ITC1587_Bchr11_P31707*	764.32	0.62	0.2	3.06	2.25E-03	4.97E-02	uncharacterized LOC103994605
Ma11_g22630	ITC1587_Bchr11_P34189	429.31	0.62	0.23	2.68	7.37E-03	9.64E-02	putative DNA-binding protein ESCAROLA
Ma01_g06530	ITC1587_Bchr11_P33897*	1070.39	0.62	0.17	3.73	1.93E-04	1.22E-02	shaggy-related protein kinase alpha-like
Ma05_g13700	ITC1587_Bchr5_P12891	870.76	0.62	0.22	2.83	4.62E-03	7.49E-02	Probable aminotransferase ACS12
Ma11_g07660	ITC1587_Bchr11_P32434	561.51	0.62	0.21	2.95	3.14E-03	6.04E-02	RNA polymerase sigma factor sigA-like
Ma04_g30270	ITC1587_Bchr4_P10840*	264.7	0.62	0.14	4.3	1.73E-05	2.91E-03	sphingosine kinase 2
Ma02_g21890	ITC1587_Bchr2_P04880	1039.43	0.62	0.21	2.92	3.55E-03	6.46E-02	ABC transporter D family member 2
Ma09_g07860	ITC1587_Bchr9_P25734	133.14	0.62	0.16	3.87	1.11E-04	8.77E-03	MAP7 domain-containing protein 2-like
Ma04_g16310	ITC1587_Bchr4_P09953	82.05	0.61	0.23	2.73	6.37E-03	8.86E-02	regulation of nuclear pre-mRNA domain-containing protein 1B-like
Ma09_g14090	ITC1587_Bchr9_P26286	566.03	0.61	0.21	2.96	3.06E-03	5.96E-02	serine protease SPPA
Ma10_g06980	ITC1587_Bchr11_P32484*	828.08	0.61	0.15	4.2	2.67E-05	3.77E-03	zinc finger CCCH domain-containing protein ZFN-like
Ma04_g10140	ITC1587_Bchr4_P09286	946.69	0.61	0.22	2.75	5.90E-03	8.46E-02	zerumbone synthase
Ma06_g12050	ITC1587_Bchr10_P30564*	7065.11	0.61	0.21	2.88	3.92E-03	6.82E-02	ABC transporter F family member 5-like
Ma11_g17400	ITC1587_Bchr11_P33723	92.31	0.61	0.23	2.67	7.49E-03	9.71E-02	iron-sulfur assembly protein IscA-like 2
Ma03_g19340	ITC1587_Bchr3_P07151	342.47	0.61	0.18	3.38	7.30E-04	2.61E-02	methyltransferase-like protein 13
Ma08_g29730	ITC1587_Bchr8_P24595	551.17	0.61	0.18	3.46	5.32E-04	2.19E-02	uncharacterized LOC103995958
Ma08_g06880	ITC1587_Bchr8_P22051	40.36	0.61	0.22	2.77	5.52E-03	8.17E-02	uncharacterized LOC103993631
Ma03_g02670	ITC1587_Bchr10_P30335*	1495.33	0.6	0.19	3.12	1.79E-03	4.42E-02	transcriptional corepressor SEUSS-like
Ma07_g16830	ITC1587_BchrUn_random_P35868*	1312.07	0.6	0.22	2.75	6.01E-03	8.55E-02	probable receptor-like protein kinase At5g47070
Ma03_g01630	ITC1587_Bchr3_P05316	640.47	0.6	0.17	3.54	4.00E-04	1.90E-02	uncharacterized LOC103977179
Ma08_g23570	ITC1587_Bchr8_P24043*	1044.36	0.6	0.22	2.78	5.49E-03	8.17E-02	uncharacterized LOC103995265
Ma06_g23980	ITC1587_Bchr6_P16882*	174.79	0.6	0.22	2.68	7.31E-03	9.59E-02	uncharacterized LOC103988718
Ma11_g22670	ITC1587_Bchr11_P34192	425.02	0.6	0.17	3.45	5.66E-04	2.28E-02	PGR5-like protein 1B

Ma06_g25570	ITC1587_Bchr6_P17334	2804.39	0.6	0.16	3.68	2.34E-04	1.34E-02	potassium channel AKT2
Ma03_g09050	ITC1587_Bchr3_P06001	1671.97	0.6	0.14	4.3	1.69E-05	2.91E-03	cytochrome P450 2A2-like
Ma08_g34540	ITC1587_Bchr8_P25030	854.48	0.6	0.17	3.46	5.33E-04	2.19E-02	uncharacterized LOC103995540
Ma06_g32930	ITC1587_Bchr6_P18018	106.12	0.6	0.22	2.74	6.24E-03	8.74E-02	uncharacterized LOC103989560
Ma04_g30940	ITC1587_Bchr4_P10899	603.71	0.6	0.22	2.69	7.09E-03	9.46E-02	serine/threonine-protein kinase HT1-like
Ma01_g01800	ITC1587_Bchr1_P00594	84.63	0.6	0.21	2.82	4.85E-03	7.62E-02	protein REVEILLE 3-like
Ma08_g05820	ITC1587_Bchr8_P21955	384.19	0.6	0.22	2.69	7.09E-03	9.47E-02	uncharacterized LOC103993732
Ma08_g24630	ITC1587_Bchr8_P24135	347.48	0.6	0.19	3.11	1.87E-03	4.51E-02	TBC1 domain family member 5 homolog A-like
Ma10_g21760	ITC1587_Bchr10_P30810	577	0.59	0.22	2.66	7.87E-03	9.95E-02	plant-specific domain TIGR01589 family protein
Ma01_g09950	ITC1587_Bchr1_P01455	484.65	0.59	0.2	3.02	2.56E-03	5.33E-02	GDT1-like protein 1
Ma01_g18280	ITC1587_Bchr1_P02168	73.96	0.59	0.2	2.89	3.84E-03	6.72E-02	F-box/kelch-repeat protein At3g24760-like
Ma00_g00130	ITC1587_Bchr8_P24423*	77.89	0.59	0.17	3.41	6.60E-04	2.48E-02	reticulon-4-interacting protein 1
Ma01_g20090	ITC1587_Bchr1_P02359	344.82	0.59	0.17	3.39	7.09E-04	2.58E-02	homeobox protein knotted-1-like 13
Ma02_g19160	ITC1587_Bchr7_P20302*	382.02	-0.59	0.21	-2.79	5.29E-03	7.99E-02	syntaxin-132-like
Ma02_g07090	ITC1587_Bchr2_P03568*	84.09	-0.59	0.21	-2.74	6.21E-03	8.73E-02	uncharacterized LOC103972446
Ma11_g03930	ITC1587_Bchr11_P32065	372.06	-0.59	0.2	-2.96	3.11E-03	6.02E-02	clathrin light chain 2-like
Ma04_g01310	ITC1587_Bchr4_P08504	50.45	-0.59	0.21	-2.81	4.90E-03	7.64E-02	putative Transcription initiation factor IIB
Ma09_g12310	ITC1587_Bchr9_P26130	83.87	-0.59	0.2	-2.94	3.24E-03	6.16E-02	uncharacterized CRM domain-containing protein At3g25440
Ma03_g15200	ITC1587_Bchr3_P06717	198.58	-0.59	0.2	-2.93	3.42E-03	6.31E-02	F-box protein SKIP23-like
Ma02_g03230	ITC1587_Bchr10_P29824	425.48	-0.59	0.18	-3.24	1.20E-03	3.53E-02	glucose-6-phosphate/phosphate translocator 2
Ma06_g09840	ITC1587_Bchr6_P15516	80.49	-0.6	0.2	-2.99	2.76E-03	5.65E-02	uncharacterized protein At5g41620-like
Ma08_g33960	ITC1587_Bchr8_P24971	148.74	-0.6	0.18	-3.29	1.02E-03	3.18E-02	probable LRR receptor-like serine/threonine-protein kinase RKF3
Ma03_g30360	ITC1587_Bchr3_P08084	175.65	-0.6	0.2	-2.94	3.26E-03	6.18E-02	probable ribose-5-phosphate isomerase 2
Ma05_g30410	ITC1587_Bchr10_P30075*	223.4	-0.6	0.22	-2.76	5.83E-03	8.43E-02	40S ribosomal protein S7-like
Ma08_g02990	ITC1587_Bchr7_P18685*	345.7	-0.6	0.2	-2.99	2.83E-03	5.71E-02	40S ribosomal protein S18-like
Ma03_g27370	ITC1587_Bchr3_P07858	353.65	-0.6	0.23	-2.67	7.58E-03	9.75E-02	inositol-tetrakisphosphate 1-kinase 2-like

Ma03_g04840	ITC1587_Bchr3_P05598	115.44	-0.6	0.19	-3.15	1.63E-03	4.20E-02	mitogen-activated protein kinase kinase 5-like
Ma04_g27730	ITC1587_Bchr4_P10598	507.88	-0.6	0.21	-2.94	3.27E-03	6.19E-02	60S ribosomal protein L4-1-like
Ma04_g20650	ITC1587_Bchr1_P00099	180.68	-0.6	0.17	-3.45	5.52E-04	2.24E-02	60S ribosomal protein L21-1
Ma06_g26580	ITC1587_Bchr6_P17414	268.81	-0.61	0.17	-3.53	4.23E-04	1.94E-02	UDP-D-xylose:L-fucose alpha-1
Ma04_g22360	ITC1587_Bchr1_P00241*	183.98	-0.62	0.23	-2.69	7.08E-03	9.46E-02	probable 4-hydroxy-tetrahydrodipicolinate reductase 2
Ma10_g11200	ITC1587_Bchr10_P29909	201.73	-0.62	0.21	-3	2.67E-03	5.51E-02	RNA-binding protein 24-like
Ma02_g06960		202.84	-0.62	0.23	-2.7	6.98E-03	9.37E-02	Hypothetical protein
Ma08_g32210	ITC1587_Bchr1_P02303*	420.37	-0.62	0.21	-2.97	2.97E-03	5.89E-02	phosphoenolpyruvate/phosphate translocator 1
Ma06_g32850	ITC1587_Bchr6_P18010	329.87	-0.62	0.2	-3.1	1.91E-03	4.54E-02	60S ribosomal protein L15-1-like
Ma03_g32390	ITC1587_Bchr3_P08260	273.42	-0.63	0.2	-3.19	1.44E-03	3.91E-02	60S acidic ribosomal protein P2A-like
Ma10_g29730	ITC1587_BchrUn_random_P34647*	101.06	-0.63	0.19	-3.3	9.80E-04	3.12E-02	60S ribosomal protein L34-like
Ma11_g11720	ITC1587_Bchr11_P33134	80.04	-0.63	0.22	-2.81	4.94E-03	7.66E-02	uncharacterized LOC103971353
Ma05_g29810	ITC1587_Bchr5_P14586*	158.05	-0.63	0.21	-2.95	3.20E-03	6.12E-02	gibberellin receptor GID1C-like
Ma10_g02940	ITC1587_Bchr10_P29020	250.23	-0.63	0.21	-2.97	3.02E-03	5.94E-02	protein arginine N-methyltransferase 5
Ma10_g12590	ITC1587_Bchr10_P30024	245.35	-0.63	0.23	-2.77	5.57E-03	8.20E-02	acetyl-coenzyme A synthetase
Ma06_g04550	ITC1587_Bchr6_P18493*	254.5	-0.64	0.2	-3.22	1.28E-03	3.65E-02	pyrophosphate-energized vacuolar membrane proton pump-like
Ma10_g26720	ITC1587_Bchr2_P04512*	89.7	-0.64	0.23	-2.79	5.31E-03	8.01E-02	60S ribosomal protein L35-like
Ma06_g32440	ITC1587_BchrUn_random_P37970*	579.8	-0.64	0.19	-3.42	6.34E-04	2.44E-02	Alpha-1, 4-glucan-protein synthase [UDP-forming] 2
Ma08_g13450	ITC1587_Bchr8_P22699	77.73	-0.65	0.24	-2.74	6.08E-03	8.61E-02	uncharacterized LOC103994479
Ma09_g29770	ITC1587_Bchr9_P28191	144.16	-0.65	0.17	-3.91	9.17E-05	7.94E-03	uncharacterized LOC103999345
Ma03_g24770	ITC1587_Bchr5_P14516*	893.86	-0.65	0.2	-3.28	1.05E-03	3.23E-02	Tubulin beta-1 chain
Ma09_g30080	ITC1587_Bchr9_P28221*	69.21	-0.65	0.23	-2.79	5.19E-03	7.90E-02	pathogenesis-related protein 5-like
Ma05_g13940	ITC1587_Bchr5_P12916	39.94	-0.65	0.23	-2.82	4.78E-03	7.58E-02	DNA repair protein RAD51 homolog 3
Ma10_g11950	ITC1587_Bchr10_P29975	201.21	-0.65	0.23	-2.78	5.51E-03	8.17E-02	non-specific phospholipase C6-like
Ma10_g07470	ITC1587_Bchr10_P29510	425.59	-0.65	0.21	-3.06	2.24E-03	4.97E-02	peroxisome proliferator-activated receptor gamma coactivator-related protein 1-like

Ma11_g07290	ITC1587_Bchr11_P32401	56.77	-0.65	0.24	-2.74	6.05E-03	8.58E-02	anthocyanidin 3-O-glucoside 6''-O-acyltransferase-like
Ma09_g00190	ITC1587_Bchr9_P25083*	85.88	-0.66	0.2	-3.36	7.71E-04	2.70E-02	60S ribosomal protein L37a
Ma06_g34090	ITC1587_Bchr6_P18128	135.27	-0.66	0.22	-2.99	2.78E-03	5.67E-02	transmembrane protein 87B-like
Ma02_g01500	ITC1587_BchrUn_random_P37400	195.78	-0.66	0.25	-2.68	7.41E-03	9.66E-02	probable 60S ribosomal protein L14
Ma04_g38190	ITC1587_Bchr4_P11506	193.38	-0.66	0.21	-3.11	1.88E-03	4.52E-02	homeobox-leucine zipper protein HOX32-like
Ma03_g09900	ITC1587_Bchr3_P06080	867.73	-0.66	0.24	-2.78	5.46E-03	8.15E-02	Ethylene-responsive transcription factor 1
Ma08_g27330	ITC1587_Bchr8_P24384	87.78	-0.66	0.2	-3.35	8.11E-04	2.80E-02	uncharacterized LOC103996176
Ma10_g29790	ITC1587_Bchr10_P31519	155.12	-0.66	0.14	-4.67	3.06E-06	1.03E-03	probable galacturonosyltransferase 9
Ma10_g17390	ITC1587_Bchr10_P30448	1178.65	-0.67	0.2	-3.32	9.10E-04	2.99E-02	calnexin homolog
Ma07_g26860	ITC1587_Bchr7_P21273	135.58	-0.67	0.25	-2.69	7.15E-03	9.49E-02	Hydroxyacylglutathione hydrolase 3
Ma05_g04190	ITC1587_Bchr5_P12049	790.89	-0.67	0.2	-3.35	8.03E-04	2.79E-02	transmembrane 9 superfamily member 4-like
Ma06_g30150	ITC1587_Bchr6_P17760	197.01	-0.67	0.22	-3.05	2.31E-03	5.05E-02	probable inactive receptor kinase At1g27190
Ma11_g14090	ITC1587_Bchr7_P21022*	302.1	-0.67	0.22	-3.12	1.81E-03	4.44E-02	40S ribosomal protein S27-2-like
Ma08_g02060	ITC1587_Bchr8_P21662	166.99	-0.68	0.21	-3.2	1.36E-03	3.79E-02	L-type lectin-domain containing receptor kinase VIII.1-like
Ma04_g22130	ITC1587_Bchr5_P12653*	85.13	-0.68	0.2	-3.37	7.49E-04	2.67E-02	expressed protein
Ma06_g16940	ITC1587_Bchr6_P16156	110.09	-0.68	0.24	-2.87	4.11E-03	7.01E-02	UDP-glucuronic acid decarboxylase 1-like
Ma10_g15600	ITC1587_Bchr10_P30290	181.71	-0.68	0.22	-3.04	2.35E-03	5.10E-02	putative glycosyltransferase 5
Ma07_g04850	ITC1587_Bchr9_P28180*	157.65	-0.68	0.18	-3.86	1.13E-04	8.85E-03	putative phagocytic receptor 1b
Ma11_g11830	ITC1587_Bchr11_P33114	368.52	-0.69	0.26	-2.66	7.76E-03	9.85E-02	uncharacterized LOC103971361
Ma09_g07540	ITC1587_Bchr9_P25711	126.3	-0.69	0.22	-3.13	1.74E-03	4.36E-02	zinc finger AN1 domain-containing stress-associated protein 15-like
Ma08_g33990	ITC1587_Bchr9_P28331*	115.82	-0.69	0.24	-2.88	3.94E-03	6.82E-02	acyl carrier protein 1
Ma05_g13270	ITC1587_Bchr5_P12851	194.49	-0.69	0.25	-2.82	4.83E-03	7.60E-02	delta(24)-sterol reductase-like
Ma05_g22470	ITC1587_Bchr5_P13878	143.92	-0.69	0.2	-3.41	6.56E-04	2.48E-02	probable methyltransferase PMT13
Ma07_g10640	ITC1587_Bchr6_P15291*	71.4	-0.69	0.26	-2.68	7.41E-03	9.66E-02	60S ribosomal protein L37-3-like
Ma03_g19230	ITC1587_Bchr3_P07143	646.2	-0.69	0.22	-3.17	1.51E-03	4.02E-02	calcium/calmodulin-dependent serine/threonine-protein kinase 1-like

Ma10_g25130	ITC1587_Bchr10_P31111	499.16	-0.7	0.17	-4.21	2.54E-05	3.66E-03	mitochondrial phosphate carrier protein 3
Ma02_g15720	ITC1587_Bchr2_P04317	231.33	-0.7	0.17	-4.14	3.45E-05	4.46E-03	phytosulfokine receptor 2
Ma03_g18060	ITC1587_Bchr2_P03625*	116.19	-0.7	0.24	-2.98	2.93E-03	5.85E-02	transcription factor bHLH113-like
Ma04_g15020	ITC1587_Bchr4_P09760	44.42	-0.7	0.25	-2.84	4.52E-03	7.37E-02	high mobility group B protein 14-like
Ma10_g04640	ITC1587_Bchr10_P29325	233.08	-0.7	0.25	-2.82	4.77E-03	7.58E-02	histone deacetylase HDT2-like
Ma10_g13880	ITC1587_Bchr3_P07073*	120.68	-0.71	0.26	-2.77	5.57E-03	8.21E-02	uncharacterized protein At3g49720-like
Ma10_g17870	ITC1587_Bchr6_P15793*	333.78	-0.71	0.19	-3.78	1.55E-04	1.08E-02	uncharacterized LOC103968538
Ma08_g06670	ITC1587_Bchr8_P22030	141.14	-0.71	0.26	-2.77	5.63E-03	8.24E-02	broad-range acid phosphatase DET1-like
Ma10_g03610	ITC1587_Bchr10_P29484	413.98	-0.71	0.2	-3.49	4.86E-04	2.08E-02	transmembrane 9 superfamily member 4-like
Ma02_g03590	ITC1587_Bchr3_P07664*	336.27	-0.72	0.19	-3.7	2.13E-04	1.28E-02	40S ribosomal protein S4-like
Ma05_g02670	ITC1587_Bchr4_P10631*	2138.71	-0.72	0.24	-3.03	2.42E-03	5.17E-02	5-methyltetrahydropteroyltriglutamate--homocysteine methyltransferase 2-like
Ma03_g33550	ITC1587_Bchr10_P30678*	51.92	-0.72	0.25	-2.9	3.72E-03	6.59E-02	NO-associated protein 1
Ma10_g14310	ITC1587_Bchr10_P30172	59.58	-0.72	0.25	-2.84	4.48E-03	7.32E-02	60S ribosomal protein L34-like
Ma04_g36390	ITC1587_Bchr4_P11365	105.73	-0.72	0.22	-3.32	9.11E-04	2.99E-02	SufE-like protein
Ma01_g15110	ITC1587_Bchr1_P01914	2215.62	-0.72	0.16	-4.58	4.64E-06	1.28E-03	cystathionine gamma-synthase
Ma08_g17270	ITC1587_BchrUn_random_P36900	1157.25	-0.72	0.19	-3.74	1.84E-04	1.19E-02	inositol-3-phosphate synthase
Ma03_g26960	ITC1587_Bchr3_P07815	340.29	-0.72	0.19	-3.75	1.79E-04	1.18E-02	serine carboxypeptidase-like 2
Ma10_g09480	ITC1587_Bchr10_P29688	76.91	-0.73	0.26	-2.77	5.67E-03	8.27E-02	U3 small nucleolar RNA-interacting protein 2-like
Ma11_g23360	ITC1587_Bchr11_P34245	571.31	-0.73	0.25	-2.96	3.08E-03	5.99E-02	zinc finger CCCH domain-containing protein 53-like
Ma11_g08120	ITC1587_Bchr11_P32475	43.29	-0.73	0.27	-2.7	6.85E-03	9.26E-02	uncharacterized LOC103970715
Ma07_g11270	ITC1587_Bchr7_P19596	89.26	-0.73	0.2	-3.63	2.81E-04	1.51E-02	F-box only protein 13-like
Ma10_g16940	ITC1587_Bchr6_P15375*	96.25	-0.73	0.18	-3.98	6.76E-05	6.75E-03	copper amine oxidase 1-like
Ma02_g17970	ITC1587_Bchr10_P30113*	213.89	-0.74	0.19	-3.84	1.24E-04	9.39E-03	actin-depolymerizing factor 7
Ma04_g40030	ITC1587_Bchr4_P11652	71.83	-0.74	0.26	-2.88	3.93E-03	6.82E-02	transmembrane protein 97-like
Ma04_g36960	ITC1587_Bchr4_P11409	43.63	-0.74	0.27	-2.78	5.48E-03	8.16E-02	F-box/LRR-repeat protein At3g48880-like
Ma07_g15170	ITC1587_Bchr7_P19973	126.9	-0.74	0.26	-2.85	4.34E-03	7.21E-02	coatomer subunit zeta-1-like
Ma04_g05510	ITC1587_Bchr4_P08876	214.52	-0.74	0.26	-2.86	4.23E-03	7.11E-02	putative dihydroxy-acid dehydratase

Ma10_g18160	ITC1587_Bchr10_P30511	155.94	-0.74	0.24	-3.12	1.80E-03	4.43E-02	mannose-1-phosphate guanyltransferase alpha-like
Ma03_g14150	ITC1587_BchrUn_random_P35656	29.13	-0.75	0.26	-2.88	4.02E-03	6.92E-02	lysM domain receptor-like kinase 3
Ma05_g29830	ITC1587_Bchr5_P14586	144.54	-0.75	0.25	-2.96	3.04E-03	5.95E-02	gibberellin receptor GID1C-like
Ma11_g24850	ITC1587_Bchr8_P22179*	41.98	-0.75	0.26	-2.84	4.47E-03	7.32E-02	uncharacterized LOC103972491
Ma11_g12160	ITC1587_Bchr3_P07139*	266.93	-0.75	0.19	-3.88	1.04E-04	8.56E-03	ADP-ribosylation factor 1
Ma10_g04890	ITC1587_Bchr10_P29355	350.37	-0.76	0.22	-3.38	7.16E-04	2.60E-02	zinc finger CCCH domain-containing protein 53-like
Ma09_g00980	ITC1587_Bchr9_P25149*	58.64	-0.76	0.27	-2.83	4.63E-03	7.50E-02	probable carbohydrate esterase At4g34215
Ma08_g16380	ITC1587_Bchr8_P23257	728.37	-0.76	0.28	-2.75	6.05E-03	8.58E-02	adenosylhomocysteinase-like
Ma03_g13260	ITC1587_BchrUn_random_P37677	211.07	-0.76	0.28	-2.73	6.29E-03	8.78E-02	chaperone protein ClpD1
Ma08_g04690	ITC1587_Bchr8_P21863	332.22	-0.77	0.28	-2.77	5.63E-03	8.24E-02	proline-rich receptor-like protein kinase PERK8
Ma01_g20900	ITC1587_Bchr1_P02594	156.58	-0.77	0.24	-3.19	1.40E-03	3.87E-02	GDP-mannose transporter GONST3-like
Ma04_g38400	ITC1587_Bchr11_P34301*	108.23	-0.77	0.29	-2.69	7.14E-03	9.49E-02	probable UDP-arabinose 4-epimerase 1
Ma01_g16380	ITC1587_Bchr1_P01145*	99.19	-0.77	0.24	-3.16	1.55E-03	4.09E-02	40S ribosomal protein S2-2-like
Ma11_g03020	ITC1587_Bchr11_P31973	207.06	-0.78	0.26	-2.96	3.08E-03	5.98E-02	transcription factor PCF2-like
Ma08_g31710	ITC1587_Bchr8_P24786	38.77	-0.78	0.26	-3.02	2.55E-03	5.32E-02	origin recognition complex subunit 1-like
Ma03_g12450	ITC1587_Bchr3_P06301	42.23	-0.78	0.25	-3.1	1.93E-03	4.57E-02	protein FAR1-RELATED SEQUENCE 5-like
Ma03_g03160	ITC1587_Bchr3_P05448	1074.68	-0.78	0.25	-3.17	1.52E-03	4.05E-02	plasma membrane-associated cation-binding protein 1-like
Ma05_g00330	ITC1587_Bchr5_P11695	453.37	-0.78	0.29	-2.71	6.80E-03	9.24E-02	MAPK-interacting and spindle-stabilizing protein-like
Ma06_g11370	ITC1587_Bchr6_P15658	56.23	-0.79	0.28	-2.78	5.41E-03	8.09E-02	60S ribosomal protein L26-2-like
Ma10_g21360	ITC1587_Bchr10_P30776	98.92	-0.79	0.28	-2.83	4.60E-03	7.47E-02	cell wall protein IFF6-like
Ma10_g02450	ITC1587_Bchr10_P28387*	33.12	-0.79	0.26	-2.99	2.79E-03	5.67E-02	ASC1-like protein 3
Ma03_g00670	ITC1587_Bchr3_P05227	574.07	-0.79	0.29	-2.78	5.51E-03	8.17E-02	REF/SRPP-like protein OsI_017815
Ma06_g29810	ITC1587_Bchr6_P17728	191.33	-0.79	0.24	-3.27	1.08E-03	3.30E-02	beta-1, 4-mannosyl-glycoprotein 4-beta-N-acetylglucosaminyltransferase-like
Ma02_g02280	ITC1587_BchrUn_random_P34551	139.42	-0.79	0.26	-3.08	2.09E-03	4.77E-02	probable glucan endo-1, 3-beta-glucosidase A6

Ma04_g13740	ITC1587_Bchr4_P09626*	143.34	-0.79	0.26	-3.08	2.08E-03	4.75E-02	probable serine/threonine-protein kinase At1g18390
Ma11_g07710	ITC1587_Bchr4_P09522*	63	-0.79	0.3	-2.69	7.18E-03	9.51E-02	uncharacterized LOC103970681
Ma07_g23820	ITC1587_Bchr7_P21001	746.24	-0.8	0.27	-2.93	3.37E-03	6.25E-02	exocyst complex component EXO70B1-like
Ma07_g00690	ITC1587_Bchr7_P18608*	34.58	-0.8	0.3	-2.7	6.95E-03	9.35E-02	glycine, alanine and asparagine-rich protein-like
Ma01_g09850	ITC1587_Bchr1_P01445	76.28	-0.8	0.19	-4.21	2.51E-05	3.66E-03	multiple C2 and transmembrane domain-containing protein 1-like
Ma08_g34120	ITC1587_Bchr8_P24984	186.21	-0.8	0.2	-4	6.33E-05	6.55E-03	60S ribosomal protein L22-2-like
Ma07_g19850	ITC1587_Bchr7_P20643	102.13	-0.8	0.25	-3.18	1.46E-03	3.95E-02	protein trichome birefringence-like 16
Ma07_g16150	ITC1587_Bchr7_P20115	157.31	-0.8	0.27	-2.93	3.43E-03	6.31E-02	UDP-glucuronate 4-epimerase 1-like
Ma05_g30340	ITC1587_Bchr5_P14635	259.38	-0.81	0.17	-4.79	1.63E-06	7.39E-04	uncharacterized LOC103986200
Ma05_g14730	ITC1587_Bchr4_P10186	199.21	-0.81	0.27	-3.05	2.29E-03	5.03E-02	uncharacterized LOC103984972
Ma06_g07790	ITC1587_Bchr6_P15328	59.32	-0.81	0.3	-2.71	6.72E-03	9.17E-02	probable 3-beta-hydroxysteroid-Delta(8)
Ma04_g02590	ITC1587_Bchr4_P08626	1102.82	-0.81	0.26	-3.16	1.59E-03	4.14E-02	probable rhamnose biosynthetic enzyme 1
Ma08_g08720	ITC1587_Bchr8_P22225	95.12	-0.81	0.29	-2.79	5.25E-03	7.95E-02	uncharacterized LOC103993468
Ma08_g33620	ITC1587_Bchr8_P22055*	63.31	-0.81	0.22	-3.72	2.03E-04	1.26E-02	putative serine/threonine-protein kinase {ECO:0000250|UniProtKB:Q9FE20
Ma08_g02680	ITC1587_Bchr8_P21713	36.69	-0.82	0.3	-2.77	5.61E-03	8.24E-02	allene oxide synthase 1
Ma09_g05250	ITC1587_Bchr9_P25519	459.17	-0.82	0.29	-2.86	4.19E-03	7.07E-02	calcium-dependent protein kinase 3-like
Ma05_g15890		53.72	-0.82	0.21	-3.96	7.45E-05	7.04E-03	transcription initiation factor TFIID subunit 13
Ma07_g15360	ITC1587_Bchr7_P19990	493.08	-0.82	0.26	-3.18	1.46E-03	3.95E-02	L-ascorbate peroxidase
Ma10_g24940	ITC1587_Bchr10_P31090	266.13	-0.82	0.19	-4.44	8.92E-06	2.05E-03	glucose-6-phosphate isomerase 1
Ma03_g14510	ITC1587_Bchr3_P06402	48.46	-0.82	0.28	-2.93	3.36E-03	6.25E-02	MLO-like protein 13
Ma09_g31040	ITC1587_Bchr9_P28299	80.66	-0.83	0.31	-2.7	7.00E-03	9.38E-02	F-box/kelch-repeat protein At5g60570-like
Ma03_g26890	ITC1587_Bchr3_P07810	365.86	-0.83	0.3	-2.79	5.29E-03	7.99E-02	allene oxide synthase 2-like
Ma02_g19580	ITC1587_Bchr2_P04671	201.87	-0.83	0.31	-2.67	7.68E-03	9.83E-02	BAG family molecular chaperone regulator 7-like
Ma09_g29410	ITC1587_Bchr11_P32644*	169.7	-0.83	0.31	-2.71	6.71E-03	9.16E-02	mitochondrial dicarboxylate/tricarboxylate transporter DTC-like
Ma03_g01580	ITC1587_Bchr3_P05308	76.61	-0.83	0.31	-2.66	7.91E-03	9.98E-02	ras-related protein Rab11D-like

Ma06_g08260	ITC1587_Bchr6_P15375	671.05	-0.84	0.28	-3.02	2.55E-03	5.32E-02	copper amine oxidase 1-like
Ma06_g06710	ITC1587_Bchr9_P28124*	776.82	-0.84	0.27	-3.16	1.56E-03	4.09E-02	UDP-glucuronic acid decarboxylase 6-like
Ma05_g13550	ITC1587_Bchr5_P12874	359.41	-0.85	0.22	-3.86	1.15E-04	8.89E-03	GATA transcription factor 6-like
Ma07_g23470	ITC1587_Bchr7_P20967	38.29	-0.85	0.32	-2.67	7.60E-03	9.77E-02	Predicted protein
Ma10_g30380	ITC1587_Bchr10_P31576	1025.41	-0.85	0.16	-5.41	6.47E-08	9.08E-05	Putative DRF-like transcription factor DRFL2a
Ma08_g28570	ITC1587_Bchr8_P24495	66.48	-0.85	0.29	-2.89	3.87E-03	6.77E-02	probable protein phosphatase 2C 7
Ma01_g15260	ITC1587_Bchr1_P01923	124.37	-0.85	0.31	-2.78	5.42E-03	8.10E-02	putative protein TIFY 10B
Ma01_g23590	ITC1587_Bchr9_P26965	129.53	-0.85	0.2	-4.17	3.11E-05	4.16E-03	eukaryotic translation initiation factor 3 subunit J {ECO:0000255|HAMAP-Rule:MF_03009}-like
Ma11_g01930	ITC1587_Bchr11_P31866	110.42	-0.85	0.24	-3.53	4.22E-04	1.94E-02	probable acetyltransferase NATA1-like
Ma08_g19590	ITC1587_Bchr8_P22968	870.55	-0.85	0.28	-3.01	2.63E-03	5.46E-02	Hypothetical protein
Ma01_g15280	ITC1587_BchrUn_random_P37575*	46.6	-0.86	0.3	-2.84	4.47E-03	7.32E-02	uncharacterized LOC103991192
Ma03_g02050	ITC1587_Bchr3_P05351	158.94	-0.86	0.31	-2.77	5.66E-03	8.27E-02	probable inactive receptor kinase At1g48480
Ma03_g31050	ITC1587_Bchr3_P08147*	89.86	-0.86	0.31	-2.73	6.41E-03	8.88E-02	U-box domain-containing protein 4-like
Ma03_g22740	ITC1587_Bchr3_P07463	104.39	-0.86	0.25	-3.5	4.57E-04	2.02E-02	uncharacterized LOC103979164
Ma01_g05010	ITC1587_Bchr1_P01016	71.9	-0.86	0.24	-3.62	2.91E-04	1.54E-02	hexokinase-3-like
Ma02_g18100	ITC1587_Bchr2_P04532	43.93	-0.87	0.31	-2.8	5.11E-03	7.81E-02	probable receptor-like protein kinase At5g47070
Ma02_g17950	ITC1587_Bchr2_P04518	187.87	-0.87	0.32	-2.72	6.44E-03	8.91E-02	myb-related protein MYBAS2
Ma08_g00080	ITC1587_Bchr8_P21474	83.63	-0.87	0.31	-2.78	5.41E-03	8.09E-02	transcription factor PCF2-like
Ma02_g04620	ITC1587_Bchr2_P03330	80.01	-0.87	0.23	-3.75	1.77E-04	1.18E-02	GDP-mannose 4
Ma10_g04060	ITC1587_Bchr10_P29264	60.03	-0.87	0.28	-3.11	1.88E-03	4.52E-02	60S ribosomal protein L38-like
Ma04_g37560	ITC1587_Bchr4_P11458	31.83	-0.87	0.32	-2.75	5.88E-03	8.46E-02	protein BZR1 homolog 2-like
Ma04_g35500		24.3	-0.88	0.32	-2.77	5.55E-03	8.19E-02	uncharacterized LOC103982446
Ma06_g27910	ITC1587_Bchr6_P17550	156.95	-0.88	0.27	-3.22	1.28E-03	3.66E-02	serine/threonine-protein kinase HT1-like
Ma01_g06520	ITC1587_Bchr1_P01149	1829.07	-0.88	0.33	-2.69	7.17E-03	9.50E-02	S-adenosylmethionine synthase 1
Ma03_g10940	ITC1587_Bchr3_P06168	105.83	-0.88	0.3	-2.91	3.57E-03	6.47E-02	uncharacterized LOC103977903
Ma09_g02880	ITC1587_Bchr9_P25328	51.33	-0.89	0.33	-2.67	7.61E-03	9.77E-02	uncharacterized LOC103996804

Ma09_g27010	ITC1587_Bchr9_P27945	148.66	-0.89	0.3	-2.93	3.42E-03	6.31E-02	BTB/POZ domain-containing protein At5g66560-like
Ma10_g07130	ITC1587_Bchr10_P29454	67.14	-0.89	0.32	-2.8	5.06E-03	7.76E-02	putative serine/threonine-protein kinase {ECO:0000250|UniProtKB:Q9FE20
Ma06_g09320	ITC1587_Bchr6_P15472	31.35	-0.89	0.33	-2.72	6.61E-03	9.08E-02	basic blue protein-like
Ma04_g01610	ITC1587_Bchr4_P08532	197.06	-0.89	0.28	-3.17	1.54E-03	4.09E-02	probable galactinol--sucrose galactosyltransferase 1
Ma02_g22350	ITC1587_Bchr6_P15936*	92.66	-0.9	0.33	-2.72	6.61E-03	9.08E-02	protein MKS1-like
Ma05_g14540	ITC1587_Bchr5_P12964	109.16	-0.9	0.25	-3.53	4.18E-04	1.93E-02	auxin response factor 18
Ma04_g02300	ITC1587_Bchr4_P08599	62.08	-0.9	0.25	-3.6	3.20E-04	1.64E-02	exocyst complex component EXO70A1-like
Ma03_g04420	ITC1587_Bchr3_P05554	89.33	-0.9	0.29	-3.07	2.14E-03	4.85E-02	uncharacterized LOC103976931
Ma06_g33040	ITC1587_Bchr6_P18028	140.16	-0.9	0.26	-3.51	4.54E-04	2.02E-02	NAC domain-containing protein 21/22
Ma11_g10700	ITC1587_Bchr3_P05930*	38.46	-0.91	0.29	-3.09	1.99E-03	4.67E-02	ubiquitin-40S ribosomal protein S27a-1-like
Ma05_g03140	ITC1587_Bchr5_P11943*	50.61	-0.91	0.28	-3.23	1.23E-03	3.59E-02	F-box protein PP2-A13-like
Ma06_g03670	ITC1587_Bchr6_P14950	56.19	-0.91	0.31	-2.94	3.29E-03	6.20E-02	ubiquitin carboxyl-terminal hydrolase 36-like
Ma11_g15360	ITC1587_Bchr11_P34028*	283.76	-0.91	0.32	-2.8	5.18E-03	7.89E-02	Niemann-Pick C1 protein-like
Ma02_g13530	ITC1587_Bchr2_P04133	69.56	-0.91	0.34	-2.68	7.33E-03	9.60E-02	uncharacterized LOC103975783
Ma03_g31530	ITC1587_Bchr3_P08190	1648.07	-0.91	0.3	-3.02	2.52E-03	5.29E-02	UDP-glucose 6-dehydrogenase 4
Ma03_g12530	ITC1587_Bchr3_P06310	663.95	-0.91	0.27	-3.34	8.36E-04	2.85E-02	S-adenosylmethionine synthase
Ma04_g08910	ITC1587_Bchr4_P09173	810.17	-0.92	0.28	-3.31	9.19E-04	2.99E-02	systemin receptor SR160-like
Ma10_g21340	ITC1587_Bchr10_P30774	197.66	-0.92	0.28	-3.28	1.06E-03	3.25E-02	NADP-dependent malic enzyme
Ma09_g27000	ITC1587_Bchr9_P27944	311.16	-0.93	0.3	-3.07	2.17E-03	4.87E-02	cold-regulated 413 plasma membrane protein 2-like
Ma06_g22600	ITC1587_Bchr7_P19335*	192.5	-0.93	0.33	-2.82	4.81E-03	7.60E-02	kinesin-4-like
Ma05_g17000	ITC1587_BchrUn_random_P35437	100.11	-0.93	0.35	-2.69	7.07E-03	9.46E-02	E3 ubiquitin-protein ligase RMA1H1-like
Ma08_g02110	ITC1587_Bchr8_P21667	432.15	-0.93	0.25	-3.78	1.55E-04	1.08E-02	auxin transporter-like protein 4
Ma03_g32570	ITC1587_Bchr3_P08276	147.52	-0.93	0.34	-2.75	5.94E-03	8.51E-02	receptor-like protein kinase HSL1
Ma10_g25700	ITC1587_Bchr10_P31151	80.36	-0.94	0.29	-3.21	1.31E-03	3.70E-02	thioredoxin-like 1-2
Ma03_g14070	ITC1587_BchrUn_random_P35664	126.42	-0.94	0.29	-3.26	1.13E-03	3.38E-02	cellulose synthase-like protein D2

Ma06_g01470	ITC1587_Bchr11_P32719	1197.66	-0.94	0.23	-4.13	3.60E-05	4.62E-03	glyceraldehyde-3-phosphate dehydrogenase 2
Ma06_g15660	ITC1587_Bchr6_P16045	110.31	-0.94	0.33	-2.81	4.94E-03	7.66E-02	F-box/kelch-repeat protein At3g61590-like
Ma10_g06360	ITC1587_Bchr2_P03602*	31.5	-0.95	0.31	-3.05	2.30E-03	5.05E-02	sodium/hydrogen exchanger 6-like
Ma05_g07520	ITC1587_Bchr5_P12358	367.99	-0.95	0.35	-2.71	6.79E-03	9.22E-02	uncharacterized LOC103984209
Ma07_g28090	ITC1587_Bchr7_P21373*	238.01	-0.95	0.33	-2.88	3.99E-03	6.88E-02	UDP-glucose 6-dehydrogenase 1-like
Ma10_g25440	ITC1587_Bchr10_P31130	82.01	-0.95	0.3	-3.14	1.70E-03	4.31E-02	plastidic glucose transporter 4-like
Ma07_g10110	ITC1587_Bchr7_P19502	34.42	-0.95	0.35	-2.76	5.75E-03	8.34E-02	HMG-Y-related protein A-like
Ma09_g24000	ITC1587_Bchr9_P27648	351.44	-0.96	0.34	-2.85	4.41E-03	7.27E-02	probable voltage-gated potassium channel subunit beta
Ma03_g02140		56.71	-0.96	0.25	-3.81	1.39E-04	1.01E-02	protein Asterix-like
Ma05_g00610	ITC1587_Bchr5_P11712	161.63	-0.96	0.36	-2.68	7.44E-03	9.68E-02	aminomethyltransferase
Ma06_g27740	ITC1587_Bchr6_P17536	123.92	-0.96	0.33	-2.93	3.42E-03	6.31E-02	probable LRR receptor-like serine/threonine-protein kinase At1g53440
Ma10_g10660	ITC1587_Bchr10_P29796	384.46	-0.96	0.31	-3.07	2.17E-03	4.86E-02	serine/threonine-protein kinase SAPK7-like
Ma09_g10900	ITC1587_Bchr9_P26005	259.38	-0.96	0.22	-4.45	8.41E-06	1.98E-03	uncharacterized LOC103997625
Ma09_g04940	ITC1587_Bchr9_P25493	218.6	-0.96	0.28	-3.41	6.53E-04	2.47E-02	phospholipid-transporting ATPase 1-like
Ma03_g27330	ITC1587_Bchr3_P07852	167.09	-0.96	0.3	-3.17	1.50E-03	4.02E-02	probable LRR receptor-like serine/threonine-protein kinase At2g16250
Ma06_g16240	ITC1587_Bchr6_P16101	323.29	-0.96	0.33	-2.94	3.30E-03	6.20E-02	aquaporin NIP1-1-like
Ma05_g27300	ITC1587_Bchr5_P14358	200.03	-0.96	0.35	-2.74	6.24E-03	8.74E-02	Peroxidase 55
Ma04_g27130	ITC1587_Bchr4_P10542	69.15	-0.97	0.34	-2.83	4.65E-03	7.50E-02	probable GDP-L-fucose synthase 1
Ma01_g18500	ITC1587_Bchr1_P02144	224.17	-0.98	0.35	-2.82	4.78E-03	7.58E-02	cellulose synthase-like protein D2
Ma01_g21460	ITC1587_Bchr4_P09328*	26.03	-0.98	0.33	-2.94	3.28E-03	6.19E-02	uncharacterized LOC103999823
Ma06_g21940	ITC1587_BchrUn_random_P38089	123.02	-0.98	0.27	-3.56	3.74E-04	1.82E-02	nuclear transport factor 2-like
Ma04_g30110	ITC1587_Bchr4_P10823	41.97	-0.98	0.36	-2.73	6.28E-03	8.78E-02	putative leucine-rich repeat receptor-like serine/threonine-protein kinase At2g14440
Ma03_g31040	ITC1587_Bchr3_P08146	304.42	-0.98	0.29	-3.41	6.48E-04	2.47E-02	cysteine proteinase inhibitor 1 {ECO:0000303|PubMed:14697268}-like
Ma04_g37920	ITC1587_Bchr4_P11483	48.05	-0.98	0.32	-3.03	2.44E-03	5.20E-02	uncharacterized protein At2g39795
Ma09_g04790	ITC1587_Bchr9_P25483	1020.69	-0.99	0.36	-2.72	6.57E-03	9.05E-02	receptor-like protein kinase FERONIA

Ma01_g09940	ITC1587_BchrUn_random_P35537*	64.85	-0.99	0.27	-3.67	2.47E-04	1.38E-02	40S ribosomal protein S13
Ma02_g06760	ITC1587_Bchr2_P03541	24.86	-0.99	0.3	-3.33	8.54E-04	2.89E-02	pentatricopeptide repeat-containing protein At3g12770
Ma02_g03350	ITC1587_Bchr11_P34300*	527.68	-0.99	0.29	-3.39	7.01E-04	2.57E-02	Tubulin beta-1 chain
Ma04_g02630	ITC1587_Bchr4_P08630	186.88	-0.99	0.32	-3.07	2.15E-03	4.85E-02	uncharacterized LOC103980237
Ma10_g25040	ITC1587_Bchr10_P31099	78.21	-1	0.27	-3.75	1.79E-04	1.18E-02	probable protein phosphatase 2C 12
Ma06_g12640	ITC1587_Bchr6_P15773	349.5	-1	0.34	-2.96	3.03E-03	5.94E-02	probable protein phosphatase 2C 78
Ma01_g19220	ITC1587_Bchr1_P02321	318.58	-1	0.23	-4.33	1.51E-05	2.69E-03	uncharacterized LOC103998092
Ma05_g06290	ITC1587_Bchr5_P12247	124.5	-1	0.31	-3.27	1.09E-03	3.31E-02	profilin-like
Ma11_g20050	ITC1587_Bchr11_P33968	1031.87	-1	0.27	-3.68	2.38E-04	1.35E-02	homeobox-leucine zipper protein ROC5-like
Ma06_g37210		132.17	-1.01	0.28	-3.54	4.05E-04	1.90E-02	Hypothetical protein
Ma06_g07720	ITC1587_Bchr6_P15322*	32.83	-1.01	0.36	-2.81	4.92E-03	7.65E-02	Mitochondrial import inner membrane translocase subunit Tim8
Ma06_g32130	ITC1587_Bchr6_P17941	39.05	-1.01	0.34	-2.95	3.13E-03	6.04E-02	F-box protein FBW2-like
Ma08_g20040	ITC1587_Bchr8_P22916	58.65	-1.01	0.31	-3.3	9.77E-04	3.12E-02	glucan endo-1, 3-beta-glucosidase 4-like
Ma07_g26830	ITC1587_Bchr7_P21270	142.64	-1.01	0.27	-3.79	1.52E-04	1.07E-02	auxin response factor 3-like
Ma05_g29150	ITC1587_Bchr5_P14526	86.83	-1.02	0.32	-3.18	1.46E-03	3.95E-02	glycine cleavage system H protein 2
Ma02_g04640	ITC1587_Bchr2_P03334	145.92	-1.02	0.27	-3.81	1.37E-04	1.00E-02	GDP-mannose 4, 6 dehydratase 1-like
Ma06_g08180	ITC1587_Bchr6_P15366	82.03	-1.02	0.28	-3.63	2.88E-04	1.53E-02	U-box domain-containing protein 9-like
Ma08_g20600	ITC1587_Bchr8_P23801	160.88	-1.02	0.3	-3.37	7.65E-04	2.69E-02	uncharacterized LOC103995103
Ma07_g19300	ITC1587_Bchr7_P20588	149.05	-1.02	0.29	-3.47	5.15E-04	2.16E-02	probable galacturonosyltransferase 9
Ma01_g22680	ITC1587_Bchr8_P24834*	23.35	-1.02	0.31	-3.29	1.00E-03	3.16E-02	serine/threonine protein phosphatase 2A 57 kDa regulatory subunit B' theta isoform-like
Ma08_g06100	ITC1587_Bchr3_P07706*	98.25	-1.02	0.36	-2.82	4.73E-03	7.55E-02	GDSL esterase/lipase LIP-4-like
Ma10_g13960	ITC1587_Bchr10_P30136	206.06	-1.03	0.38	-2.67	7.55E-03	9.74E-02	glycerol-3-phosphate 2-O-acyltransferase 6-like
Ma05_g27310	ITC1587_Bchr5_P14358*	36.97	-1.03	0.38	-2.68	7.29E-03	9.57E-02	Peroxidase 55
Ma04_g17610	ITC1587_Bchr4_P10124	86.19	-1.03	0.33	-3.11	1.85E-03	4.48E-02	uncharacterized LOC103981816
Ma06_g19230	ITC1587_Bchr6_P16371	36.94	-1.03	0.35	-2.92	3.54E-03	6.46E-02	transcription factor TCP8-like

Ma05_g30610	ITC1587_Bchr5_P14656	3572.13	-1.03	0.34	-3.07	2.15E-03	4.85E-02	zinc finger CCCH domain-containing protein 33-like
Ma06_g38470	ITC1587_Bchr6_P18502*	656.48	-1.03	0.32	-3.2	1.37E-03	3.80E-02	probable methyltransferase PMT3
Ma03_g24950	ITC1587_Bchr3_P07641	19.66	-1.04	0.38	-2.71	6.74E-03	9.19E-02	ATPase WRNIP1
Ma11_g11550	ITC1587_BchrUn_random_P37128	69.06	-1.04	0.28	-3.74	1.87E-04	1.20E-02	nuclear transcription factor Y subunit A-7-like
Ma07_g26490	ITC1587_BchrUn_random_P39404	62.97	-1.04	0.38	-2.75	5.95E-03	8.51E-02	putative probable LRR receptor-like serine/threonine-protein kinase At1g51810
Ma04_g24480	ITC1587_Bchr1_P00447	481.22	-1.04	0.36	-2.86	4.29E-03	7.17E-02	calmodulin-like protein 8
Ma09_g03930	ITC1587_Bchr9_P25411	30.69	-1.04	0.36	-2.92	3.53E-03	6.45E-02	probable protein phosphatase 2C 23
Ma05_g14570	ITC1587_Bchr5_P12967	73.88	-1.04	0.36	-2.9	3.69E-03	6.58E-02	uncharacterized LOC103984958
Ma05_g23080	ITC1587_Bchr5_P13946*	188.32	-1.04	0.35	-2.96	3.06E-03	5.96E-02	nucleolar protein 58-like
Ma10_g10740	ITC1587_Bchr10_P29800	54.52	-1.04	0.39	-2.71	6.70E-03	9.16E-02	transcription factor TCP8-like
Ma05_g23510	ITC1587_Bchr5_P13995	414.42	-1.04	0.24	-4.35	1.38E-05	2.59E-03	ocs element-binding factor 1-like
Ma05_g31260	ITC1587_Bchr6_P18246*	20.25	-1.05	0.35	-3.02	2.56E-03	5.33E-02	nascent polypeptide-associated complex subunit alpha
Ma10_g03640	ITC1587_Bchr10_P29481	366.27	-1.05	0.32	-3.3	9.71E-04	3.10E-02	probable WRKY transcription factor 40
Ma04_g23800	ITC1587_Bchr1_P00382	49.14	-1.05	0.38	-2.81	4.99E-03	7.70E-02	calcium-transporting ATPase
Ma09_g29440	ITC1587_Bchr9_P28163	819.81	-1.05	0.39	-2.67	7.54E-03	9.74E-02	U-box domain-containing protein 17-like
Ma07_g06680	ITC1587_Bchr7_P19143	43.54	-1.06	0.36	-2.94	3.24E-03	6.16E-02	GTP-binding protein YPTM2-like
Ma09_g28550		12.34	-1.06	0.39	-2.72	6.57E-03	9.05E-02	uncharacterized LOC103999241
Ma08_g08170	ITC1587_Bchr8_P22174	45.8	-1.06	0.34	-3.14	1.66E-03	4.24E-02	RNA recognition motif containing protein
Ma07_g11090	ITC1587_Bchr10_P31164*	83.47	-1.06	0.31	-3.4	6.67E-04	2.49E-02	homeobox-leucine zipper protein ROC8-like
Ma05_g03960	ITC1587_Bchr5_P12030*	267.89	-1.06	0.27	-3.91	9.05E-05	7.92E-03	GPI-anchored protein LORELEI-like
Ma09_g04480	ITC1587_Bchr9_P25460	62.05	-1.06	0.4	-2.66	7.76E-03	9.85E-02	LIMR family protein Os06g0128200-like
Ma02_g04580	ITC1587_Bchr2_P03326	374.58	-1.06	0.34	-3.08	2.04E-03	4.73E-02	cysteine-rich and transmembrane domain-containing protein B-like
Ma09_g25000	ITC1587_Bchr9_P27754	591.59	-1.06	0.4	-2.68	7.29E-03	9.57E-02	sugar carrier protein C-like
Ma06_g26280	ITC1587_Bchr6_P17382	89.81	-1.07	0.3	-3.54	4.01E-04	1.90E-02	acidic leucine-rich nuclear phosphoprotein 32-related protein-like
Ma07_g28080	ITC1587_Bchr4_P08691*	621.55	-1.07	0.32	-3.31	9.34E-04	3.02E-02	UDP-glucose 6-dehydrogenase 4-like
Ma11_g05540	ITC1587_Bchr11_P32215	86.67	-1.08	0.37	-2.88	3.97E-03	6.85E-02	probable nucleolar protein 5-2

Ma11_g20040	ITC1587_Bchr11_P33966	396.72	-1.08	0.27	-4.01	6.10E-05	6.43E-03	inactive rhomboid protein 1-like
Ma06_g33550	ITC1587_Bchr6_P18079	197.84	-1.08	0.23	-4.63	3.65E-06	1.16E-03	respiratory burst oxidase homolog protein A-like
Ma06_g35430	ITC1587_Bchr6_P18220	147.89	-1.08	0.34	-3.19	1.43E-03	3.91E-02	L-type lectin-domain containing receptor kinase IV.4-like
Ma06_g02690	ITC1587_Bchr6_P14865	96.85	-1.08	0.29	-3.71	2.09E-04	1.26E-02	regulatory protein NPR1-like
Ma02_g15950	ITC1587_Bchr2_P04340	65.82	-1.08	0.4	-2.68	7.27E-03	9.55E-02	BTB/POZ domain-containing protein At3g22104-like
Ma07_g27160	ITC1587_Bchr7_P21295	318.82	-1.09	0.35	-3.08	2.08E-03	4.76E-02	2-dihydroxy-3-keto-5-methylthiopentene dioxygenase 2-like
Ma07_g18870	ITC1587_BchrUn_random _P35607	14.48	-1.09	0.36	-3.08	2.10E-03	4.78E-02	B-box zinc finger protein 20-like
Ma04_g23110	ITC1587_Bchr1_P00307	39.8	-1.1	0.36	-3.08	2.07E-03	4.74E-02	probable pectate lyase 5
Ma07_g08510	ITC1587_Bchr7_P19325	40.93	-1.1	0.39	-2.79	5.24E-03	7.94E-02	putative disease resistance protein RPS2
Ma03_g14160	ITC1587_BchrUn_random _P35655	158.61	-1.1	0.31	-3.52	4.25E-04	1.94E-02	RING-H2 finger protein ATL16-like
Ma08_g32440	ITC1587_Bchr8_P24842	36.8	-1.1	0.3	-3.71	2.07E-04	1.26E-02	putative glucose-6-phosphate 1-epimerase
Ma08_g23940	ITC1587_Bchr8_P24077	80.45	-1.1	0.28	-3.98	6.98E-05	6.80E-03	phosphatidylinositol 4-phosphate 5-kinase 9-like
Ma10_g19740	ITC1587_Bchr10_P30642*	42.34	-1.1	0.41	-2.69	7.23E-03	9.53E-02	probable calcium-binding protein CML18
Ma04_g24730	ITC1587_Bchr1_P00474	71.84	-1.1	0.42	-2.66	7.90E-03	9.97E-02	mannan endo-1, 4-beta-mannosidase 2-like
Ma04_g15890	ITC1587_Bchr4_P10027	131.03	-1.1	0.22	-5.11	3.16E-07	2.48E-04	F-box protein At5g39450-like
Ma04_g08960	ITC1587_Bchr4_P09177	203.95	-1.11	0.36	-3.05	2.29E-03	5.03E-02	probable inactive receptor kinase At2g26730
Ma04_g19590	ITC1587_Bchr4_P10423	39.84	-1.11	0.38	-2.94	3.27E-03	6.19E-02	E3 ubiquitin-protein ligase Os04g0590900-like
Ma01_g05050	ITC1587_Bchr1_P01020	552.21	-1.11	0.3	-3.71	2.07E-04	1.26E-02	protein EARLY RESPONSIVE TO DEHYDRATION 15-like
Ma06_g16730	ITC1587_Bchr6_P16137*	18.5	-1.11	0.4	-2.76	5.83E-03	8.42E-02	thaumatin-like protein
Ma03_g10820	ITC1587_Bchr3_P06160	152.89	-1.11	0.36	-3.11	1.84E-03	4.47E-02	polygalacturonase ADPG2
Ma11_g02320	ITC1587_Bchr11_P31906	24.13	-1.11	0.38	-2.96	3.08E-03	5.98E-02	uncharacterized LOC103970226
Ma05_g03220	ITC1587_Bchr5_P11951	33.55	-1.12	0.4	-2.81	4.96E-03	7.67E-02	uncharacterized LOC103983823
Ma02_g19100	ITC1587_Bchr2_P04623	30.69	-1.12	0.33	-3.33	8.60E-04	2.90E-02	peroxisomal membrane protein 13-like
Ma05_g04050	ITC1587_Bchr5_P12037	27.65	-1.12	0.4	-2.78	5.37E-03	8.06E-02	serine/threonine-protein kinase D6PK-like

Ma06_g20870	ITC1587_Bchr6_P16551	154.86	-1.12	0.3	-3.75	1.76E-04	1.17E-02	soluble inorganic pyrophosphatase-like
Ma10_g03660	ITC1587_Bchr10_P29479	35.55	-1.13	0.35	-3.23	1.24E-03	3.60E-02	uncharacterized LOC104000135
Ma09_g27170	ITC1587_Bchr9_P27958	668.11	-1.13	0.4	-2.81	5.03E-03	7.73E-02	zinc finger protein AZF3-like
Ma02_g14610	ITC1587_Bchr2_P04225	131.47	-1.13	0.37	-3.03	2.48E-03	5.24E-02	glucan endo-1, 3-beta-glucosidase 8-like
Ma04_g03820	ITC1587_Bchr4_P08730	49.32	-1.13	0.36	-3.18	1.47E-03	3.95E-02	probable ribose-5-phosphate isomerase 2
Ma11_g23380	ITC1587_Bchr5_P14617*	47.01	-1.13	0.39	-2.92	3.52E-03	6.44E-02	putative DUF246 domain-containing protein At1g04910
Ma11_g11680	ITC1587_Bchr11_P33142	10.44	-1.13	0.41	-2.75	5.94E-03	8.51E-02	pentatricopeptide repeat-containing protein At3g29230-like
Ma06_g16400	ITC1587_Bchr6_P16116	24.3	-1.14	0.4	-2.86	4.19E-03	7.07E-02	profilin-like
Ma00_g03890	ITC1587_BchrUn_random _P37065	26.56	-1.14	0.31	-3.73	1.91E-04	1.22E-02	50S ribosomal protein L12
Ma02_g23470	ITC1587_Bchr2_P05018	69.07	-1.14	0.38	-2.99	2.80E-03	5.68E-02	glucan endo-1, 3-beta-glucosidase 3-like
Ma11_g06570	ITC1587_Bchr11_P32330	28.7	-1.14	0.35	-3.22	1.26E-03	3.62E-02	CASP-like protein 1
Ma05_g17880	ITC1587_Bchr5_P13390*	26.98	-1.14	0.34	-3.31	9.17E-04	2.99E-02	uncharacterized LOC103985228
Ma09_g10110	ITC1587_Bchr9_P25933	74.93	-1.14	0.4	-2.86	4.24E-03	7.11E-02	uncharacterized LOC103997556
Ma09_g18880	ITC1587_Bchr9_P27191	146.24	-1.15	0.42	-2.7	6.86E-03	9.26E-02	uncharacterized LOC103998466
Ma05_g03160	ITC1587_Bchr5_P11945	32.54	-1.16	0.38	-3.06	2.22E-03	4.93E-02	transcription factor PCF2-like
Ma06_g01570	ITC1587_Bchr11_P32729	320.26	-1.16	0.37	-3.1	1.92E-03	4.55E-02	fructokinase-1-like
Ma09_g25110	ITC1587_Bchr9_P27770	135.21	-1.16	0.39	-3	2.68E-03	5.53E-02	uncharacterized LOC103998875
Ma01_g06030	ITC1587_Bchr1_P01106	32.88	-1.16	0.4	-2.91	3.62E-03	6.54E-02	solute carrier family 25 member 44-like
Ma08_g26380	ITC1587_Bchr8_P24294	67.94	-1.16	0.37	-3.16	1.57E-03	4.10E-02	probably inactive leucine-rich repeat receptor-like protein kinase At5g06940
Ma00_g00550	ITC1587_BchrUn_random _P35275	301.06	-1.16	0.33	-3.54	4.02E-04	1.90E-02	transmembrane protein 53-A
Ma03_g01910	ITC1587_Bchr3_P05339	1062.41	-1.16	0.36	-3.27	1.08E-03	3.30E-02	3-ketoacyl-CoA synthase 10
Ma06_g16010	ITC1587_Bchr6_P16081	160.06	-1.17	0.34	-3.47	5.20E-04	2.17E-02	xylem cysteine proteinase 1-like
Ma05_g11120	ITC1587_Bchr1_P00949*	57.72	-1.17	0.41	-2.87	4.07E-03	6.97E-02	uncharacterized LOC103984561
Ma05_g13370	ITC1587_Bchr5_P12862*	29.25	-1.17	0.38	-3.07	2.15E-03	4.85E-02	malate dehydrogenase
Ma02_g19020	ITC1587_Bchr2_P04615	621.57	-1.17	0.35	-3.37	7.51E-04	2.67E-02	probable LRR receptor-like serine/threonine-protein kinase At2g16250
Ma08_g07800	ITC1587_Bchr8_P22132	77.74	-1.17	0.36	-3.21	1.31E-03	3.70E-02	cellulose synthase A catalytic subunit 5 [UDP-forming]-like

Ma03_g29900	ITC1587_Bchr3_P08055*	21.79	-1.17	0.44	-2.69	7.10E-03	9.47E-02	putative cysteine-rich receptor-like protein kinase 41
Ma09_g03040	ITC1587_Bchr9_P25338	180.25	-1.18	0.39	-3.02	2.51E-03	5.27E-02	floral homeotic protein APETALA 2-like
Ma01_g09270	ITC1587_Bchr1_P01396	151.73	-1.18	0.33	-3.62	2.92E-04	1.54E-02	S-adenosylmethionine synthase 2
Ma10_g15180	ITC1587_Bchr10_P30256	58.28	-1.18	0.42	-2.79	5.24E-03	7.94E-02	auxin response factor 11-like
Ma01_g20240	ITC1587_Bchr1_P02427	29.09	-1.18	0.43	-2.77	5.52E-03	8.17E-02	beta-glucosidase 25-like
Ma01_g03980	ITC1587_Bchr1_P00788	359.05	-1.18	0.4	-2.99	2.81E-03	5.69E-02	uncharacterized LOC103978475
Ma04_g04160	ITC1587_Bchr4_P09410*	47.16	-1.18	0.35	-3.37	7.54E-04	2.67E-02	D-3-phosphoglycerate dehydrogenase 1
Ma05_g02460	ITC1587_Bchr5_P11872	120.28	-1.19	0.36	-3.34	8.43E-04	2.86E-02	F-box protein At5g46170-like
Ma01_g12260	ITC1587_Bchr1_P01674	249.5	-1.19	0.41	-2.89	3.82E-03	6.71E-02	transcription factor PIF3-like
Ma02_g03950	ITC1587_Bchr2_P03279	99.18	-1.19	0.32	-3.69	2.29E-04	1.32E-02	uncharacterized LOC103969571
Ma05_g19310	ITC1587_Bchr5_P13299	39.63	-1.19	0.38	-3.1	1.92E-03	4.55E-02	26S protease regulatory subunit 6A-like
Ma03_g22510	ITC1587_Bchr3_P07443	39.32	-1.19	0.39	-3.04	2.39E-03	5.14E-02	uncharacterized LOC103979183
Ma07_g12800	ITC1587_Bchr7_P19747	90.27	-1.19	0.4	-2.95	3.23E-03	6.15E-02	probable protein phosphatase 2C 12
Ma07_g13610	ITC1587_Bchr7_P19823	57.32	-1.19	0.37	-3.24	1.18E-03	3.48E-02	monoglyceride lipase-like
Ma10_g22970	ITC1587_Bchr10_P30923	40.11	-1.2	0.44	-2.69	7.16E-03	9.50E-02	trihelix transcription factor GT-3b-like
Ma08_g03470	ITC1587_Bchr8_P21762	292.43	-1.2	0.37	-3.26	1.13E-03	3.39E-02	putative ethylene-responsive transcription factor 4
Ma06_g01240	ITC1587_Bchr11_P32698	40.68	-1.2	0.44	-2.75	5.98E-03	8.54E-02	dof zinc finger protein DOF5.3-like
Ma04_g13060	ITC1587_Bchr2_P03593*	141.9	-1.2	0.3	-3.92	8.68E-05	7.77E-03	probable ADP-ribosylation factor GTPase-activating protein AGD13
Ma04_g35850	ITC1587_Bchr4_P11315	15.51	-1.2	0.43	-2.77	5.63E-03	8.24E-02	LRR receptor-like serine/threonine-protein kinase FLS2
Ma01_g18040	ITC1587_BchrUn_random_P38021	179.03	-1.2	0.45	-2.67	7.50E-03	9.72E-02	probable linoleate 9S-lipoxygenase 5
Ma10_g10050	ITC1587_Bchr10_P29744	289.3	-1.2	0.35	-3.44	5.91E-04	2.34E-02	probable protein NAP1
Ma10_g06510	ITC1587_Bchr10_P28573	102.14	-1.2	0.37	-3.26	1.11E-03	3.35E-02	uncharacterized LOC103999709
Ma01_g17200	ITC1587_Bchr1_P02273	112.29	-1.2	0.36	-3.3	9.65E-04	3.08E-02	uncharacterized LOC103997286
Ma03_g00880	ITC1587_Bchr3_P05242	50.3	-1.21	0.44	-2.77	5.55E-03	8.19E-02	probable LRR receptor-like serine/threonine-protein kinase At2g24230
Ma10_g24690	ITC1587_Bchr10_P31070	82.85	-1.21	0.45	-2.71	6.63E-03	9.10E-02	uncharacterized LOC103969146
Ma04_g35250	ITC1587_Bchr4_P11264	117.54	-1.21	0.44	-2.74	6.05E-03	8.58E-02	uncharacterized LOC103982467

Ma11_g01700	ITC1587_Bchr11_P31841	104.12	-1.22	0.43	-2.84	4.48E-03	7.32E-02	transcription factor TCP21-like
Ma06_g38740	ITC1587_Bchr6_P18519	26.74	-1.22	0.38	-3.19	1.44E-03	3.91E-02	putative histone-lysine N-methyltransferase
Ma09_g19480	ITC1587_Bchr1_P02003*	21.33	-1.22	0.4	-3.07	2.14E-03	4.85E-02	putative protein SEC13 homolog
Ma08_g04820	ITC1587_Bchr8_P21876	53.09	-1.22	0.34	-3.55	3.86E-04	1.85E-02	protein phloem protein 2-like A10-like
Ma05_g24280	ITC1587_Bchr5_P14081	122.33	-1.22	0.25	-4.87	1.12E-06	5.46E-04	protein enhanced disease resistance 2-like
Ma04_g22460	ITC1587_Bchr1_P00249	62.05	-1.22	0.4	-3.08	2.04E-03	4.73E-02	24-methylenesterol C-methyltransferase 2
Ma04_g40140	ITC1587_Bchr4_P11668	46.37	-1.23	0.42	-2.92	3.52E-03	6.44E-02	receptor-like serine/threonine-protein kinase SD1-8
Ma04_g22750	ITC1587_Bchr1_P00270	37.74	-1.23	0.36	-3.42	6.31E-04	2.44E-02	elicitor-responsive protein 3-like
Ma06_g22380	ITC1587_Bchr9_P25673*	146.84	-1.23	0.33	-3.76	1.69E-04	1.14E-02	40S ribosomal protein S8-like
Ma05_g17050	ITC1587_Bchr4_P11046*	66.29	-1.24	0.34	-3.69	2.29E-04	1.32E-02	oxidoreductase
Ma03_g22790	ITC1587_Bchr3_P07468	41.64	-1.24	0.42	-2.98	2.88E-03	5.79E-02	RNA-binding protein 24-A-like
Ma04_g04300	ITC1587_Bchr4_P08767	160.6	-1.25	0.43	-2.92	3.50E-03	6.42E-02	protein ASPARTIC PROTEASE IN GUARD CELL 2-like
Ma05_g06430	ITC1587_Bchr5_P12261	474.61	-1.25	0.43	-2.9	3.67E-03	6.57E-02	transcription factor TCP4-like
Ma03_g01200	ITC1587_Bchr3_P05274	41.88	-1.25	0.37	-3.36	7.79E-04	2.72E-02	Hypothetical protein
Ma04_g19480	ITC1587_Bchr4_P10414	36.29	-1.26	0.43	-2.9	3.75E-03	6.61E-02	homeobox-leucine zipper protein ROC3
Ma10_g24780	ITC1587_Bchr10_P31078	422.11	-1.26	0.44	-2.86	4.30E-03	7.17E-02	E3 ubiquitin-protein ligase ATL6-like
Ma10_g18880	ITC1587_Bchr6_P15717*	66.14	-1.26	0.38	-3.34	8.28E-04	2.84E-02	uncharacterized LOC103968626
Ma08_g26630	ITC1587_Bchr3_P06099*	55.14	-1.26	0.37	-3.37	7.63E-04	2.69E-02	nucleobase-ascorbate transporter 6-like
Ma03_g16090	ITC1587_Bchr3_P06760	189.09	-1.26	0.44	-2.85	4.43E-03	7.28E-02	homeobox-leucine zipper protein HOX19
Ma03_g24180	ITC1587_Bchr3_P07576	336.46	-1.26	0.43	-2.93	3.43E-03	6.31E-02	calcium-binding protein PBP1-like
Ma08_g09370	ITC1587_Bchr8_P22281	32.9	-1.26	0.33	-3.87	1.08E-04	8.69E-03	U-box domain-containing protein 4-like
Ma03_g01740	ITC1587_Bchr3_P05326	60.39	-1.26	0.36	-3.52	4.28E-04	1.95E-02	E3 ubiquitin-protein ligase RING1-like
Ma02_g14140	ITC1587_Bchr2_P04180	19.53	-1.27	0.37	-3.43	6.08E-04	2.38E-02	uncharacterized LOC103975734
Ma09_g11730	ITC1587_Bchr9_P26079	137.11	-1.27	0.36	-3.5	4.59E-04	2.03E-02	disease resistance RPP13-like protein 4
Ma05_g06580	ITC1587_Bchr3_P08325*	12.3	-1.27	0.44	-2.88	3.93E-03	6.82E-02	expressed protein
Ma03_g18680	ITC1587_Bchr3_P07069	55.72	-1.28	0.46	-2.74	6.10E-03	8.61E-02	methyltransferase-like protein 10
Ma06_g13290	ITC1587_Bchr6_P15831	365.05	-1.28	0.47	-2.71	6.79E-03	9.22E-02	respiratory burst oxidase homolog protein B

Ma11_g22440	ITC1587_Bchr11_P34176	2114.09	-1.28	0.34	-3.81	1.39E-04	1.01E-02	nematode resistance protein-like HSPRO2
Ma06_g13590	ITC1587_Bchr6_P15858*	168.09	-1.28	0.36	-3.53	4.21E-04	1.93E-02	BTB/POZ and TAZ domain-containing protein 2-like
Ma03_g25900	ITC1587_Bchr3_P07722	200.61	-1.28	0.42	-3.09	2.02E-03	4.72E-02	arogenate dehydratase/prephenate dehydratase 2
Ma10_g11380	ITC1587_Bchr10_P29925	35.46	-1.28	0.41	-3.11	1.90E-03	4.54E-02	60S acidic ribosomal protein P2A-like
Ma06_g16200	ITC1587_Bchr6_P16096	83.84	-1.28	0.36	-3.53	4.13E-04	1.92E-02	forkhead box protein G1-like
Ma06_g17740	ITC1587_Bchr10_P30024*	202.36	-1.28	0.45	-2.88	3.98E-03	6.87E-02	acetyl-coenzyme A synthetase
Ma01_g05830	ITC1587_Bchr1_P01089	37.6	-1.29	0.41	-3.12	1.79E-03	4.42E-02	uncharacterized LOC103987768
Ma03_g13230	ITC1587_BchrUn_random_P37674	181.05	-1.29	0.37	-3.48	5.04E-04	2.13E-02	tetraspanin-7-like
Ma07_g14220	ITC1587_Bchr7_P19882	59.15	-1.29	0.3	-4.25	2.11E-05	3.33E-03	WRKY transcription factor 22-like
Ma09_g11910	ITC1587_Bchr9_P26094	347.12	-1.29	0.48	-2.67	7.48E-03	9.71E-02	probable L-type lectin-domain containing receptor kinase S.5
Ma08_g11940	ITC1587_Bchr8_P22529	34.47	-1.29	0.34	-3.76	1.68E-04	1.14E-02	transcription repressor OFP7-like
Ma06_g03290		86.79	-1.29	0.47	-2.77	5.59E-03	8.22E-02	Hypothetical protein
Ma05_g22440	ITC1587_Bchr5_P13876	110.33	-1.29	0.48	-2.69	7.13E-03	9.48E-02	homeobox-leucine zipper protein ROC5
Ma08_g19330	ITC1587_Bchr8_P22997	24.2	-1.29	0.33	-3.87	1.09E-04	8.71E-03	serine/threonine-protein kinase D6PK-like
Ma11_g22820	ITC1587_Bchr11_P34203	73.22	-1.3	0.42	-3.07	2.11E-03	4.81E-02	protein UPSTREAM OF FLC-like
Ma11_g21200	ITC1587_Bchr11_P34074	183.15	-1.3	0.29	-4.47	7.99E-06	1.93E-03	auxin response factor 18-like
Ma03_g32830	ITC1587_Bchr3_P08296	102.46	-1.3	0.44	-2.96	3.05E-03	5.96E-02	protein ASPARTIC PROTEASE IN GUARD CELL 1-like
Ma08_g28200	ITC1587_Bchr8_P24460	28.05	-1.31	0.46	-2.82	4.86E-03	7.62E-02	putative leucine-rich repeat receptor-like serine/threonine-protein kinase At2g19230
Ma03_g24830	ITC1587_Bchr5_P14526*	25.63	-1.31	0.37	-3.56	3.76E-04	1.82E-02	glycine cleavage system H protein 2
Ma03_g06150	ITC1587_Bchr3_P05717	1090.27	-1.31	0.3	-4.33	1.51E-05	2.69E-03	putative endo-1
Ma05_g29040	ITC1587_Bchr5_P14516	231.57	-1.31	0.47	-2.76	5.70E-03	8.30E-02	Tubulin beta-1 chain
Ma10_g15690	ITC1587_Bchr10_P30298	38.65	-1.31	0.43	-3.04	2.38E-03	5.14E-02	glucan endo-1
Ma01_g17640	ITC1587_Bchr1_P02230	165.11	-1.32	0.47	-2.79	5.20E-03	7.91E-02	putative glycosyltransferase 2
Ma06_g15260	ITC1587_Bchr6_P16005	100.05	-1.32	0.36	-3.63	2.85E-04	1.52E-02	aspartic proteinase nepenthesin-2-like
Ma03_g14800	ITC1587_Bchr3_P06579	25.35	-1.32	0.49	-2.71	6.70E-03	9.16E-02	dof zinc finger protein DOF1.4-like
Ma10_g26480	ITC1587_Bchr10_P31217	84.87	-1.32	0.4	-3.32	9.02E-04	2.98E-02	methylsterol monooxygenase 2-1-like

Ma05_g06780	ITC1587_Bchr5_P12286	41.92	-1.33	0.39	-3.43	6.03E-04	2.37E-02	BEL1-like homeodomain protein 9
Ma10_g10220	ITC1587_Bchr10_P29758	109.07	-1.33	0.31	-4.34	1.45E-05	2.64E-03	uncharacterized LOC104000324
Ma03_g08390	ITC1587_Bchr3_P05933	539.59	-1.33	0.36	-3.72	1.96E-04	1.23E-02	S-adenosylmethionine synthase
Ma10_g22570	ITC1587_Bchr10_P30887	1685.09	-1.33	0.33	-4.1	4.17E-05	5.06E-03	E3 ubiquitin-protein ligase RING1
Ma06_g12960	ITC1587_Bchr10_P30481*	184.33	-1.34	0.5	-2.67	7.69E-03	9.83E-02	uncharacterized LOC103987550
Ma05_g28100	ITC1587_Bchr5_P14423	302.95	-1.34	0.4	-3.39	7.04E-04	2.57E-02	premnaspirodiene oxygenase-like
Ma08_g09940	ITC1587_Bchr8_P22331	14	-1.35	0.5	-2.67	7.56E-03	9.74E-02	protein FAM179A-like
Ma11_g22270	ITC1587_Bchr11_P34163	145	-1.35	0.48	-2.82	4.79E-03	7.58E-02	Actin-2
Ma04_g04550	ITC1587_Bchr4_P08793	17.76	-1.35	0.38	-3.5	4.57E-04	2.02E-02	probable transcription factor KAN2
Ma11_g24030	ITC1587_Bchr11_P34300	707.9	-1.35	0.5	-2.7	6.86E-03	9.26E-02	Tubulin beta-1 chain
Ma07_g09810	ITC1587_Bchr6_P15238*	23.73	-1.35	0.43	-3.16	1.60E-03	4.16E-02	uncharacterized protein At2g34160-like
Ma03_g30350	ITC1587_Bchr3_P08083	158.95	-1.35	0.49	-2.75	5.88E-03	8.46E-02	uncharacterized LOC103980088
Ma07_g03780	ITC1587_Bchr7_P18881	287.9	-1.36	0.37	-3.67	2.44E-04	1.37E-02	bifunctional 3-dehydroquinate dehydratase/shikimate dehydrogenase
Ma02_g06820	ITC1587_Bchr2_P03543	84.13	-1.36	0.42	-3.26	1.11E-03	3.36E-02	plasma membrane ATPase-like
Ma08_g02910	ITC1587_Bchr8_P21722	133.16	-1.36	0.31	-4.41	1.05E-05	2.28E-03	syntaxin-121-like
Ma03_g17530	ITC1587_Bchr3_P06901	22.37	-1.36	0.39	-3.52	4.29E-04	1.95E-02	uncharacterized LOC103978427
Ma10_g02120	ITC1587_Bchr10_P28877	45.63	-1.36	0.42	-3.24	1.21E-03	3.54E-02	histone H2A-like
Ma09_g11100	ITC1587_Bchr9_P26023	437.72	-1.37	0.34	-4.01	6.05E-05	6.42E-03	putative ethylene-responsive transcription factor 4
Ma03_g24170	ITC1587_BchrUn_random _P38237*	13.43	-1.37	0.46	-2.96	3.05E-03	5.96E-02	G-type lectin S-receptor-like serine/threonine-protein kinase At4g03230
Ma03_g26900	ITC1587_Bchr3_P07811	106.07	-1.37	0.37	-3.67	2.43E-04	1.37E-02	BTB/POZ domain-containing protein At5g66560-like
Ma05_g23560	ITC1587_Bchr5_P14000	287.76	-1.37	0.38	-3.56	3.73E-04	1.82E-02	uncharacterized LOC103985615
Ma10_g11400	ITC1587_Bchr10_P29928	14.33	-1.37	0.47	-2.94	3.33E-03	6.22E-02	uncharacterized LOC104000788
Ma10_g25680	ITC1587_Bchr10_P31149	10.06	-1.37	0.44	-3.13	1.76E-03	4.37E-02	F-box only protein 13-like
Ma11_g22720	ITC1587_Bchr10_P30895*	1400.19	-1.38	0.37	-3.71	2.04E-04	1.26E-02	metallothionein-like protein type 2
Ma09_g06810	ITC1587_Bchr9_P25642	46.15	-1.38	0.47	-2.93	3.35E-03	6.25E-02	protein SPIRAL1-like 3
Ma06_g14710	ITC1587_Bchr6_P15949	138.16	-1.38	0.43	-3.25	1.16E-03	3.45E-02	Beta-fructofuranosidase
Ma08_g04940	ITC1587_Bchr8_P21884	142.99	-1.38	0.4	-3.48	4.92E-04	2.09E-02	homeobox-leucine zipper protein HOX19-like

Ma05_g11010	ITC1587_Bchr5_P12657	38.82	-1.38	0.5	-2.75	5.87E-03	8.45E-02	thaumatin-like protein 1b
Ma06_g29160	ITC1587_Bchr8_P24509*	22.86	-1.39	0.36	-3.82	1.31E-04	9.80E-03	GDP-mannose transporter GONST3-like
Ma08_g14350	ITC1587_Bchr8_P23396	66.73	-1.39	0.44	-3.14	1.69E-03	4.29E-02	probable leucine-rich repeat receptor-like protein kinase At1g68400
Ma04_g18160	ITC1587_Bchr4_P10236	42.21	-1.39	0.47	-2.96	3.04E-03	5.95E-02	putative glutamine amidotransferase YLR126C
Ma11_g21660	ITC1587_Bchr11_P34111	34.22	-1.39	0.5	-2.77	5.55E-03	8.19E-02	inosine-5'-monophosphate dehydrogenase-like
Ma01_g17160	ITC1587_Bchr1_P02277	13.84	-1.4	0.52	-2.67	7.55E-03	9.74E-02	probable calcium-binding protein CML14
Ma10_g27470	ITC1587_BchrUn_random_P35070*	96.77	-1.4	0.46	-3.03	2.42E-03	5.18E-02	transmembrane 9 superfamily member 4-like
Ma06_g05980	ITC1587_Bchr6_P15180	181.59	-1.4	0.39	-3.55	3.90E-04	1.86E-02	serine/threonine-protein kinase At5g01020-like
Ma05_g06590	ITC1587_Bchr5_P12271	202.61	-1.4	0.5	-2.8	5.16E-03	7.87E-02	probable inactive receptor kinase At4g23740
Ma07_g10510	ITC1587_BchrUn_random_P39657	23.08	-1.4	0.52	-2.68	7.28E-03	9.57E-02	uncharacterized LOC103975064
Ma00_g03220	ITC1587_BchrUn_random_P38524	101.6	-1.4	0.47	-2.96	3.10E-03	6.01E-02	vacuolar-sorting receptor 6-like
Ma07_g18260	ITC1587_Bchr2_P03467*	41.42	-1.4	0.52	-2.68	7.41E-03	9.66E-02	serine carboxypeptidase-like 51
Ma07_g24960	ITC1587_Bchr7_P21106	17.81	-1.41	0.5	-2.8	5.10E-03	7.80E-02	serine/threonine-protein kinase-like protein CCR4
Ma09_g10930	ITC1587_Bchr9_P26007*	15.36	-1.41	0.49	-2.85	4.33E-03	7.19E-02	DNA binding protein
Ma01_g04570	ITC1587_Bchr1_P00848	34.66	-1.41	0.53	-2.66	7.71E-03	9.83E-02	protein LIGHT-DEPENDENT SHORT HYPOCOTYLS 4-like
Ma11_g10680	ITC1587_BchrUn_random_P34403	53.45	-1.42	0.42	-3.39	7.03E-04	2.57E-02	transcription factor MYB108-like
Ma01_g10480	ITC1587_Bchr10_P30895*	1003.73	-1.42	0.45	-3.13	1.77E-03	4.38E-02	metallothionein-like protein 2A
Ma08_g01300	ITC1587_Bchr8_P21590	138.86	-1.42	0.33	-4.38	1.20E-05	2.43E-03	Whole genome shotgun sequence of line PN40024
Ma10_g05230	ITC1587_Bchr8_P21629*	11.31	-1.42	0.43	-3.33	8.63E-04	2.90E-02	importin subunit beta-1-like
Ma04_g11410	ITC1587_Bchr4_P09411	37.17	-1.43	0.41	-3.46	5.48E-04	2.23E-02	uncharacterized LOC103981192
Ma10_g18290	ITC1587_Bchr10_P30520	216.25	-1.43	0.36	-3.94	8.31E-05	7.57E-03	UDP-glucose 6-dehydrogenase 4-like
Ma01_g13620	ITC1587_Bchr1_P01788	129.35	-1.43	0.49	-2.91	3.57E-03	6.47E-02	putative acid phosphatase 1
Ma07_g23800	ITC1587_Bchr7_P21000*	236.89	-1.43	0.31	-4.58	4.57E-06	1.28E-03	PRA1 family protein B4-like

Ma05_g15470	ITC1587_Bchr5_P13066	127.77	-1.44	0.38	-3.8	1.47E-04	1.05E-02	probable inorganic phosphate transporter 1-8
Ma09_g30460	ITC1587_Bchr9_P28255	22.09	-1.44	0.45	-3.17	1.51E-03	4.03E-02	uncharacterized LOC103999560
Ma04_g28230	ITC1587_Bchr11_P33475*	29.73	-1.44	0.41	-3.48	4.97E-04	2.10E-02	uncharacterized LOC103983064
Ma01_g03010	ITC1587_Bchr1_P00694*	97.53	-1.44	0.53	-2.7	6.87E-03	9.27E-02	protein STAY-GREEN
Ma07_g18480	ITC1587_BchrUn_random_P37067	271.06	-1.44	0.39	-3.66	2.55E-04	1.42E-02	uncharacterized LOC103974030
Ma02_g15930	ITC1587_Bchr2_P04339*	19.14	-1.45	0.46	-3.17	1.54E-03	4.08E-02	formin-like protein 11
Ma04_g18740	ITC1587_Bchr4_P10331	135.54	-1.45	0.53	-2.73	6.32E-03	8.82E-02	Myb-related protein Myb4
Ma06_g18720	ITC1587_Bchr6_P16327	38.45	-1.45	0.48	-3.01	2.60E-03	5.41E-02	serine/threonine-protein kinase D6PKL2-like
Ma06_g28160	ITC1587_Bchr6_P17572	10.45	-1.45	0.5	-2.9	3.73E-03	6.60E-02	putative dual specificity protein phosphatase DSP8
Ma03_g26950	ITC1587_Bchr3_P07815*	20.28	-1.45	0.47	-3.09	2.00E-03	4.68E-02	uncharacterized LOC103978798
Ma02_g00320	ITC1587_BchrUn_random_P36466	120.03	-1.45	0.37	-3.94	8.10E-05	7.40E-03	COBRA-like protein 7
Ma07_g22480	ITC1587_Bchr7_P20882	89.75	-1.46	0.32	-4.48	7.53E-06	1.85E-03	uncharacterized protein At3g28850-like
Ma09_g00610	ITC1587_Bchr9_P25116	131.04	-1.46	0.46	-3.15	1.64E-03	4.22E-02	uncharacterized LOC103996591
Ma09_g28410	ITC1587_Bchr9_P28064	87.46	-1.46	0.39	-3.77	1.61E-04	1.11E-02	probably inactive leucine-rich repeat receptor-like protein kinase At5g06940
Ma05_g04590	ITC1587_Bchr5_P12091	21.71	-1.46	0.5	-2.94	3.33E-03	6.22E-02	protein ENHANCED DISEASE RESISTANCE 2-like
Ma03_g29050	ITC1587_Bchr3_P08058*	94.82	-1.46	0.5	-2.94	3.27E-03	6.19E-02	probable leucine-rich repeat receptor-like protein kinase At5g63930
Ma04_g04440	ITC1587_Bchr4_P08780	10.09	-1.46	0.54	-2.7	6.96E-03	9.35E-02	putative kinase-like protein TMKL1
Ma08_g02670	ITC1587_Bchr9_P27947*	112.82	-1.46	0.3	-4.91	9.29E-07	5.16E-04	wall-associated receptor kinase-like 14
Ma09_g31010	ITC1587_Bchr9_P28297	52.22	-1.46	0.42	-3.5	4.70E-04	2.05E-02	uncharacterized LOC103999518
Ma05_g14050	ITC1587_Bchr5_P12922	85.76	-1.47	0.54	-2.72	6.60E-03	9.08E-02	homeobox-leucine zipper protein ROC3-like
Ma07_g11470	ITC1587_Bchr7_P19614*	37.14	-1.47	0.51	-2.87	4.14E-03	7.02E-02	uncharacterized glycosyl hydrolase Rv2006/MT2062
Ma02_g01190	ITC1587_Bchr2_P03173	84.88	-1.47	0.53	-2.77	5.56E-03	8.20E-02	uncharacterized LOC103968921
Ma07_g15490	ITC1587_Bchr7_P20007	608.55	-1.47	0.36	-4.06	4.91E-05	5.60E-03	probable protein phosphatase 2C 32
Ma01_g14460	ITC1587_Bchr1_P01857	456.99	-1.48	0.49	-3.03	2.48E-03	5.24E-02	uncharacterized LOC103991966

Ma07_g24410	ITC1587_Bchr7_P21054	49.95	-1.49	0.35	-4.3	1.74E-05	2.91E-03	squamosa promoter-binding-like protein 16
Ma02_g14160	ITC1587_Bchr2_P04183	26.65	-1.49	0.52	-2.87	4.14E-03	7.02E-02	phosphoenolpyruvate carboxylase kinase 2-like
Ma06_g16790	ITC1587_Bchr6_P16144	45.12	-1.49	0.49	-3.04	2.35E-03	5.10E-02	putative wall-associated receptor kinase-like 16
Ma04_g22340	ITC1587_Bchr1_P00239	32.79	-1.49	0.56	-2.66	7.87E-03	9.95E-02	uncharacterized LOC103977327
Ma11_g16180	ITC1587_Bchr11_P33443	14.16	-1.49	0.43	-3.46	5.46E-04	2.23E-02	agamous-like MADS-box protein AGL80
Ma02_g01750	ITC1587_BchrUn_random_P38966	103.14	-1.5	0.54	-2.78	5.36E-03	8.06E-02	uncharacterized LOC103975027
Ma11_g23040	ITC1587_Bchr11_P34222	452.47	-1.5	0.35	-4.28	1.90E-05	3.13E-03	zinc finger CCCH domain-containing protein 33-like
Ma04_g00220	ITC1587_Bchr3_P06002*	58.19	-1.5	0.5	-3.02	2.53E-03	5.31E-02	putative auxin-induced protein 5NG4
Ma03_g18040	ITC1587_Bchr3_P06978*	332.68	-1.5	0.52	-2.9	3.75E-03	6.61E-02	calmodulin-like protein 3
Ma07_g10340	ITC1587_Bchr7_P19518	56.41	-1.5	0.48	-3.14	1.68E-03	4.27E-02	transcription factor AS1-like
Ma11_g22040	ITC1587_Bchr11_P34145	52.46	-1.5	0.43	-3.47	5.30E-04	2.19E-02	uncharacterized LOC103972236
Ma08_g23320	ITC1587_Bchr8_P24019	268.19	-1.51	0.47	-3.18	1.47E-03	3.95E-02	formin-like protein 16
Ma02_g23190	ITC1587_Bchr2_P04997	46.34	-1.52	0.4	-3.82	1.34E-04	9.95E-03	probable BOI-related E3 ubiquitin-protein ligase 2
Ma04_g08450	ITC1587_Bchr4_P09125	112.99	-1.52	0.54	-2.83	4.62E-03	7.49E-02	uncharacterized LOC103980939
Ma05_g29260	ITC1587_Bchr5_P14533	378.95	-1.52	0.51	-3	2.71E-03	5.56E-02	uncharacterized LOC103986497
Ma07_g14210	ITC1587_Bchr7_P19543*	100.5	-1.52	0.51	-2.99	2.83E-03	5.71E-02	protein SGT1 homolog
Ma03_g24970	ITC1587_Bchr3_P07643	272.66	-1.53	0.45	-3.41	6.56E-04	2.48E-02	putative ethylene-responsive transcription factor 11
Ma03_g25490	ITC1587_Bchr3_P07688	22.48	-1.53	0.55	-2.79	5.33E-03	8.01E-02	serine/arginine repetitive matrix protein 1-like
Ma09_g22170	ITC1587_Bchr9_P27468*	181.08	-1.53	0.41	-3.74	1.80E-04	1.18E-02	uncharacterized LOC103998624
Ma10_g28280	ITC1587_Bchr10_P31382	267.12	-1.54	0.48	-3.23	1.24E-03	3.59E-02	leucine-rich repeat extensin-like protein 3
Ma05_g28740	ITC1587_Bchr5_P14480	95.66	-1.54	0.39	-3.89	1.00E-04	8.37E-03	calcium-binding protein KIC-like
Ma09_g03600	ITC1587_Bchr9_P25383	38.37	-1.54	0.5	-3.05	2.28E-03	5.01E-02	auxin transporter-like protein 2
Ma06_g34670	ITC1587_Bchr6_P18175	9.69	-1.55	0.58	-2.68	7.35E-03	9.62E-02	uncharacterized LOC103989717
Ma03_g15260	ITC1587_Bchr3_P06712	71.93	-1.55	0.51	-3.03	2.44E-03	5.20E-02	probable serine/threonine-protein kinase NAK

Ma02_g17710	ITC1587_Bchr2_P04498	48.34	-1.55	0.45	-3.48	5.01E-04	2.12E-02	phosphatidylcholine:diacylglycerol cholinephosphotransferase 1-like
Ma06_g28400	ITC1587_Bchr6_P17592	227.18	-1.55	0.49	-3.18	1.48E-03	3.97E-02	aspartic proteinase nepenthesin-2-like
Ma07_g16650	ITC1587_Bchr7_P20364	291.49	-1.55	0.42	-3.71	2.05E-04	1.26E-02	RING-H2 finger protein ATL2-like
Ma05_g12170	ITC1587_Bchr1_P00039*	1116.97	-1.55	0.54	-2.89	3.84E-03	6.72E-02	Probable aquaporin PIP1-2
Ma01_g02560	ITC1587_Bchr1_P00659	30.62	-1.56	0.42	-3.69	2.27E-04	1.32E-02	UPF0392 protein RCOM_0530710-like
Ma04_g32660	ITC1587_Bchr4_P11045	27.96	-1.56	0.39	-3.95	7.87E-05	7.24E-03	putative ethylene-responsive transcription factor ERF053
Ma08_g14410	ITC1587_Bchr8_P23702	15.66	-1.56	0.49	-3.16	1.60E-03	4.16E-02	maspardin-like
Ma04_g27580	ITC1587_Bchr5_P13665*	17.36	-1.56	0.46	-3.39	7.00E-04	2.57E-02	E3 ubiquitin-protein ligase ATL4-like
Ma05_g11760	ITC1587_Bchr5_P12735	476.65	-1.56	0.37	-4.25	2.13E-05	3.34E-03	protein YLS9-like
Ma10_g25060	ITC1587_Bchr10_P31103	92.08	-1.56	0.52	-3.02	2.55E-03	5.32E-02	galactoside 2-alpha-L-fucosyltransferase-like
Ma00_g01120	ITC1587_Bchr10_P29197	11.28	-1.56	0.54	-2.87	4.11E-03	7.01E-02	uncharacterized LOC103999928
Ma10_g07640	ITC1587_Bchr10_P29525	54.29	-1.57	0.38	-4.12	3.87E-05	4.84E-03	uncharacterized LOC104000176
Ma04_g38240	ITC1587_Bchr4_P11511	20.28	-1.57	0.51	-3.06	2.21E-03	4.92E-02	probable LRR receptor-like serine/threonine-protein kinase At4g20940
Ma01_g03960	ITC1587_Bchr1_P00789*	22.87	-1.57	0.5	-3.15	1.66E-03	4.23E-02	Truncated NBS-LRR disease resistance protein (Fragment)
Ma06_g09960	ITC1587_Bchr6_P15527	34.97	-1.57	0.44	-3.57	3.54E-04	1.76E-02	uncharacterized LOC103987289
Ma08_g33410	ITC1587_Bchr8_P24918	28.91	-1.57	0.4	-3.91	9.21E-05	7.95E-03	arogenate dehydratase/prephenate dehydratase 6
Ma04_g26540	ITC1587_Bchr4_P10489	117	-1.57	0.56	-2.8	5.12E-03	7.82E-02	probable methyltransferase PMT27
Ma06_g16800	ITC1587_Bchr6_P16145	129.25	-1.57	0.4	-3.95	7.70E-05	7.13E-03	wall-associated receptor kinase 2-like
Ma09_g22990	ITC1587_Bchr4_P09411*	329.38	-1.58	0.33	-4.78	1.74E-06	7.49E-04	uncharacterized LOC103998683
Ma10_g28680	ITC1587_Bchr10_P31419	393.34	-1.58	0.54	-2.95	3.21E-03	6.14E-02	uncharacterized LOC103969480
Ma04_g21160	ITC1587_Bchr1_P00051	159.4	-1.58	0.53	-2.98	2.89E-03	5.80E-02	Hypothetical protein
Ma11_g22950	ITC1587_Bchr11_P34214	12.66	-1.58	0.55	-2.9	3.69E-03	6.58E-02	nuclear transcription factor Y subunit B-like
Ma06_g36050	ITC1587_Bchr6_P18279	73.72	-1.58	0.51	-3.14	1.71E-03	4.32E-02	scarecrow-like protein 9
Ma06_g25670	ITC1587_Bchr6_P17348	135.51	-1.59	0.49	-3.27	1.09E-03	3.31E-02	chitin-inducible gibberellin-responsive protein 1-like
Ma06_g20750	ITC1587_Bchr6_P16539	380.79	-1.59	0.39	-4.09	4.28E-05	5.17E-03	probable CCR4-associated factor 1 homolog 11

Ma02_g21450	ITC1587_Bchr2_P04835	20.58	-1.59	0.58	-2.74	6.22E-03	8.74E-02	2-aminoethanethiol dioxygenase-like
Ma06_g00820	ITC1587_Bchr11_P32662	91.15	-1.59	0.41	-3.84	1.25E-04	9.46E-03	protein YLS9-like
Ma08_g25980	ITC1587_Bchr8_P24250	26.83	-1.59	0.48	-3.28	1.03E-03	3.20E-02	uncharacterized LOC103996295
Ma03_g23850	ITC1587_Bchr3_P07550	476.97	-1.59	0.31	-5.12	3.13E-07	2.48E-04	zinc finger A20 and AN1 domain-containing stress-associated protein 5-like
Ma10_g19470	ITC1587_Bchr10_P30622	29.71	-1.59	0.33	-4.89	1.01E-06	5.33E-04	putative ethylene-responsive transcription factor ERF012
Ma07_g00070	ITC1587_Bchr7_P18556	18.3	-1.59	0.57	-2.78	5.41E-03	8.09E-02	chr7
Ma06_g11900	ITC1587_Bchr6_P15704*	38.17	-1.6	0.56	-2.86	4.26E-03	7.14E-02	uncharacterized LOC103987461
Ma07_g28030	ITC1587_Bchr7_P21366	222.01	-1.6	0.43	-3.68	2.32E-04	1.33E-02	uncharacterized LOC103992910
Ma02_g14570	ITC1587_Bchr2_P04223*	149.47	-1.6	0.42	-3.82	1.31E-04	9.80E-03	GATA transcription factor 12-like
Ma05_g28110	ITC1587_Bchr5_P14424	268.11	-1.6	0.4	-4.04	5.40E-05	5.91E-03	premnaspirodiene oxygenase-like
Ma03_g05070	ITC1587_Bchr3_P05618	684.13	-1.6	0.38	-4.17	3.06E-05	4.14E-03	probable xyloglucan endotransglucosylase/hydrolase protein 23
Ma04_g23080	ITC1587_Bchr1_P00304	285.93	-1.6	0.59	-2.71	6.68E-03	9.15E-02	uncharacterized LOC103976658
Ma11_g16450	ITC1587_Bchr11_P33645	191.12	-1.6	0.43	-3.74	1.88E-04	1.20E-02	probable xyloglucan endotransglucosylase/hydrolase protein 30
Ma06_g26320	ITC1587_Bchr1_P02200*	18.62	-1.6	0.57	-2.81	4.94E-03	7.66E-02	Protein DROOPING LEAF
Ma10_g10150	ITC1587_Bchr10_P29751	57.47	-1.61	0.43	-3.71	2.10E-04	1.26E-02	putative AP2 domain containing protein
Ma06_g34700	ITC1587_Bchr6_P18177	43.83	-1.61	0.53	-3.06	2.20E-03	4.91E-02	L-type lectin-domain containing receptor kinase VIII.1-like
Ma10_g04550	ITC1587_Bchr10_P29320	80.03	-1.61	0.55	-2.96	3.12E-03	6.02E-02	NAC-domain protein
Ma04_g36230	ITC1587_Bchr4_P11354	218.97	-1.61	0.55	-2.95	3.14E-03	6.04E-02	uncharacterized LOC103982392
Ma03_g25660	ITC1587_Bchr3_P07702	40.22	-1.61	0.52	-3.13	1.76E-03	4.37E-02	serine carboxypeptidase-like 33
Ma11_g12720	ITC1587_Bchr11_P33239	53.19	-1.62	0.49	-3.31	9.32E-04	3.02E-02	probable polygalacturonase
Ma09_g30270	ITC1587_Bchr9_P28239	716.18	-1.63	0.54	-2.99	2.80E-03	5.68E-02	protein YLS9
Ma02_g22840	ITC1587_Bchr2_P04965	36.27	-1.63	0.39	-4.22	2.45E-05	3.62E-03	ankyrin-3-like
Ma06_g29580	ITC1587_Bchr6_P17702	598.27	-1.63	0.44	-3.71	2.07E-04	1.26E-02	induced stolen tip protein TUB8-like
Ma07_g00230	ITC1587_Bchr7_P18571	21.42	-1.63	0.57	-2.87	4.13E-03	7.02E-02	uncharacterized LOC103991026
Ma11_g07880	ITC1587_Bchr11_P32453	38.48	-1.63	0.42	-3.84	1.22E-04	9.30E-03	uncharacterized LOC103970695
Ma02_g22630	ITC1587_Bchr2_P04946	15.4	-1.63	0.54	-3	2.68E-03	5.52E-02	U-box domain-containing protein 25-like
Ma08_g07650	ITC1587_Bchr8_P22116	299	-1.64	0.59	-2.79	5.35E-03	8.04E-02	momilactone A synthase-like

Ma01_g20070	ITC1587_Bchr1_P02362	185.47	-1.64	0.61	-2.67	7.67E-03	9.83E-02	pyrophosphate-energized vacuolar membrane proton pump
Ma11_g03740	ITC1587_Bchr11_P32042	76.99	-1.64	0.6	-2.75	6.04E-03	8.58E-02	transcription factor EGL1-like
Ma06_g25770	ITC1587_Bchr6_P17363*	26.71	-1.64	0.45	-3.64	2.77E-04	1.50E-02	neo-calmodulin-like
Ma09_g15420	ITC1587_Bchr9_P26419	858.73	-1.65	0.51	-3.21	1.35E-03	3.77E-02	putative lipoxygenase 5
Ma00_g00070	ITC1587_BchrUn_random_P37651	16.07	-1.65	0.59	-2.79	5.30E-03	8.00E-02	putative cytokinin-O-glucosyltransferase 3
Ma06_g21300	ITC1587_BchrUn_random_P39709	33.44	-1.65	0.5	-3.29	9.99E-04	3.16E-02	protein G1-like1
Ma07_g15760	ITC1587_Bchr7_P20063	33.01	-1.65	0.62	-2.66	7.77E-03	9.85E-02	uncharacterized LOC103991932
Ma10_g26200	ITC1587_Bchr10_P31194	12.51	-1.65	0.56	-2.97	3.00E-03	5.93E-02	GDSL esterase/lipase At4g10955-like
Ma08_g30910	ITC1587_Bchr8_P24713	87.1	-1.65	0.48	-3.46	5.40E-04	2.22E-02	U-box domain-containing protein 27-like
Ma08_g01170	ITC1587_Bchr8_P21575	23.51	-1.65	0.58	-2.86	4.17E-03	7.06E-02	uncharacterized LOC103994122
Ma04_g37730	ITC1587_Bchr4_P11473	13.09	-1.66	0.59	-2.83	4.66E-03	7.50E-02	uncharacterized LOC103982280
Ma03_g31250	ITC1587_Bchr3_P08161	100.98	-1.66	0.43	-3.87	1.07E-04	8.64E-03	cysteine-rich and transmembrane domain-containing protein A-like
Ma06_g34140	ITC1587_Bchr6_P18129	57.82	-1.66	0.48	-3.43	6.12E-04	2.38E-02	probably inactive leucine-rich repeat receptor-like protein kinase At3g28040
Ma08_g25890	ITC1587_Bchr8_P24240	177.15	-1.66	0.36	-4.64	3.45E-06	1.12E-03	U-box domain-containing protein 16-like
Ma02_g15740	ITC1587_Bchr2_P04319	11.31	-1.66	0.59	-2.83	4.63E-03	7.50E-02	glucan endo-1, 3-beta-glucosidase 1-like
Ma11_g21890	ITC1587_Bchr11_P34130	9.73	-1.67	0.62	-2.67	7.51E-03	9.72E-02	long-chain-alcohol oxidase FAO4A-like
Ma11_g12470	ITC1587_Bchr11_P33212	46.63	-1.67	0.62	-2.69	7.15E-03	9.50E-02	uncharacterized LOC103971440
Ma01_g03590	ITC1587_Bchr1_P00754	21.69	-1.67	0.53	-3.15	1.64E-03	4.21E-02	putative DNA-binding protein ESCAROLA
Ma04_g23660	ITC1587_Bchr1_P00367	12.53	-1.67	0.53	-3.16	1.56E-03	4.10E-02	Ethylene-responsive transcription factor ERF025
Ma10_g10470	ITC1587_Bchr10_P29779	607.24	-1.68	0.35	-4.74	2.15E-06	8.04E-04	U-box domain-containing protein 27-like
Ma10_g29700	ITC1587_Bchr10_P31513	14.98	-1.68	0.63	-2.68	7.39E-03	9.65E-02	vacuolar amino acid transporter 1
Ma02_g11500	ITC1587_Bchr2_P03969	96.42	-1.68	0.44	-3.82	1.35E-04	9.96E-03	uncharacterized LOC103975929
Ma01_g10930	ITC1587_Bchr1_P01548	13.57	-1.68	0.55	-3.04	2.38E-03	5.13E-02	dof zinc finger protein DOF1.4-like
Ma01_g18800	ITC1587_Bchr1_P02104	35.44	-1.68	0.54	-3.1	1.93E-03	4.56E-02	uncharacterized membrane protein At1g16860-like
Ma01_g04690	ITC1587_Bchr1_P00983	524.42	-1.68	0.54	-3.09	1.97E-03	4.64E-02	putative acid phosphatase 1

Ma04_g38270	ITC1587_Bchr4_P11514	25.73	-1.69	0.59	-2.86	4.19E-03	7.07E-02	patatin-like protein 3
Ma04_g28130	ITC1587_Bchr4_P10636	91.65	-1.69	0.5	-3.41	6.57E-04	2.48E-02	uncharacterized LOC103983075
Ma10_g05560	ITC1587_Bchr10_P28481	69.79	-1.69	0.46	-3.68	2.30E-04	1.32E-02	Glucan endo-1, 3-beta-glucosidase
Ma03_g04360	ITC1587_Bchr3_P05550	95.48	-1.69	0.54	-3.12	1.78E-03	4.40E-02	F-box/LRR-repeat protein 14-like
Ma01_g14880	ITC1587_Bchr7_P19725*	9.11	-1.69	0.58	-2.9	3.69E-03	6.58E-02	phospho-2-dehydro-3-deoxyheptonate aldolase 2
Ma03_g00420	ITC1587_Bchr3_P05201	17.75	-1.7	0.58	-2.94	3.28E-03	6.19E-02	probable WRKY transcription factor 11
Ma07_g03740	ITC1587_Bchr7_P18878	522.19	-1.7	0.35	-4.81	1.50E-06	6.91E-04	putative uncharacterized protein
Ma10_g09990	ITC1587_Bchr10_P29738	36.03	-1.7	0.54	-3.13	1.75E-03	4.36E-02	protein IRX15-LIKE-like
Ma06_g32650	ITC1587_Bchr6_P17991	9.02	-1.7	0.59	-2.88	3.94E-03	6.82E-02	structural maintenance of chromosomes protein 4
Ma04_g13850	ITC1587_Bchr3_P08133*	59.96	-1.7	0.49	-3.47	5.17E-04	2.16E-02	cycloartenol-C-24-methyltransferase 1-like
Ma03_g10830	ITC1587_Bchr3_P06161	31	-1.71	0.47	-3.65	2.58E-04	1.43E-02	probable inactive leucine-rich repeat receptor-like protein kinase At1g66830
Ma08_g16980	ITC1587_BchrUn_random_P37229	118.15	-1.71	0.52	-3.3	9.59E-04	3.07E-02	probable leucine-rich repeat receptor-like protein kinase At5g49770
Ma02_g12270	ITC1587_Bchr2_P04028	27.97	-1.71	0.41	-4.17	3.08E-05	4.14E-03	ELMO domain-containing protein A
Ma03_g31210	ITC1587_Bchr3_P08157	181.06	-1.71	0.42	-4.13	3.69E-05	4.69E-03	long chain base biosynthesis protein 2d-like
Ma01_g00720	ITC1587_Bchr1_P00918	398.52	-1.72	0.55	-3.12	1.84E-03	4.47E-02	putative probable WRKY transcription factor 40
Ma05_g01920	ITC1587_Bchr5_P11821	48.64	-1.72	0.48	-3.61	3.08E-04	1.60E-02	uncharacterized LOC103983571
Ma09_g16260	ITC1587_BchrUn_random_P36793	30.5	-1.72	0.64	-2.7	6.96E-03	9.35E-02	regulatory protein NPR5
Ma01_g14250	ITC1587_Bchr1_P01834	132.88	-1.72	0.51	-3.33	8.54E-04	2.89E-02	uncharacterized LOC103992216
Ma11_g01320	ITC1587_Bchr11_P31807	117.1	-1.73	0.57	-3.03	2.46E-03	5.22E-02	uncharacterized LOC103970138
Ma02_g08720	ITC1587_Bchr2_P03719	71.84	-1.74	0.44	-3.91	9.05E-05	7.92E-03	transcription factor TGA2-like
Ma09_g18570	ITC1587_Bchr9_P27174	173.17	-1.74	0.45	-3.89	1.02E-04	8.47E-03	zinc finger A20 and AN1 domain-containing stress-associated protein 1-like
Ma07_g15780	ITC1587_Bchr7_P20058	131.06	-1.74	0.52	-3.32	8.90E-04	2.96E-02	transcription factor bHLH35
Ma10_g04350	ITC1587_Bchr10_P29297	22.17	-1.74	0.62	-2.81	4.89E-03	7.64E-02	probable pectinesterase/pectinesterase inhibitor 51
Ma07_g19640	ITC1587_Bchr7_P20619	428.06	-1.74	0.47	-3.71	2.05E-04	1.26E-02	trans-cinnamate 4-monooxygenase-like

Ma04_g37620	ITC1587_Bchr4_P11465	101	-1.74	0.53	-3.3	9.57E-04	3.07E-02	GDSL esterase/lipase At5g45910-like
Ma04_g12450	ITC1587_Bchr4_P09516	25.73	-1.74	0.61	-2.87	4.12E-03	7.01E-02	protein TRI1-like
Ma10_g26710	ITC1587_Bchr10_P31239	15.68	-1.74	0.64	-2.74	6.09E-03	8.61E-02	caffeoylshikimate esterase-like
Ma09_g01870	ITC1587_Bchr9_P25239	33.19	-1.75	0.52	-3.34	8.32E-04	2.84E-02	GDSL esterase/lipase At3g26430-like
Ma00_g00080	ITC1587_BchrUn_random _P37652	12.42	-1.75	0.48	-3.65	2.62E-04	1.45E-02	scopoletin glucosyltransferase-like
Ma09_g11650	ITC1587_Bchr9_P26071	632.23	-1.75	0.54	-3.26	1.12E-03	3.36E-02	chitin-inducible gibberellin-responsive protein 1-like
Ma10_g08570	ITC1587_Bchr10_P29607	60.24	-1.75	0.53	-3.29	1.00E-03	3.16E-02	arginine decarboxylase-like
Ma03_g10770	ITC1587_Bchr3_P06155	38.25	-1.75	0.51	-3.46	5.42E-04	2.22E-02	protein TRANSPARENT TESTA 1-like
Ma10_g02930	ITC1587_Bchr8_P24130*	27.76	-1.75	0.56	-3.13	1.74E-03	4.36E-02	squamosa promoter-binding-like protein 12
Ma01_g12210	ITC1587_Bchr1_P01669	34.72	-1.76	0.57	-3.08	2.10E-03	4.78E-02	uncharacterized LOC103993920
Ma08_g12790		10	-1.76	0.58	-3.05	2.27E-03	5.01E-02	putative macrolide export ATP-binding/permease protein macB 3
Ma08_g27630	ITC1587_Bchr8_P24407	38.82	-1.76	0.5	-3.5	4.73E-04	2.05E-02	BURP domain-containing protein 12
Ma06_g34190	ITC1587_Bchr6_P18133	29.86	-1.76	0.55	-3.18	1.46E-03	3.95E-02	transcription factor RF2b-like
Ma01_g14190	ITC1587_Bchr1_P01830	38.54	-1.76	0.43	-4.11	3.92E-05	4.86E-03	glycosyltransferase protein
Ma09_g00900	ITC1587_Bchr9_P25141	49.13	-1.76	0.54	-3.25	1.16E-03	3.45E-02	BON1-associated protein 2-like
Ma05_g06810	ITC1587_Bchr5_P12290	458.56	-1.77	0.59	-2.99	2.83E-03	5.71E-02	uncharacterized LOC103984145
Ma05_g10420	ITC1587_Bchr4_P10977*	5.88	-1.77	0.6	-2.94	3.33E-03	6.22E-02	heavy metal-associated isoprenylated plant protein 26-like
Ma00_g03740	ITC1587_BchrUn_random _P37785	9.18	-1.77	0.61	-2.91	3.57E-03	6.47E-02	glyceraldehyde-3-phosphate dehydrogenase 2
Ma04_g15160	ITC1587_Bchr4_P09085*	6.85	-1.77	0.61	-2.89	3.86E-03	6.75E-02	Hypothetical protein
Ma08_g28460	ITC1587_Bchr8_P24485	92.67	-1.77	0.55	-3.22	1.28E-03	3.66E-02	putative fatty acid desaturase 2
Ma09_g30200	ITC1587_Bchr9_P28230	12.31	-1.78	0.61	-2.9	3.67E-03	6.57E-02	protein MKS1-like
Ma01_g04000	ITC1587_Bchr1_P00790	10.3	-1.78	0.55	-3.21	1.32E-03	3.72E-02	putative expressed protein
Ma05_g15100	ITC1587_Bchr3_P07135*	12.46	-1.78	0.62	-2.87	4.07E-03	6.97E-02	probable receptor-like protein kinase At1g33260
Ma01_g13890	ITC1587_Bchr1_P01806	10.85	-1.78	0.44	-4.07	4.70E-05	5.43E-03	dof zinc finger protein DOF1.7-like
Ma08_g21890	ITC1587_Bchr8_P23883	15.5	-1.78	0.54	-3.33	8.79E-04	2.93E-02	putative zinc finger and SCAN domain-containing protein 29

Ma07_g03730	ITC1587_Bchr7_P18876	88.3	-1.79	0.51	-3.49	4.86E-04	2.08E-02	protein ECERIFERUM 26-like
Ma08_g24770	ITC1587_Bchr8_P24145	34.87	-1.79	0.63	-2.83	4.70E-03	7.53E-02	probable sugar phosphate/phosphate translocator At5g25400
Ma10_g15060	ITC1587_Bchr10_P30243	68.18	-1.79	0.6	-2.97	3.00E-03	5.93E-02	proline-rich protein 2-like
Ma06_g34620	ITC1587_Bchr6_P18166	63.57	-1.79	0.57	-3.17	1.53E-03	4.08E-02	putative serine/threonine-protein kinase-like protein CCR3
Ma09_g08240	ITC1587_Bchr9_P25769	70.27	-1.79	0.61	-2.93	3.39E-03	6.27E-02	patellin-3-like
Ma02_g04770	ITC1587_BchrUn_random _P36847*	21.48	-1.79	0.57	-3.15	1.64E-03	4.22E-02	secretory carrier-associated membrane protein 1-like
Ma01_g14590	ITC1587_Bchr1_P01870	49.43	-1.79	0.42	-4.22	2.39E-05	3.57E-03	uncharacterized LOC103991867
Ma05_g17850	ITC1587_Bchr6_P16802*	189.44	-1.79	0.65	-2.76	5.78E-03	8.37E-02	chitinase 6-like
Ma05_g21690	ITC1587_Bchr5_P13797	132.39	-1.8	0.57	-3.17	1.55E-03	4.09E-02	wall-associated receptor kinase 3-like
Ma06_g15240	ITC1587_Bchr6_P16002	5.03	-1.8	0.59	-3.04	2.41E-03	5.15E-02	transcription factor bHLH144-like
Ma10_g26510	ITC1587_Bchr10_P31221	19.39	-1.8	0.62	-2.89	3.85E-03	6.74E-02	uncharacterized LOC103969300
Ma07_g18640	ITC1587_Bchr7_P20722*	175.88	-1.8	0.59	-3.04	2.39E-03	5.14E-02	alpha-humulene synthase-like
Ma11_g09260	ITC1587_Bchr8_P23045*	42.14	-1.8	0.47	-3.82	1.33E-04	9.90E-03	probable protein S-acyltransferase 6
Ma10_g03120	ITC1587_Bchr10_P28716	53.5	-1.8	0.65	-2.78	5.40E-03	8.09E-02	Peroxidase 65
Ma01_g01620	ITC1587_Bchr1_P00581	89.88	-1.81	0.63	-2.87	4.05E-03	6.94E-02	protein NRT1/ PTR FAMILY 7.3-like
Ma06_g30170	ITC1587_Bchr6_P17763	241.14	-1.81	0.39	-4.6	4.32E-06	1.26E-03	lipoxygenase 6
Ma01_g14820		66.74	-1.81	0.42	-4.34	1.42E-05	2.62E-03	lysine-rich arabinogalactan protein 19-like
Ma08_g21900	ITC1587_Bchr8_P23884	129.56	-1.81	0.47	-3.86	1.14E-04	8.85E-03	omega-3 fatty acid desaturase
Ma06_g03700	ITC1587_Bchr6_P14954	67.72	-1.82	0.39	-4.64	3.43E-06	1.12E-03	uncharacterized LOC103986701
Ma01_g11840	ITC1587_Bchr1_P01634	77.87	-1.82	0.46	-3.99	6.55E-05	6.68E-03	uncharacterized LOC103994308
Ma05_g02360	ITC1587_Bchr5_P11861	50.25	-1.83	0.56	-3.26	1.13E-03	3.39E-02	BTB/POZ domain-containing protein At1g30440-like
Ma04_g27210	ITC1587_Bchr7_P19002*	24.3	-1.83	0.6	-3.07	2.16E-03	4.86E-02	afadin- and alpha-actinin-binding protein-like
Ma04_g29240	ITC1587_Bchr4_P10741	485.04	-1.83	0.63	-2.88	3.93E-03	6.82E-02	5-methyltetrahydropteroyltriglutamate--homocysteine methyltransferase 2
Ma02_g00280	ITC1587_BchrUn_random _P35829	30.66	-1.83	0.58	-3.18	1.49E-03	3.99E-02	transcription factor MYB108-like
Ma02_g21380	ITC1587_Bchr2_P04827	12.54	-1.83	0.66	-2.8	5.17E-03	7.88E-02	60S ribosomal protein L18a-like protein
Ma10_g04500	ITC1587_Bchr10_P29315	28.19	-1.83	0.43	-4.26	2.04E-05	3.27E-03	probable receptor protein kinase TMK1

Ma02_g13100	ITC1587_Bchr2_P04098	54.78	-1.83	0.68	-2.69	7.23E-03	9.53E-02	subtilisin-like protease
Ma08_g24250	ITC1587_Bchr8_P24105*	12.32	-1.84	0.66	-2.77	5.67E-03	8.27E-02	alpha-trehalose-phosphate synthase [UDP-forming] 9
Ma07_g18650	ITC1587_Bchr7_P20722*	34.82	-1.84	0.67	-2.73	6.29E-03	8.78E-02	putative delta-cadinene synthase
Ma01_g20780	ITC1587_Bchr1_P02581	89.02	-1.85	0.62	-2.96	3.03E-03	5.94E-02	squalene monooxygenase-like
Ma08_g09070	ITC1587_Bchr8_P22259	16.56	-1.85	0.66	-2.81	4.97E-03	7.67E-02	mini zinc finger protein 2-like
Ma01_g11850	ITC1587_Bchr1_P01635	60.7	-1.85	0.45	-4.08	4.44E-05	5.24E-03	chaperone protein dnaJ 11
Ma07_g13600	ITC1587_Bchr7_P19822	17.48	-1.85	0.64	-2.91	3.56E-03	6.47E-02	uncharacterized LOC103991602
Ma11_g16170	ITC1587_Bchr11_P31736*	56.3	-1.86	0.4	-4.62	3.83E-06	1.20E-03	Hydrophobic protein LTI6B
Ma11_g22280	ITC1587_Bchr11_P34164*	39.8	-1.86	0.46	-4.02	5.87E-05	6.29E-03	zinc finger protein ZAT11-like
Ma06_g38910	ITC1587_Bchr6_P18537	490.18	-1.86	0.53	-3.5	4.65E-04	2.05E-02	phospholipase A1-Ibeta2
Ma02_g23480	ITC1587_Bchr2_P05019	35.31	-1.87	0.55	-3.39	6.91E-04	2.54E-02	probable inactive receptor kinase RLK902
Ma06_g07330	ITC1587_Bchr6_P15287	453.65	-1.87	0.61	-3.05	2.27E-03	5.01E-02	probable WRKY transcription factor 41
Ma10_g15290	ITC1587_Bchr10_P30264	138.04	-1.87	0.66	-2.83	4.67E-03	7.51E-02	protein ASPARTIC PROTEASE IN GUARD CELL 2-like
Ma07_g04120	ITC1587_Bchr7_P18910	56.75	-1.87	0.69	-2.71	6.77E-03	9.21E-02	uncharacterized LOC103990689
Ma02_g18610	ITC1587_Bchr2_P04578	33.66	-1.87	0.66	-2.84	4.56E-03	7.42E-02	protein NRT1/ PTR FAMILY 4.6-like
Ma11_g05230	ITC1587_BchrUn_random_P36126*	6.68	-1.87	0.67	-2.79	5.29E-03	7.99E-02	ethylene-responsive transcription factor
Ma08_g11550	ITC1587_Bchr8_P22489	14.76	-1.87	0.7	-2.66	7.80E-03	9.88E-02	uncharacterized LOC103993226
Ma11_g04990	ITC1587_Bchr11_P32164	10.88	-1.88	0.68	-2.78	5.50E-03	8.17E-02	uncharacterized LOC103970452
Ma02_g07200	ITC1587_Bchr2_P03576	50.65	-1.88	0.66	-2.85	4.34E-03	7.21E-02	F-box protein SKIP2-like
Ma05_g31460	ITC1587_Bchr5_P14728	182.54	-1.88	0.6	-3.13	1.76E-03	4.37E-02	probable protein phosphatase 2C 4
Ma00_g01700	ITC1587_BchrUn_random_P38289	10.95	-1.89	0.59	-3.22	1.26E-03	3.62E-02	uncharacterized LOC103974666
Ma06_g24010	ITC1587_Bchr6_P16870	102.81	-1.9	0.44	-4.27	1.92E-05	3.14E-03	uncharacterized LOC103988717
Ma11_g24260	ITC1587_Bchr11_P34322	49.75	-1.9	0.48	-3.93	8.40E-05	7.59E-03	calcium-binding protein PBP1-like
Ma10_g07280	ITC1587_Bchr10_P29471	33.33	-1.9	0.46	-4.08	4.42E-05	5.24E-03	exocyst complex component EXO70B1-like
Ma07_g19650	ITC1587_Bchr7_P20620	16.65	-1.9	0.5	-3.81	1.37E-04	1.00E-02	Hypothetical protein
Ma11_g19050	ITC1587_Bchr11_P33872	265.83	-1.91	0.44	-4.37	1.24E-05	2.44E-03	uncharacterized LOC103971974
Ma09_g12720	ITC1587_Bchr10_P31604*	225.65	-1.91	0.31	-6.1	1.08E-09	5.78E-06	vegetative cell wall protein gp1-like

Ma07_g25860	ITC1587_Bchr3_P08336*	13.26	-1.91	0.59	-3.23	1.25E-03	3.61E-02	protein trichome birefringence-like 38
Ma06_g07960	ITC1587_Bchr6_P15344	17.16	-1.91	0.6	-3.21	1.31E-03	3.70E-02	early nodulin-like protein 1
Ma04_g23650	ITC1587_Bchr1_P00366	21.76	-1.91	0.68	-2.8	5.04E-03	7.73E-02	uncharacterized LOC103976091
Ma06_g34480	ITC1587_Bchr6_P18156	11.44	-1.91	0.64	-2.97	2.94E-03	5.87E-02	protein G1-like7
Ma11_g09090	ITC1587_Bchr11_P32572	9.67	-1.91	0.68	-2.82	4.86E-03	7.62E-02	glucan endo-1, 3-beta-glucosidase 3-like
Ma10_g23400	ITC1587_Bchr10_P30956	341.14	-1.91	0.35	-5.5	3.84E-08	6.82E-05	probable xyloglucan endotransglucosylase/hydrolase protein 25
Ma07_g20720	ITC1587_Bchr7_P20722*	122.76	-1.92	0.55	-3.5	4.74E-04	2.05E-02	alpha-humulene synthase-like
Ma05_g26320	ITC1587_Bchr5_P14261	135.38	-1.92	0.58	-3.29	9.90E-04	3.14E-02	chromosome-associated kinesin KIF4A-like
Ma08_g13180	ITC1587_Bchr2_P04061*	50.68	-1.92	0.67	-2.88	3.92E-03	6.82E-02	fatty acid 2-hydroxylase 1-like
Ma06_g15250	ITC1587_Bchr6_P16004	38.82	-1.92	0.51	-3.74	1.85E-04	1.19E-02	GDSL esterase/lipase At4g10955-like
Ma06_g12550	ITC1587_Bchr6_P15766	99.22	-1.92	0.58	-3.29	1.00E-03	3.16E-02	UDP-glucose 6-dehydrogenase 5-like
Ma10_g27530	ITC1587_Bchr10_P31316*	178.46	-1.92	0.56	-3.41	6.43E-04	2.47E-02	probable inositol oxygenase
Ma05_g31300	ITC1587_Bchr5_P14710	875.8	-1.92	0.38	-5.09	3.61E-07	2.65E-04	nematode resistance protein-like HSPRO2
Ma06_g01950	ITC1587_Bchr6_P14806	30.96	-1.93	0.5	-3.89	1.02E-04	8.47E-03	putative ethylene-responsive transcription factor 4
Ma07_g27780	ITC1587_Bchr7_P21345*	17.24	-1.93	0.48	-4	6.27E-05	6.53E-03	LOB domain-containing protein 38-like
Ma03_g08750	ITC1587_Bchr3_P05974	7.42	-1.93	0.69	-2.82	4.88E-03	7.63E-02	uncharacterized LOC103977707
Ma01_g19330	ITC1587_Bchr1_P02334	58.4	-1.93	0.37	-5.24	1.57E-07	1.68E-04	Hypothetical protein
Ma05_g26980	ITC1587_Bchr7_P19678*	15.82	-1.93	0.41	-4.76	1.95E-06	7.90E-04	HIPL1 protein-like
Ma07_g21120	ITC1587_Bchr7_P20767	31.69	-1.94	0.72	-2.68	7.43E-03	9.68E-02	xylosyltransferase 1-like
Ma01_g01030	ITC1587_Bchr1_P00942	67.67	-1.94	0.51	-3.83	1.28E-04	9.63E-03	probable xyloglucan endotransglucosylase/hydrolase protein 7
Ma01_g10570	ITC1587_Bchr4_P08875*	9.86	-1.94	0.73	-2.67	7.52E-03	9.72E-02	Tubulin beta-1 chain
Ma11_g14440	ITC1587_Bchr11_P33439	259.52	-1.95	0.46	-4.2	2.66E-05	3.77E-03	COBRA-like protein 7
Ma04_g24510	ITC1587_Bchr1_P00450	272.92	-1.96	0.66	-2.97	2.95E-03	5.88E-02	3-ketoacyl-CoA synthase 17
Ma04_g05470	ITC1587_Bchr4_P08872	17.11	-1.97	0.57	-3.43	6.06E-04	2.38E-02	25.3 kDa vesicle transport protein
Ma08_g09300	ITC1587_Bchr8_P22276	328.43	-1.97	0.62	-3.16	1.57E-03	4.10E-02	basic 7S globulin-like
Ma04_g01500	ITC1587_Bchr4_P08521	16.41	-1.97	0.73	-2.71	6.71E-03	9.16E-02	glycine-rich protein-like
Ma08_g28380	ITC1587_Bchr8_P24478	8.93	-1.97	0.64	-3.11	1.90E-03	4.54E-02	probable WRKY transcription factor 65

Ma06_g14300	ITC1587_Bchr6_P15917	6.65	-1.98	0.72	-2.75	5.93E-03	8.50E-02	cell wall / vacuolar inhibitor of fructosidase 2-like
Ma09_g26110	ITC1587_Bchr9_P27865*	55.82	-1.98	0.58	-3.41	6.40E-04	2.46E-02	protein tesmin/TSO1-like CXC 2
Ma10_g31200	ITC1587_Bchr10_P31652	4.52	-1.98	0.71	-2.77	5.54E-03	8.19E-02	F-box/kelch-repeat protein At1g57790-like
Ma04_g00530	ITC1587_Bchr4_P08428	40.81	-1.98	0.62	-3.21	1.33E-03	3.73E-02	homeobox-leucine zipper protein ATHB-13-like
Ma07_g25430	ITC1587_Bchr7_P21152	1118.13	-1.98	0.37	-5.3	1.13E-07	1.37E-04	expressed protein
Ma01_g16170	ITC1587_Bchr1_P02014	9.24	-1.99	0.61	-3.28	1.05E-03	3.24E-02	CASP-like protein 2B1
Ma08_g09890	ITC1587_Bchr8_P22325	286.08	-1.99	0.41	-4.88	1.06E-06	5.43E-04	ingression protein fic1-like
Ma06_g08130	ITC1587_Bchr9_P25209*	42.7	-1.99	0.55	-3.6	3.17E-04	1.63E-02	serine hydroxymethyltransferase 4
Ma10_g24580	ITC1587_Bchr10_P31060	164.9	-1.99	0.49	-4.11	3.93E-05	4.86E-03	uncharacterized LOC103969136
Ma10_g14480	ITC1587_Bchr10_P30187	12.44	-2	0.67	-2.97	3.01E-03	5.93E-02	GDSL esterase/lipase At5g45910-like
Ma07_g29040	ITC1587_Bchr7_P21415	11.47	-2	0.74	-2.71	6.64E-03	9.10E-02	uncharacterized LOC103993063
Ma05_g24120	ITC1587_Bchr6_P16210*	21.93	-2	0.65	-3.06	2.18E-03	4.89E-02	Tubulin beta-1 chain
Ma05_g13480	ITC1587_Bchr5_P12867	26.63	-2	0.74	-2.71	6.64E-03	9.10E-02	CPuORF23 - conserved peptide uORF-containing transcript
Ma08_g21680	ITC1587_Bchr8_P23864	226.51	-2	0.61	-3.26	1.11E-03	3.35E-02	UDP-glucuronic acid decarboxylase 6-like
Ma09_g15500	ITC1587_Bchr9_P26428*	10.51	-2.02	0.73	-2.75	6.00E-03	8.55E-02	MOB kinase activator-like 1
Ma04_g06770	ITC1587_Bchr4_P08977	34.26	-2.02	0.41	-4.96	6.91E-07	4.28E-04	zinc finger protein 6-like
Ma03_g23970	ITC1587_Bchr3_P07561	14.92	-2.02	0.6	-3.38	7.23E-04	2.61E-02	nucleobase-ascorbate transporter 12-like
Ma03_g05090	ITC1587_Bchr3_P05620	576.13	-2.02	0.71	-2.85	4.39E-03	7.26E-02	probable xyloglucan endotransglucosylase/hydrolase protein 23
Ma10_g19240	ITC1587_Bchr10_P30601	11.85	-2.03	0.68	-2.99	2.83E-03	5.71E-02	GEM-like protein 1
Ma05_g25220	ITC1587_Bchr5_P14165	19.88	-2.03	0.67	-3.03	2.43E-03	5.18E-02	uncharacterized LOC103985474
Ma01_g07950	ITC1587_Bchr1_P01282	490.81	-2.03	0.4	-5.15	2.66E-07	2.29E-04	glycine dehydrogenase (decarboxylating)
Ma06_g18500	ITC1587_Bchr6_P16306	88.45	-2.04	0.65	-3.12	1.80E-03	4.43E-02	mitogen-activated protein kinase kinase kinase A-like
Ma08_g30800	ITC1587_Bchr8_P24700	192.92	-2.04	0.73	-2.81	4.91E-03	7.64E-02	uncharacterized LOC103995857
Ma02_g21760	ITC1587_Bchr2_P04866	12.9	-2.05	0.72	-2.82	4.76E-03	7.58E-02	myb-related protein 3R-1-like
Ma02_g13320	ITC1587_Bchr2_P04118	6.87	-2.05	0.73	-2.82	4.82E-03	7.60E-02	formin-like protein 1
Ma07_g20660	ITC1587_Bchr7_P20722	450.18	-2.05	0.64	-3.19	1.41E-03	3.89E-02	alpha-humulene synthase-like

Ma06_g16290	ITC1587_Bchr6_P16104	60.73	-2.05	0.55	-3.72	2.01E-04	1.26E-02	putative dehydration-responsive element-binding protein 3
Ma09_g01600	ITC1587_Bchr9_P25210	264.48	-2.05	0.63	-3.24	1.20E-03	3.53E-02	adenosylhomocysteinase
Ma01_g17580	ITC1587_Bchr1_P02237	10.81	-2.06	0.62	-3.3	9.56E-04	3.07E-02	uncharacterized LOC103996892
Ma09_g07320	ITC1587_Bchr9_P25694	761.71	-2.06	0.57	-3.58	3.39E-04	1.71E-02	notchless protein homolog 1-like
Ma03_g22260	ITC1587_Bchr3_P07415	7.09	-2.06	0.71	-2.92	3.51E-03	6.44E-02	putative pumilio homolog 7
Ma05_g05640	ITC1587_Bchr5_P12187	16.32	-2.06	0.68	-3.04	2.33E-03	5.07E-02	HMG-Y-related protein A-like
Ma03_g19730	ITC1587_Bchr3_P07191	14.41	-2.06	0.6	-3.43	5.98E-04	2.36E-02	mitogen-activated protein kinase kinase kinase 2-like
Ma08_g01990	ITC1587_Bchr8_P21656	39.55	-2.06	0.58	-3.56	3.68E-04	1.81E-02	probable serine/threonine-protein kinase NAK
Ma10_g28610	ITC1587_Bchr10_P31413	35.15	-2.06	0.68	-3.04	2.36E-03	5.12E-02	uncharacterized LOC103969475
Ma10_g24570	ITC1587_Bchr10_P31059	378.38	-2.06	0.63	-3.29	1.00E-03	3.16E-02	uncharacterized LOC103969135
Ma03_g03460	ITC1587_Bchr3_P05477	13.07	-2.06	0.57	-3.63	2.79E-04	1.50E-02	gibberellic acid methyltransferase 2-like
Ma06_g06580	ITC1587_Bchr6_P15224	25.58	-2.07	0.6	-3.43	6.02E-04	2.37E-02	leucine-rich repeat extensin-like protein 4
Ma09_g30530	ITC1587_Bchr9_P28260	25.01	-2.07	0.74	-2.78	5.52E-03	8.17E-02	uncharacterized LOC103999553
Ma08_g11830	ITC1587_Bchr8_P22517	60.85	-2.07	0.42	-4.97	6.64E-07	4.21E-04	transcription factor RF2a-like
Ma08_g01800	ITC1587_Bchr3_P06499*	24.3	-2.07	0.64	-3.24	1.18E-03	3.47E-02	extensin-like
Ma08_g19270	ITC1587_Bchr8_P23003	34.83	-2.08	0.6	-3.47	5.11E-04	2.15E-02	Actin-7
Ma01_g05960	ITC1587_Bchr1_P01101	8.37	-2.08	0.61	-3.42	6.22E-04	2.41E-02	uncharacterized LOC103987678
Ma05_g16120	ITC1587_BchrUn_random_P35166	765.44	-2.08	0.54	-3.86	1.12E-04	8.82E-03	E3 ubiquitin-protein ligase RHA1B-like
Ma10_g04690	ITC1587_Bchr10_P29331	14.13	-2.08	0.63	-3.28	1.05E-03	3.24E-02	sugar carrier protein C-like
Ma07_g12510	ITC1587_Bchr7_P19721	196.68	-2.08	0.42	-4.92	8.60E-07	4.99E-04	uncharacterized LOC103991516
Ma01_g11650	ITC1587_Bchr1_P01615	14.21	-2.08	0.5	-4.19	2.81E-05	3.90E-03	alpha-trehalose-phosphate synthase [UDP-forming] 9
Ma07_g08040	ITC1587_Bchr7_P19282	157.59	-2.08	0.51	-4.05	5.19E-05	5.71E-03	uncharacterized LOC103990325
Ma10_g17080	ITC1587_Bchr10_P30419	12.03	-2.08	0.68	-3.08	2.06E-03	4.74E-02	induced stolen tip protein TUB8-like
Ma01_g21800	ITC1587_BchrUn_random_P35520	98.15	-2.08	0.62	-3.35	8.21E-04	2.82E-02	AP2/ERF and B3 domain-containing transcription repressor RAV2-like
Ma03_g27360	ITC1587_Bchr3_P07857	35.15	-2.08	0.69	-3.02	2.51E-03	5.27E-02	transcription factor bHLH25-like
Ma04_g32180	ITC1587_Bchr4_P11011	9.95	-2.09	0.63	-3.34	8.39E-04	2.85E-02	3-ketoacyl-CoA synthase 1

Ma09_g08840	ITC1587_Bchr9_P25822	60.36	-2.09	0.38	-5.46	4.69E-08	7.35E-05	calmodulin-like protein 7
Ma06_g12070	ITC1587_Bchr6_P15720	39.09	-2.09	0.48	-4.36	1.30E-05	2.49E-03	uncharacterized LOC103987475
Ma06_g26940	ITC1587_Bchr6_P17458	36.66	-2.1	0.7	-3.02	2.54E-03	5.32E-02	probable inactive receptor kinase At5g58300
Ma05_g30370	ITC1587_Bchr5_P14637	14.54	-2.1	0.7	-3.00	2.72E-03	5.59E-02	uncharacterized LOC103986199
Ma06_g34080	ITC1587_Bchr6_P18127	323.65	-2.11	0.48	-4.39	1.14E-05	2.39E-03	uncharacterized LOC103989668
Ma05_g01720	ITC1587_Bchr5_P11806	17.75	-2.11	0.66	-3.19	1.45E-03	3.93E-02	uncharacterized LOC103983586
Ma05_g29360	ITC1587_Bchr5_P14543	34.59	-2.11	0.67	-3.13	1.75E-03	4.37E-02	respiratory burst oxidase homolog protein B-like
Ma06_g20990	ITC1587_Bchr9_P28222*	14.8	-2.11	0.68	-3.12	1.83E-03	4.46E-02	probable receptor-like protein kinase At1g49730
Ma03_g11590	ITC1587_Bchr3_P06226	22.57	-2.11	0.64	-3.32	9.16E-04	2.99E-02	protein TRANSPARENT TESTA 1-like
Ma08_g21570	ITC1587_Bchr8_P23856	1113.22	-2.12	0.39	-5.38	7.50E-08	9.52E-05	E3 ubiquitin-protein ligase RING1-like
Ma02_g15330	ITC1587_Bchr2_P04288	8.5	-2.12	0.67	-3.17	1.54E-03	4.08E-02	uncharacterized LOC103975629
Ma10_g27820	ITC1587_Bchr10_P31338	26.74	-2.12	0.58	-3.64	2.72E-04	1.48E-02	Cationic peroxidase 1
Ma06_g10030	ITC1587_Bchr6_P15534	33.46	-2.12	0.67	-3.16	1.56E-03	4.10E-02	Tubulin beta-1 chain
Ma06_g14930	ITC1587_Bchr6_P15972	6.74	-2.12	0.77	-2.75	5.89E-03	8.46E-02	uncharacterized LOC103987724
Ma01_g14830	ITC1587_Bchr1_P01890	14.03	-2.13	0.66	-3.21	1.33E-03	3.73E-02	homeobox-leucine zipper protein HOX13-like
Ma05_g00260	ITC1587_Bchr5_P11689	6.95	-2.13	0.66	-3.25	1.15E-03	3.44E-02	uncharacterized LOC103983773
Ma04_g13810	ITC1587_Bchr4_P09632	68.53	-2.13	0.45	-4.78	1.71E-06	7.49E-04	LOB domain-containing protein 41-like
Ma07_g20440	ITC1587_Bchr7_P20706	13.86	-2.14	0.78	-2.73	6.42E-03	8.89E-02	serine/threonine-protein kinase-like protein At1g28390
Ma08_g32810	ITC1587_Bchr8_P24870	9.19	-2.14	0.78	-2.73	6.35E-03	8.84E-02	uncharacterized LOC103995689
Ma04_g36860	ITC1587_Bchr4_P11402	28.2	-2.14	0.7	-3.05	2.31E-03	5.05E-02	GATA transcription factor 4-like
Ma10_g03840	ITC1587_Bchr10_P29239	139.89	-2.15	0.8	-2.69	7.11E-03	9.47E-02	BTB/POZ and TAZ domain-containing protein 1-like
Ma06_g36150	ITC1587_Bchr10_P29144*	32.9	-2.16	0.7	-3.1	1.94E-03	4.58E-02	uncharacterized LOC103990158
Ma04_g29180	ITC1587_Bchr4_P10735	19.17	-2.16	0.67	-3.2	1.37E-03	3.80E-02	uncharacterized LOC103982976
Ma08_g32110	ITC1587_Bchr1_P02060*	5.2	-2.16	0.73	-2.95	3.13E-03	6.04E-02	pathogenesis-related protein PR-4-like
Ma03_g21680	ITC1587_Bchr3_P07364	12.52	-2.16	0.78	-2.79	5.33E-03	8.01E-02	1-deoxy-D-xylulose 5-phosphate reductoisomerase
Ma07_g18620	ITC1587_Bchr7_P20728*	124.41	-2.17	0.54	-3.98	7.00E-05	6.80E-03	alpha-humulene synthase-like

Ma06_g09240	ITC1587_Bchr6_P15464	194.08	-2.17	0.51	-4.29	1.83E-05	3.02E-03	mitogen-activated protein kinase kinase kinase NPK1-like
Ma05_g15830	ITC1587_Bchr4_P08839*	13.77	-2.17	0.8	-2.72	6.53E-03	9.01E-02	GDSL esterase/lipase At5g55050-like
Ma09_g23090	ITC1587_Bchr9_P27563	12.95	-2.17	0.63	-3.43	6.11E-04	2.38E-02	probable LRR receptor-like serine/threonine-protein kinase At4g37250
Ma03_g06610	ITC1587_Bchr3_P05755	15.28	-2.18	0.67	-3.24	1.19E-03	3.49E-02	transcription factor bHLH94-like
Ma11_g16060	ITC1587_Bchr11_P33612	85.1	-2.18	0.7	-3.1	1.91E-03	4.54E-02	transcription factor EGL1-like
Ma08_g09130	ITC1587_Bchr8_P22265	33.58	-2.19	0.59	-3.7	2.16E-04	1.29E-02	protein SCARECROW 2-like
Ma03_g17200	ITC1587_Bchr3_P06857	53.62	-2.19	0.58	-3.75	1.77E-04	1.18E-02	probable GABA transporter 2
Ma06_g13360	ITC1587_Bchr6_P15836	5.99	-2.19	0.79	-2.78	5.49E-03	8.17E-02	putative UPF0481 protein At3g47200
Ma10_g19760	ITC1587_Bchr10_P30644	12.59	-2.19	0.77	-2.83	4.65E-03	7.50E-02	uncharacterized LOC103968711
Ma03_g01210	ITC1587_Bchr3_P05275	5.95	-2.2	0.71	-3.11	1.88E-03	4.53E-02	uncharacterized LOC103977214
Ma04_g32260	ITC1587_Bchr4_P11017	78.45	-2.2	0.61	-3.58	3.46E-04	1.73E-02	proline-rich receptor-like protein kinase PERK3
Ma06_g06940	ITC1587_Bchr4_P11433*	35.37	-2.21	0.61	-3.60	3.15E-04	1.63E-02	protein argonaute PNH1-like
Ma05_g00400	ITC1587_Bchr5_P11700	142.31	-2.21	0.73	-3.03	2.46E-03	5.22E-02	CASP-like protein MA4_106O17.50
Ma08_g23400	ITC1587_Bchr8_P24028	89.17	-2.21	0.55	-4.05	5.18E-05	5.71E-03	putative lipoxygenase 5
Ma05_g18660	ITC1587_Bchr5_P13340	7.96	-2.21	0.76	-2.90	3.78E-03	6.66E-02	WRKY transcription factor 6
Ma04_g13980	ITC1587_Bchr4_P09651	96.3	-2.21	0.59	-3.77	1.66E-04	1.13E-02	polyol transporter 5-like
Ma04_g05850	ITC1587_Bchr4_P08907	6.93	-2.21	0.82	-2.70	7.00E-03	9.38E-02	uncharacterized LOC103980716
Ma06_g33880	ITC1587_Bchr5_P14610*	17.35	-2.22	0.67	-3.31	9.23E-04	3.00E-02	putative uncharacterized protein Sb03g037890
Ma04_g32410	ITC1587_Bchr4_P11026	15.36	-2.22	0.8	-2.77	5.68E-03	8.27E-02	uncharacterized LOC103982698
Ma08_g12360	ITC1587_Bchr8_P22568	5.76	-2.22	0.79	-2.82	4.73E-03	7.55E-02	helicase swr-1
Ma05_g24190	ITC1587_Bchr5_P14071	13.83	-2.23	0.71	-3.15	1.61E-03	4.18E-02	uncharacterized LOC103985562
Ma06_g28770	ITC1587_Bchr6_P17631*	14.88	-2.24	0.77	-2.93	3.43E-03	6.31E-02	Hypothetical protein
Ma06_g35800	ITC1587_Bchr6_P18260	11.63	-2.24	0.67	-3.35	7.96E-04	2.78E-02	mitochondrial uncoupling protein 1-like
Ma03_g02770	ITC1587_Bchr3_P05417	10.1	-2.24	0.67	-3.35	8.00E-04	2.78E-02	Actin
Ma09_g20070	ITC1587_Bchr9_P26831	185.12	-2.25	0.72	-3.14	1.69E-03	4.29E-02	F-box protein SKIP2-like
Ma04_g25460	ITC1587_Bchr1_P00542	43.56	-2.25	0.48	-4.68	2.81E-06	9.82E-04	uncharacterized LOC103999827
Ma02_g05360	ITC1587_Bchr2_P03409*	21.43	-2.25	0.65	-3.45	5.59E-04	2.26E-02	chaperone protein dnaJ 11

Ma07_g06000	ITC1587_Bchr7_P19081	452.98	-2.27	0.52	-4.35	1.34E-05	2.54E-03	putative dehydration-responsive element-binding protein 1D
Ma02_g05470	ITC1587_Bchr2_P03417	12.64	-2.28	0.49	-4.63	3.64E-06	1.16E-03	putative UPF0496 protein 2
Ma04_g29120	ITC1587_Bchr4_P10730	19.89	-2.28	0.66	-3.44	5.77E-04	2.30E-02	calmodulin-like
Ma09_g31060	ITC1587_Bchr9_P28301	19.71	-2.28	0.56	-4.10	4.12E-05	5.02E-03	serine/arginine repetitive matrix protein 2-like
Ma06_g32480	ITC1587_Bchr6_P17975	19.88	-2.29	0.57	-3.99	6.60E-05	6.69E-03	isoflavone 2'-hydroxylase-like
Ma10_g25810	ITC1587_Bchr10_P31160	15.41	-2.29	0.57	-4.05	5.03E-05	5.66E-03	ethylene-responsive transcription factor ERF017-like
Ma07_g18610	ITC1587_Bchr7_P20722*	175.19	-2.31	0.68	-3.41	6.51E-04	2.47E-02	beta-eudesmol synthase-like
Ma06_g36260	ITC1587_Bchr6_P18294	17.57	-2.31	0.86	-2.67	7.61E-03	9.77E-02	zinc finger protein ZAT8-like
Ma11_g09830	ITC1587_Bchr11_P32948	44.65	-2.31	0.64	-3.61	3.04E-04	1.59E-02	Actin-7
Ma01_g09530	ITC1587_Bchr1_P01418	35.3	-2.31	0.52	-4.41	1.04E-05	2.27E-03	ARMADILLO BTB ARABIDOPSIS PROTEIN 1-like
Ma06_g03940	ITC1587_Bchr6_P14978	12.68	-2.32	0.73	-3.20	1.37E-03	3.80E-02	peamaclein-like
Ma11_g18310	ITC1587_Bchr11_P33807	78.64	-2.32	0.85	-2.74	6.18E-03	8.70E-02	Probable inactive receptor kinase At5g58300
Ma08_g10980	ITC1587_Bchr8_P22436	54.16	-2.34	0.82	-2.87	4.11E-03	7.01E-02	protein NRT1/ PTR FAMILY 6.3-like
Ma02_g23730	ITC1587_Bchr2_P05041	2190.05	-2.34	0.52	-4.53	5.79E-06	1.51E-03	sucrose synthase 2-like
Ma04_g35640	ITC1587_Bchr4_P11298	145.77	-2.35	0.62	-3.80	1.44E-04	1.03E-02	1-aminocyclopropane-1-carboxylate synthase
Ma03_g20380	ITC1587_Bchr3_P07254	13.23	-2.35	0.79	-2.96	3.06E-03	5.96E-02	probable LRR receptor-like serine/threonine-protein kinase At5g48740
Ma10_g08850	ITC1587_Bchr10_P29634	23.61	-2.36	0.64	-3.71	2.08E-04	1.26E-02	GDP-mannose 4
Ma10_g05210	ITC1587_Bchr10_P28406	29.79	-2.36	0.84	-2.81	4.89E-03	7.64E-02	abscisic acid receptor PYL4-like
Ma02_g16470	ITC1587_Bchr2_P04392	28.44	-2.36	0.67	-3.51	4.54E-04	2.02E-02	G-type lectin S-receptor-like serine/threonine-protein kinase At5g35370
Ma07_g19950	ITC1587_Bchr7_P20655	45.12	-2.37	0.42	-5.59	2.29E-08	4.36E-05	putative clathrin assembly protein At1g25240
Ma01_g12160	ITC1587_Bchr1_P01666	4.97	-2.37	0.86	-2.76	5.71E-03	8.30E-02	putative Transcription factor bHLH14
Ma02_g11730	ITC1587_Bchr2_P03989	265.09	-2.37	0.65	-3.64	2.71E-04	1.48E-02	MATE efflux family protein LAL5-like
Ma06_g23440	ITC1587_Bchr6_P16951	59.5	-2.37	0.82	-2.88	4.01E-03	6.90E-02	fasciclin-like arabinogalactan protein 12
Ma07_g14930	ITC1587_Bchr7_P19944	6.24	-2.37	0.78	-3.03	2.47E-03	5.23E-02	putative DNA-binding protein ESCAROLA

Ma07_g14130	ITC1587_Bchr7_P19873	111.91	-2.38	0.62	-3.82	1.32E-04	9.87E-03	Hypothetical protein
Ma11_g19490	ITC1587_Bchr11_P33922	12.58	-2.38	0.7	-3.41	6.40E-04	2.46E-02	CBS domain-containing protein CBSX5
Ma06_g02290	ITC1587_Bchr6_P14833	6.44	-2.38	0.84	-2.83	4.68E-03	7.51E-02	probable indole-3-acetic acid-amido synthetase GH3.1
Ma02_g05080	ITC1587_Bchr2_P03379	9.31	-2.39	0.68	-3.49	4.84E-04	2.08E-02	uncharacterized LOC103970726
Ma09_g07210	ITC1587_Bchr9_P25689	401.06	-2.39	0.47	-5.08	3.86E-07	2.71E-04	uncharacterized LOC103997178
Ma05_g13300	ITC1587_Bchr5_P12854	13.96	-2.39	0.71	-3.38	7.23E-04	2.61E-02	zinc finger protein ZAT9-like
Ma01_g17140	ITC1587_Bchr1_P02279	38.04	-2.4	0.67	-3.59	3.30E-04	1.68E-02	proline-rich receptor-like protein kinase PERK8
Ma11_g24340	ITC1587_Bchr11_P34328	10.47	-2.41	0.89	-2.7	6.99E-03	9.38E-02	myosin-2-like
Ma08_g01770	ITC1587_Bchr8_P21632	21.18	-2.42	0.5	-4.86	1.19E-06	5.58E-04	protein YIPF5 homolog
Ma02_g18130	ITC1587_Bchr2_P04535	6.68	-2.42	0.82	-2.95	3.18E-03	6.10E-02	probable WRKY transcription factor 49
Ma06_g23490	ITC1587_Bchr1_P02617	7.65	-2.43	0.56	-4.3	1.70E-05	2.91E-03	putative expressed protein
Ma03_g10210	ITC1587_Bchr3_P06107	5.33	-2.43	0.82	-2.95	3.19E-03	6.11E-02	heat shock factor-binding protein 1-like
Ma08_g18540	ITC1587_Bchr8_P23091	5.23	-2.43	0.88	-2.77	5.52E-03	8.17E-02	Myb-related protein 306
Ma10_g17780	ITC1587_Bchr10_P30481	141.24	-2.43	0.53	-4.58	4.62E-06	1.28E-03	uncharacterized LOC103968531
Ma08_g15160	ITC1587_Bchr8_P23687	23.78	-2.43	0.85	-2.87	4.17E-03	7.06E-02	uncharacterized LOC103994997
Ma09_g26460	ITC1587_Bchr9_P27894	7.92	-2.45	0.75	-3.25	1.15E-03	3.44E-02	protein TIFY 5A-like
Ma07_g14050	ITC1587_Bchr7_P19865	11.29	-2.45	0.81	-3.04	2.35E-03	5.10E-02	uncharacterized protein At5g05190-like
Ma08_g19170	ITC1587_Bchr8_P23014	4.89	-2.45	0.89	-2.77	5.59E-03	8.22E-02	putative ethylene-responsive transcription factor ERF021
Ma07_g13700	ITC1587_Bchr7_P19833	14.74	-2.46	0.71	-3.45	5.70E-04	2.29E-02	uncharacterized LOC103991611
Ma05_g21280	ITC1587_Bchr5_P13755	10.79	-2.46	0.84	-2.92	3.53E-03	6.45E-02	actin-depolymerizing factor 5-like
Ma10_g14360	ITC1587_Bchr10_P30176	26.15	-2.46	0.65	-3.81	1.39E-04	1.01E-02	peptide-N4-(N-acetyl-beta-glucosaminyl)asparagine amidase A-like
Ma01_g16610	ITC1587_Bchr1_P02055	4.71	-2.46	0.87	-2.83	4.65E-03	7.50E-02	kinesin-like protein NACK1
Ma04_g26300	ITC1587_Bchr4_P10473	65.05	-2.47	0.72	-3.45	5.67E-04	2.28E-02	dehydration-responsive element-binding protein 1D
Ma08_g24180	ITC1587_Bchr8_P24100	74.67	-2.47	0.84	-2.95	3.14E-03	6.04E-02	fasciclin-like arabinogalactan protein 1
Ma05_g02370	ITC1587_Bchr5_P11862	45.51	-2.47	0.91	-2.7	6.83E-03	9.25E-02	auxin response factor 17
Ma07_g13720	ITC1587_Bchr7_P19835	4.12	-2.48	0.8	-3.09	2.02E-03	4.72E-02	serine/threonine-protein kinase At3g07070-like

Ma09_g17640	ITC1587_Bchr9_P26625	7.42	-2.48	0.75	-3.29	1.00E-03	3.16E-02	pollen-specific protein SF3-like
Ma04_g11990	ITC1587_Bchr4_P09471	105.76	-2.48	0.61	-4.05	5.02E-05	5.66E-03	probable xyloglucan endotransglucosylase/hydrolase protein 32
Ma09_g13070	ITC1587_Bchr9_P26191	29.94	-2.49	0.54	-4.60	4.20E-06	1.26E-03	UDP-glucuronate 4-epimerase 1
Ma11_g13910	ITC1587_Bchr11_P33372	17.53	-2.49	0.77	-3.23	1.25E-03	3.61E-02	probable glucan endo-1, 3-beta-glucosidase A6
Ma05_g05130	ITC1587_Bchr8_P24098*	36.06	-2.49	0.56	-4.43	9.37E-06	2.10E-03	RNA-binding protein 38-like
Ma09_g30640	ITC1587_Bchr9_P28271	11.69	-2.49	0.59	-4.23	2.39E-05	3.57E-03	wound induced protein
Ma10_g11070	ITC1587_Bchr10_P29898*	5.49	-2.5	0.89	-2.81	4.89E-03	7.64E-02	uncharacterized LOC104000794
Ma06_g05050	ITC1587_Bchr6_P15089	10.32	-2.51	0.7	-3.57	3.59E-04	1.77E-02	probable inactive leucine-rich repeat receptor-like protein kinase At1g66830
Ma08_g23930	ITC1587_Bchr8_P24076	24.38	-2.51	0.93	-2.71	6.75E-03	9.20E-02	putative uncharacterized protein Sb04g034290
Ma06_g00990	ITC1587_Bchr11_P32678	11.14	-2.51	0.93	-2.71	6.79E-03	9.22E-02	uncharacterized LOC103971064
Ma08_g04700	ITC1587_Bchr8_P21864	108.7	-2.51	0.8	-3.13	1.75E-03	4.37E-02	allene oxide synthase 2-like
Ma03_g17210	ITC1587_Bchr3_P06858	262.27	-2.51	0.51	-4.91	9.18E-07	5.16E-04	probable WRKY transcription factor 41
Ma11_g06010	ITC1587_Bchr11_P32272	99.12	-2.52	0.88	-2.85	4.32E-03	7.19E-02	ruBisCO large subunit-binding protein subunit beta
Ma01_g03380	ITC1587_Bchr1_P00730	302.36	-2.54	0.77	-3.27	1.06E-03	3.26E-02	ABC transporter G family member 39-like
Ma03_g31930	ITC1587_Bchr3_P08224	4.3	-2.54	0.86	-2.95	3.15E-03	6.06E-02	uncharacterized LOC103980060
Ma04_g36920	ITC1587_Bchr4_P11121*	74.04	-2.54	0.58	-4.38	1.18E-05	2.43E-03	indole-3-acetic acid-induced protein ARG7-like
Ma09_g12200	ITC1587_Bchr9_P26119*	22.28	-2.54	0.83	-3.05	2.30E-03	5.05E-02	arabinogalactan peptide 23-like
Ma07_g14940	ITC1587_Bchr7_P19945	308.34	-2.54	0.55	-4.61	3.94E-06	1.22E-03	uncharacterized LOC103991701
Ma01_g09920	ITC1587_Bchr1_P01452	11.23	-2.54	0.7	-3.63	2.86E-04	1.53E-02	uncharacterized LOC103983940
Ma04_g35750	ITC1587_Bchr4_P11307	27.45	-2.55	0.77	-3.32	9.13E-04	2.99E-02	GDSL esterase/lipase At5g33370-like
Ma09_g29210	ITC1587_Bchr9_P28142	6.88	-2.55	0.91	-2.81	5.02E-03	7.72E-02	RNA-binding protein cabeza-like
Ma03_g14690	ITC1587_Bchr3_P06593	1231.45	-2.55	0.95	-2.67	7.50E-03	9.72E-02	catalase isozyme A-like
Ma02_g23970	ITC1587_Bchr10_P30575*	6.1	-2.56	0.95	-2.71	6.82E-03	9.24E-02	non-specific lipid-transfer protein-like protein At5g64080
Ma09_g21650	ITC1587_BchrUn_random_P35241*	8.86	-2.56	0.58	-4.45	8.59E-06	2.00E-03	uncharacterized LOC103973198
Ma08_g07230	ITC1587_Bchr8_P22080	17.19	-2.56	0.66	-3.87	1.09E-04	8.71E-03	uncharacterized LOC103993598

Ma05_g13330	ITC1587_Bchr5_P12858	46.09	-2.57	0.68	-3.77	1.66E-04	1.13E-02	GATA transcription factor 4-like
Ma04_g03810	ITC1587_Bchr4_P08729	18.85	-2.57	0.7	-3.69	2.21E-04	1.30E-02	LOB domain-containing protein 41-like
Ma11_g18960	ITC1587_Bchr11_P33863	28.55	-2.57	0.85	-3.02	2.49E-03	5.26E-02	pectinesterase-like
Ma05_g06620	ITC1587_Bchr5_P12275	14.06	-2.57	0.89	-2.88	3.97E-03	6.85E-02	indole-3-pyruvate monooxygenase YUCCA2-like
Ma11_g03080	ITC1587_Bchr11_P31980	15.79	-2.57	0.75	-3.45	5.68E-04	2.28E-02	Dehydration-responsive element-binding protein 1G
Ma08_g23110		18.5	-2.57	0.8	-3.23	1.23E-03	3.57E-02	extensin-1-like
Ma10_g05760	ITC1587_Bchr10_P28450	219.18	-2.58	0.49	-5.23	1.70E-07	1.68E-04	nematode resistance protein-like HSPRO2
Ma06_g36300	ITC1587_Bchr9_P25344*	24.95	-2.58	0.79	-3.29	1.00E-03	3.16E-02	uncharacterized LOC103990145
Ma05_g19060	ITC1587_Bchr5_P13596	10.35	-2.59	0.78	-3.31	9.35E-04	3.02E-02	phytosulfokine receptor 1-like
Ma02_g10610	ITC1587_Bchr2_P03888	13.71	-2.59	0.76	-3.40	6.86E-04	2.54E-02	Non-symbiotic hemoglobin 2
Ma03_g22970	ITC1587_Bchr3_P07482	12.24	-2.6	0.95	-2.75	6.03E-03	8.58E-02	uncharacterized LOC103979139
Ma04_g04810	ITC1587_Bchr4_P08814	12.93	-2.61	0.94	-2.77	5.61E-03	8.24E-02	leucine-rich repeat receptor-like protein kinase TDR
Ma10_g25820	ITC1587_Bchr10_P31161*	10.62	-2.61	0.58	-4.49	7.14E-06	1.81E-03	transcription initiation factor TFIID subunit 9-like
Ma00_g02690	ITC1587_BchrUn_random_P36587	13.62	-2.61	0.78	-3.35	8.04E-04	2.79E-02	geranylgeranyl pyrophosphate synthase 7
Ma04_g06820	ITC1587_Bchr4_P08981	39.65	-2.61	0.58	-4.53	6.02E-06	1.56E-03	LOB domain-containing protein 41-like
Ma08_g27500	ITC1587_Bchr8_P24395	12.78	-2.62	0.82	-3.19	1.42E-03	3.89E-02	protein IQ-DOMAIN 14-like
Ma07_g19240	ITC1587_Bchr2_P04614*	8.33	-2.63	0.73	-3.62	2.90E-04	1.54E-02	tubulin alpha chain-like
Ma09_g02190	ITC1587_Bchr9_P25266	128.87	-2.63	0.95	-2.78	5.50E-03	8.17E-02	cycloeucalenol cycloisomerase-like
Ma05_g06250	ITC1587_Bchr5_P12241	158.15	-2.63	0.6	-4.37	1.24E-05	2.44E-03	uncharacterized LOC103984099
Ma10_g20250	ITC1587_Bchr10_P30682	13.39	-2.64	0.84	-3.15	1.63E-03	4.20E-02	piriformospora indica-insensitive protein 2-like
Ma03_g06360	ITC1587_Bchr3_P05737	84.16	-2.64	0.93	-2.85	4.37E-03	7.24E-02	uncharacterized LOC103977492
Ma07_g22520	ITC1587_Bchr7_P20887	17.32	-2.64	0.72	-3.69	2.28E-04	1.32E-02	transcription factor TCP15-like
Ma07_g15950	ITC1587_Bchr7_P20166	97.45	-2.65	0.60	-4.44	9.04E-06	2.05E-03	probable xyloglucan endotransglucosylase/hydrolase protein 23
Ma03_g31820	ITC1587_Bchr3_P08215	18.14	-2.65	0.86	-3.07	2.15E-03	4.85E-02	leucine-rich repeat receptor-like serine/threonine-protein kinase BAM1
Ma03_g12500	ITC1587_Bchr3_P06308	195.47	-2.65	0.84	-3.16	1.55E-03	4.09E-02	probable boron transporter 2

Ma07_g19790	ITC1587_Bchr7_P20632	546.79	-2.65	0.49	-5.42	5.80E-08	8.60E-05	probable galacturonosyltransferase-like 6
Ma06_g29690	ITC1587_Bchr6_P17717	250.02	-2.66	0.82	-3.25	1.17E-03	3.47E-02	probably inactive leucine-rich repeat receptor-like protein kinase At3g28040
Ma07_g08160	ITC1587_Bchr7_P19295	10.24	-2.66	0.96	-2.77	5.68E-03	8.27E-02	uncharacterized LOC103990315
Ma03_g30740	ITC1587_Bchr3_P08118	32.96	-2.66	0.74	-3.58	3.43E-04	1.72E-02	LOB domain-containing protein 40
Ma06_g09230	ITC1587_Bchr6_P15464*	61.57	-2.66	0.57	-4.70	2.66E-06	9.47E-04	mitogen-activated protein kinase kinase kinase NPK1-like
Ma08_g07110	ITC1587_Bchr8_P22071	90.51	-2.66	0.80	-3.32	9.06E-04	2.99E-02	cytochrome P450 77A3-like
Ma09_g23420	ITC1587_Bchr9_P27596	168.87	-2.67	0.55	-4.86	1.15E-06	5.46E-04	probable WRKY transcription factor 17
Ma03_g14710	ITC1587_Bchr3_P07923	3.04	-2.67	0.91	-2.93	3.35E-03	6.25E-02	molybdate transporter 1-like
Ma05_g07100	ITC1587_Bchr5_P12316	62.97	-2.68	0.71	-3.77	1.65E-04	1.13E-02	probable protein phosphatase 2C 32
Ma02_g19010	ITC1587_Bchr2_P04614	291.16	-2.68	0.79	-3.41	6.54E-04	2.47E-02	Tubulin alpha-1 chain
Ma03_g20840	ITC1587_Bchr6_P15727*	11.52	-2.68	1.00	-2.69	7.21E-03	9.52E-02	HVA22-like protein a
Ma10_g05380	ITC1587_Bchr3_P06498	19.93	-2.68	0.76	-3.54	4.03E-04	1.90E-02	interactor of constitutive active ROPs 3-like
Ma06_g12780	ITC1587_Bchr6_P15787	125.92	-2.7	0.77	-3.52	4.38E-04	1.98E-02	U-box domain-containing protein 21-like
Ma05_g29880	ITC1587_Bchr5_P14591	80.4	-2.7	0.95	-2.85	4.39E-03	7.26E-02	putative probable glutathione S-transferase
Ma09_g12180	ITC1587_Bchr9_P26117	6.21	-2.71	1.01	-2.68	7.44E-03	9.68E-02	60S ribosomal protein L31-like
Ma03_g25130	ITC1587_Bchr3_P07656	47.39	-2.71	0.70	-3.89	9.99E-05	8.37E-03	CASP-like protein 5
Ma08_g20310	ITC1587_Bchr8_P23841	5.83	-2.72	0.78	-3.50	4.71E-04	2.05E-02	GRF1-interacting factor 1-like
Ma06_g16090	ITC1587_Bchr6_P16085*	10.23	-2.73	0.75	-3.64	2.72E-04	1.48E-02	Peroxidase 70
Ma04_g14180	ITC1587_Bchr4_P09673	4.02	-2.73	0.88	-3.08	2.06E-03	4.74E-02	protein SYM1-like
Ma08_g04050	ITC1587_Bchr8_P21808	92.76	-2.73	0.79	-3.47	5.27E-04	2.18E-02	putative probable 3-ketoacyl-CoA synthase 2
Ma09_g27700	ITC1587_Bchr9_P27999	22.96	-2.74	0.93	-2.93	3.34E-03	6.24E-02	galactoside 2-alpha-L-fucosyltransferase-like
Ma09_g05070	ITC1587_Bchr9_P25505	6.53	-2.74	0.86	-3.20	1.36E-03	3.79E-02	probable E3 ubiquitin-protein ligase RHA2B
Ma06_g28510	ITC1587_Bchr6_P17603	5.79	-2.75	0.73	-3.74	1.84E-04	1.19E-02	CBL-interacting serine/threonine-protein kinase 12-like
Ma04_g15370	ITC1587_Bchr4_P09793	6.45	-2.75	0.99	-2.79	5.23E-03	7.94E-02	zinc finger CCCH domain-containing protein 2-like
Ma06_g07550	ITC1587_Bchr6_P15308	7.03	-2.76	0.99	-2.81	5.01E-03	7.72E-02	probable inactive receptor kinase At1g48480

Ma08_g33400	ITC1587_Bchr8_P24916	24.47	-2.78	0.93	-2.99	2.83E-03	5.71E-02	cytochrome P450 94C1-like
Ma07_g25090	ITC1587_Bchr7_P21120	7.13	-2.78	0.81	-3.45	5.61E-04	2.27E-02	uncharacterized LOC103992655
Ma08_g04230	ITC1587_Bchr8_P21821	22.84	-2.79	0.88	-3.15	1.62E-03	4.20E-02	cytosolic sulfotransferase 12-like
Ma08_g00620	ITC1587_Bchr8_P21527	56.38	-2.79	0.61	-4.6	4.18E-06	1.26E-03	glycerophosphodiester phosphodiesterase GDE1-like
Ma06_g14720	ITC1587_Bchr6_P15951	49.93	-2.79	0.76	-3.67	2.46E-04	1.38E-02	uncharacterized LOC103987702
Ma06_g36600	ITC1587_Bchr6_P18326	137.87	-2.8	0.99	-2.82	4.87E-03	7.62E-02	L-ascorbate oxidase homolog
Ma01_g17600	ITC1587_Bchr1_P02234	122.67	-2.8	0.63	-4.46	8.04E-06	1.93E-03	uncharacterized LOC103996876
Ma03_g01640	ITC1587_Bchr3_P05317	12.54	-2.8	1.04	-2.69	7.10E-03	9.47E-02	UDP-glucuronate:xylan alpha-glucuronosyltransferase 2-like
Ma02_g13620	ITC1587_Bchr2_P04137	34.15	-2.81	0.84	-3.35	8.08E-04	2.79E-02	GDSL esterase/lipase At5g33370-like
Ma02_g08560	ITC1587_Bchr2_P03698	9.17	-2.81	1.02	-2.76	5.85E-03	8.44E-02	heptahelical transmembrane protein 4-like
Ma07_g27150	ITC1587_Bchr7_P21294	241.48	-2.82	0.73	-3.86	1.14E-04	8.85E-03	putative calcium-transporting ATPase 13
Ma06_g02740	ITC1587_Bchr6_P14869	9.54	-2.82	0.81	-3.50	4.69E-04	2.05E-02	probable inositol oxygenase
Ma05_g13670	ITC1587_Bchr3_P07125*	5.58	-2.82	0.97	-2.90	3.70E-03	6.58E-02	vacuolar protein 8-like
Ma09_g13640	ITC1587_Bchr9_P26245	106.86	-2.83	0.82	-3.46	5.41E-04	2.22E-02	leucine-rich repeat extensin-like protein 4
Ma11_g03090	ITC1587_Bchr11_P31981	24.88	-2.83	0.91	-3.12	1.82E-03	4.46E-02	putative ethylene-responsive transcription factor ERF024
Ma08_g17410		9.5	-2.83	0.93	-3.05	2.32E-03	5.06E-02	putative uncharacterized protein
Ma05_g00230	ITC1587_Bchr5_P11686	13.21	-2.83	0.75	-3.8	1.44E-04	1.03E-02	protein G1-like1
Ma06_g13970	ITC1587_Bchr6_P15889	108.31	-2.84	0.75	-3.79	1.52E-04	1.07E-02	fructokinase-1-like
Ma08_g11860	ITC1587_Bchr8_P22521	16.81	-2.84	0.96	-2.97	2.97E-03	5.89E-02	probable LRR receptor-like serine/threonine-protein kinase At4g36180
Ma10_g12270	ITC1587_Bchr10_P29998	24.88	-2.85	0.7	-4.08	4.58E-05	5.33E-03	obtusifoliol 14-alpha demethylase
Ma07_g20050	ITC1587_BchrUn_random_P38638	44.15	-2.85	0.63	-4.55	5.49E-06	1.46E-03	uncharacterized protein At1g66480-like
Ma10_g14730	ITC1587_Bchr10_P30212	15.02	-2.85	0.95	-3.00	2.68E-03	5.52E-02	GDSL esterase/lipase At4g01130
Ma10_g07300	ITC1587_Bchr10_P29473	58.01	-2.86	0.68	-4.23	2.29E-05	3.47E-03	Peroxidase 4
Ma08_g30810	ITC1587_Bchr8_P24701	5.45	-2.86	0.98	-2.94	3.31E-03	6.20E-02	PI-PLC X domain-containing protein At5g67130-like
Ma06_g10710	ITC1587_Bchr6_P15596	107.98	-2.87	0.76	-3.79	1.50E-04	1.06E-02	uncharacterized LOC103987361
Ma02_g02850	ITC1587_Bchr10_P29855	81.9	-2.87	0.6	-4.75	2.01E-06	7.95E-04	protein YLS9-like

Ma03_g28120	ITC1587_Bchr3_P07923*	4	-2.87	0.86	-3.32	8.92E-04	2.96E-02	molybdate transporter 1-like
Ma10_g20040	ITC1587_Bchr10_P30664	9.65	-2.87	0.85	-3.4	6.79E-04	2.53E-02	uncharacterized LOC103968734
Ma03_g09530	ITC1587_Bchr3_P06046	47.82	-2.88	0.72	-3.98	6.92E-05	6.80E-03	receptor-like protein kinase FERONIA
Ma04_g28060	ITC1587_Bchr4_P10630	35.63	-2.88	0.51	-5.70	1.22E-08	3.24E-05	Hypothetical protein
Ma04_g09870	ITC1587_Bchr4_P09263	15.63	-2.89	0.94	-3.08	2.05E-03	4.73E-02	probable inactive receptor kinase At5g67200
Ma10_g13650	ITC1587_Bchr10_P30109	11.29	-2.9	0.72	-4.04	5.33E-05	5.85E-03	zinc finger protein ZAT12-like
Ma11_g04680	ITC1587_Bchr11_P32138	142	-2.91	0.99	-2.94	3.29E-03	6.20E-02	transcription factor MYB108-like
Ma02_g00070	ITC1587_BchrUn_random_P36126	26	-2.91	0.69	-4.21	2.59E-05	3.70E-03	Hypothetical protein
Ma09_g12170	ITC1587_Bchr9_P26116	62.46	-2.91	0.87	-3.35	7.98E-04	2.78E-02	DNA-damage-repair/toleration protein DRT100-like
Ma06_g23400	ITC1587_Bchr6_P17073	5.86	-2.92	1.1	-2.66	7.73E-03	9.84E-02	uncharacterized LOC103988814
Ma03_g05680	ITC1587_Bchr3_P05672	9.68	-2.92	0.67	-4.39	1.14E-05	2.39E-03	pectinesterase-like
Ma10_g25860	ITC1587_Bchr10_P31164	17.21	-2.93	0.81	-3.61	3.08E-04	1.60E-02	homeobox-leucine zipper protein ROC8-like
Ma09_g01640	ITC1587_Bchr9_P25215	5.47	-2.93	1.06	-2.77	5.62E-03	8.24E-02	kinesin-like protein KIN12B
Ma07_g14030	ITC1587_Bchr7_P19864	354.92	-2.94	0.64	-4.57	4.90E-06	1.33E-03	probable WRKY transcription factor 41
Ma10_g30010	ITC1587_Bchr10_P31541	5.47	-2.94	1	-2.93	3.38E-03	6.25E-02	putative uncharacterized protein Sb04g034290
Ma06_g14410	ITC1587_BchrUn_random_P39500	50.69	-2.94	0.95	-3.09	2.01E-03	4.70E-02	1-aminocyclopropane-1-carboxylate oxidase-like
Ma10_g16460	ITC1587_Bchr10_P30364	8.84	-2.94	0.86	-3.43	5.95E-04	2.35E-02	probable fructokinase-1
Ma04_g32220	ITC1587_Bchr4_P11014	10.27	-2.94	0.96	-3.06	2.19E-03	4.91E-02	glucan endo-1, 3-beta-glucosidase 11-like
Ma10_g04750	ITC1587_Bchr10_P29340	60.78	-2.95	0.85	-3.47	5.24E-04	2.17E-02	homeobox-leucine zipper protein ROC2-like
Ma04_g11950	ITC1587_Bchr4_P09468	13.87	-2.95	0.76	-3.90	9.48E-05	8.08E-03	Tubulin beta-1 chain
Ma06_g15990	ITC1587_Bchr6_P16079	207.64	-2.96	0.66	-4.48	7.54E-06	1.85E-03	epidermis-specific secreted glycoprotein EP1
Ma07_g07210	ITC1587_Bchr7_P19202	15.08	-2.97	0.62	-4.77	1.89E-06	7.74E-04	abscisic acid 8'-hydroxylase 1-like
Ma07_g08820	ITC1587_Bchr7_P19388	14.18	-2.98	1.06	-2.82	4.77E-03	7.58E-02	cytokinin dehydrogenase 6-like
Ma07_g03310	ITC1587_Bchr7_P18846	569.78	-2.99	0.59	-5.10	3.47E-07	2.64E-04	E3 ubiquitin-protein ligase PUB23-like
Ma03_g15640	ITC1587_Bchr3_P06620	6.32	-2.99	0.83	-3.60	3.17E-04	1.63E-02	uncharacterized LOC103978282

Ma08_g14000	ITC1587_Bchr8_P22643*	21.14	-2.99	0.69	-4.31	1.64E-05	2.86E-03	uncharacterized LOC103994530
Ma07_g26900	ITC1587_Bchr7_P21276	41.31	-2.99	0.9	-3.32	9.13E-04	2.99E-02	putative OsWRKY11 - Superfamily of TFs having WRKY and zinc finger domains
Ma08_g10970	ITC1587_Bchr8_P22435	14.79	-3	0.92	-3.25	1.15E-03	3.44E-02	Nitrate transporter 1.1
Ma01_g23310	ITC1587_Bchr1_P02780	18.5	-3	0.94	-3.20	1.36E-03	3.78E-02	CTP synthase-like
Ma06_g26140	ITC1587_Bchr6_P17166	5.5	-3	0.95	-3.15	1.65E-03	4.22E-02	CBL-interacting protein kinase 4-like
Ma10_g22540	ITC1587_Bchr10_P30883	128.19	-3.01	0.75	-4.03	5.64E-05	6.09E-03	uncharacterized LOC103968974
Ma04_g36140	ITC1587_Bchr4_P11346	9.85	-3.02	0.96	-3.14	1.69E-03	4.29E-02	defensin Ec-AMP-D1 {ECO:0000303|PubMed:18625284}-like
Ma05_g05700	ITC1587_Bchr5_P12192	134.63	-3.02	0.62	-4.87	1.14E-06	5.46E-04	nudix hydrolase 17
Ma05_g21310	ITC1587_Bchr5_P13758	3.16	-3.03	1.06	-2.84	4.45E-03	7.30E-02	probable receptor-like serine/threonine-protein kinase At5g57670
Ma06_g35550	ITC1587_Bchr6_P18231	32.16	-3.03	0.81	-3.73	1.95E-04	1.23E-02	probable anion transporter 2
Ma04_g01250	ITC1587_Bchr4_P08494	66.11	-3.03	0.7	-4.3	1.74E-05	2.91E-03	serine carboxypeptidase-like 12
Ma09_g19260	ITC1587_Bchr1_P00394*	6.41	-3.03	0.86	-3.54	4.05E-04	1.90E-02	Probable aquaporin NIP5-1
Ma01_g06630	ITC1587_Bchr1_P01158	5.69	-3.04	1.1	-2.77	5.65E-03	8.26E-02	uncharacterized LOC103989520
Ma06_g04150	ITC1587_Bchr6_P14994	408.97	-3.04	0.69	-4.37	1.22E-05	2.44E-03	U-box domain-containing protein 21-like
Ma05_g24600	ITC1587_Bchr5_P14115	1301.12	-3.04	0.48	-6.29	3.24E-10	2.88E-06	putative nuclease HARBI1
Ma04_g09400	ITC1587_Bchr4_P09222	21.04	-3.04	0.93	-3.27	1.06E-03	3.26E-02	probable LRR receptor-like serine/threonine-protein kinase At4g37250
Ma07_g20260	ITC1587_Bchr7_P20688	63.54	-3.05	0.52	-5.87	4.24E-09	1.61E-05	lipase
Ma10_g27380	ITC1587_Bchr10_P31305	64.6	-3.05	0.73	-4.18	2.93E-05	4.01E-03	uncharacterized LOC103969384
Ma10_g25140	ITC1587_Bchr10_P31112	8.1	-3.05	1.03	-2.95	3.18E-03	6.10E-02	putative uncharacterized protein Sb04g034290
Ma01_g17590	ITC1587_Bchr1_P02234*	133.65	-3.06	0.63	-4.89	1.02E-06	5.33E-04	uncharacterized LOC103996881
Ma01_g17700	ITC1587_Bchr1_P02223	22.67	-3.08	0.83	-3.71	2.08E-04	1.26E-02	uncharacterized LOC103996818
Ma02_g22650	ITC1587_Bchr2_P04949*	5.84	-3.08	0.84	-3.68	2.38E-04	1.35E-02	cell wall / vacuolar inhibitor of fructosidase 2-like
Ma04_g01520	ITC1587_Bchr4_P08524	10.5	-3.08	0.77	-4.00	6.23E-05	6.52E-03	probable protein phosphatase 2C 66
Ma09_g06290	ITC1587_Bchr9_P25602	20.92	-3.09	0.95	-3.25	1.16E-03	3.44E-02	gibberellin 2-beta-dioxygenase-like
Ma09_g12240	ITC1587_Bchr9_P26123	79.22	-3.09	0.55	-5.67	1.46E-08	3.55E-05	uncharacterized LOC103997737

Ma03_g18650	ITC1587_Bchr3_P07066	134.52	-3.1	0.78	-3.99	6.71E-05	6.75E-03	glycerol-3-phosphate 2-O-acyltransferase 6-like
Ma08_g24920	ITC1587_Bchr8_P24157*	14.78	-3.1	0.94	-3.30	9.59E-04	3.07E-02	ABC transporter B family member 19
Ma05_g23650	ITC1587_Bchr5_P14009	35.98	-3.11	0.69	-4.50	6.84E-06	1.75E-03	uncharacterized LOC103985606
Ma09_g29700	ITC1587_Bchr9_P28185	9.22	-3.11	0.88	-3.54	4.05E-04	1.90E-02	G-type lectin S-receptor-like serine/threonine-protein kinase At5g35370
Ma02_g20740	ITC1587_Bchr2_P04765	23.41	-3.11	0.84	-3.70	2.13E-04	1.28E-02	patellin-6-like
Ma08_g25110	ITC1587_Bchr8_P24173	27.52	-3.12	0.96	-3.27	1.08E-03	3.30E-02	beta-glucosidase 24-like
Ma09_g02840	ITC1587_Bchr9_P25325	20.45	-3.12	0.8	-3.88	1.05E-04	8.56E-03	receptor-like protein kinase THESEUS 1
Ma01_g21640	ITC1587_Bchr1_P02753	190.83	-3.12	0.6	-5.23	1.69E-07	1.68E-04	probable calcium-binding protein CML31
Ma03_g03430	ITC1587_Bchr3_P05474	8.13	-3.14	1.08	-2.90	3.75E-03	6.61E-02	protein YLS9-like
Ma07_g12500	ITC1587_Bchr7_P19720	176.74	-3.15	0.45	-6.98	2.95E-12	3.93E-08	uncharacterized LOC103991515
Ma03_g24400	ITC1587_Bchr3_P07592	20.46	-3.15	0.97	-3.25	1.16E-03	3.44E-02	cytochrome P450 77A3-like
Ma11_g17680	ITC1587_Bchr11_P33750	52.75	-3.16	1.12	-2.82	4.83E-03	7.60E-02	fructokinase-1-like
Ma02_g04960	ITC1587_Bchr4_P11644*	6.2	-3.16	1.19	-2.66	7.83E-03	9.92E-02	CASP-like protein VIT_19s0090g00570
Ma06_g14420	ITC1587_BchrUn_random _P39500*	25.96	-3.17	0.97	-3.28	1.04E-03	3.23E-02	1-aminocyclopropane-1-carboxylate oxidase-like
Ma05_g15530	ITC1587_Bchr5_P13076	28.21	-3.17	1	-3.19	1.43E-03	3.91E-02	probable pectate lyase 8
Ma09_g28820	ITC1587_Bchr9_P28107	11.79	-3.18	0.83	-3.85	1.18E-04	9.10E-03	cyclin-D1-2-like
Ma04_g21130	ITC1587_Bchr1_P00054	9.82	-3.18	0.99	-3.22	1.26E-03	3.62E-02	cyclin-P4-1-like
Ma07_g21980	ITC1587_Bchr7_P20841*	7.49	-3.19	1.04	-3.05	2.27E-03	5.01E-02	anthranilate O-methyltransferase 3-like
Ma06_g25850	ITC1587_Bchr6_P17218	40.01	-3.19	0.94	-3.39	7.06E-04	2.57E-02	3-ketoacyl-CoA synthase 11
Ma06_g34860	ITC1587_Bchr6_P18193	24.27	-3.2	0.86	-3.71	2.05E-04	1.26E-02	zinc finger protein ZAT12-like
Ma04_g20470	ITC1587_Bchr8_P22601*	81.96	-3.2	1.2	-2.66	7.71E-03	9.83E-02	Probable aquaporin TIP1-1
Ma04_g25030	ITC1587_Bchr1_P00499	6.26	-3.21	1.16	-2.76	5.87E-03	8.45E-02	probable WRKY transcription factor 72
Ma02_g05070	ITC1587_Bchr2_P03378	5.31	-3.21	0.95	-3.37	7.63E-04	2.69E-02	uncharacterized LOC103970718
Ma07_g01020	ITC1587_Bchr7_P18638	18.3	-3.21	0.73	-4.38	1.18E-05	2.43E-03	probable LRR receptor-like serine/threonine-protein kinase At4g20940
Ma06_g04640	ITC1587_Bchr6_P15045	164.18	-3.24	1.03	-3.13	1.73E-03	4.36E-02	36.4 kDa proline-rich protein-like
Ma09_g13490	ITC1587_Bchr9_P26231	11.92	-3.24	0.84	-3.87	1.11E-04	8.77E-03	uncharacterized LOC103997893
Ma09_g30440	ITC1587_Bchr6_P16524*	50.79	-3.24	1.12	-2.91	3.66E-03	6.56E-02	fatty acyl-CoA reductase 3-like

Ma01_g21990	ITC1587_BchrUn_random_P35548	19.35	-3.25	0.99	-3.27	1.06E-03	3.25E-02	uncharacterized LOC103973334
Ma10_g15930	ITC1587_Bchr10_P30315	21.31	-3.25	0.91	-3.57	3.56E-04	1.76E-02	uncharacterized LOC103968378
Ma08_g26370	ITC1587_Bchr9_P28060*	8.82	-3.25	1.12	-2.91	3.57E-03	6.47E-02	uncharacterized LOC103996260
Ma05_g05400	ITC1587_Bchr5_P12160	25.63	-3.26	0.97	-3.38	7.26E-04	2.61E-02	missing_product
Ma05_g03690	ITC1587_Bchr5_P11998	54.58	-3.28	1	-3.28	1.03E-03	3.21E-02	myb-related protein Myb4-like
Ma06_g32560	ITC1587_Bchr6_P17982	7.62	-3.28	0.82	-3.98	6.83E-05	6.80E-03	probable xyloglucan endotransglucosylase/hydrolase protein 23
Ma02_g18300	ITC1587_Bchr2_P04549	38.14	-3.28	0.75	-4.39	1.12E-05	2.39E-03	probable mannose-1-phosphate guanylyltransferase 1
Ma06_g02570	ITC1587_Bchr6_P14855*	444.7	-3.3	0.7	-4.68	2.85E-06	9.82E-04	putative nuclease HARBI1
Ma09_g22130	ITC1587_Bchr9_P27465*	11.78	-3.3	1.05	-3.13	1.76E-03	4.37E-02	protein YLS3-like
Ma09_g18960	ITC1587_BchrUn_random_P36066	125.83	-3.3	0.57	-5.82	5.98E-09	1.99E-05	nudix hydrolase 17
Ma04_g39430	ITC1587_Bchr4_P11601*	32.71	-3.31	0.99	-3.33	8.74E-04	2.93E-02	UDP-arabinopyranose mutase 3
Ma03_g21690	ITC1587_Bchr3_P07365	48.73	-3.32	0.84	-3.96	7.47E-05	7.04E-03	putative Transcription factor bHLH36
Ma08_g15280	ITC1587_Bchr8_P22875	10.35	-3.32	0.96	-3.47	5.17E-04	2.16E-02	Hypothetical protein
Ma05_g22570	ITC1587_Bchr5_P13888	55.14	-3.32	1.06	-3.13	1.74E-03	4.36E-02	subtilisin-like protease
Ma01_g06950	ITC1587_Bchr1_P01188	8.56	-3.33	1.04	-3.21	1.31E-03	3.70E-02	probable inactive leucine-rich repeat receptor-like protein kinase At1g66830
Ma11_g18320	ITC1587_Bchr11_P33808	8.54	-3.34	1.06	-3.14	1.71E-03	4.32E-02	uncharacterized LOC103971906
Ma10_g10000	ITC1587_Bchr10_P29739	14.14	-3.34	1	-3.34	8.33E-04	2.84E-02	probable inactive receptor kinase At5g67200
Ma06_g23720	ITC1587_Bchr6_P16915	16.68	-3.36	0.94	-3.56	3.70E-04	1.81E-02	gibberellin 2-beta-dioxygenase 8
Ma10_g26320	ITC1587_Bchr10_P31203	83.67	-3.37	0.71	-4.73	2.24E-06	8.20E-04	uncharacterized LOC103969282
Ma06_g07680	ITC1587_Bchr6_P15318	4.02	-3.37	1.1	-3.05	2.27E-03	5.01E-02	neurofilament medium polypeptide-like
Ma11_g21700		8.49	-3.39	1.23	-2.75	5.96E-03	8.52E-02	uncharacterized LOC103972202
Ma08_g22130	ITC1587_Bchr8_P23909	3.65	-3.41	1.24	-2.75	6.00E-03	8.55E-02	uncharacterized LOC103995402
Ma03_g06600	ITC1587_Bchr3_P05755*	62.95	-3.41	0.87	-3.91	9.13E-05	7.93E-03	uncharacterized LOC103977510
Ma04_g13620	ITC1587_Bchr4_P09615*	341.56	-3.42	1.15	-2.97	2.94E-03	5.87E-02	glu S.griseus protease inhibitor-like
Ma10_g24420	ITC1587_Bchr10_P31042	5.72	-3.42	1.15	-2.97	2.99E-03	5.91E-02	uncharacterized LOC103969118
Ma11_g19990	ITC1587_Bchr11_P33961	25.13	-3.43	0.72	-4.74	2.09E-06	7.96E-04	Dehydration-responsive element-binding protein 1E

Ma09_g10860	ITC1587_Bchr9_P26002	10.95	-3.44	1.11	-3.11	1.85E-03	4.48E-02	dirigent protein 11-like
Ma03_g10560	ITC1587_Bchr9_P28080*	25.95	-3.44	1.02	-3.38	7.20E-04	2.60E-02	uncharacterized LOC103977867
Ma10_g22190	ITC1587_Bchr10_P30849	206.72	-3.46	0.93	-3.72	2.02E-04	1.26E-02	Probable aquaporin TIP2-2
Ma04_g33470	ITC1587_Bchr4_P11108	20.63	-3.46	1.14	-3.02	2.50E-03	5.27E-02	probable pectinesterase 68
Ma10_g04340	ITC1587_Bchr10_P29296	6.57	-3.46	0.99	-3.51	4.49E-04	2.00E-02	putative UDP-glucuronate:xylan alpha-glucuronosyltransferase 3
Ma00_g01600	ITC1587_BchrUn_random_P34617	8.36	-3.47	0.89	-3.91	9.09E-05	7.92E-03	Hypothetical protein
Ma00_g00090	ITC1587_Bchr3_P06825	5.48	-3.47	1.25	-2.78	5.42E-03	8.10E-02	ethylene-responsive transcription factor ERF091
Ma08_g32530	ITC1587_Bchr8_P24850	8.77	-3.49	0.91	-3.85	1.19E-04	9.14E-03	protein IQ-DOMAIN 14-like
Ma05_g28340	ITC1587_Bchr5_P14442	6.24	-3.52	0.83	-4.24	2.20E-05	3.42E-03	patellin-3-like
Ma06_g03990	ITC1587_Bchr6_P14983	20.26	-3.53	1.09	-3.23	1.26E-03	3.62E-02	Ethylene-responsive transcription factor ERF071
Ma07_g28510	ITC1587_Bchr7_P21463	7.37	-3.53	0.95	-3.7	2.16E-04	1.29E-02	leucine-rich repeat receptor-like serine/threonine-protein kinase BAM1
Ma04_g31590	ITC1587_Bchr4_P10960	30.25	-3.54	1.33	-2.66	7.87E-03	9.95E-02	uncharacterized LOC103982764
Ma09_g18930	ITC1587_Bchr9_P27181	52.03	-3.55	1.09	-3.26	1.13E-03	3.38E-02	alpha-dioxygenase 1-like
Ma03_g19660		35.47	-3.55	0.91	-3.92	8.77E-05	7.80E-03	lysine-rich arabinogalactan protein 18-like
Ma03_g32610	ITC1587_Bchr3_P08282	39.24	-3.56	0.94	-3.78	1.54E-04	1.08E-02	uncharacterized LOC103979736
Ma11_g12510	ITC1587_Bchr11_P33216	263.79	-3.56	0.98	-3.63	2.84E-04	1.52E-02	probable beta-D-xylosidase 2
Ma08_g33860	ITC1587_Bchr2_P03229*	78.6	-3.57	0.99	-3.60	3.20E-04	1.64E-02	extensin-like
Ma05_g16710	ITC1587_BchrUn_random_P36267	7.72	-3.61	1.28	-2.81	4.96E-03	7.67E-02	glucan endo-1, 3-beta-glucosidase 11-like
Ma01_g17270	ITC1587_Bchr1_P02265	5.34	-3.61	1.13	-3.18	1.46E-03	3.95E-02	retinitis pigmentosa 1-like 1 protein
Ma09_g30690	ITC1587_Bchr9_P28273	3.73	-3.61	1	-3.61	3.05E-04	1.59E-02	wound induced protein
Ma09_g26140	ITC1587_Bchr7_P19253*	11.06	-3.63	1.17	-3.11	1.89E-03	4.53E-02	non-specific lipid-transfer protein-like protein At2g13820
Ma03_g28150	ITC1587_Bchr3_P07926	8.11	-3.64	1.29	-2.82	4.86E-03	7.62E-02	UTP--glucose-1-phosphate uridylyltransferase-like
Ma09_g14870	ITC1587_Bchr10_P31540*	11.4	-3.64	1	-3.64	2.72E-04	1.48E-02	lysM domain-containing GPI-anchored protein 1-like
Ma03_g07220	ITC1587_Bchr3_P05812	10.04	-3.65	0.94	-3.86	1.13E-04	8.85E-03	thaumatin-like protein

Ma07_g11230	ITC1587_Bchr7_P19593	33.11	-3.67	0.78	-4.71	2.43E-06	8.77E-04	ethylene-responsive transcription factor ERF017-like
Ma08_g09390	ITC1587_Bchr8_P22283	91.99	-3.67	1.13	-3.25	1.17E-03	3.45E-02	protein ASPARTIC PROTEASE IN GUARD CELL 2-like
Ma04_g03470	ITC1587_Bchr4_P08697	13.37	-3.68	1.12	-3.29	1.01E-03	3.17E-02	probable indole-3-acetic acid-amido synthetase GH3.8
Ma01_g21650	ITC1587_Bchr1_P02753*	26.96	-3.68	0.71	-5.16	2.43E-07	2.24E-04	probable calcium-binding protein CML31
Ma08_g16580	ITC1587_Bchr9_P25132*	4.87	-3.69	1.37	-2.70	6.97E-03	9.36E-02	uncharacterized LOC103994558
Ma09_g20470	ITC1587_Bchr9_P27291	49.83	-3.7	1.11	-3.34	8.45E-04	2.87E-02	probable xyloglucan endotransglucosylase/hydrolase protein 32
Ma05_g31990	ITC1587_Bchr5_P14775	8.5	-3.7	1.31	-2.83	4.68E-03	7.51E-02	transcription repressor OFP1-like
Ma09_g01250	ITC1587_Bchr9_P25174	44.67	-3.73	1.15	-3.23	1.22E-03	3.57E-02	neurofilament heavy polypeptide
Ma05_g26750	ITC1587_Bchr5_P14305	6.79	-3.74	1.02	-3.65	2.63E-04	1.45E-02	protein NRT1/ PTR FAMILY 3.1
Ma06_g10920	ITC1587_Bchr6_P15614	21.72	-3.74	1.03	-3.64	2.75E-04	1.49E-02	uncharacterized GPI-anchored protein At4g28100-like
Ma10_g08220	ITC1587_Bchr11_P32383*	27.71	-3.74	1.17	-3.19	1.40E-03	3.87E-02	uncharacterized LOC103972743
Ma04_g27430	ITC1587_Bchr4_P10571	5.08	-3.77	1.33	-2.84	4.51E-03	7.37E-02	proline-rich receptor-like protein kinase PERK1
Ma01_g18460	ITC1587_Bchr1_P02150	50.21	-3.77	1.37	-2.76	5.77E-03	8.36E-02	WEB family protein At5g16730
Ma06_g14620	ITC1587_Bchr6_P15942	97.97	-3.79	0.77	-4.93	8.08E-07	4.79E-04	UPF0496 protein 4-like
Ma10_g23960	ITC1587_Bchr10_P31001	3.24	-3.79	1.34	-2.82	4.77E-03	7.58E-02	transcription factor bHLH82-like
Ma03_g12680	ITC1587_Bchr3_P06323	77.98	-3.8	0.74	-5.15	2.61E-07	2.29E-04	RPM1-interacting protein 4-like
Ma09_g31400	ITC1587_Bchr9_P28328	26.46	-3.85	1.22	-3.15	1.64E-03	4.22E-02	8-hydroxygeraniol dehydrogenase-like
Ma04_g33260	ITC1587_Bchr4_P11092	16.02	-3.85	1.37	-2.81	4.90E-03	7.64E-02	probable pectate lyase 8
Ma01_g16110	ITC1587_Bchr1_P02009	5.34	-3.86	0.95	-4.06	4.91E-05	5.60E-03	transcription repressor OFP7-like
Ma09_g06370	ITC1587_Bchr6_P17615*	15.86	-3.86	1.22	-3.16	1.55E-03	4.09E-02	transcription factor RF2b-like
Ma02_g24190	ITC1587_Bchr2_P05079	12.15	-3.89	1.11	-3.50	4.60E-04	2.03E-02	patatin-like protein 2
Ma06_g19420	ITC1587_Bchr6_P16384	6.06	-3.9	1.05	-3.71	2.06E-04	1.26E-02	uncharacterized LOC103988103
Ma10_g27780	ITC1587_Bchr7_P19431*	71.67	-3.91	0.98	-3.98	7.03E-05	6.80E-03	protodermal factor 1-like
Ma01_g17450	ITC1587_Bchr1_P02249	14.72	-3.92	0.82	-4.75	2.04E-06	7.95E-04	myb-related protein 305-like
Ma03_g07350	ITC1587_Bchr3_P05821	68.32	-3.92	1.26	-3.10	1.94E-03	4.59E-02	3-ketoacyl-CoA synthase 6
Ma03_g03150	ITC1587_Bchr3_P05447*	2.94	-3.93	1.41	-2.78	5.47E-03	8.15E-02	calcineurin B-like protein 7

Ma10_g30050	ITC1587_Bchr10_P31546	52.3	-3.95	1.13	-3.50	4.74E-04	2.05E-02	Ethylene-responsive transcription factor ERF071
Ma06_g27860	ITC1587_Bchr6_P17546	9.6	-3.96	1.27	-3.11	1.90E-03	4.54E-02	subtilisin-like protease Glyma18g48580
Ma10_g18410	ITC1587_Bchr10_P30528	3.69	-3.96	1.46	-2.72	6.55E-03	9.04E-02	putative protein TIFY 9
Ma04_g10500	ITC1587_Bchr4_P09322	45.02	-3.97	0.87	-4.54	5.72E-06	1.51E-03	L-gulonolactone oxidase
Ma08_g07730	ITC1587_Bchr8_P24897*	3.1	-3.97	1.4	-2.83	4.65E-03	7.50E-02	histone H3.2
Ma05_g13640	ITC1587_Bchr5_P12886	5.3	-3.97	1.14	-3.49	4.82E-04	2.08E-02	IQ calmodulin-binding motif family protein
Ma02_g17570	ITC1587_Bchr2_P04487	3.9	-4	1.35	-2.96	3.03E-03	5.94E-02	uncharacterized LOC103975432
Ma07_g19970	ITC1587_Bchr7_P20657	100.06	-4	0.91	-4.42	9.80E-06	2.16E-03	U-box domain-containing protein 21-like
Ma04_g34330	ITC1587_Bchr4_P11181	4.46	-4.01	1.33	-3.02	2.55E-03	5.33E-02	glucan endo-1, 3-beta-glucosidase 1-like
Ma07_g03320	ITC1587_Bchr7_P18846*	11.08	-4.02	0.77	-5.23	1.65E-07	1.68E-04	E3 ubiquitin-protein ligase PUB22
Ma05_g12190	ITC1587_Bchr5_P12767	37.25	-4.02	1.12	-3.57	3.50E-04	1.75E-02	cytochrome P450 86A2-like
Ma07_g08690	ITC1587_Bchr7_P19375	8.36	-4.02	0.94	-4.30	1.73E-05	2.91E-03	probable WRKY transcription factor 72
Ma03_g06210	ITC1587_Bchr3_P05720	13.45	-4.02	1.04	-3.88	1.03E-04	8.53E-03	protein NSP-INTERACTING KINASE 1-like
Ma10_g14940	ITC1587_Bchr2_P04868*	5.96	-4.03	1.05	-3.83	1.28E-04	9.65E-03	dynein light chain LC6
Ma08_g11720	ITC1587_Bchr8_P22508	12.34	-4.04	1.23	-3.28	1.03E-03	3.21E-02	dehydration-responsive element-binding protein 1G
Ma03_g14260	ITC1587_BchrUn_random_P35644	210.95	-4.05	1.47	-2.76	5.73E-03	8.32E-02	BAHD acyltransferase DCR
Ma07_g10420	ITC1587_Bchr7_P19526*	9.21	-4.09	1.24	-3.31	9.41E-04	3.04E-02	putative DNA-binding protein ESCAROLA
Ma08_g31940	ITC1587_Bchr8_P24803	10.86	-4.09	1.35	-3.02	2.54E-03	5.32E-02	N66 matrix protein-like
Ma06_g02940	ITC1587_Bchr6_P14888	123.68	-4.12	1.43	-2.88	3.94E-03	6.82E-02	Acyl-[acyl-carrier-protein] desaturase
Ma00_g00150	ITC1587_Bchr3_P06819	19.09	-4.12	0.75	-5.47	4.41E-08	7.35E-05	monothiol glutaredoxin-S9-like
Ma02_g17270	ITC1587_Bchr2_P04463	29.33	-4.14	1.4	-2.96	3.12E-03	6.02E-02	Peroxidase 52
Ma11_g02920	ITC1587_Bchr11_P31963	7.54	-4.18	1.42	-2.95	3.23E-03	6.15E-02	probable LRR receptor-like serine/threonine-protein kinase At4g36180
Ma06_g27800	ITC1587_Bchr6_P17542	82.06	-4.18	0.97	-4.33	1.47E-05	2.67E-03	GDSL esterase/lipase At5g45670-like
Ma07_g08830	ITC1587_Bchr7_P19389	51.34	-4.18	1.37	-3.06	2.22E-03	4.93E-02	cytokinin dehydrogenase 7-like
Ma07_g07670	ITC1587_Bchr7_P19248	5.11	-4.19	1.38	-3.03	2.45E-03	5.21E-02	calmodulin-like protein 8
Ma03_g17260	ITC1587_Bchr3_P07769*	28.57	-4.22	1.02	-4.12	3.76E-05	4.75E-03	splicing factor 3A subunit 2-like

Ma02_g08860	ITC1587_Bchr2_P03734	5.37	-4.23	1.43	-2.96	3.04E-03	5.95E-02	uncharacterized LOC103976145
Ma08_g12080	ITC1587_Bchr8_P22539	6.46	-4.23	1.31	-3.22	1.26E-03	3.62E-02	RING-H2 finger protein ATL1-like
Ma06_g08860	ITC1587_Bchr6_P15425	10.98	-4.23	1.47	-2.89	3.88E-03	6.78E-02	protein RALF-like 34
Ma01_g23540	ITC1587_Bchr1_P02826	38.52	-4.25	1.19	-3.57	3.51E-04	1.75E-02	protein trichome birefringence-like 26
Ma09_g14060	ITC1587_Bchr9_P26283	7.16	-4.26	1.5	-2.84	4.48E-03	7.32E-02	protein NSP-INTERACTING KINASE 1-like
Ma02_g11020	ITC1587_Bchr2_P03928	3.91	-4.27	1.44	-2.97	2.96E-03	5.88E-02	uncharacterized LOC103976363
Ma08_g20500	ITC1587_Bchr8_P23819	13.03	-4.31	1.51	-2.85	4.39E-03	7.26E-02	leucine-rich repeat extensin-like protein 6
Ma10_g28070	ITC1587_Bchr10_P31362	5.9	-4.32	1.52	-2.84	4.55E-03	7.42E-02	cytokinin dehydrogenase 6-like
Ma02_g16140	ITC1587_Bchr2_P04360*	16.41	-4.33	1.53	-2.83	4.61E-03	7.48E-02	polygalacturonase At1g48100-like
Ma10_g15350	ITC1587_Bchr10_P30268	12.14	-4.35	1.52	-2.86	4.21E-03	7.08E-02	glucan endo-1, 3-beta-glucosidase 3-like
Ma01_g17930	ITC1587_Bchr1_P02198	5.82	-4.37	1.6	-2.73	6.33E-03	8.83E-02	uncharacterized LOC103996578
Ma05_g29230	ITC1587_Bchr5_P14531*	10.22	-4.4	1.47	-2.99	2.78E-03	5.67E-02	putative AP2/ERF domain-containing transcription factor
Ma07_g08490	ITC1587_Bchr7_P19324	94.12	-4.43	1.42	-3.13	1.75E-03	4.36E-02	pleiotropic drug resistance protein 6
Ma09_g29840	ITC1587_Bchr9_P28197	9.74	-4.45	1.03	-4.31	1.60E-05	2.82E-03	vegetative cell wall protein gp1-like
Ma08_g11260	ITC1587_Bchr8_P22467	8.14	-4.46	0.9	-4.96	7.18E-07	4.35E-04	uncharacterized LOC103993249
Ma09_g02800	ITC1587_Bchr9_P25321	4.81	-4.47	1.43	-3.12	1.81E-03	4.44E-02	F-box protein SKIP27
Ma07_g04040	ITC1587_Bchr7_P18900	2.21	-4.48	1.47	-3.04	2.39E-03	5.14E-02	transcription factor bHLH93-like
Ma09_g08470	ITC1587_Bchr6_P17479*	10.6	-4.5	1.54	-2.91	3.56E-03	6.47E-02	serine decarboxylase 1-like
Ma04_g05500	ITC1587_Bchr4_P08875	11.15	-4.5	1.07	-4.21	2.55E-05	3.66E-03	Tubulin beta-1 chain
Ma06_g06480	ITC1587_Bchr6_P15218	380.29	-4.59	1.05	-4.38	1.20E-05	2.43E-03	putative nuclease HARBI1
Ma10_g02140	ITC1587_Bchr10_P28874	3.57	-4.61	1.27	-3.62	2.94E-04	1.55E-02	histone H2B.4
Ma03_g30090	ITC1587_Bchr3_P08065	46.17	-4.66	1.11	-4.21	2.55E-05	3.66E-03	mitogen-activated protein kinase kinase kinase A-like
Ma06_g13220	ITC1587_Bchr6_P15823	37.14	-4.68	1.75	-2.67	7.58E-03	9.75E-02	probable flavin-containing monooxygenase 1
Ma08_g28960	ITC1587_Bchr8_P24531*	4.53	-4.74	1.36	-3.49	4.90E-04	2.09E-02	transcription factor FAMA-like
Ma09_g22100	ITC1587_Bchr9_P27464	7.27	-4.74	1.62	-2.93	3.38E-03	6.25E-02	beta-galactosidase 5-like
Ma08_g16720	ITC1587_BchrUn_random_P38204	19.16	-4.75	1.2	-3.94	8.00E-05	7.34E-03	piriformospora indica-insensitive protein 2-like
Ma04_g21110	ITC1587_Bchr1_P00056	21.8	-4.77	1.26	-3.79	1.48E-04	1.05E-02	phospholipase A1-Ibeta2

Ma03_g16350	ITC1587_BchrUn_random_P34776	4.84	-4.78	1.61	-2.97	2.94E-03	5.87E-02	leucine-rich repeat extensin-like protein 3
Ma09_g13960	ITC1587_Bchr3_P06099*	18.09	-4.8	1.15	-4.19	2.80E-05	3.90E-03	nucleobase-ascorbate transporter 6-like
Ma07_g15500	ITC1587_Bchr7_P20008	190.19	-4.81	1.69	-2.85	4.40E-03	7.27E-02	putative cell wall protein
Ma08_g26890	ITC1587_BchrUn_random_P34776*	5	-4.84	1.79	-2.71	6.77E-03	9.21E-02	leucine-rich repeat extensin-like protein 4
Ma03_g30570	ITC1587_Bchr3_P08106	5.24	-4.84	1.58	-3.07	2.14E-03	4.85E-02	AP2/ERF and B3 domain-containing protein Os05g0549800-like
Ma05_g21260	ITC1587_Bchr5_P13753	49.82	-4.84	1.24	-3.9	9.44E-05	8.07E-03	butyrate--CoA ligase AAE11
Ma09_g25750	ITC1587_Bchr9_P27833	5.99	-4.85	1.38	-3.51	4.43E-04	1.98E-02	probable isoaspartyl peptidase/L-asparaginase 2
Ma08_g33150	ITC1587_Bchr8_P24897*	3.76	-4.87	1.79	-2.72	6.55E-03	9.04E-02	histone H3.2
Ma06_g34460	ITC1587_Bchr6_P18155*	7.19	-4.96	1.43	-3.47	5.24E-04	2.17E-02	cytosolic endo-beta-N-acetylglucosaminidase-like
Ma04_g12900	ITC1587_Bchr4_P09552	14.54	-4.97	1.27	-3.91	9.36E-05	8.03E-03	uncharacterized protein At4g06744-like
Ma03_g11030	ITC1587_Bchr1_P01745*	4.58	-4.97	1.56	-3.18	1.47E-03	3.95E-02	auxin-induced protein 6B
Ma03_g32600	ITC1587_Bchr4_P09327*	21.93	-4.98	1.38	-3.59	3.25E-04	1.67E-02	transcription factor RF2a-like
Ma06_g08540	ITC1587_Bchr8_P24778*	3.29	-5	1.68	-2.98	2.88E-03	5.79E-02	WW domain containing protein
Ma10_g17850	ITC1587_Bchr10_P30486	2.55	-5.02	1.49	-3.36	7.69E-04	2.70E-02	type I inositol 1
Ma01_g03800	ITC1587_Bchr1_P00772	25.9	-5.09	1.48	-3.44	5.92E-04	2.34E-02	probable xyloglucan endotransglucosylase/hydrolase protein 8
Ma09_g16290	ITC1587_Bchr9_P27181*	125.48	-5.1	1.26	-4.05	5.11E-05	5.71E-03	alpha-dioxygenase 1-like
Ma05_g02400	ITC1587_Bchr5_P11865	12.8	-5.19	1.39	-3.74	1.84E-04	1.19E-02	phospholipase A1-Igamma1
Ma04_g08060	ITC1587_Bchr4_P09085	3.18	-5.19	1.89	-2.75	5.93E-03	8.51E-02	interactor of constitutive active ROPs 1-like
Ma06_g31650	ITC1587_Bchr6_P17897	3.56	-5.20	1.67	-3.11	1.84E-03	4.47E-02	protein RESTRICTED TEV MOVEMENT 2-like
Ma08_g29760	ITC1587_Bchr8_P24598	12.85	-5.21	1.20	-4.35	1.33E-05	2.54E-03	protein NRT1/ PTR FAMILY 4.3-like
Ma07_g21450	ITC1587_Bchr7_P20803	22.94	-5.26	1.48	-3.55	3.86E-04	1.85E-02	snakin-2-like
Ma01_g14600	ITC1587_Bchr1_P01871	42.07	-5.30	0.93	-5.71	1.11E-08	3.24E-05	AP2/ERF and B3 domain-containing protein Os05g0549800-like
Ma03_g06060	ITC1587_Bchr3_P05707	4.47	-5.31	1.37	-3.88	1.05E-04	8.56E-03	CST complex subunit CTC1
Ma01_g04800	ITC1587_Bchr1_P00993	71.06	-5.34	1.04	-5.12	3.00E-07	2.48E-04	GDSL esterase/lipase At5g33370-like
Ma06_g25310	ITC1587_Bchr6_P17305	53.11	-5.37	1.38	-3.9	9.59E-05	8.12E-03	uncharacterized LOC103988981

Ma06_g16390	ITC1587_Bchr6_P16115	2.67	-5.38	1.99	-2.71	6.82E-03	9.24E-02	uncharacterized LOC103987843
Ma03_g07410	ITC1587_Bchr3_P05828	4.21	-5.43	1.59	-3.41	6.45E-04	2.47E-02	nudix hydrolase 17
Ma03_g28380	ITC1587_Bchr3_P07948	5.77	-5.62	1.85	-3.03	2.42E-03	5.18E-02	cytokinin hydroxylase-like
Ma06_g04670	ITC1587_Bchr6_P15048	19.67	-5.66	2.13	-2.67	7.70E-03	9.83E-02	LTPL126 - Protease inhibitor/seed storage/LTP family protein precursor
Ma03_g13420	ITC1587_BchrUn_random_P35146*	14.63	-5.78	0.81	-7.10	1.22E-12	3.24E-08	plant intracellular Ras-group-related LRR protein 1-like
Ma01_g13670	ITC1587_Bchr1_P01790*	2.79	-5.9	1.90	-3.11	1.84E-03	4.47E-02	Hypothetical protein
Ma09_g11440	ITC1587_Bchr9_P26051	137.1	-5.92	1.06	-5.60	2.18E-08	4.36E-05	isocitrate lyase
Ma09_g19380	ITC1587_Bchr4_P11321*	9.79	-5.92	1.54	-3.84	1.22E-04	9.32E-03	serine carboxypeptidase-like 35
Ma11_g15810	ITC1587_Bchr11_P33583	74.38	-5.94	1.37	-4.34	1.40E-05	2.62E-03	protein ECERIFERUM 1-like
Ma09_g10350	ITC1587_Bchr9_P25955	101.97	-5.96	1.75	-3.41	6.38E-04	2.46E-02	putative horcolin
Ma11_g13300	ITC1587_Bchr11_P33322	3.97	-5.98	1.88	-3.18	1.49E-03	4.00E-02	early nodulin-like protein 3
Ma04_g32080	ITC1587_Bchr4_P11001	6.86	-6.12	1.87	-3.28	1.04E-03	3.23E-02	GDSL esterase/lipase EXL3-like
Ma07_g27600	ITC1587_Bchr6_P17412*	3.07	-6.13	2.00	-3.06	2.23E-03	4.94E-02	uncharacterized LOC103992872
Ma01_g04650	ITC1587_Bchr1_P00980	24.29	-6.19	1.5	-4.13	3.67E-05	4.69E-03	3-ketoacyl-CoA synthase 12
Ma05_g23260	ITC1587_Bchr5_P13966	29.37	-6.2	2.21	-2.81	4.98E-03	7.69E-02	14 kDa proline-rich protein DC2.15
Ma04_g33640	ITC1587_Bchr4_P11121	2.17	-6.27	1.72	-3.64	2.69E-04	1.48E-02	uncharacterized LOC103982609
Ma03_g05830	ITC1587_Bchr3_P05688	5.75	-6.36	2.19	-2.91	3.66E-03	6.56E-02	ethylene-responsive transcription factor ERF016-like
Ma03_g21640	ITC1587_Bchr3_P07361	161.58	-6.56	1.49	-4.40	1.07E-05	2.30E-03	Peroxidase 72
Ma03_g12690	ITC1587_Bchr3_P06324	6.98	-6.59	1.68	-3.93	8.35E-05	7.58E-03	GDSL esterase/lipase At4g26790-like
Ma05_g00950		44.77	-6.7	1.70	-3.95	7.86E-05	7.24E-03	extensin-like
Ma08_g00630	ITC1587_Bchr8_P21529	14.13	-7.31	2.71	-2.70	6.94E-03	9.34E-02	bidirectional sugar transporter SWEET14-like
Ma04_g33390	ITC1587_Bchr4_P11102	20.49	-7.87	2.11	-3.73	1.94E-04	1.22E-02	14 kDa proline-rich protein DC2.15
Ma01_g13710	ITC1587_Bchr1_P01790	51.81	-8.48	1.43	-5.93	3.03E-09	1.35E-05	protein ECERIFERUM 1-like

Note: Gene IDs from *Musa balbisiana* are based on the results of the reciprocal best BLAST search. The best BLAST hit is reported for the genes where the reciprocal best BLAST search hit was not available and it is denoted with *.

Table S3: Complete list of genes differentially expressed in BXW-susceptible genotype Pisang Awak in response to *Xanthomonas campestris* pv. *musacearum* at 48 hpi.

Gene ID from *Musa acuminata* (DH Pahang)	Gene ID from *Musa balbisiana* (Pisang Klutuk Wulung)	BaseMean	Log2 Fold Change	lfcSE	Stat	Pvalue	Padj	Description
Ma01_g10670	ITC1587_Bchr1_P01520	65.59	1.46	0.37	3.99	6.59E-05	0.05	U-box domain-containing protein 33-like
Ma09_g06030	ITC1587_Bchr9_P25584	1605.14	1.4	0.27	5.21	1.89E-07	0	probable ubiquitin-conjugating enzyme E2 24
Ma08_g16960	ITC1587_BchrUn_random_P38061*	63.22	1.33	0.33	4.00	6.35E-05	0.05	Probable LRR receptor-like serine/threonine-protein kinase At1g56130
Ma04_g14080	ITC1587_BchrUn_random_P34764*	44.64	1.32	0.3	4.38	1.18E-05	0.03	myosin-11-like
Ma07_g16540	ITC1587_BchrUn_random_P38061	68.46	1.21	0.31	3.90	9.54E-05	0.05	probable LRR receptor-like serine/threonine-protein kinase At1g56140
Ma09_g13050	ITC1587_Bchr9_P26190	38.72	1.19	0.31	3.86	1.14E-04	0.06	protein GAMETE EXPRESSED 2
Ma07_g24330	ITC1587_Bchr7_P21046	90.44	1.08	0.3	3.62	2.96E-04	0.08	uncharacterized LOC103992585
Ma01_g08890	ITC1587_Bchr1_P01358	177.19	0.97	0.27	3.64	2.76E-04	0.08	uncharacterized LOC103984893
Ma03_g29980	ITC1587_Bchr3_P08046*	30.93	0.96	0.26	3.69	2.23E-04	0.08	uncharacterized LOC103979962
Ma10_g18840	ITC1587_Bchr10_P30560*	121.66	0.88	0.24	3.63	2.84E-04	0.08	myb-like protein Q
Ma02_g24410	ITC1587_Bchr2_P05097	181.61	0.86	0.23	3.75	1.76E-04	0.07	uncharacterized LOC103976670
Ma09_g07230	ITC1587_Bchr9_P25690	314.38	0.83	0.21	4.03	5.64E-05	0.04	uncharacterized LOC103997302
Ma01_g21900	ITC1587_BchrUn_random_P35539	430.58	0.81	0.15	5.29	1.25E-07	0	putative ATPase N2B
Ma04_g24750	ITC1587_Bchr1_P00475	276.62	0.78	0.2	3.85	1.18E-04	0.06	probable LRR receptor-like serine/threonine-protein kinase At1g53430
Ma11_g21640	ITC1587_Bchr11_P34110	774.32	0.75	0.18	4.1	4.08E-05	0.04	uncharacterized LOC103972199
Ma09_g11170	ITC1587_Bchr9_P26028	249.22	0.75	0.18	4.08	4.47E-05	0.04	ATP-dependent helicase rhp16
Ma10_g17070	ITC1587_Bchr10_P30418*	3082.33	0.74	0.18	4.16	3.21E-05	0.04	uncharacterized LOC103968471
Ma07_g22780	ITC1587_Bchr7_P20910	77.06	0.71	0.2	3.64	2.71E-04	0.08	nuclear control of ATPase protein 2-like

Ma06_g15040	ITC1587_Bchr6_P15983	379.67	0.67	0.17	3.96	7.37E-05	0.05	4-alpha-glucan-branching enzyme 3
Ma11_g12280	ITC1587_Bchr11_P33183	459.51	0.66	0.17	3.9	9.47E-05	0.05	peptide chain release factor 1-like
Ma09_g26050	ITC1587_Bchr9_P27859	126.77	0.65	0.18	3.6	3.15E-04	0.08	uncharacterized LOC103999400
Ma07_g17440	ITC1587_Bchr2_P03571*	1060.32	0.65	0.16	4.13	3.68E-05	0.04	calmodulin-binding receptor-like cytoplasmic kinase 3
Ma03_g07190	ITC1587_Bchr10_P30745*	1686.67	0.64	0.18	3.56	3.75E-04	0.09	translation factor GUF1 homolog
Ma10_g16090	ITC1587_Bchr10_P30328	1437.2	0.62	0.15	4.21	2.56E-05	0.04	dynamin-like protein ARC5
Ma11_g15300	ITC1587_Bchr5_P13764*	751.29	0.62	0.15	4.2	2.62E-05	0.04	prolyl endopeptidase-like
Ma05_g10740	ITC1587_Bchr5_P12637*	1007.99	0.62	0.17	3.68	2.35E-04	0.08	protein EXECUTER 1
Ma04_g38380	ITC1587_BchrUn_random_P39023	302.17	0.61	0.17	3.68	2.37E-04	0.08	phospholipid-transporting ATPase 2-like
Ma10_g09040	ITC1587_Bchr10_P29653	571.95	0.61	0.16	3.72	1.98E-04	0.07	uncharacterized LOC104000429
Ma03_g26960	ITC1587_Bchr3_P07815	340.29	0.61	0.13	4.66	3.14E-06	0.01	serine carboxypeptidase-like 2
Ma08_g16970	ITC1587_BchrUn_random_P37225	231.98	0.61	0.14	4.28	1.88E-05	0.04	UPF0613 protein PB24D3.06c
Ma07_g25100	ITC1587_Bchr7_P21121*	628.27	0.61	0.14	4.36	1.30E-05	0.03	2-C-methyl-D-erythritol 4-phosphate cytidylyltransferase
Ma10_g06980	ITC1587_Bchr11_P32484*	828.08	0.6	0.15	4.12	3.79E-05	0.04	zinc finger CCCH domain-containing protein ZFN-like
Ma04_g38370	ITC1587_Bchr4_P11519	290.13	0.59	0.15	3.96	7.41E-05	0.05	phospholipid-transporting ATPase 2-like
Ma02_g20180	ITC1587_Bchr2_P04723	336.29	-0.64	0.18	-3.61	3.04E-04	0.08	putative L-cysteine desulfhydrase 1
Ma09_g29480	ITC1587_Bchr6_P15289*	836.41	-0.68	0.17	-4.04	5.40E-05	0.04	ubiquitin-conjugating enzyme E2 10-like
Ma09_g17580	ITC1587_Bchr9_P26619*	83.71	-0.72	0.2	-3.61	3.12E-04	0.08	uncharacterized LOC103998293
Ma06_g03910	ITC1587_Bchr6_P14976	418.5	-0.73	0.2	-3.67	2.42E-04	0.08	uncharacterized LOC103986718
Ma09_g25550	ITC1587_Bchr9_P27813	64.56	-0.78	0.21	-3.65	2.58E-04	0.08	uncharacterized LOC103998907
Ma06_g27510	ITC1587_Bchr6_P17517	315.21	-0.81	0.16	-5.06	4.25E-07	0	thioredoxin Y
Ma02_g20390	ITC1587_Bchr2_P04737*	159.39	-0.82	0.23	-3.62	2.99E-04	0.08	uncharacterized LOC103974800
Ma06_g05670	ITC1587_Bchr6_P15153	529.95	-0.82	0.2	-4.18	2.91E-05	0.04	uncharacterized LOC103986919
Ma11_g17090	ITC1587_Bchr11_P33698	310.78	-0.83	0.22	-3.78	1.59E-04	0.06	uncharacterized LOC103971804
Ma06_g19380	ITC1587_Bchr6_P15426*	213.69	-0.83	0.21	-3.97	7.05E-05	0.05	ubiquitin-fold modifier 1
Ma09_g27850	ITC1587_Bchr9_P28014	188.52	-0.87	0.23	-3.75	1.80E-04	0.07	probable peroxygenase 4

Ma03_g30360	ITC1587_Bchr3_P08084	175.65	-0.88	0.22	-3.92	8.71E-05	0.05	probable ribose-5-phosphate isomerase 2
Ma11_g05280	ITC1587_Bchr5_P14016*	400.07	-0.89	0.25	-3.58	3.37E-04	0.08	uncharacterized LOC103970471
Ma10_g05050	ITC1587_Bchr10_P28426	200.45	-0.92	0.26	-3.55	3.79E-04	0.09	uncharacterized LOC103999623
Ma08_g02190	ITC1587_Bchr8_P21678	282.4	-0.92	0.24	-3.78	1.55E-04	0.06	missing_product
Ma06_g10380	ITC1587_Bchr6_P15562*	440.29	-0.93	0.23	-3.99	6.50E-05	0.05	ABC transporter G family member 20-like
Ma09_g09130	ITC1587_Bchr9_P25848	168.96	-0.98	0.27	-3.59	3.33E-04	0.08	WPP domain-containing protein 1-like
Ma06_g24330	ITC1587_Bchr6_P17241	290.54	-1.03	0.27	-3.84	1.22E-04	0.06	gamma-glutamyltranspeptidase 3-like
Ma06_g35390	ITC1587_BchrUn_random_P39343	59.51	-1.05	0.27	-3.94	8.29E-05	0.05	uncharacterized LOC103972734
Ma04_g13930	ITC1587_Bchr4_P09646	103.94	-1.05	0.28	-3.74	1.83E-04	0.07	E3 ubiquitin ligase BIG BROTHER-like
Ma06_g18220	ITC1587_Bchr10_P29966*	51.87	-1.07	0.28	-3.9	9.58E-05	0.05	uncharacterized LOC103988005
Ma05_g15720	ITC1587_BchrUn_random_P39159	42.32	-1.08	0.3	-3.61	3.01E-04	0.08	coiled-coil domain-containing protein 124-like
Ma11_g01930	ITC1587_Bchr11_P31866	110.42	-1.12	0.31	-3.68	2.29E-04	0.08	probable acetyltransferase NATA1-like
Ma02_g24750	ITC1587_Bchr2_P05125	137.24	-1.23	0.33	-3.75	1.75E-04	0.07	protein MIZU-KUSSEI 1-like
Ma03_g01580	ITC1587_Bchr3_P05308	76.61	-1.24	0.33	-3.79	1.51E-04	0.06	ras-related protein Rab11D-like
Ma05_g11310	ITC1587_Bchr5_P12686	111.66	-1.29	0.31	-4.15	3.36E-05	0.04	hippocampus abundant transcript-like protein 1
Ma06_g03350	ITC1587_Bchr6_P14922*	120.34	-1.39	0.38	-3.64	2.70E-04	0.08	uncharacterized LOC103986672
Ma03_g22770	ITC1587_Bchr3_P07466	59.83	-1.43	0.37	-3.89	9.87E-05	0.05	F-box protein At3g26010-like
Ma07_g22220	ITC1587_Bchr7_P20861	58.08	-1.44	0.39	-3.68	2.34E-04	0.08	protein MIZU-KUSSEI 1-like
Ma03_g02870	ITC1587_Bchr3_P05424	32.55	-1.45	0.4	-3.64	2.72E-04	0.08	uncharacterized LOC103977065
Ma11_g22300	ITC1587_Bchr11_P34165	56.8	-1.48	0.4	-3.67	2.45E-04	0.08	mannose-specific lectin-like
Ma03_g18730	ITC1587_BchrUn_random_P38210	145.06	-1.5	0.4	-3.77	1.64E-04	0.06	probable protein phosphatase 2C 68
Ma10_g22650	ITC1587_Bchr10_P30894*	35.71	-1.51	0.36	-4.14	3.44E-05	0.04	outer envelope pore protein 16
Ma10_g13960	ITC1587_Bchr10_P30136	206.06	-1.55	0.4	-3.88	1.04E-04	0.05	glycerol-3-phosphate 2-O-acyltransferase 6-like
Ma02_g18860	ITC1587_Bchr2_P04598	18.01	-1.55	0.41	-3.81	1.38E-04	0.06	EG45-like domain containing protein

Ma03_g25930	ITC1587_Bchr3_P07724*	624.9	-1.55	0.41	-3.82	1.31E-04	0.06	uncharacterized LOC103978898
Ma02_g19880	ITC1587_Bchr2_P04697	56.78	-1.66	0.35	-4.81	1.50E-06	0.01	non-specific phospholipase C4-like
Ma09_g01330	ITC1587_Bchr9_P25183	42.28	-1.67	0.35	-4.71	2.45E-06	0.01	uncharacterized LOC103996657
Ma04_g10560	ITC1587_Bchr4_P09329	31.22	-1.7	0.4	-4.22	2.47E-05	0.04	uncharacterized LOC103981121
Ma05_g16520	ITC1587_BchrUn_random_P34967	22.6	-1.72	0.47	-3.66	2.54E-04	0.08	no apical meristem protein
Ma05_g13510		34.32	-1.73	0.41	-4.18	2.86E-05	0.04	Hypothetical protein
Ma04_g36440	ITC1587_Bchr4_P11370	76.3	-1.74	0.48	-3.61	3.06E-04	0.08	cytosolic sulfotransferase 6-like
Ma09_g11790	ITC1587_Bchr9_P26085	24.29	-1.93	0.47	-4.12	3.75E-05	0.04	wound induced protein
Ma07_g18600	ITC1587_Bchr7_P20730*	56.89	-2	0.48	-4.16	3.14E-05	0.04	premnaspirodiene oxygenase-like
Ma04_g02740	ITC1587_Bchr4_P08641	14.43	-2.04	0.49	-4.12	3.79E-05	0.04	uncharacterized LOC103980228
Ma06_g21300	ITC1587_BchrUn_random_P39709	33.44	-2.04	0.52	-3.94	8.00E-05	0.05	protein G1-like1
Ma03_g26370	ITC1587_Bchr3_P07764	17.49	-2.28	0.6	-3.77	1.62E-04	0.06	phospholipase A1-II 1
Ma10_g27880	ITC1587_Bchr10_P31344	68.99	-2.49	0.61	-4.09	4.32E-05	0.04	zinc finger CCCH domain-containing protein 2-like
Ma07_g20770	ITC1587_Bchr7_P20730	16.41	-2.52	0.66	-3.8	1.46E-04	0.06	premnaspirodiene oxygenase-like
Ma01_g14490	ITC1587_Bchr1_P01860	965.93	-2.57	0.62	-4.12	3.84E-05	0.04	18.5 kDa class I heat shock protein
Ma04_g37530	ITC1587_Bchr4_P11456	43.99	-3.43	0.94	-3.66	2.53E-04	0.08	heat stress transcription factor C-2a-like
Ma02_g01380		4.81	-3.8	0.97	-3.91	9.35E-05	0.05	Hypothetical protein
Ma05_g03150	ITC1587_Bchr5_P11943	32.04	-4.2	1.17	-3.58	3.48E-04	0.09	protein HOTHEAD-like
Ma07_g02370	ITC1587_Bchr7_P18768	8.62	-4.43	1.17	-3.79	1.53E-04	0.06	type I inositol 1
Ma09_g19240	ITC1587_BchrUn_random_P36290	12.3	-18.29	3.58	-5.11	3.18E-07	0	GDSL esterase/lipase At5g33370-like

Note: Gene IDs from *Musa balbisiana* are based on the results of the reciprocal best BLAST search. The best BLAST hit is reported for the genes where the reciprocal best BLAST search hit was not available and it is denoted with *.

Table S4: Complete list of genes differentially expressed in BXW-resistant wild type banana *Musa balbisiana* in response to *Xanthomonas campestris* pv. *musacearum* at 48 hpi.

Gene ID from *Musa acuminata* (DH Pahang)	Gene ID from *Musa balbisiana* (Pisang Klutuk Wulung)	BaseMean	Log2 Fold Change	lfcSE	Stat	Pvalue	Padj	Description
Ma05_g23260	ITC1587_Bchr5_P13966	29.37	17.59	2.55	6.89	5.67E-12	1.34E-07	14 kDa proline-rich protein DC2.15
Ma05_g07110	ITC1587_Bchr5_P12317	18.35	17.27	2.91	5.94	2.88E-09	3.40E-05	putative cell wall protein
Ma02_g20530	ITC1587_Bchr2_P04749	23.31	16.16	3.73	4.33	1.47E-05	1.49E-02	Germin-like protein 8-14
Ma11_g18240	ITC1587_Bchr11_P33802	82.41	14.29	2.73	5.24	1.58E-07	9.33E-04	non-specific lipid-transfer protein-like
Ma02_g09860	ITC1587_Bchr2_P03815	224.1	8.03	1.71	4.71	2.51E-06	7.31E-03	uncharacterized protein C24B11.05-like
Ma06_g01320	ITC1587_Bchr11_P32703	32.3	6.78	1.66	4.07	4.67E-05	2.12E-02	putative auxin efflux carrier component 8
Ma02_g12010	ITC1587_Bchr2_P04009	11.75	5.98	1.59	3.77	1.63E-04	4.08E-02	heavy metal-associated isoprenylated plant protein 26
Ma07_g22430		11.83	5.41	1.44	3.76	1.68E-04	4.12E-02	hypothetical protein
Ma03_g16020	ITC1587_Bchr3_P06748	12.35	5.25	1.33	3.94	8.24E-05	3.07E-02	uncharacterized LOC103978341
Ma07_g12820	ITC1587_Bchr10_P31102	24	4.37	1.17	3.73	1.91E-04	4.46E-02	EF hand family protein
Ma03_g02250	ITC1587_Bchr3_P05368*	17.82	3.99	1.13	3.53	4.12E-04	6.61E-02	uncharacterized LOC103977124
Ma08_g04050	ITC1587_Bchr8_P21808	92.76	3.76	1.13	3.32	8.90E-04	9.00E-02	putative probable 3-ketoacyl-CoA synthase 2
Ma04_g02930	ITC1587_Bchr4_P08659	72.77	3.28	0.76	4.29	1.79E-05	1.55E-02	glucose-1-phosphate adenylyltransferase large subunit 1-like
Ma01_g17260	ITC1587_Bchr1_P02266	13.24	3.19	0.97	3.28	1.04E-03	9.39E-02	putative Transcription factor MYB86
Ma10_g26390	ITC1587_Bchr10_P31208	150.78	3.04	0.79	3.85	1.18E-04	3.58E-02	aluminum-activated malate transporter 9-like
Ma08_g29620	ITC1587_Bchr8_P24584*	17.4	3.02	0.86	3.53	4.16E-04	6.63E-02	22.0 kDa class IV heat shock protein-like
Ma03_g10020	ITC1587_Bchr3_P06090*	32.76	2.82	0.8	3.51	4.48E-04	6.99E-02	uncharacterized LOC103977822
Ma02_g10140	ITC1587_Bchr2_P03841*	80.57	2.59	0.61	4.21	2.51E-05	1.56E-02	zinc finger Ran-binding domain-containing protein 2-like
Ma02_g11000	ITC1587_Bchr2_P03925*	17.12	2.52	0.77	3.28	1.04E-03	9.39E-02	uncharacterized LOC103975973
Ma05_g29440	ITC1587_Bchr11_P31698*	58.55	2.5	0.75	3.35	8.06E-04	8.78E-02	uncharacterized LOC103986283
Ma03_g20120	ITC1587_BchrUn_random_P38266	20.25	2.18	0.65	3.38	7.27E-04	8.51E-02	ankyrin repeat protein SKIP35-like

Ma06_g14360	ITC1587_Bchr6_P15924	248.59	1.94	0.5	3.9	9.75E-05	3.17E-02	lipase-like PAD4
Ma03_g16030	ITC1587_Bchr3_P06749	109.65	1.92	0.58	3.29	9.89E-04	9.32E-02	growth-regulating factor 6
Ma01_g18330	ITC1587_Bchr1_P02164	373.44	1.92	0.54	3.55	3.91E-04	6.40E-02	trihelix transcription factor GTL1-like
Ma03_g20500	ITC1587_Bchr3_P07270	25.4	1.86	0.53	3.49	4.82E-04	7.16E-02	LRR receptor-like serine/threonine-protein kinase RPK2
Ma04_g39220	ITC1587_Bchr4_P11583	98.18	1.86	0.56	3.33	8.65E-04	8.97E-02	probable receptor-like protein kinase At5g61350
Ma11_g24690	ITC1587_Bchr11_P34357	62.94	1.83	0.56	3.28	1.02E-03	9.39E-02	patellin-3-like
Ma06_g04740		23.93	1.82	0.52	3.5	4.64E-04	7.09E-02	hypothetical protein
Ma08_g14070	ITC1587_Bchr8_P22634	47.76	1.79	0.54	3.32	9.03E-04	9.00E-02	mechanosensitive ion channel protein 6-like
Ma02_g24230	ITC1587_Bchr2_P05084*	51.88	1.67	0.47	3.53	4.09E-04	6.61E-02	polcalcin Phl p 7-like
Ma06_g29630	ITC1587_Bchr3_P07249*	52.66	1.58	0.45	3.51	4.51E-04	6.99E-02	probable importin-7 homolog
Ma09_g10290	ITC1587_Bchr9_P25949	107.77	1.51	0.4	3.75	1.78E-04	4.27E-02	uncharacterized LOC103997570
Ma05_g01810	ITC1587_Bchr5_P11814*	119.41	1.49	0.39	3.87	1.10E-04	3.41E-02	magnesium transporter MRS2-3
Ma09_g04920		29.13	1.49	0.44	3.38	7.37E-04	8.52E-02	hypothetical protein
Ma09_g29570	ITC1587_Bchr9_P28175	87.48	1.46	0.37	3.97	7.20E-05	2.88E-02	putative Tyrosine aminotransferase
Ma06_g15030	ITC1587_Bchr6_P15980	517.37	1.46	0.45	3.27	1.07E-03	9.51E-02	allene oxide synthase
Ma03_g21160	ITC1587_Bchr3_P07325	94.75	1.44	0.42	3.44	5.82E-04	7.74E-02	3-ketodihydrosphingosine reductase-like
Ma08_g20370	ITC1587_Bchr8_P23835	328.23	1.41	0.37	3.86	1.14E-04	3.50E-02	auxin-induced protein 22D-like
Ma09_g12980	ITC1587_Bchr9_P26183	54.68	1.39	0.42	3.32	9.06E-04	9.00E-02	putative leucine-rich repeat receptor-like serine/threonine-protein kinase At2g24130
Ma03_g11520	ITC1587_Bchr3_P06219	6097.91	1.34	0.31	4.28	1.88E-05	1.55E-02	linoleate 13S-lipoxygenase 2-1
Ma07_g04810	ITC1587_Bchr7_P18970	174.51	1.34	0.4	3.31	9.35E-04	9.13E-02	transcription factor MYC4-like
Ma08_g21300	ITC1587_Bchr7_P19893*	29.84	1.32	0.4	3.32	8.89E-04	9.00E-02	Casein kinase I isoform delta-like
Ma07_g24250	ITC1587_BchrUn_random_P37476*	506.07	1.31	0.4	3.3	9.60E-04	9.24E-02	uncharacterized LOC103992577
Ma10_g20730	ITC1587_Bchr10_P30723	57.36	1.28	0.37	3.47	5.15E-04	7.23E-02	L-tryptophan--pyruvate aminotransferase 1-like
Ma08_g23410	ITC1587_Bchr8_P24029	50.52	1.28	0.38	3.33	8.60E-04	8.97E-02	protein Brevis radix-like 1
Ma03_g19080	ITC1587_Bchr3_P07100	32.82	1.27	0.36	3.56	3.77E-04	6.29E-02	Superoxide dismutase [Mn] 3.1
Ma05_g10880	ITC1587_Bchr5_P12649	120.08	1.22	0.34	3.59	3.30E-04	5.98E-02	uncharacterized LOC103984583

Ma06_g04970	ITC1587_Bchr6_P15080	37.78	1.21	0.36	3.34	8.36E-04	8.91E-02	probable mitochondrial adenine nucleotide transporter BTL3
Ma02_g22370	ITC1587_Bchr2_P04925	93.59	1.21	0.29	4.18	2.86E-05	1.57E-02	uncharacterized LOC103976537
Ma01_g20630	ITC1587_Bchr1_P02550*	50.11	1.19	0.33	3.64	2.70E-04	5.30E-02	Hypothetical protein
Ma11_g07650	ITC1587_Bchr6_P14889*	35.92	1.18	0.34	3.49	4.89E-04	7.16E-02	uncharacterized LOC103970676
Ma02_g24350	ITC1587_Bchr2_P05093	264.81	1.18	0.36	3.28	1.04E-03	9.39E-02	DELLA protein SLR1-like
Ma11_g22910	ITC1587_Bchr11_P34211	102.26	1.17	0.32	3.65	2.62E-04	5.22E-02	[Fructose-bisphosphate aldolase]-lysine N-methyltransferase
Ma03_g22330	ITC1587_Bchr3_P07422	61.01	1.17	0.3	3.9	9.65E-05	3.17E-02	UBP1-associated protein 2B-like
Ma11_g22200	ITC1587_Bchr11_P34157	157.28	1.13	0.31	3.69	2.26E-04	4.87E-02	uncharacterized LOC103972256
Ma08_g02290	ITC1587_Bchr10_P28441*	174.4	1.12	0.34	3.28	1.05E-03	9.43E-02	delta-1-pyrroline-5-carboxylate synthase-like
Ma07_g14180	ITC1587_Bchr7_P19878	103.75	1.07	0.33	3.27	1.07E-03	9.51E-02	uncharacterized LOC103991649
Ma10_g15490	ITC1587_Bchr10_P30279	312.88	1.05	0.32	3.28	1.03E-03	9.39E-02	BTB/POZ domain-containing protein At3g44820
Ma09_g26260	ITC1587_Bchr9_P27875	218.34	1.05	0.27	3.87	1.10E-04	3.41E-02	glucan endo-1
Ma09_g03660	ITC1587_Bchr9_P25389	73.46	1.01	0.26	3.81	1.36E-04	3.71E-02	uncharacterized LOC103996880
Ma09_g24360	ITC1587_Bchr9_P27692	128.72	1	0.23	4.27	1.97E-05	1.55E-02	glutamate receptor 3.3-like
Ma07_g20630	ITC1587_BchrUn_random_P36840*	334.02	0.95	0.26	3.7	2.15E-04	4.78E-02	phosphopantothenate--cysteine ligase 2-like
Ma05_g21410	ITC1587_Bchr5_P12648*	134.8	0.94	0.29	3.28	1.03E-03	9.39E-02	protein S-acyltransferase 10-like
Ma00_g01860	ITC1587_BchrUn_random_P36249	76.6	0.94	0.28	3.41	6.58E-04	8.21E-02	uncharacterized LOC103973645
Ma06_g36360	ITC1587_Bchr6_P18305	262.61	0.93	0.2	4.61	3.98E-06	7.83E-03	uncharacterized LOC103990140
Ma06_g35270	ITC1587_Bchr6_P18218	46.92	0.93	0.28	3.3	9.81E-04	9.31E-02	E3 ubiquitin-protein ligase SDIR1-like
Ma06_g05210	ITC1587_Bchr6_P15106	67.51	0.92	0.25	3.7	2.19E-04	4.82E-02	putative ZOS4-07 - C2H2 zinc finger protein
Ma07_g02900	ITC1587_Bchr7_P18812	168.76	0.92	0.27	3.43	5.94E-04	7.74E-02	CMP-sialic acid transporter 3-like
Ma11_g19850	ITC1587_Bchr11_P33948	149.7	0.91	0.21	4.24	2.26E-05	1.55E-02	protein FIZZY-RELATED 2-like
Ma11_g02180	ITC1587_Bchr11_P31893	294.02	0.9	0.27	3.32	9.01E-04	9.00E-02	uncharacterized LOC103970212
Ma06_g23730	ITC1587_Bchr6_P16911	47.62	0.89	0.27	3.26	1.10E-03	9.68E-02	uncharacterized LOC103988737
Ma09_g03630	ITC1587_Bchr9_P25386	123.38	0.89	0.25	3.62	2.89E-04	5.59E-02	UPF0420 protein
Ma11_g24420	ITC1587_Bchr11_P34335	40.17	0.89	0.25	3.58	3.40E-04	6.07E-02	putative uncharacterized protein

								DDB_G0277003
Ma11_g01300	ITC1587_Bchr11_P31805	188.3	0.88	0.27	3.32	9.03E-04	9.00E-02	bromodomain-containing protein 9-like
Ma11_g02290	ITC1587_Bchr11_P31905	676.88	0.85	0.24	3.57	3.62E-04	6.28E-02	ABC transporter C family member 5-like
Ma06_g11950	ITC1587_Bchr2_P05058*	268.31	0.85	0.18	4.75	2.06E-06	7.31E-03	probable protein phosphatase 2C 71
Ma09_g16130	ITC1587_BchrUn_random_ P36803	79.64	0.84	0.24	3.48	4.93E-04	7.16E-02	AP2-like ethylene-responsive transcription factor At2g41710
Ma09_g23510	ITC1587_Bchr9_P27604*	564.69	0.83	0.25	3.36	7.87E-04	8.71E-02	V-type proton ATPase catalytic subunit A
Ma01_g03450	ITC1587_Bchr1_P00739	183.25	0.83	0.2	4.19	2.76E-05	1.57E-02	molybdate-anion transporter
Ma09_g07100	ITC1587_Bchr9_P25677	1154.06	0.83	0.19	4.38	1.21E-05	1.44E-02	phosphoglucan
Ma08_g05390	ITC1587_Bchr5_P14113*	468.31	0.82	0.25	3.32	8.88E-04	9.00E-02	V-type proton ATPase subunit B 2
Ma11_g03560	ITC1587_Bchr11_P32024	242.3	0.82	0.23	3.49	4.84E-04	7.16E-02	tubulin-folding cofactor D
Ma01_g20120	ITC1587_Bchr1_P02354	189.1	0.8	0.25	3.26	1.12E-03	9.73E-02	formin-like protein 6
Ma11_g04510	ITC1587_Bchr11_P32121	527.45	0.8	0.25	3.26	1.12E-03	9.74E-02	uncharacterized protein At3g06530
Ma04_g13890	ITC1587_Bchr4_P09643	506.33	0.79	0.21	3.69	2.25E-04	4.87E-02	zinc finger CCCH domain-containing protein 24-like
Ma00_g00040	ITC1587_BchrUn_random_ P37648	89.16	0.78	0.2	3.93	8.35E-05	3.07E-02	tobamovirus multiplication protein 1-like
Ma05_g26770	ITC1587_Bchr5_P14308	132.44	0.76	0.19	4.07	4.68E-05	2.12E-02	DNA mismatch repair protein MSH6
Ma09_g00910	ITC1587_Bchr9_P25142	192.69	0.76	0.2	3.84	1.21E-04	3.61E-02	uncharacterized LOC103996620
Ma07_g00090	ITC1587_Bchr7_P18560	317.98	0.76	0.22	3.38	7.29E-04	8.51E-02	B3 domain-containing protein Os07g0563300-like
Ma07_g22660	ITC1587_Bchr7_P20902	135.21	0.76	0.23	3.28	1.02E-03	9.39E-02	dual specificity protein phosphatase 1B-like
Ma02_g18580	ITC1587_Bchr2_P04575	657.07	0.75	0.22	3.36	7.92E-04	8.72E-02	uncharacterized LOC103975341
Ma04_g34730	ITC1587_Bchr4_P11215*	147.66	0.75	0.23	3.29	1.02E-03	9.39E-02	uncharacterized LOC103983378
Ma10_g26450	ITC1587_Bchr10_P31213	106.61	0.71	0.21	3.34	8.32E-04	8.91E-02	protein NEDD1
Ma04_g24930	ITC1587_Bchr6_P18223*	285.07	0.7	0.2	3.48	5.02E-04	7.17E-02	ELF3 protein
Ma03_g28310	ITC1587_Bchr3_P07940	151.12	0.7	0.21	3.32	8.96E-04	9.00E-02	katanin p80 WD40 repeat-containing subunit B1 homolog
Ma08_g29410	ITC1587_Bchr8_P24564	101.92	0.69	0.2	3.4	6.74E-04	8.25E-02	uncharacterized LOC103996469
Ma02_g02010	ITC1587_BchrUn_random_ P34585	763.86	0.68	0.16	4.33	1.52E-05	1.49E-02	mitochondrial Rho GTPase 1-like
Ma10_g26810	ITC1587_Bchr10_P31249	187.36	0.66	0.2	3.37	7.62E-04	8.60E-02	uncharacterized LOC103969330

Ma11_g10100	ITC1587_Bchr3_P06302*	366.47	0.66	0.19	3.42	6.25E-04	7.98E-02	probable ATP-dependent RNA helicase DHX35
Ma03_g13750	ITC1587_BchrUn_random_P35115	156.84	0.66	0.19	3.47	5.14E-04	7.23E-02	uncharacterized LOC103973109
Ma07_g24660	ITC1587_Bchr8_P22150*	113.08	0.64	0.18	3.47	5.13E-04	7.23E-02	E3 ubiquitin-protein ligase RNF170-like
Ma08_g07810	ITC1587_Bchr3_P07526*	814.95	0.62	0.15	4.19	2.85E-05	1.57E-02	polypyrimidine tract-binding protein homolog 3-like
Ma11_g21080	ITC1587_Bchr11_P34062	527.23	0.61	0.18	3.41	6.57E-04	8.21E-02	uncharacterized LOC103972157
Ma02_g03660	ITC1587_Bchr2_P03254	1390.01	0.6	0.17	3.46	5.32E-04	7.28E-02	glyoxysomal fatty acid beta-oxidation multifunctional protein MFP-a-like
Ma08_g14500	ITC1587_Bchr8_P23715	131.89	0.6	0.17	3.57	3.54E-04	6.22E-02	translocase of chloroplast 120
Ma07_g23520	ITC1587_Bchr11_P33804*	1921.58	0.59	0.17	3.37	7.60E-04	8.60E-02	vacuolar-sorting receptor 1
Ma11_g23320	ITC1587_Bchr11_P34241	361.61	0.59	0.18	3.31	9.41E-04	9.13E-02	mediator of RNA polymerase II transcription subunit 33A-like
Ma04_g07390	ITC1587_Bchr4_P09036	364.59	-0.59	0.18	-3.34	8.26E-04	8.91E-02	uncharacterized LOC103980853
Ma09_g28560	ITC1587_Bchr9_P28079	306.14	-0.59	0.17	-3.43	6.05E-04	7.84E-02	ras-related protein RABB1c-like
Ma03_g20310	ITC1587_Bchr9_P25242*	386.67	-0.6	0.15	-3.9	9.70E-05	3.17E-02	Probable cytochrome b5 isoform 2
Ma04_g10390	ITC1587_Bchr4_P09116*	298.32	-0.6	0.16	-3.72	1.98E-04	4.57E-02	ER lumen protein-retaining receptor A-like
Ma09_g03890	ITC1587_Bchr1_P01513*	728.87	-0.6	0.17	-3.57	3.63E-04	6.28E-02	oxysterol-binding protein-related protein 3A-like
Ma09_g05450	ITC1587_Bchr9_P25535	292.88	-0.61	0.17	-3.6	3.22E-04	5.92E-02	ras-related protein Rab11D
Ma04_g03620	ITC1587_Bchr4_P08710	234.62	-0.62	0.17	-3.64	2.72E-04	5.30E-02	mitochondrial import inner membrane translocase subunit TIM22-3-like
Ma08_g01280	ITC1587_Bchr8_P21588	663.93	-0.62	0.14	-4.34	1.40E-05	1.49E-02	F-box/kelch-repeat protein SKIP11-like
Ma09_g07230	ITC1587_Bchr9_P25690	314.38	-0.64	0.2	-3.3	9.84E-04	9.31E-02	uncharacterized LOC103997302
Ma11_g10060	ITC1587_Bchr9_P27483*	392.86	-0.65	0.16	-3.95	7.84E-05	3.03E-02	clavaminate synthase-like protein At3g21360
Ma05_g03790	ITC1587_Bchr5_P12009	565.72	-0.65	0.18	-3.66	2.54E-04	5.22E-02	peptidyl-tRNA hydrolase ICT1
Ma06_g04590	ITC1587_Bchr6_P15040	190.86	-0.65	0.17	-3.88	1.05E-04	3.34E-02	Coiled-coil domain-containing protein 72
Ma04_g02710	ITC1587_Bchr4_P08638	289.38	-0.66	0.17	-3.81	1.37E-04	3.71E-02	shaggy-related protein kinase eta
Ma04_g10470	ITC1587_Bchr4_P09318	119.76	-0.66	0.19	-3.38	7.20E-04	8.51E-02	AP-4 complex subunit sigma
Ma10_g24020	ITC1587_Bchr10_P31007	137.81	-0.66	0.17	-3.83	1.28E-04	3.71E-02	DCN1-like protein 4
Ma02_g08630	ITC1587_Bchr8_P24756*	385.98	-0.69	0.2	-3.49	4.83E-04	7.16E-02	phosphoglucomutase

Ma03_g02530	ITC1587_Bchr3_P05396*	223.19	-0.71	0.17	-4.15	3.28E-05	1.72E-02	protein phosphatase inhibitor 2-like
Ma05_g24920	ITC1587_Bchr5_P14142	198.59	-0.72	0.16	-4.37	1.23E-05	1.44E-02	uncharacterized LOC103985495
Ma07_g28530	ITC1587_Bchr4_P08676*	695.39	-0.72	0.2	-3.55	3.80E-04	6.30E-02	uncharacterized LOC103993105
Ma04_g08680	ITC1587_Bchr4_P09148	486.79	-0.74	0.16	-4.5	6.91E-06	1.05E-02	vesicle transport v-SNARE 13-like
Ma04_g05360	ITC1587_Bchr4_P08862	222.53	-0.75	0.19	-3.9	9.61E-05	3.17E-02	transcription factor ILR3-like
Ma04_g11640	ITC1587_Bchr4_P09437	201.99	-0.75	0.19	-4.02	5.86E-05	2.46E-02	vacuolar protein-sorting-associated protein 37 homolog 1-like
Ma04_g06320	ITC1587_Bchr4_P08946	153.12	-0.78	0.24	-3.27	1.07E-03	9.51E-02	vacuolar protein sorting-associated protein 32 homolog 2-like
Ma02_g24690	ITC1587_Bchr2_P05121	640.98	-0.78	0.22	-3.62	2.94E-04	5.64E-02	glycosyltransferase
Ma11_g06120	ITC1587_Bchr11_P32279	378.44	-0.81	0.23	-3.53	4.09E-04	6.61E-02	uncharacterized LOC103970545
Ma04_g12330	ITC1587_Bchr4_P09504	178.89	-0.82	0.22	-3.74	1.84E-04	4.33E-02	putative DnaJ homolog subfamily B member 1
Ma04_g07230	ITC1587_Bchr4_P09022	636.56	-0.83	0.18	-4.59	4.52E-06	8.19E-03	uncharacterized LOC103980838
Ma06_g03340	ITC1587_Bchr6_P14921	1710.18	-0.84	0.23	-3.56	3.72E-04	6.28E-02	molybdate transporter 2
Ma01_g02340	ITC1587_Bchr1_P00642*	2621.9	-0.85	0.23	-3.71	2.05E-04	4.61E-02	protein BPS1
Ma04_g26080	ITC1587_Bchr4_P10452	83.41	-0.86	0.24	-3.51	4.47E-04	6.99E-02	ubiquitin-conjugating enzyme E2-17 kDa-like
Ma01_g21420		888	-0.87	0.23	-3.82	1.33E-04	3.71E-02	uncharacterized LOC103999804
Ma02_g21100	ITC1587_Bchr2_P04798	355.51	-0.89	0.27	-3.3	9.52E-04	9.20E-02	uncharacterized LOC103976420
Ma04_g08640	ITC1587_Bchr4_P09144	696.42	-0.9	0.27	-3.29	1.00E-03	9.39E-02	eukaryotic translation initiation factor 1A
Ma09_g03400	ITC1587_Bchr9_P25371	138.16	-0.92	0.2	-4.66	3.10E-06	7.31E-03	uncharacterized LOC103996853
Ma01_g18760	ITC1587_Bchr1_P02111	81.05	-0.92	0.25	-3.65	2.59E-04	5.22E-02	basic leucine zipper 9-like
Ma05_g31140	ITC1587_Bchr5_P14700*	121.08	-0.94	0.27	-3.42	6.31E-04	7.98E-02	ras-related protein Rab11C-like
Ma04_g01420	ITC1587_Bchr9_P26114*	137.85	-0.96	0.21	-4.61	3.99E-06	7.83E-03	thioredoxin-like protein Clot
Ma10_g25520	ITC1587_Bchr10_P31135	103.93	-0.98	0.28	-3.56	3.72E-04	6.28E-02	mitogen-activated protein kinase kinase kinase 1-like
Ma04_g01960	ITC1587_Bchr4_P08569	63.09	-0.99	0.3	-3.36	7.71E-04	8.64E-02	BTB/POZ domain-containing protein NPY1-like
Ma04_g01230	ITC1587_Bchr4_P08492	185.31	-1	0.24	-4.24	2.25E-05	1.55E-02	lysM domain receptor-like kinase 4
Ma10_g21770	ITC1587_Bchr10_P30812	200.28	-1.02	0.29	-3.47	5.27E-04	7.28E-02	F-box/kelch-repeat protein At1g80440-like
Ma10_g13670	ITC1587_Bchr6_P16151*	365.14	-1.03	0.23	-4.49	7.11E-06	1.05E-02	soluble inorganic pyrophosphatase-like

Ma04_g15540	ITC1587_Bchr4_P09814*	123.48	-1.04	0.3	-3.47	5.29E-04	7.28E-02	probable WRKY transcription factor 65
Ma04_g31680	ITC1587_Bchr4_P10969	80.33	-1.05	0.31	-3.35	8.04E-04	8.78E-02	cyclin-dependent kinases regulatory subunit 1-like
Ma04_g12300	ITC1587_Bchr4_P09502	96.37	-1.06	0.27	-3.9	9.82E-05	3.17E-02	uncharacterized LOC103981265
Ma02_g03360	ITC1587_Bchr2_P03220	153.91	-1.07	0.26	-4.13	3.59E-05	1.82E-02	protein PHLOEM PROTEIN 2-LIKE A9-like
Ma06_g10270	ITC1587_Bchr6_P15554	123.04	-1.08	0.29	-3.69	2.27E-04	4.87E-02	adenine/guanine permease AZG1-like
Ma10_g15540	ITC1587_Bchr10_P30285	140.45	-1.11	0.34	-3.25	1.14E-03	9.83E-02	uncharacterized LOC103968343
Ma04_g37600	ITC1587_Bchr4_P11463	60.03	-1.15	0.35	-3.28	1.03E-03	9.39E-02	RING-H2 finger protein ATL39-like
Ma05_g21510	ITC1587_Bchr5_P13777	65.41	-1.17	0.35	-3.33	8.67E-04	8.97E-02	protein NLP1-like
Ma07_g24770	ITC1587_Bchr7_P21092	119.99	-1.21	0.29	-4.23	2.37E-05	1.55E-02	pentatricopeptide repeat-containing protein At1g52640
Ma04_g29670	ITC1587_Bchr4_P10780	94.92	-1.24	0.34	-3.67	2.39E-04	5.00E-02	protein DA1-related 1-like
Ma04_g03560	ITC1587_Bchr4_P08705	96.94	-1.26	0.35	-3.64	2.68E-04	5.30E-02	scarecrow-like protein 15
Ma09_g01320	ITC1587_Bchr9_P25650*	37.34	-1.27	0.35	-3.61	3.12E-04	5.83E-02	uncharacterized LOC103996656
Ma03_g11650	ITC1587_Bchr3_P06232	417.76	-1.29	0.37	-3.5	4.59E-04	7.07E-02	protein ASPARTIC PROTEASE IN GUARD CELL 2-like
Ma01_g18420	ITC1587_Bchr1_P02154	119.23	-1.3	0.31	-4.22	2.44E-05	1.55E-02	haloacid dehalogenase-like hydrolase domain-containing protein Sgpp
Ma07_g05990	ITC1587_Bchr7_P19080	102.47	-1.31	0.36	-3.67	2.39E-04	5.00E-02	vacuolar protein 8-like
Ma02_g15270	ITC1587_Bchr2_P04283	798.28	-1.31	0.39	-3.38	7.18E-04	8.51E-02	zinc finger protein 1-like
Ma03_g26680	ITC1587_Bchr3_P07793	110.64	-1.31	0.31	-4.26	2.01E-05	1.55E-02	uncharacterized LOC103978824
Ma07_g09220	ITC1587_Bchr7_P19429	2527.74	-1.32	0.33	-3.98	6.99E-05	2.84E-02	Peroxidase 52
Ma04_g02440	ITC1587_Bchr4_P08610	857.51	-1.33	0.36	-3.67	2.40E-04	5.00E-02	NAC domain-containing protein 29
Ma09_g11880	ITC1587_Bchr9_P26091	459.93	-1.34	0.39	-3.48	4.97E-04	7.16E-02	uncharacterized LOC103997701
Ma09_g15010	ITC1587_Bchr9_P26376	56.4	-1.36	0.4	-3.42	6.31E-04	7.98E-02	U-box domain-containing protein 10
Ma09_g11240	ITC1587_Bchr9_P26034*	644.83	-1.36	0.33	-4.17	3.06E-05	1.64E-02	15.4 kDa class V heat shock protein
Ma06_g14760	ITC1587_Bchr6_P15955	78.86	-1.37	0.37	-3.65	2.59E-04	5.22E-02	uncharacterized LOC103987707
Ma07_g06960	ITC1587_Bchr7_P19171	981.15	-1.39	0.42	-3.3	9.80E-04	9.31E-02	putative ethylene-responsive transcription factor ERF105
Ma00_g02790	ITC1587_BchrUn_random_P37313	706.01	-1.39	0.37	-3.78	1.57E-04	3.99E-02	putative ethylene-responsive transcription factor 9
Ma02_g13630	ITC1587_Bchr2_P04139	67.68	-1.41	0.42	-3.34	8.31E-04	8.91E-02	uncharacterized LOC103975776

Ma10_g04830	ITC1587_Bchr10_P29348	299.16	-1.41	0.43	-3.26	1.11E-03	9.70E-02	probable calcium-binding protein CML27
Ma09_g27170	ITC1587_Bchr9_P27958	668.11	-1.41	0.41	-3.42	6.17E-04	7.95E-02	zinc finger protein AZF3-like
Ma10_g25640	ITC1587_Bchr10_P31145	429.99	-1.41	0.43	-3.29	1.01E-03	9.39E-02	uncharacterized LOC103969226
Ma07_g22610	ITC1587_Bchr7_P20897	1050.27	-1.42	0.4	-3.58	3.49E-04	6.18E-02	probable calcium-binding protein CML30
Ma10_g03380	ITC1587_Bchr10_P29032	82.4	-1.46	0.43	-3.36	7.74E-04	8.64E-02	dof zinc finger protein DOF2.1-like
Ma04_g01000	ITC1587_Bchr4_P08473	103.43	-1.46	0.39	-3.74	1.83E-04	4.33E-02	6-phosphofructokinase 3
Ma06_g16200	ITC1587_Bchr6_P16096	83.84	-1.47	0.43	-3.39	6.88E-04	8.32E-02	forkhead box protein G1-like
Ma03_g14360	ITC1587_BchrUn_random_ P35631	475.04	-1.5	0.44	-3.44	5.87E-04	7.74E-02	thebaine 6-O-demethylase-like
Ma05_g08890	ITC1587_Bchr5_P12481	152.41	-1.5	0.45	-3.31	9.20E-04	9.07E-02	uncharacterized LOC103984321
Ma11_g20950	ITC1587_Bchr11_P34050	164.43	-1.51	0.44	-3.4	6.63E-04	8.22E-02	uncharacterized LOC103972143
Ma01_g10090	ITC1587_Bchr1_P01463	87.31	-1.53	0.44	-3.52	4.25E-04	6.72E-02	uncharacterized LOC103983795
Ma06_g14710	ITC1587_Bchr6_P15949	138.16	-1.55	0.45	-3.46	5.34E-04	7.28E-02	Beta-fructofuranosidase
Ma09_g30960		64.79	-1.58	0.45	-3.49	4.90E-04	7.16E-02	Hypothetical protein
Ma10_g29370	ITC1587_Bchr10_P31483*	54.75	-1.59	0.48	-3.33	8.68E-04	8.97E-02	copper transport protein CCH
Ma03_g01740	ITC1587_Bchr3_P05326	60.39	-1.6	0.48	-3.35	8.09E-04	8.78E-02	E3 ubiquitin-protein ligase RING1-like
Ma03_g22010	ITC1587_Bchr3_P07395	540.87	-1.61	0.48	-3.33	8.68E-04	8.97E-02	AP2/ERF and B3 domain-containing transcription repressor RAV2-like
Ma06_g20750	ITC1587_Bchr6_P16539	380.79	-1.63	0.41	-3.95	7.82E-05	3.03E-02	probable CCR4-associated factor 1 homolog 11
Ma04_g21170	ITC1587_Bchr1_P00050*	661.7	-1.65	0.39	-4.21	2.58E-05	1.56E-02	putative ethylene-responsive transcription factor ERF105
Ma09_g11100	ITC1587_Bchr9_P26023	437.72	-1.65	0.37	-4.44	9.16E-06	1.20E-02	putative ethylene-responsive transcription factor 4
Ma07_g23800	ITC1587_Bchr7_P21000*	236.89	-1.65	0.37	-4.45	8.62E-06	1.19E-02	PRA1 family protein B4-like
Ma10_g24780	ITC1587_Bchr10_P31078	422.11	-1.66	0.46	-3.61	3.09E-04	5.82E-02	E3 ubiquitin-protein ligase ATL6-like
Ma06_g28960	ITC1587_Bchr6_P17646	140.79	-1.69	0.41	-4.11	4.04E-05	1.94E-02	uncharacterized LOC103989217
Ma07_g28030	ITC1587_Bchr7_P21366	222.01	-1.73	0.45	-3.84	1.23E-04	3.62E-02	uncharacterized LOC103992910
Ma09_g01900	ITC1587_Bchr9_P25241	20.9	-1.74	0.46	-3.81	1.42E-04	3.75E-02	high-affinity nitrate transporter-activating protein 2.1
Ma08_g21570	ITC1587_Bchr8_P23856	1113.22	-1.75	0.41	-4.26	2.08E-05	1.55E-02	E3 ubiquitin-protein ligase RING1-like
Ma10_g08130	ITC1587_Bchr10_P29571	292.17	-1.82	0.51	-3.59	3.28E-04	5.98E-02	NBS-LRR disease resistance protein

Ma09_g18570	ITC1587_Bchr9_P27174	173.17	-1.82	0.48	-3.79	1.50E-04	3.90E-02	zinc finger A20 and AN1 domain-containing stress-associated protein 1-like
Ma02_g23000	ITC1587_Bchr2_P04978	687.87	-1.84	0.43	-4.23	2.37E-05	1.55E-02	NAC transcription factor NAM-B1-like
Ma04_g07750	ITC1587_Bchr4_P09063*	305.74	-1.85	0.38	-4.85	1.22E-06	5.76E-03	E3 ubiquitin-protein ligase RING1-like
Ma01_g14820		66.74	-1.85	0.49	-3.79	1.54E-04	3.94E-02	lysine-rich arabinogalactan protein 19-like
Ma05_g16120	ITC1587_BchrUn_random_P35166	765.44	-1.86	0.55	-3.38	7.21E-04	8.51E-02	E3 ubiquitin-protein ligase RHA1B-like
Ma07_g28560	ITC1587_Bchr7_P21459	61.46	-1.87	0.45	-4.2	2.66E-05	1.57E-02	homeobox-leucine zipper protein HAT22-like
Ma10_g27530	ITC1587_Bchr10_P31316*	178.46	-1.87	0.57	-3.29	1.02E-03	9.39E-02	probable inositol oxygenase
Ma09_g30300	ITC1587_Bchr9_P28242	1679.4	-1.88	0.4	-4.68	2.82E-06	7.31E-03	probable CCR4-associated factor 1 homolog 11
Ma11_g23420	ITC1587_Bchr11_P34250	29.4	-1.9	0.5	-3.76	1.67E-04	4.12E-02	myb-like protein Q
Ma05_g29260	ITC1587_Bchr5_P14533	378.95	-1.9	0.52	-3.66	2.55E-04	5.22E-02	uncharacterized LOC103986497
Ma05_g22280	ITC1587_Bchr5_P13859	61.42	-1.93	0.49	-3.92	8.92E-05	3.17E-02	uncharacterized LOC103985720
Ma04_g02760	ITC1587_Bchr4_P08643	114.06	-1.96	0.57	-3.43	5.94E-04	7.74E-02	uncharacterized LOC103980226
Ma06_g05470	ITC1587_Bchr6_P15135	29.48	-1.98	0.58	-3.4	6.79E-04	8.25E-02	uncharacterized LOC103986901
Ma10_g18710	ITC1587_Bchr10_P30551	276.15	-2	0.53	-3.81	1.42E-04	3.75E-02	probable calcium-binding protein CML45
Ma02_g19880	ITC1587_Bchr2_P04697	56.78	-2.01	0.53	-3.82	1.35E-04	3.71E-02	non-specific phospholipase C4-like
Ma01_g03350	ITC1587_Bchr1_P00730*	1265.81	-2.02	0.57	-3.55	3.85E-04	6.35E-02	ABC transporter G family member 39-like
Ma01_g14250	ITC1587_Bchr1_P01834	132.88	-2.04	0.57	-3.59	3.36E-04	6.05E-02	uncharacterized LOC103992216
Ma06_g37470	ITC1587_Bchr9_P25803*	181.4	-2.05	0.52	-3.92	8.78E-05	3.17E-02	cationic amino acid transporter 7
Ma08_g17710	ITC1587_Bchr8_P23528	119.73	-2.07	0.49	-4.25	2.14E-05	1.55E-02	AP2/ERF and B3 domain-containing transcription repressor RAV2-like
Ma05_g08420	ITC1587_Bchr5_P12435	653.57	-2.11	0.51	-4.13	3.63E-05	1.82E-02	zinc finger protein ZAT6-like
Ma04_g10050	ITC1587_Bchr4_P09278	65.22	-2.13	0.54	-3.91	9.22E-05	3.17E-02	metacaspase-9
Ma06_g12070	ITC1587_Bchr6_P15720	39.09	-2.2	0.66	-3.32	9.09E-04	9.00E-02	uncharacterized LOC103987475
Ma04_g12590	ITC1587_Bchr4_P09526	120.96	-2.22	0.66	-3.38	7.26E-04	8.51E-02	CASP-like protein 2C1
Ma05_g28810	ITC1587_Bchr5_P14487	32.8	-2.24	0.51	-4.36	1.30E-05	1.46E-02	transcription factor bHLH123-like
Ma08_g29810	ITC1587_Bchr8_P24604	42.04	-2.25	0.59	-3.82	1.35E-04	3.71E-02	serine/threonine-protein kinase-like protein CCR4

Ma01_g21550	ITC1587_BchrUn_random_P37779	987.54	-2.27	0.67	-3.38	7.34E-04	8.52E-02	3-hydroxy-3-methylglutaryl-coenzyme A reductase 3-like
Ma02_g01530	ITC1587_Bchr8_P23662*	46.69	-2.28	0.48	-4.72	2.37E-06	7.31E-03	no apical meristem protein
Ma04_g18750	ITC1587_BchrUn_random_P39561	26.94	-2.3	0.66	-3.46	5.43E-04	7.35E-02	phosphoenolpyruvate carboxylase kinase 2-like
Ma05_g01620	ITC1587_Bchr5_P11796	83.49	-2.32	0.68	-3.39	6.93E-04	8.33E-02	zinc finger protein 1-like
Ma01_g21640	ITC1587_Bchr1_P02753	190.83	-2.35	0.65	-3.6	3.15E-04	5.84E-02	probable calcium-binding protein CML31
Ma07_g18480	ITC1587_BchrUn_random_P37067	271.06	-2.41	0.43	-5.56	2.64E-08	2.08E-04	uncharacterized LOC103974030
Ma04_g02740	ITC1587_Bchr4_P08641	14.43	-2.44	0.64	-3.83	1.30E-04	3.71E-02	uncharacterized LOC103980228
Ma04_g21160	ITC1587_Bchr1_P00051	159.4	-2.45	0.66	-3.72	2.01E-04	4.60E-02	hypothetical protein
Ma07_g06000	ITC1587_Bchr7_P19081	452.98	-2.47	0.55	-4.52	6.21E-06	1.05E-02	putative dehydration-responsive element-binding protein 1D
Ma04_g03780	ITC1587_Bchr4_P08726	168.99	-2.67	0.63	-4.24	2.21E-05	1.55E-02	AP2/ERF and B3 domain-containing transcription factor RAV1-like
Ma06_g11140	ITC1587_Bchr6_P15633	39.96	-2.97	0.75	-3.98	6.96E-05	2.84E-02	Myb-related protein 308
Ma05_g04060	ITC1587_Bchr5_P12038	35.83	-3.2	0.95	-3.37	7.55E-04	8.60E-02	U-box domain-containing protein 35
Ma09_g17280	ITC1587_BchrUn_random_P36874	15.24	-3.25	0.76	-4.27	1.94E-05	1.55E-02	protein NRT1/ PTR FAMILY 5.1-like
Ma04_g00440	ITC1587_Bchr4_P08419	18.13	-3.25	0.83	-3.9	9.74E-05	3.17E-02	extracellular ribonuclease LE-like
Ma02_g10360	ITC1587_Bchr2_P03864	48.75	-3.44	1	-3.45	5.61E-04	7.55E-02	uncharacterized LOC103976380
Ma05_g26410	ITC1587_Bchr5_P14270	25.86	-3.84	0.94	-4.08	4.56E-05	2.12E-02	pectinesterase/pectinesterase inhibitor PPE8B-like
Ma10_g27290	ITC1587_Bchr10_P31295	2094.05	-4.38	1.32	-3.31	9.38E-04	9.13E-02	cytochrome P450 86B1-like
Ma11_g09840	ITC1587_Bchr11_P32947	18.92	-4.58	1.4	-3.27	1.09E-03	9.56E-02	E3 ubiquitin-protein ligase PUB23-like
Ma06_g19500	ITC1587_Bchr6_P16394	31.47	-4.93	1.3	-3.79	1.50E-04	3.90E-02	U-box domain-containing protein 27-like

Note: Gene IDs from *Musa balbisiana* are based on the results of the reciprocal best BLAST search. The best BLAST hit is reported for the genes where the reciprocal best BLAST search hit was not available and it is denoted with *.

Table S5: Complete list of genes differentially expressed due to genotypes interaction with *Xanthomonas campestris* pv. *musacearum* at 12 hpi.

Gene ID from *Musa acuminata* (DH Pahang)	Gene ID from *Musa balbisiana* (Pisang Klutuk Wulung)	BaseMean	Log2 Fold Change	lfcSE	Stat	Pvalue	Padj	Description
Ma08_g16700	ITC1587_Bchr6_P15616*	18.1	8.1	2.22	3.65	2.58E-04	2.55E-02	protein MOTHER of FT and TF 1-like
Ma06_g06020	ITC1587_Bchr6_P15185	10.32	6.45	1.76	3.68	2.38E-04	2.46E-02	mavicyanin-like
Ma06_g14430	ITC1587_BchrUn_random_P38187	114.05	6.42	2.04	3.14	1.69E-03	6.57E-02	1-aminocyclopropane-1-carboxylate oxidase-like
Ma06_g06860	ITC1587_BchrUn_random_P38862	6.00	6.23	1.81	3.44	5.83E-04	3.89E-02	geranylgeranyl pyrophosphate synthase 7
Ma08_g29360	ITC1587_Bchr8_P24561	38.48	6.13	1.64	3.73	1.91E-04	2.16E-02	uncharacterized LOC103995997
Ma06_g13260	ITC1587_Bchr6_P15827	76.29	5.81	1.58	3.67	2.40E-04	2.47E-02	proline-rich receptor-like protein kinase PERK10
Ma04_g02930	ITC1587_Bchr4_P08659	53.57	5.74	1.41	4.08	4.52E-05	9.23E-03	glucose-1-phosphate adenylyltransferase large subunit 1-like
Ma09_g29150	ITC1587_Bchr9_P28137	16.52	4.88	1.49	3.28	1.05E-03	5.12E-02	putative 26.5 kDa heat shock protein
Ma05_g11490	ITC1587_Bchr5_P12707	10.56	4.82	1.24	3.89	9.84E-05	1.43E-02	RING-H2 finger protein ATL8-like
Ma08_g08200	ITC1587_Bchr8_P22177	95.73	4.76	1.14	4.19	2.79E-05	6.92E-03	putative expressed protein
Ma04_g39350	ITC1587_Bchr4_P11595	19.99	4.46	1.36	3.27	1.07E-03	5.17E-02	ninja-family protein AFP3-like
Ma10_g29680	ITC1587_Bchr10_P31511*	110.13	4.42	1.23	3.58	3.39E-04	2.96E-02	ATP-dependent zinc metalloprotease FTSH 6
Ma09_g28620	ITC1587_Bchr9_P28084	48.14	4.39	1.08	4.07	4.74E-05	9.47E-03	RING-H2 finger protein ATL43-like
Ma03_g04050	ITC1587_Bchr3_P05519	97.08	4.26	1.11	3.83	1.28E-04	1.68E-02	BTB/POZ domain-containing protein At1g55760-like
Ma02_g05420	ITC1587_Bchr2_P03415	13.44	4	1.18	3.4	6.79E-04	4.25E-02	uncharacterized LOC103973692
Ma08_g17800	ITC1587_Bchr8_P23541	56.81	3.94	1.13	3.5	4.72E-04	3.57E-02	protein PLASTID MOVEMENT IMPAIRED 2-like
Ma06_g09540	ITC1587_Bchr6_P15490*	14.52	3.77	1.24	3.03	2.41E-03	7.84E-02	protein HVA22-like
Ma08_g04900	ITC1587_Bchr8_P21882*	18.72	3.76	0.86	4.37	1.25E-05	4.44E-03	fragment
Ma03_g31600	ITC1587_Bchr3_P08196	45.25	3.75	1.19	3.15	1.62E-03	6.35E-02	ninja-family protein 6-like
Ma09_g02560	ITC1587_Bchr9_P25297	15.91	3.66	1.18	3.1	1.91E-03	6.94E-02	uncharacterized LOC103996779

Ma10_g12720	ITC1587_Bchr5_P14061*	119.76	3.64	1.22	2.99	2.79E-03	8.58E-02	Hypothetical protein
Ma10_g02420	ITC1587_Bchr10_P28390	50.18	3.62	1.14	3.17	1.52E-03	6.20E-02	BAHD acyltransferase DCR-like
Ma10_g11690	ITC1587_Bchr4_P09673*	8.64	3.58	1.08	3.31	9.31E-04	4.91E-02	protein SYM1-like
Ma05_g24030	ITC1587_Bchr5_P14049	97.05	3.53	0.91	3.89	1.02E-04	1.43E-02	heparan-alpha-glucosaminide N-acetyltransferase-like
Ma08_g15990	ITC1587_Bchr8_P23247	20.91	3.53	0.83	4.24	2.24E-05	6.09E-03	annexin-like protein RJ4
Ma11_g02070	ITC1587_Bchr11_P31881	159.46	3.44	0.72	4.75	2.06E-06	1.46E-03	probable protein phosphatase 2C 75
Ma02_g08740	ITC1587_Bchr2_P03721	26.62	3.39	0.82	4.12	3.82E-05	8.38E-03	phosphatidylinositol 4-phosphate 5-kinase 6-like
Ma09_g09290		70.81	3.34	0.89	3.74	1.87E-04	2.16E-02	putative expressed protein
Ma08_g19300	ITC1587_Bchr8_P22999	144.8	2.98	0.76	3.94	8.19E-05	1.30E-02	putative expressed protein
Ma09_g16350	ITC1587_Bchr9_P26575	56.74	2.92	0.78	3.76	1.70E-04	2.05E-02	cytokinin dehydrogenase 4-like
Ma04_g35970	ITC1587_Bchr4_P11328	71.11	2.91	1	2.93	3.43E-03	9.41E-02	subtilisin-like protease
Ma04_g26590	ITC1587_Bchr4_P10494	413.65	2.9	0.78	3.73	1.94E-04	2.18E-02	uncharacterized LOC103983225
Ma01_g19260		12.86	2.89	0.91	3.19	1.44E-03	6.08E-02	Hypothetical protein
Ma04_g11920	ITC1587_Bchr4_P09465	18.78	2.75	0.74	3.73	1.89E-04	2.16E-02	suppressor of phythchrome A
Ma09_g06650	ITC1587_Bchr9_P25627	60.24	2.73	0.67	4.06	4.82E-05	9.55E-03	glucose-1-phosphate adenylyltransferase large subunit 1-like
Ma06_g28600	ITC1587_Bchr9_P25602*	58.99	2.68	0.68	3.92	8.89E-05	1.35E-02	gibberellin 2-beta-dioxygenase 1-like
Ma03_g06900	ITC1587_Bchr3_P05783	53.7	2.68	0.91	2.94	3.33E-03	9.31E-02	abscisic acid 8'-hydroxylase 3-like
Ma04_g07150	ITC1587_Bchr4_P09012	15.08	2.62	0.89	2.94	3.33E-03	9.31E-02	homeobox-leucine zipper protein HOX12-like
Ma01_g13530	ITC1587_Bchr1_P01779	44.08	2.61	0.8	3.28	1.04E-03	5.11E-02	13-hydroxylupanine O-tigloyltransferase-like
Ma06_g27540	ITC1587_Bchr6_P17520	39.67	2.61	0.76	3.42	6.30E-04	4.07E-02	exocyst complex component EXO70B1-like
Ma02_g07220	ITC1587_Bchr2_P03579	555.54	2.6	0.77	3.39	7.01E-04	4.30E-02	cinnamate beta-D-glucosyltransferase-like
Ma06_g01860	ITC1587_Bchr6_P14797*	353.61	2.59	0.69	3.78	1.55E-04	1.91E-02	uncharacterized LOC103986549
Ma06_g12080	ITC1587_Bchr6_P15721*	23.38	2.55	0.8	3.2	1.39E-03	5.99E-02	uncharacterized LOC103987476
Ma07_g09320	ITC1587_Bchr7_P19437	60.85	2.52	0.81	3.11	1.86E-03	6.90E-02	uncharacterized LOC103991249
Ma03_g12470	ITC1587_Bchr3_P06303	387.5	2.48	0.48	5.22	1.77E-07	3.13E-04	uncharacterized LOC103978016
Ma06_g05410	ITC1587_Bchr1_P02099*	20.47	2.48	0.68	3.64	2.70E-04	2.63E-02	villin-4-like

Ma10_g23080	ITC1587_Bchr10_P30931	2544.37	2.43	0.61	3.98	7.00E-05	1.19E-02	alpha-aminoadipic semialdehyde synthase
Ma04_g28140	ITC1587_Bchr4_P10637	98.71	2.43	0.8	3.05	2.32E-03	7.65E-02	expansin-A7-like
Ma10_g13630	ITC1587_Bchr10_P30106	21.97	2.39	0.71	3.36	7.76E-04	4.45E-02	Aquaporin TIP1-3
Ma05_g11350	ITC1587_Bchr11_P34036*	35.24	2.37	0.76	3.12	1.81E-03	6.81E-02	expansin-like A1
Ma04_g08840	ITC1587_Bchr4_P09163	44.29	2.37	0.68	3.48	5.07E-04	3.63E-02	protein CHUP1
Ma06_g12870	ITC1587_Bchr6_P15794*	223.72	2.36	0.74	3.19	1.40E-03	5.99E-02	monoglyceride lipase-like
Ma02_g08780	ITC1587_Bchr2_P03725	398.26	2.33	0.7	3.33	8.63E-04	4.70E-02	alpha carbonic anhydrase 1
Ma07_g01870	ITC1587_Bchr7_P18719	839.75	2.33	0.56	4.14	3.50E-05	7.90E-03	probable galactinol--sucrose galactosyltransferase 2
Ma11_g09710	ITC1587_Bchr11_P32964	1155	2.32	0.59	3.91	9.12E-05	1.37E-02	ferric reduction oxidase 7
Ma05_g26690	ITC1587_Bchr6_P15148*	100.12	2.27	0.58	3.89	1.01E-04	1.43E-02	uncharacterized LOC103985344
Ma02_g22270	ITC1587_Bchr2_P04917	215.73	2.26	0.47	4.84	1.29E-06	1.07E-03	protein FANTASTIC FOUR 1-like
Ma04_g12630	ITC1587_Bchr4_P09530	201.68	2.24	0.66	3.38	7.35E-04	4.37E-02	probable homogentisate phytyltransferase 1
Ma01_g14540	ITC1587_Bchr1_P01860*	3020.52	2.21	0.6	3.66	2.49E-04	2.53E-02	chr1
Ma02_g24650	ITC1587_Bchr2_P05115	74.28	2.2	0.72	3.05	2.30E-03	7.62E-02	uncharacterized LOC103976654
Ma10_g30810	ITC1587_Bchr10_P31614	32.71	2.2	0.75	2.93	3.39E-03	9.35E-02	zinc finger protein 4-like
Ma04_g39080	ITC1587_Bchr2_P04285*	282.3	2.18	0.53	4.1	4.10E-05	8.58E-03	LOB domain-containing protein 37-like
Ma04_g26870	ITC1587_Bchr4_P10518	112.22	2.18	0.42	5.14	2.73E-07	3.67E-04	uncharacterized LOC103983203
Ma09_g19630	ITC1587_Bchr9_P26735	44.77	2.18	0.48	4.56	5.13E-06	2.51E-03	scarecrow-like protein 8
Ma05_g03580	ITC1587_Bchr5_P11986	438.57	2.17	0.56	3.85	1.20E-04	1.62E-02	ubiquinol oxidase 2
Ma04_g33310	ITC1587_Bchr4_P11096	52.16	2.17	0.63	3.42	6.18E-04	4.03E-02	phenolic glucoside malonyltransferase 1-like
Ma10_g27770	ITC1587_Bchr10_P31333	539.67	2.16	0.7	3.09	2.01E-03	7.15E-02	heat stress transcription factor C-2b-like
Ma04_g25840	ITC1587_Bchr1_P00570	1647.33	2.14	0.59	3.61	3.07E-04	2.84E-02	granule-bound starch synthase 1
Ma02_g16590	ITC1587_Bchr2_P04403	495.24	2.14	0.53	4.05	5.18E-05	9.80E-03	uncharacterized glycosyl hydrolase Rv2006/MT2062
Ma07_g17570	ITC1587_BchrUn_random_P37718	995.18	2.12	0.47	4.55	5.31E-06	2.51E-03	CBL-interacting protein kinase 1-like
Ma03_g18730	ITC1587_BchrUn_random_P38210	187.78	2.12	0.59	3.61	3.04E-04	2.84E-02	probable protein phosphatase 2C 68
Ma01_g02150	ITC1587_Bchr1_P00623*	855.24	2.1	0.47	4.45	8.63E-06	3.61E-03	probable ascorbate-specific transmembrane electron transporter 1

Ma05_g11180	ITC1587_Bchr5_P12673	48.72	2.08	0.62	3.34	8.33E-04	4.62E-02	uncharacterized LOC103984779
Ma05_g25720	ITC1587_Bchr5_P14209	250	2.08	0.69	2.99	2.75E-03	8.53E-02	Aquaporin NIP2-1
Ma10_g04770	ITC1587_Bchr10_P29341	624.12	2.04	0.61	3.34	8.48E-04	4.66E-02	fatty acid desaturase 4
Ma06_g29770	ITC1587_Bchr6_P17724	362.52	2.04	0.39	5.21	1.84E-07	3.13E-04	serine/threonine-protein kinase BLUS1
Ma04_g35590	ITC1587_Bchr4_P11292	101.44	2.02	0.59	3.45	5.55E-04	3.77E-02	heat stress transcription factor B-2b-like
Ma03_g05910	ITC1587_Bchr3_P05693	2027.55	2.02	0.63	3.22	1.28E-03	5.71E-02	protein NRT1/ PTR FAMILY 6.4
Ma01_g01360	ITC1587_Bchr11_P33828*	671.39	2.02	0.46	4.39	1.14E-05	4.17E-03	uncharacterized LOC103982299
Ma06_g15590	ITC1587_Bchr6_P16038	56.58	2.02	0.64	3.18	1.48E-03	6.13E-02	mitochondrial arginine transporter BAC2
Ma04_g11810	ITC1587_Bchr4_P09454	53.88	2	0.49	4.06	4.97E-05	9.68E-03	uncharacterized LOC103981226
Ma05_g16190	ITC1587_BchrUn_random_ P36900*	1083.33	2	0.47	4.21	2.55E-05	6.58E-03	inositol-3-phosphate synthase-like
Ma03_g00900	ITC1587_Bchr3_P05244	257.65	1.97	0.62	3.17	1.54E-03	6.24E-02	protein ECERIFERUM 3-like
Ma11_g14170	ITC1587_Bchr11_P33405	94.84	1.97	0.4	4.92	8.45E-07	8.30E-04	truncated transcription factor CAULIFLOWER A-like
Ma01_g12450	ITC1587_Bchr1_P01690	80.87	1.97	0.64	3.11	1.90E-03	6.94E-02	phosphatidylinositol/phosphatidylcholine transfer protein SFH2-like
Ma01_g01110	ITC1587_BchrUn_random_ P35233*	369.57	1.96	0.6	3.29	1.01E-03	5.09E-02	uncharacterized LOC103982057
Ma09_g14370	ITC1587_Bchr9_P26312	158.71	1.95	0.3	6.51	7.51E-11	4.80E-07	homeobox-leucine zipper protein HOX4-like
Ma09_g11210	ITC1587_Bchr9_P26031	451.71	1.95	0.5	3.92	8.91E-05	1.35E-02	probable nucleoredoxin 2
Ma05_g15740	ITC1587_Bchr5_P13143	15.81	1.93	0.62	3.09	2.02E-03	7.16E-02	RING-H2 finger protein ATL45
Ma08_g24440	ITC1587_Bchr8_P24119	153.72	1.91	0.59	3.23	1.24E-03	5.61E-02	18.6 kDa class III heat shock protein
Ma02_g08840	ITC1587_Bchr2_P03732	196.45	1.9	0.6	3.16	1.57E-03	6.29E-02	annexin D3-like
Ma04_g26780	ITC1587_Bchr4_P10509	54.07	1.87	0.56	3.33	8.76E-04	4.75E-02	uncharacterized LOC103983209
Ma06_g08930	ITC1587_Bchr6_P15433	771.36	1.87	0.48	3.91	9.22E-05	1.37E-02	potassium channel AKT2-like
Ma06_g21380	ITC1587_Bchr6_P16609	331.12	1.86	0.58	3.21	1.33E-03	5.84E-02	uncharacterized protein PHLOEM PROTEIN 2-LIKE A4-like
Ma03_g11520	ITC1587_Bchr3_P06219	10765.1	1.84	0.26	7.22	5.32E-13	1.36E-08	linoleate 13S-lipoxygenase 2-1
Ma10_g07810	ITC1587_Bchr10_P29542	126.62	1.83	0.47	3.85	1.19E-04	1.61E-02	zinc finger protein ZAT5-like
Ma05_g07450	ITC1587_Bchr5_P12351	41.94	1.81	0.52	3.47	5.15E-04	3.66E-02	putative Myb-related protein 306
Ma04_g10170	ITC1587_Bchr4_P09289	360.22	1.81	0.56	3.24	1.21E-03	5.50E-02	Granule-bound starch synthase 2

Ma04_g12960	ITC1587_Bchr4_P09559	416.74	1.8	0.52	3.5	4.67E-04	3.56E-02	cinnamyl alcohol dehydrogenase 2-like
Ma09_g06800	ITC1587_Bchr6_P17663*	5725.74	1.8	0.47	3.8	1.45E-04	1.82E-02	carbonic anhydrase 2
Ma09_g21420	ITC1587_Bchr9_P27340	25.99	1.8	0.54	3.33	8.56E-04	4.68E-02	auxin-induced protein 15A-like
Ma08_g04580	ITC1587_Bchr8_P21853	856.74	1.79	0.5	3.6	3.12E-04	2.84E-02	ABC transporter G family member 22-like
Ma10_g02900	ITC1587_Bchr10_P29012	128.18	1.79	0.43	4.18	2.93E-05	7.06E-03	uncharacterized LOC103999850
Ma00_g03250	ITC1587_BchrUn_random_P37589	34.83	1.79	0.54	3.32	9.07E-04	4.85E-02	serine/threonine-protein kinase HT1
Ma05_g14690	ITC1587_Bchr7_P18716*	33.04	1.78	0.5	3.54	3.98E-04	3.30E-02	expressed protein
Ma04_g04500	ITC1587_Bchr4_P08787	85.64	1.75	0.38	4.6	4.17E-06	2.22E-03	mitogen-activated protein kinase homolog MMK2-like
Ma10_g17650	ITC1587_Bchr10_P30471	192.69	1.75	0.4	4.35	1.36E-05	4.61E-03	Hypothetical protein
Ma06_g13850	ITC1587_Bchr6_P15877	442.55	1.74	0.55	3.19	1.43E-03	6.04E-02	Stromal 70 kDa heat shock-related protein
Ma05_g06010	ITC1587_Bchr5_P12219	1634.52	1.74	0.51	3.43	6.11E-04	4.02E-02	dehydrodolichyl diphosphate synthase 2-like
Ma03_g33240	ITC1587_Bchr3_P08330	614.05	1.73	0.55	3.14	1.71E-03	6.61E-02	probable protein phosphatase 2C 51
Ma10_g15660	ITC1587_Bchr10_P30296	94.19	1.72	0.46	3.71	2.08E-04	2.26E-02	uncharacterized LOC103968354
Ma05_g09070	ITC1587_Bchr5_P12498*	31.36	1.7	0.54	3.13	1.75E-03	6.66E-02	Rd22-c
Ma05_g15800	ITC1587_Bchr5_P13135	11754.15	1.7	0.39	4.38	1.16E-05	4.19E-03	catalase isozyme 2
Ma06_g20720	ITC1587_Bchr6_P16536	149.92	1.69	0.5	3.38	7.27E-04	4.35E-02	IAA-amino acid hydrolase ILR1-like 3
Ma04_g19490	ITC1587_Bchr4_P10415	902.08	1.68	0.44	3.84	1.24E-04	1.66E-02	putative Probable carotenoid cleavage dioxygenase 4
Ma04_g20640	ITC1587_Bchr1_P00100*	130.63	1.68	0.53	3.17	1.54E-03	6.24E-02	probable folate-biopterin transporter 8
Ma06_g29120	ITC1587_Bchr6_P17663	10291.28	1.68	0.51	3.3	9.57E-04	4.97E-02	carbonic anhydrase 2-like
Ma07_g22220	ITC1587_Bchr7_P20861	75.37	1.68	0.4	4.14	3.45E-05	7.86E-03	protein MIZU-KUSSEI 1-like
Ma05_g05440	ITC1587_Bchr5_P12164	328.02	1.67	0.43	3.91	9.29E-05	1.37E-02	putative ABSCISIC ACID-INSENSITIVE 5-like protein 5
Ma04_g29770	ITC1587_Bchr4_P10790	391.29	1.63	0.47	3.48	4.98E-04	3.63E-02	probable N-acetyltransferase HLS1
Ma01_g16260	ITC1587_Bchr1_P02022	102.55	1.61	0.44	3.66	2.56E-04	2.54E-02	putative MO25-like protein At5g47540
Ma01_g00940	ITC1587_Bchr1_P00935	102.12	1.6	0.4	4.04	5.27E-05	9.80E-03	CBL-interacting serine/threonine-protein kinase 21
Ma11_g19950	ITC1587_Bchr11_P33957	189.85	1.6	0.41	3.94	8.07E-05	1.30E-02	C4-dicarboxylate transporter/malic acid transport protein domain containing

								protein
Ma01_g00370	ITC1587_Bchr1_P00881	48.38	1.6	0.52	3.09	2.01E-03	7.15E-02	serine/threonine-protein kinase HT1-like
Ma04_g11690	ITC1587_Bchr4_P09441*	72.56	1.6	0.42	3.84	1.25E-04	1.66E-02	uncharacterized LOC103981214
Ma09_g06240	ITC1587_Bchr9_P25599	361.16	1.59	0.37	4.33	1.51E-05	4.96E-03	phosphoenolpyruvate carboxylase 2-like
Ma10_g28290	ITC1587_Bchr10_P31383	862.06	1.59	0.34	4.74	2.19E-06	1.51E-03	crocetin glucosyltransferase
Ma02_g24140	ITC1587_Bchr2_P05074	1265.06	1.58	0.46	3.46	5.38E-04	3.69E-02	uncharacterized LOC103976698
Ma02_g10490	ITC1587_BchrUn_random_ P39260	1402.88	1.58	0.26	6.11	9.87E-10	5.04E-06	uncharacterized LOC103975211
Ma08_g25160	ITC1587_Bchr8_P24177	5651.96	1.58	0.47	3.34	8.36E-04	4.62E-02	serine--glyoxylate aminotransferase
Ma08_g22300	ITC1587_Bchr8_P23926*	533.76	1.56	0.34	4.63	3.64E-06	2.00E-03	monoacylglycerol lipase abhd6-A
Ma11_g21590	ITC1587_Bchr11_P34105	337.28	1.55	0.47	3.28	1.03E-03	5.11E-02	uncharacterized LOC103972193
Ma04_g37350	ITC1587_Bchr4_P11441	203.2	1.54	0.5	3.12	1.83E-03	6.87E-02	putative GATA transcription factor 22
Ma11_g08230	ITC1587_Bchr11_P32485	350.83	1.54	0.48	3.18	1.47E-03	6.11E-02	putative quinone-oxidoreductase homolog
Ma03_g26030	ITC1587_Bchr3_P07734	165.64	1.53	0.44	3.51	4.46E-04	3.52E-02	uncharacterized LOC103978888
Ma04_g30210	ITC1587_Bchr4_P10833	158.06	1.53	0.48	3.21	1.33E-03	5.84E-02	uncharacterized LOC103982883
Ma11_g07820	ITC1587_Bchr11_P32446	650.59	1.53	0.42	3.66	2.51E-04	2.53E-02	putative Zinc finger protein CONSTANS-LIKE 5
Ma05_g22300	ITC1587_Bchr5_P13862	7388.11	1.52	0.23	6.51	7.44E-11	4.80E-07	fructose-bisphosphate aldolase 1
Ma04_g21080	ITC1587_Bchr1_P00061	365.42	1.52	0.49	3.09	2.03E-03	7.18E-02	BOI-related E3 ubiquitin-protein ligase 1-like
Ma02_g20940	ITC1587_Bchr2_P04782	265.12	1.51	0.49	3.08	2.06E-03	7.22E-02	uncharacterized protein C24B11.05-like
Ma07_g20170	ITC1587_Bchr7_P20677	437.32	1.5	0.38	3.99	6.57E-05	1.16E-02	uncharacterized LOC103992199
Ma06_g32210	ITC1587_Bchr6_P17950	558.55	1.5	0.36	4.18	2.91E-05	7.06E-03	bZIP transcription factor TRAB1
Ma04_g37190	ITC1587_Bchr4_P11429	251.13	1.49	0.49	3.07	2.11E-03	7.28E-02	zinc finger protein CONSTANS-LIKE 16-like
Ma03_g11100	ITC1587_Bchr3_P06188	393.84	1.49	0.41	3.62	2.92E-04	2.80E-02	cation/H( ) antiporter 15-like
Ma07_g12340	ITC1587_Bchr7_P19705	310.08	1.48	0.47	3.18	1.47E-03	6.11E-02	protein TIFY 6B-like
Ma10_g31280	ITC1587_Bchr10_P31664	138.32	1.48	0.4	3.69	2.25E-04	2.36E-02	ubiquitin-conjugating enzyme E2-23 kDa-like
Ma01_g11590	ITC1587_Bchr1_P01610*	242.21	1.48	0.44	3.38	7.24E-04	4.35E-02	uncharacterized protein At5g50100
Ma10_g14740	ITC1587_Bchr10_P30213	1930.07	1.48	0.5	2.96	3.05E-03	8.91E-02	ABC transporter C family member 10
Ma11_g15090	ITC1587_Bchr8_P23683*	728.24	1.47	0.42	3.46	5.32E-04	3.69E-02	cytokinin riboside 5'-monophosphate

								phosphoribohydrolase LOG1-like
Ma10_g05590	ITC1587_Bchr10_P28478	2853.91	1.46	0.32	4.51	6.35E-06	2.80E-03	phosphate metabolism protein 8-like
Ma06_g03360	ITC1587_Bchr6_P14922*	236.18	1.45	0.41	3.55	3.82E-04	3.20E-02	probable sodium/metabolite cotransporter BASS2
Ma01_g15700	ITC1587_Bchr1_P01969	308.29	1.44	0.33	4.3	1.71E-05	5.13E-03	glutamine synthetase nodule isozyme-like
Ma06_g37200	ITC1587_Bchr6_P18382	257.29	1.43	0.44	3.28	1.05E-03	5.11E-02	stress enhanced protein 2
Ma08_g14430	ITC1587_Bchr8_P23704	333.01	1.43	0.34	4.2	2.69E-05	6.73E-03	uncharacterized LOC103995009
Ma06_g17610	ITC1587_Bchr6_P16213	136.68	1.42	0.3	4.72	2.38E-06	1.55E-03	E3 ubiquitin-protein ligase SGR9
Ma04_g09240	ITC1587_Bchr4_P09206	1541.79	1.41	0.43	3.28	1.04E-03	5.11E-02	ABC1 kinase
Ma06_g08050	ITC1587_Bchr6_P15353	700.7	1.41	0.48	2.96	3.07E-03	8.93E-02	uncharacterized LOC103987119
Ma02_g22230	ITC1587_Bchr2_P04912	95.39	1.4	0.41	3.45	5.65E-04	3.79E-02	uncharacterized LOC103976522
Ma03_g06930	ITC1587_Bchr3_P05785	275.51	1.4	0.39	3.59	3.31E-04	2.93E-02	putative abscisic acid-insensitive 5-like protein 5
Ma06_g30910	ITC1587_Bchr6_P17825	1316.99	1.39	0.29	4.78	1.72E-06	1.30E-03	dnaJ homolog subfamily B member 14
Ma11_g16920	ITC1587_Bchr11_P33682	31.97	1.39	0.48	2.91	3.65E-03	9.69E-02	uncharacterized LOC103971789
Ma04_g10870	ITC1587_Bchr4_P09355	243.7	1.39	0.36	3.81	1.36E-04	1.75E-02	uncharacterized LOC103981143
Ma07_g17820	ITC1587_BchrUn_random_ P38528	476.14	1.39	0.36	3.89	1.01E-04	1.43E-02	probable pyridoxal biosynthesis protein PDX1.1
Ma01_g19250	ITC1587_Bchr1_P02324	1158.72	1.38	0.34	4.06	4.92E-05	9.66E-03	Hypothetical protein
Ma04_g25620	ITC1587_Bchr1_P00553*	147.12	1.38	0.44	3.1	1.92E-03	6.98E-02	Hypothetical protein
Ma09_g09060	ITC1587_Bchr9_P25840	1465.93	1.37	0.3	4.5	6.64E-06	2.87E-03	cyclic nucleotide-gated ion channel 2-like
Ma03_g11730	ITC1587_Bchr3_P06239	2107.91	1.37	0.27	5.08	3.69E-07	4.49E-04	fructose-bisphosphate aldolase
Ma04_g17140	ITC1587_Bchr1_P00130*	57.94	1.37	0.43	3.18	1.48E-03	6.13E-02	trans-resveratrol di-O-methyltransferase-like
Ma10_g10330	ITC1587_Bchr10_P29766	276.03	1.36	0.27	5.09	3.55E-07	4.49E-04	beta-amylase-like
Ma11_g20890	ITC1587_Bchr11_P34045	926.16	1.35	0.42	3.2	1.37E-03	5.96E-02	serine carboxypeptidase-like 18
Ma08_g21280	ITC1587_Bchr8_P23742	2679.61	1.35	0.39	3.43	6.11E-04	4.02E-02	probable tocopherol cyclase
Ma01_g11990	ITC1587_Bchr3_P06506*	401.49	1.35	0.4	3.37	7.49E-04	4.38E-02	uncharacterized LOC103994153
Ma02_g01300	ITC1587_BchrUn_random_ P34862	541.93	1.35	0.42	3.18	1.46E-03	6.11E-02	protein REVEILLE 1-like
Ma09_g21720	ITC1587_BchrUn_random_ P35233	136.2	1.35	0.45	3.02	2.56E-03	8.10E-02	uncharacterized LOC103973189

Ma11_g09780	ITC1587_Bchr11_P32955	420.07	1.35	0.41	3.3	9.53E-04	4.96E-02	thioredoxin M-type
Ma06_g28850	ITC1587_Bchr6_P17637	1109.51	1.34	0.31	4.29	1.77E-05	5.25E-03	probable solanesyl-diphosphate synthase 3
Ma11_g00990	ITC1587_Bchr11_P31777	470.27	1.34	0.39	3.4	6.81E-04	4.25E-02	serine carboxypeptidase-like 7
Ma07_g18940	ITC1587_BchrUn_random_P35615	834.44	1.33	0.31	4.31	1.63E-05	5.13E-03	geraniol 8-hydroxylase-like
Ma07_g08090	ITC1587_Bchr7_P19286	649.66	1.33	0.38	3.55	3.78E-04	3.19E-02	uncharacterized LOC103990320
Ma04_g25580	ITC1587_Bchr1_P00553	2129.76	1.33	0.4	3.31	9.32E-04	4.91E-02	uncharacterized aarF domain-containing protein kinase At1g79600
Ma01_g21690	ITC1587_Bchr1_P02747	415.72	1.33	0.37	3.55	3.83E-04	3.20E-02	probable tocopherol O-methyltransferase
Ma02_g21100	ITC1587_Bchr2_P04798	443.89	1.32	0.42	3.17	1.51E-03	6.20E-02	uncharacterized LOC103976420
Ma07_g24710	ITC1587_Bchr7_P21086	1430.45	1.32	0.28	4.71	2.50E-06	1.55E-03	NAC domain-containing protein 48
Ma09_g17750	ITC1587_Bchr9_P26635	2425.86	1.32	0.32	4.12	3.79E-05	8.38E-03	homeobox-leucine zipper protein HOX16-like
Ma09_g09320	ITC1587_Bchr9_P25865	1254.05	1.31	0.36	3.69	2.20E-04	2.34E-02	thioredoxin-like protein HCF164
Ma11_g06470		275.53	1.31	0.23	5.62	1.95E-08	5.53E-05	uncharacterized LOC103970577
Ma01_g03440	ITC1587_Bchr1_P00738	2098.3	1.31	0.43	3.06	2.19E-03	7.42E-02	transcription factor MYC3-like
Ma01_g13520	ITC1587_Bchr1_P01778	497.62	1.31	0.39	3.35	8.01E-04	4.56E-02	protein NRT1/ PTR FAMILY 6.3-like
Ma09_g10470	ITC1587_Bchr9_P25967	3957.41	1.31	0.42	3.11	1.90E-03	6.94E-02	putative Horcolin
Ma08_g22980	ITC1587_Bchr8_P23987	160.23	1.31	0.41	3.2	1.38E-03	5.96E-02	uncharacterized LOC103995318
Ma04_g02690	ITC1587_Bchr5_P12484*	98.55	1.3	0.44	2.99	2.77E-03	8.54E-02	UDP-glucose 4-epimerase 1-like
Ma06_g26260	ITC1587_Bchr6_P17372	75.29	1.3	0.44	2.96	3.10E-03	8.99E-02	uncharacterized LOC103988992
Ma04_g33860	ITC1587_Bchr4_P11141	593.98	1.29	0.37	3.48	5.00E-04	3.63E-02	putative gamma-glutamylcyclotransferase At3g02910
Ma04_g11630	ITC1587_Bchr4_P09436	107.74	1.28	0.41	3.1	1.91E-03	6.94E-02	mitogen-activated protein kinase 2-like
Ma04_g31690	ITC1587_Bchr4_P10970	2736.04	1.28	0.38	3.37	7.48E-04	4.38E-02	pheophorbide a oxygenase
Ma05_g24400	ITC1587_Bchr5_P14094	374.26	1.28	0.38	3.38	7.19E-04	4.33E-02	CASP-like protein Os04g0281900
Ma03_g05720	ITC1587_Bchr3_P05677	810.28	1.28	0.35	3.66	2.54E-04	2.53E-02	putative Ethylene-responsive transcription factor 1
Ma09_g06790	ITC1587_Bchr9_P25639	306.04	1.27	0.39	3.22	1.27E-03	5.67E-02	phosphoglucan phosphatase LSF1
Ma03_g23770	ITC1587_Bchr3_P07542	471.68	1.26	0.29	4.36	1.30E-05	4.54E-03	uncharacterized LOC103979087
Ma09_g27610	ITC1587_Bchr9_P27991	110.51	1.26	0.38	3.37	7.59E-04	4.41E-02	probable mannan synthase 4

Ma10_g15710	ITC1587_Bchr10_P30300	305.11	1.26	0.41	3.07	2.17E-03	7.40E-02	heme-binding-like protein At3g10130
Ma10_g25240	ITC1587_Bchr10_P31117*	100.47	1.26	0.36	3.47	5.20E-04	3.66E-02	pheophytinase
Ma10_g27410	ITC1587_Bchr10_P31308	483.49	1.25	0.32	3.97	7.21E-05	1.20E-02	uncharacterized LOC103969387
Ma01_g00570	ITC1587_Bchr1_P00901	1218.96	1.25	0.41	3.02	2.51E-03	8.04E-02	uncharacterized LOC103981509
Ma03_g14610	ITC1587_Bchr3_P06604	2313.24	1.24	0.43	2.89	3.84E-03	9.94E-02	uncharacterized LOC103978260
Ma03_g05220	ITC1587_Bchr6_P15124*	97.15	1.24	0.36	3.42	6.21E-04	4.04E-02	zinc finger protein CONSTANS-LIKE 10-like
Ma06_g33490	ITC1587_Bchr9_P25534*	431.15	1.24	0.37	3.37	7.45E-04	4.38E-02	uncharacterized LOC103989609
Ma11_g12920	ITC1587_Bchr11_P33263	386.62	1.23	0.39	3.15	1.62E-03	6.34E-02	uncharacterized LOC103971402
Ma09_g00790	ITC1587_Bchr9_P25133	597.85	1.23	0.4	3.09	2.03E-03	7.18E-02	protein EXECUTER 1
Ma08_g15070	ITC1587_Bchr8_P23675	3701.31	1.23	0.37	3.33	8.56E-04	4.68E-02	phosphoglucomutase
Ma11_g08430	ITC1587_Bchr11_P32505	287.22	1.22	0.26	4.7	2.55E-06	1.55E-03	uncharacterized LOC103970739
Ma11_g09700	ITC1587_Bchr11_P32968	422.66	1.22	0.35	3.53	4.09E-04	3.31E-02	E3 ubiquitin-protein ligase CHIP-like
Ma09_g18030	ITC1587_BchrUn_random_ P35238*	105.13	1.22	0.34	3.55	3.79E-04	3.19E-02	uncharacterized LOC103973521
Ma09_g01310	ITC1587_Bchr9_P25181	59.57	1.21	0.37	3.28	1.02E-03	5.11E-02	CBS domain-containing protein CBSCBSPB5-like
Ma03_g17480	ITC1587_Bchr3_P06896	2286.12	1.21	0.42	2.91	3.61E-03	9.65E-02	F-box/kelch-repeat protein At1g67480-like
Ma10_g27850	ITC1587_Bchr10_P31341	950.99	1.21	0.38	3.16	1.56E-03	6.27E-02	potassium channel KAT3-like
Ma08_g04770	ITC1587_Bchr8_P21871	3747.94	1.21	0.37	3.25	1.15E-03	5.33E-02	4-hydroxy-3-methylbut-2-enyl diphosphate reductase
Ma03_g27180	ITC1587_Bchr3_P07836	185.42	1.21	0.32	3.74	1.86E-04	2.16E-02	PITH domain-containing protein 1
Ma11_g13840	ITC1587_Bchr8_P21560*	782.22	1.2	0.25	4.84	1.29E-06	1.07E-03	thioredoxin
Ma06_g02960	ITC1587_Bchr6_P14890	69.47	1.2	0.41	2.92	3.50E-03	9.51E-02	uncharacterized LOC103986642
Ma07_g08660	ITC1587_Bchr7_P19372	514.76	1.19	0.3	3.94	8.15E-05	1.30E-02	mitochondrial outer membrane protein porin 1-like
Ma01_g18420	ITC1587_Bchr1_P02154	165.02	1.18	0.36	3.3	9.72E-04	4.98E-02	haloacid dehalogenase-like hydrolase domain-containing protein Sgpp
Ma04_g24420	ITC1587_Bchr1_P00440	321.44	1.18	0.38	3.08	2.10E-03	7.28E-02	ABC transporter G family member 22-like
Ma07_g13740	ITC1587_Bchr7_P19838	2222.23	1.18	0.34	3.43	6.10E-04	4.02E-02	CBL-interacting protein kinase 5-like
Ma11_g19430	ITC1587_Bchr11_P33915	563.31	1.17	0.34	3.45	5.66E-04	3.79E-02	peroxisomal membrane protein 11-4

Ma10_g00620	ITC1587_Bchr8_P23775*	1289.75	1.17	0.4	2.94	3.31E-03	9.28E-02	transcription factor MYB1R1-like
Ma04_g20350	ITC1587_Bchr1_P00130	917.58	1.17	0.24	4.88	1.06E-06	9.33E-04	trans-resveratrol di-O-methyltransferase-like
Ma09_g25310	ITC1587_Bchr9_P27791	226.14	1.17	0.34	3.49	4.86E-04	3.60E-02	uncharacterized LOC103998888
Ma09_g17390	ITC1587_Bchr9_P26599	397.47	1.17	0.27	4.3	1.70E-05	5.13E-03	uncharacterized LOC103998278
Ma01_g21470	ITC1587_Bchr8_P24425*	317.7	1.17	0.39	2.95	3.17E-03	9.08E-02	protein DEHYDRATION-INDUCED 19-like
Ma01_g14940	ITC1587_BchrUn_random_P36900*	5971.48	1.16	0.29	4	6.44E-05	1.15E-02	inositol-3-phosphate synthase-like
Ma06_g33250	ITC1587_Bchr6_P18049	932.32	1.16	0.33	3.56	3.65E-04	3.13E-02	phytoene synthase 2
Ma03_g15750	ITC1587_Bchr3_P06478	507.52	1.15	0.3	3.83	1.28E-04	1.68E-02	protein LURP-one-related 5-like
Ma04_g16470	ITC1587_BchrUn_random_P36409	166.4	1.15	0.23	4.94	7.74E-07	8.08E-04	uncharacterized LOC103973729
Ma06_g19410	ITC1587_Bchr6_P16383	391.19	1.15	0.36	3.21	1.31E-03	5.80E-02	putative Methenyltetrahydrofolate synthase domain-containing protein
Ma03_g26080	ITC1587_Bchr2_P03269*	501.85	1.15	0.37	3.12	1.79E-03	6.77E-02	pyruvate
Ma10_g17670	ITC1587_Bchr10_P30473*	412.33	1.14	0.33	3.48	5.07E-04	3.63E-02	protein ELF4-LIKE 3-like
Ma09_g13780	ITC1587_Bchr9_P26256	188.36	1.14	0.37	3.1	1.95E-03	7.02E-02	uncharacterized LOC103997919
Ma05_g05370	ITC1587_Bchr5_P12157	365.08	1.14	0.39	2.94	3.27E-03	9.24E-02	BRI1 kinase inhibitor 1
Ma04_g18620	ITC1587_Bchr5_P12951*	285.39	1.14	0.31	3.64	2.70E-04	2.63E-02	arginase 1
Ma00_g01620	ITC1587_BchrUn_random_P34622*	158.96	1.13	0.3	3.83	1.26E-04	1.66E-02	putative Limonoid UDP-glucosyltransferase
Ma06_g18840	ITC1587_Bchr2_P04953*	2788.37	1.13	0.31	3.71	2.07E-04	2.26E-02	putative 12-oxophytodienoate reductase 11
Ma09_g20520	ITC1587_Bchr9_P27294	69.24	1.13	0.38	2.96	3.08E-03	8.95E-02	uncharacterized LOC103998523
Ma06_g02840	ITC1587_Bchr6_P14880	672.88	1.13	0.39	2.92	3.50E-03	9.51E-02	uncharacterized LOC103986630
Ma07_g26030	ITC1587_Bchr7_P21211	679.23	1.13	0.31	3.69	2.26E-04	2.36E-02	mitochondrial adenine nucleotide transporter ADNT1
Ma09_g28220	ITC1587_Bchr9_P28047	452.72	1.13	0.3	3.73	1.95E-04	2.19E-02	uncharacterized LOC103999206
Ma06_g14890	ITC1587_Bchr6_P15968	929.37	1.13	0.29	3.87	1.09E-04	1.50E-02	uncharacterized LOC103987719
Ma05_g17940	ITC1587_Bchr5_P13381	266.32	1.13	0.25	4.42	9.78E-06	3.84E-03	putative NAC domain-containing protein 94
Ma04_g04050	ITC1587_Bchr4_P08747	193.09	1.12	0.32	3.56	3.70E-04	3.14E-02	peptidyl-prolyl cis-trans isomerase FKBP16-1

Ma08_g04920	ITC1587_Bchr3_P07822*	218.56	1.12	0.34	3.28	1.04E-03	5.11E-02	squalene monooxygenase-like
Ma07_g07720	ITC1587_Bchr7_P19254	403.41	1.12	0.35	3.17	1.55E-03	6.25E-02	folate-biopterin transporter 1
Ma08_g13290	ITC1587_Bchr8_P22710	595.61	1.12	0.34	3.26	1.12E-03	5.27E-02	bifunctional nitrilase/nitrile hydratase NIT4A-like
Ma11_g05980	ITC1587_Bchr10_P31219*	1478.4	1.11	0.29	3.82	1.34E-04	1.73E-02	D-xylose-proton symporter-like 2
Ma04_g33790		509.85	1.11	0.34	3.24	1.18E-03	5.44E-02	2-C-methyl-D-erythritol 2
Ma01_g00620	ITC1587_Bchr1_P00907	933.07	1.1	0.34	3.28	1.04E-03	5.11E-02	probable anion transporter 6
Ma04_g33610	ITC1587_Bchr1_P00159*	235.34	1.09	0.35	3.11	1.89E-03	6.94E-02	uncharacterized LOC103982612
Ma03_g11970	ITC1587_Bchr3_P06263	958.54	1.09	0.28	3.98	7.02E-05	1.19E-02	beta-carotene isomerase D27
Ma10_g05910	ITC1587_Bchr10_P28438	1880.67	1.09	0.32	3.37	7.51E-04	4.38E-02	tropinone reductase homolog At1g07440-like
Ma03_g00500	ITC1587_Bchr3_P05210	832.19	1.09	0.34	3.26	1.13E-03	5.27E-02	uncharacterized LOC103977279
Ma07_g23140	ITC1587_Bchr7_P20939	1662.21	1.09	0.35	3.13	1.75E-03	6.66E-02	protein TRANSPARENT TESTA 12-like
Ma07_g26010	ITC1587_Bchr7_P21209	1120.82	1.09	0.35	3.13	1.73E-03	6.64E-02	RNA polymerase sigma factor sigE
Ma01_g09730	ITC1587_Bchr1_P01433	709.82	1.08	0.37	2.91	3.62E-03	9.65E-02	uncharacterized LOC103984127
Ma08_g02940	ITC1587_Bchr8_P21724*	197.66	1.08	0.37	2.91	3.58E-03	9.59E-02	uncharacterized LOC103993976
Ma09_g27710	ITC1587_Bchr9_P28000	753.26	1.08	0.37	2.91	3.66E-03	9.70E-02	protein WEAK CHLOROPLAST MOVEMENT UNDER BLUE LIGHT 1-like
Ma09_g24500	ITC1587_Bchr9_P27709	6504.48	1.08	0.3	3.63	2.84E-04	2.75E-02	ATP-dependent zinc metalloprotease FTSH 1
Ma06_g38570	ITC1587_Bchr6_P18512	635.93	1.08	0.33	3.26	1.11E-03	5.26E-02	protein PROTON GRADIENT REGULATION 5
Ma09_g24160	ITC1587_Bchr9_P27663	373.38	1.06	0.34	3.14	1.70E-03	6.57E-02	WD repeat-containing protein RUP2-like
Ma09_g14090	ITC1587_Bchr9_P26286	589.4	1.06	0.33	3.18	1.50E-03	6.16E-02	serine protease SPPA
Ma06_g29480	ITC1587_Bchr6_P17692	419.62	1.05	0.35	3.02	2.55E-03	8.09E-02	putative quinone-oxidoreductase homolog
Ma09_g06980	ITC1587_Bchr6_P15345*	322.13	1.05	0.32	3.26	1.12E-03	5.26E-02	probable WRKY transcription factor 75
Ma11_g10660	ITC1587_Bchr11_P32825	110.66	1.05	0.33	3.19	1.40E-03	5.99E-02	beta-carotene isomerase D27
Ma04_g06710	ITC1587_Bchr4_P08972	990.38	1.05	0.31	3.4	6.74E-04	4.23E-02	uncharacterized LOC103980786
Ma10_g20630	ITC1587_Bchr10_P30716	151.3	1.05	0.34	3.13	1.73E-03	6.65E-02	iron-sulfur assembly protein IscA-like 1
Ma03_g12650	ITC1587_Bchr3_P06320	4621.72	1.05	0.33	3.19	1.44E-03	6.06E-02	chromoplast-specific carotenoid-associated protein C1
Ma04_g10650	ITC1587_Bchr4_P09337	793.73	1.05	0.36	2.94	3.30E-03	9.26E-02	1

Ma08_g22240	ITC1587_Bchr8_P23921	436.25	1.04	0.32	3.23	1.25E-03	5.63E-02	putative uncharacterized protein
Ma08_g14090	ITC1587_Bchr8_P22632	4159.77	1.04	0.25	4.2	2.67E-05	6.73E-03	thioredoxin-like protein CDSP32
Ma04_g04350	ITC1587_Bchr4_P08771	123.58	1.03	0.32	3.18	1.45E-03	6.10E-02	uncharacterized LOC103980583
Ma03_g26530	ITC1587_Bchr3_P07779	1036.11	1.03	0.35	2.97	3.02E-03	8.88E-02	uncharacterized LOC103978840
Ma11_g07260	ITC1587_Bchr11_P32397	209.36	1.03	0.26	3.88	1.03E-04	1.44E-02	cyclin-dependent protein kinase inhibitor SIM-like
Ma09_g25900	ITC1587_Bchr9_P27847	412.06	1.03	0.35	2.91	3.56E-03	9.58E-02	uncharacterized LOC103998931
Ma08_g22800	ITC1587_Bchr8_P23971	342.11	1.03	0.28	3.69	2.25E-04	2.36E-02	regulatory protein NPR5-like
Ma09_g07420	ITC1587_Bchr9_P25701	2079.93	1.03	0.22	4.59	4.37E-06	2.26E-03	protein phosphatase 1 regulatory subunit 12A-like
Ma04_g36740	ITC1587_Bchr4_P11394	380.43	1.02	0.35	2.95	3.15E-03	9.06E-02	protein LOW PSII ACCUMULATION 1
Ma06_g00160	ITC1587_Bchr11_P32604	375.34	1.02	0.24	4.2	2.68E-05	6.73E-03	vacuolar amino acid transporter 1
Ma06_g07890	ITC1587_Bchr6_P15337	1998.81	1.02	0.33	3.11	1.90E-03	6.94E-02	uncharacterized LOC103987105
Ma05_g23360	ITC1587_Bchr11_P33897*	428.28	1.02	0.35	2.93	3.43E-03	9.41E-02	shaggy-related protein kinase gamma
Ma10_g25210	ITC1587_Bchr10_P31117	132.52	1.01	0.29	3.51	4.45E-04	3.52E-02	pheophytinase
Ma03_g01310	ITC1587_Bchr3_P05286	2007.11	1.01	0.29	3.52	4.28E-04	3.43E-02	uncharacterized LOC103977207
Ma05_g09080	ITC1587_Bchr5_P12498	3955.16	1.01	0.28	3.57	3.61E-04	3.10E-02	BURP domain-containing protein 3
Ma04_g21490	ITC1587_Bchr1_P00017	657.15	1.01	0.31	3.25	1.15E-03	5.35E-02	thioredoxin-like
Ma10_g22400	ITC1587_Bchr10_P30871	741.06	1	0.28	3.57	3.52E-04	3.05E-02	probable anion transporter 1
Ma08_g29400	ITC1587_Bchr8_P24564*	3607.32	1	0.28	3.56	3.68E-04	3.14E-02	phosphoglycolate phosphatase 1B
Ma03_g10090	ITC1587_Bchr3_P06097	239.72	1	0.34	2.91	3.65E-03	9.69E-02	uncharacterized LOC103977829
Ma06_g19830	ITC1587_Bchr6_P16441	2521.08	1	0.27	3.67	2.45E-04	2.50E-02	transmembrane protein 64
Ma10_g27480	ITC1587_Bchr10_P31312*	483.83	1	0.23	4.25	2.12E-05	5.94E-03	probable mitochondrial adenine nucleotide transporter BTL3
Ma01_g02440	ITC1587_Bchr1_P00648	1439.99	1	0.32	3.11	1.89E-03	6.94E-02	zinc finger protein MAGPIE-like
Ma08_g15380	ITC1587_Bchr11_P31707*	973.64	1	0.33	3.04	2.37E-03	7.77E-02	uncharacterized LOC103994605
Ma06_g23980	ITC1587_Bchr6_P16882*	236.55	1	0.25	3.98	7.03E-05	1.19E-02	uncharacterized LOC103988718
Ma07_g24600	ITC1587_Bchr7_P21077	8351.57	0.99	0.34	2.89	3.86E-03	1.00E-01	photosystem II 22 kDa protein
Ma07_g12230	ITC1587_Bchr7_P19691	101.77	0.99	0.33	2.98	2.87E-03	8.69E-02	protein trichome birefringence-like 14
Ma08_g02710	ITC1587_Bchr8_P21714	284.37	0.99	0.33	3.03	2.45E-03	7.92E-02	protein PHLOEM PROTEIN 2-LIKE A10-like

Ma01_g07320	ITC1587_Bchr1_P01219	74.17	0.99	0.33	2.99	2.75E-03	8.52E-02	uncharacterized LOC103986427
Ma08_g24630	ITC1587_Bchr8_P24135	512.43	0.99	0.28	3.54	4.02E-04	3.30E-02	TBC1 domain family member 5 homolog A-like
Ma04_g27280	ITC1587_Bchr4_P10557	80.82	0.99	0.3	3.29	9.98E-04	5.05E-02	uncharacterized LOC103983166
Ma08_g29220	ITC1587_Bchr8_P24550	695.91	0.98	0.31	3.14	1.69E-03	6.57E-02	uncharacterized LOC103996007
Ma04_g28910	ITC1587_Bchr4_P10712	404.75	0.98	0.31	3.16	1.60E-03	6.31E-02	uncharacterized protein C6C3.02c-like
Ma04_g34400	ITC1587_Bchr4_P11186	3200.04	0.98	0.31	3.16	1.58E-03	6.30E-02	MATE efflux family protein LAL5-like
Ma03_g16840	ITC1587_BchrUn_random_ P39504	4450.38	0.98	0.28	3.48	5.04E-04	3.63E-02	transketolase
Ma05_g07190	ITC1587_Bchr2_P04766*	899.73	0.98	0.25	3.85	1.19E-04	1.61E-02	macrophage migration inhibitory factor homolog
Ma04_g10310	ITC1587_Bchr4_P09300*	135.79	0.97	0.3	3.24	1.18E-03	5.43E-02	uncharacterized LOC103981098
Ma08_g21090	ITC1587_Bchr8_P23757*	477.95	0.97	0.33	2.93	3.36E-03	9.31E-02	uncharacterized LOC103995066
Ma05_g18580	ITC1587_Bchr5_P13359	488.25	0.97	0.32	3.06	2.21E-03	7.46E-02	uncharacterized LOC103985194
Ma04_g26120	ITC1587_Bchr4_P10454	1422.41	0.96	0.29	3.3	9.69E-04	4.98E-02	probable galactinol--sucrose galactosyltransferase 2
Ma07_g07390	ITC1587_Bchr7_P19218	506.52	0.96	0.27	3.6	3.12E-04	2.84E-02	pyridoxal biosynthesis protein PDX2
Ma05_g04030	ITC1587_Bchr5_P12036	436.93	0.96	0.26	3.62	2.97E-04	2.81E-02	putative Zinc finger protein CONSTANS-LIKE 9
Ma08_g26550	ITC1587_Bchr4_P10458*	420.76	0.96	0.32	2.96	3.03E-03	8.89E-02	nudix hydrolase 2-like
Ma04_g23720	ITC1587_Bchr1_P00374	207.51	0.96	0.26	3.73	1.90E-04	2.16E-02	acyl-coenzyme A thioesterase 13
Ma09_g27420	ITC1587_Bchr9_P27974	1570.95	0.95	0.26	3.73	1.89E-04	2.16E-02	uncharacterized LOC103999142
Ma03_g14950	ITC1587_Bchr3_P06554	1036.95	0.95	0.31	3.02	2.50E-03	8.04E-02	uncharacterized LOC103978235
Ma01_g21420		1168.4	0.94	0.28	3.39	6.96E-04	4.28E-02	uncharacterized LOC103999804
Ma08_g19610	ITC1587_Bchr8_P22966	5813.7	0.94	0.32	2.95	3.22E-03	9.14E-02	Alanine aminotransferase 2
Ma02_g14500	ITC1587_Bchr2_P04215	3332.44	0.94	0.23	4.16	3.19E-05	7.50E-03	2-methyl-6-phytyl-1
Ma08_g08050	ITC1587_Bchr8_P22160	2295.32	0.94	0.3	3.11	1.88E-03	6.94E-02	uncharacterized LOC103993528
Ma08_g05160	ITC1587_Bchr8_P21903	872.6	0.94	0.32	2.97	2.97E-03	8.80E-02	cellulose synthase-like protein G3
Ma05_g18980	ITC1587_Bchr5_P13609	447.99	0.94	0.3	3.08	2.07E-03	7.25E-02	glutathione S-transferase PARB-like
Ma10_g20940	ITC1587_Bchr10_P30739	145.63	0.94	0.27	3.48	5.05E-04	3.63E-02	uncharacterized LOC103968822
Ma11_g14600	ITC1587_Bchr11_P33466	646.12	0.94	0.22	4.31	1.66E-05	5.13E-03	putative uncharacterized hydrolase YKL033W-A

Ma06_g27140	ITC1587_Bchr6_P17481	6218.83	0.93	0.27	3.45	5.58E-04	3.78E-02	Actin-3
Ma08_g06120	ITC1587_Bchr8_P21978	4789.15	0.93	0.27	3.45	5.60E-04	3.78E-02	chloroplast stem-loop binding protein of 41 kDa b
Ma06_g02300	ITC1587_Bchr6_P14834	1397.52	0.93	0.31	3.03	2.47E-03	7.99E-02	receptor-like serine/threonine-protein kinase SD1-8
Ma06_g05630	ITC1587_Bchr6_P15148	200.55	0.93	0.25	3.66	2.54E-04	2.53E-02	uncharacterized LOC103986915
Ma04_g11370	ITC1587_Bchr4_P09407	484.46	0.93	0.32	2.91	3.59E-03	9.61E-02	ubiquinol oxidase 4
Ma04_g07800	ITC1587_Bchr4_P09067	2206.35	0.93	0.32	2.93	3.36E-03	9.31E-02	2-methylene-furan-3-one reductase
Ma03_g02630	ITC1587_Bchr3_P05405*	2264.07	0.93	0.27	3.46	5.36E-04	3.69E-02	uncharacterized LOC103977086
Ma09_g10620	ITC1587_Bchr8_P22910*	496.78	0.92	0.25	3.66	2.49E-04	2.53E-02	uncharacterized LOC103997597
Ma01_g19020	ITC1587_Bchr1_P02082	399.83	0.92	0.23	4.09	4.37E-05	9.00E-03	uncharacterized LOC103995705
Ma09_g22470	ITC1587_Bchr9_P27498	540.68	0.92	0.31	2.96	3.07E-03	8.93E-02	two-pore potassium channel 3
Ma03_g17570	ITC1587_Bchr3_P07029	339.34	0.92	0.22	4.12	3.84E-05	8.38E-03	chaperone protein dnaJ 20
Ma04_g19840	ITC1587_Bchr3_P07123	1843.45	0.92	0.26	3.53	4.11E-04	3.31E-02	peroxisomal membrane protein 11C-like
Ma09_g14380	ITC1587_Bchr9_P26314	2801.38	0.91	0.29	3.19	1.42E-03	6.02E-02	isoamylase 3
Ma09_g03980	ITC1587_Bchr9_P25415	456.9	0.91	0.29	3.2	1.38E-03	5.96E-02	inner membrane protein PPF-1
Ma05_g11780	ITC1587_Bchr5_P12737	1053.04	0.91	0.31	2.95	3.22E-03	9.14E-02	ferredoxin-thioredoxin reductase
Ma07_g19200	ITC1587_Bchr7_P20576	362.56	0.91	0.3	3.02	2.52E-03	8.04E-02	shikimate kinase 3
Ma10_g15170	ITC1587_Bchr10_P30255	2582.09	0.91	0.21	4.28	1.89E-05	5.48E-03	glutaredoxin domain-containing cysteine-rich protein CG12206
Ma01_g03650		119.14	0.91	0.26	3.54	4.03E-04	3.30E-02	sulfiredoxin
Ma10_g27080	ITC1587_Bchr10_P31270	315.17	0.9	0.27	3.38	7.38E-04	4.37E-02	uncharacterized LOC103969357
Ma08_g28820	ITC1587_Bchr8_P24519	2554.97	0.9	0.3	2.98	2.91E-03	8.72E-02	photosystem I reaction center subunit XI
Ma04_g07100	ITC1587_Bchr4_P09007	2149.93	0.9	0.28	3.22	1.27E-03	5.67E-02	tryptophan synthase beta chain 1
Ma06_g35590	ITC1587_Bchr6_P18236	2364.53	0.9	0.26	3.38	7.17E-04	4.33E-02	phosphomethylpyrimidine synthase
Ma09_g13570	ITC1587_Bchr9_P26239	205.84	0.89	0.27	3.31	9.42E-04	4.95E-02	AIG2-like protein
Ma07_g23480	ITC1587_Bchr7_P20968	1682.92	0.89	0.28	3.15	1.61E-03	6.31E-02	Ser/Thr protein kinase
Ma07_g12010	ITC1587_Bchr7_P19666	2479.93	0.89	0.29	3.05	2.25E-03	7.56E-02	probable alanine--tRNA ligase
Ma06_g34980	ITC1587_Bchr6_P18204	270.2	0.89	0.26	3.39	6.91E-04	4.26E-02	uncharacterized LOC103989746
Ma06_g28350	ITC1587_Bchr6_P17587*	1127.65	0.87	0.26	3.32	9.16E-04	4.86E-02	uncharacterized LOC103989152
Ma05_g28600	ITC1587_Bchr5_P14468	1309.8	0.86	0.29	3.02	2.50E-03	8.04E-02	uncharacterized LOC103986353

Ma04_g22620	ITC1587_Bchr1_P00260*	8707.53	0.86	0.29	2.98	2.88E-03	8.69E-02	ferredoxin--NADP reductase
Ma10_g05790	ITC1587_Bchr10_P28448	6464.69	0.86	0.25	3.46	5.35E-04	3.69E-02	fructose-1
Ma06_g10680	ITC1587_Bchr6_P15591	1072.81	0.86	0.26	3.26	1.12E-03	5.26E-02	probable monodehydroascorbate reductase
Ma11_g17790	ITC1587_Bchr11_P33757	441.01	0.86	0.27	3.16	1.58E-03	6.30E-02	pheophytinase
Ma08_g16610	ITC1587_Bchr8_P22768	492.71	0.86	0.29	2.93	3.42E-03	9.41E-02	peptidyl-prolyl cis-trans isomerase CYP26-2
Ma09_g28770	ITC1587_Bchr7_P20801*	1484.64	0.86	0.22	3.99	6.71E-05	1.17E-02	ferredoxin-thioredoxin reductase catalytic chain
Ma01_g00970	ITC1587_Bchr1_P00937*	226.46	0.86	0.29	2.93	3.36E-03	9.31E-02	unknown protein DS12 from 2D-PAGE of leaf
Ma07_g17090	ITC1587_Bchr11_P33828*	454.02	0.86	0.29	2.98	2.89E-03	8.71E-02	uncharacterized LOC103992068
Ma04_g38310	ITC1587_Bchr4_P11515*	2359.85	0.85	0.27	3.16	1.59E-03	6.31E-02	uncharacterized oxidoreductase At1g06690
Ma06_g05400	ITC1587_Bchr5_P14153*	716.8	0.85	0.27	3.19	1.43E-03	6.04E-02	probable protein phosphatase 2C 59
Ma11_g02110	ITC1587_Bchr11_P31885	708.86	0.85	0.26	3.3	9.71E-04	4.98E-02	putative transporter arsB
Ma07_g15660	ITC1587_Bchr7_P20028	434.72	0.85	0.28	3.01	2.59E-03	8.14E-02	protein PHR1-LIKE 1-like
Ma02_g05750	ITC1587_Bchr2_P03447	567.53	0.85	0.29	2.9	3.78E-03	9.84E-02	Thioredoxin X
Ma11_g11100	ITC1587_BchrUn_random_P34470	10452.98	0.85	0.28	2.98	2.86E-03	8.69E-02	protein CHUP1
Ma11_g15570	ITC1587_Bchr11_P33560	480.39	0.84	0.24	3.5	4.64E-04	3.56E-02	thylakoid lumenal 15.0 kDa protein 2
Ma06_g28550	ITC1587_Bchr6_P17607	679.73	0.84	0.23	3.66	2.53E-04	2.53E-02	phosphoenolpyruvate carboxylase 2-like
Ma09_g16480	ITC1587_Bchr9_P26565	462.87	0.84	0.29	2.9	3.72E-03	9.75E-02	uncharacterized LOC103998234
Ma11_g01390	ITC1587_Bchr11_P31815	6806.84	0.84	0.23	3.58	3.42E-04	2.98E-02	glyceraldehyde-3-phosphate dehydrogenase B
Ma03_g09050	ITC1587_Bchr3_P06001	1816.31	0.83	0.21	3.89	1.02E-04	1.43E-02	cytochrome P450 2A2-like
Ma03_g16830	ITC1587_BchrUn_random_P36196	2395.37	0.83	0.21	3.95	7.75E-05	1.27E-02	transketolase
Ma01_g00130	ITC1587_Bchr1_P00861	4241.12	0.83	0.27	3.07	2.11E-03	7.28E-02	glucose-1-phosphate adenylyltransferase small subunit
Ma05_g15060	ITC1587_Bchr5_P13017	1736.5	0.82	0.26	3.2	1.39E-03	5.99E-02	WD repeat-containing protein YMR102C-like
Ma06_g03540	ITC1587_Bchr5_P14153*	1310.41	0.82	0.27	3.05	2.27E-03	7.58E-02	probable protein phosphatase 2C 59
Ma04_g10140	ITC1587_Bchr4_P09286	1281.28	0.82	0.25	3.31	9.32E-04	4.91E-02	zerumbone synthase

Ma02_g04410	ITC1587_Bchr2_P03315	414.99	0.82	0.26	3.12	1.78E-03	6.75E-02	methionine aminopeptidase 1B
Ma06_g21080	ITC1587_Bchr6_P16573	2370.2	0.82	0.24	3.35	8.14E-04	4.60E-02	lactoylglutathione lyase-like
Ma11_g18290	ITC1587_Bchr11_P33805	562.17	0.81	0.22	3.68	2.35E-04	2.44E-02	cysteine desulfurase 2
Ma02_g14460	ITC1587_Bchr2_P04212	161.27	0.81	0.24	3.41	6.51E-04	4.14E-02	sec-independent protein translocase protein TATA
Ma08_g24540	ITC1587_Bchr7_P20021*	534.13	0.8	0.22	3.63	2.88E-04	2.78E-02	protein TIFY 3B-like
Ma10_g20430	ITC1587_Bchr10_P30699	576.55	0.8	0.21	3.85	1.18E-04	1.61E-02	iron-sulfur cluster assembly protein 1-like
Ma02_g14580	ITC1587_Bchr2_P04223	556.1	0.8	0.24	3.3	9.68E-04	4.98E-02	uncharacterized LOC103975690
Ma08_g15730	ITC1587_Bchr8_P22821	581.18	0.8	0.23	3.51	4.54E-04	3.53E-02	pentatricopeptide repeat-containing protein At1g31920
Ma03_g14330	ITC1587_BchrUn_random_ P35636	575.59	0.8	0.25	3.22	1.29E-03	5.72E-02	zinc finger protein ZAT5-like
Ma06_g21850	ITC1587_Bchr6_P16669	298.34	0.8	0.27	2.95	3.20E-03	9.14E-02	uncharacterized LOC103988620
Ma06_g04330	ITC1587_Bchr6_P15012	432.14	0.79	0.26	3.04	2.38E-03	7.80E-02	malate dehydrogenase
Ma01_g02980	ITC1587_Bchr1_P00692	177.68	0.79	0.24	3.34	8.26E-04	4.62E-02	uncharacterized LOC103979376
Ma02_g17510	ITC1587_Bchr2_P04481	510.3	0.79	0.26	3.04	2.34E-03	7.70E-02	putative GATA transcription factor 22
Ma10_g06980	ITC1587_Bchr11_P32484*	1047.74	0.79	0.26	3.02	2.56E-03	8.10E-02	zinc finger CCCH domain-containing protein ZFN-like
Ma05_g21330	ITC1587_Bchr5_P13760	7644.63	0.78	0.22	3.51	4.47E-04	3.52E-02	glycerate dehydrogenase
Ma06_g01340	ITC1587_Bchr11_P32705*	596.07	0.78	0.23	3.36	7.81E-04	4.45E-02	uncharacterized LOC103971095
Ma04_g12290	ITC1587_Bchr4_P09501	969.43	0.78	0.26	3.02	2.51E-03	8.04E-02	uncharacterized protein At1g32220
Ma11_g04890	ITC1587_Bchr11_P32156	3172.33	0.78	0.21	3.67	2.45E-04	2.50E-02	calvin cycle protein CP12-1
Ma03_g26760	ITC1587_Bchr3_P07798*	1263.25	0.77	0.2	3.89	1.01E-04	1.43E-02	uncharacterized LOC103978816
Ma07_g24440	ITC1587_Bchr7_P21058	957.47	0.77	0.19	4.11	3.99E-05	8.55E-03	CBS domain-containing protein CBSCBSPB1-like
Ma02_g21410	ITC1587_Bchr5_P12227*	299.32	0.77	0.24	3.15	1.63E-03	6.37E-02	iron-sulfur assembly protein IscA
Ma09_g00620	ITC1587_Bchr9_P25117	395.71	0.76	0.22	3.45	5.63E-04	3.79E-02	Hypothetical protein
Ma09_g02610	ITC1587_Bchr9_P25301	542.67	0.76	0.22	3.49	4.75E-04	3.57E-02	uncharacterized LOC103996785
Ma04_g07230	ITC1587_Bchr4_P09022	771.76	0.76	0.23	3.32	9.10E-04	4.85E-02	uncharacterized LOC103980838
Ma06_g00630	ITC1587_Bchr11_P32644	1164.64	0.76	0.22	3.47	5.14E-04	3.66E-02	mitochondrial dicarboxylate/tricarboxylate transporter DTC-like
Ma08_g32500	ITC1587_BchrUn_random_	544.94	0.76	0.22	3.36	7.67E-04	4.43E-02	Probable hydroxyacylglutathione

	P38371*							hydrolase 2
Ma04_g05050	ITC1587_Bchr4_P08837	372.79	0.75	0.24	3.11	1.88E-03	6.94E-02	U-box domain-containing protein 44-like
Ma04_g24520	ITC1587_Bchr1_P00453	376.96	0.75	0.25	3.07	2.17E-03	7.40E-02	peptide deformylase 1B
Ma07_g25820	ITC1587_Bchr7_P21188	504.48	0.75	0.24	3.11	1.87E-03	6.94E-02	eukaryotic translation initiation factor 1A-like
Ma07_g07340	ITC1587_Bchr7_P19210*	1496.81	0.75	0.25	2.96	3.06E-03	8.93E-02	uncharacterized LOC103990389
Ma02_g20100	ITC1587_Bchr2_P04716	1492.25	0.75	0.24	3.08	2.08E-03	7.27E-02	uncharacterized LOC103975098
Ma07_g28850	ITC1587_Bchr7_P21430	2169.37	0.75	0.2	3.7	2.17E-04	2.33E-02	uncharacterized LOC103993077
Ma09_g11820	ITC1587_Bchr9_P27755	460.55	0.75	0.24	3.05	2.26E-03	7.57E-02	wound induced protein
Ma04_g15280	ITC1587_Bchr4_P09787*	376.35	0.74	0.23	3.3	9.54E-04	4.96E-02	uncharacterized LOC103981506
Ma03_g31990	ITC1587_Bchr3_P08228	141.15	0.74	0.26	2.91	3.57E-03	9.58E-02	uncharacterized LOC103979791
Ma06_g25570	ITC1587_Bchr6_P17334	3582.05	0.74	0.24	3.13	1.77E-03	6.69E-02	potassium channel AKT2
Ma01_g07940	ITC1587_Bchr1_P01281	989.14	0.73	0.25	2.92	3.52E-03	9.54E-02	thioredoxin-like 2
Ma09_g13240	ITC1587_Bchr9_P26207	1385.91	0.73	0.22	3.25	1.17E-03	5.40E-02	VIN3-like protein 2
Ma10_g26800	ITC1587_Bchr10_P31248	1577.78	0.73	0.22	3.37	7.41E-04	4.38E-02	zinc finger protein CONSTANS-LIKE 16-like
Ma03_g01630	ITC1587_Bchr3_P05316	690.32	0.73	0.25	2.93	3.36E-03	9.31E-02	uncharacterized LOC103977179
Ma07_g23840	ITC1587_Bchr7_P21003	260.84	0.72	0.2	3.62	2.98E-04	2.81E-02	Predicted protein
Ma09_g22750	ITC1587_Bchr9_P27526	1038.72	0.72	0.2	3.54	3.94E-04	3.28E-02	farnesyl pyrophosphate synthase 1-like
Ma03_g03180	ITC1587_Bchr3_P05450	641.62	0.72	0.24	2.97	3.02E-03	8.88E-02	VHS domain-containing protein At3g16270-like
Ma07_g19710	ITC1587_Bchr7_P20625	2056.83	0.72	0.25	2.89	3.80E-03	9.86E-02	uncharacterized LOC103992165
Ma03_g20140	ITC1587_Bchr3_P07230	399.07	0.72	0.23	3.11	1.85E-03	6.90E-02	outer envelope pore protein 37
Ma04_g14270	ITC1587_Bchr4_P09684	310.52	0.72	0.21	3.36	7.71E-04	4.45E-02	pentatricopeptide repeat-containing protein At3g29290
Ma05_g30420	ITC1587_Bchr5_P14641	1155.06	0.72	0.24	2.98	2.90E-03	8.71E-02	gamma-interferon-inducible lysosomal thiol reductase-like
Ma09_g25040	ITC1587_Bchr9_P27761	7774.79	0.71	0.24	2.9	3.70E-03	9.73E-02	cytochrome b6-f complex iron-sulfur subunit
Ma09_g20320	ITC1587_BchrUn_random_ P36341	564.11	0.71	0.21	3.43	6.14E-04	4.03E-02	uncharacterized LOC103973687
Ma07_g03120	ITC1587_Bchr7_P18830	721.63	0.71	0.23	3.07	2.11E-03	7.28E-02	UDP-glucuronate:xylan alpha-glucuronosyltransferase 1-like

Ma09_g21670	ITC1587_BchrUn_random_P35240	1231.93	0.71	0.24	2.98	2.92E-03	8.73E-02	cytochrome P450 89A2-like
Ma09_g29100	ITC1587_Bchr7_P19503*	1057.87	0.7	0.24	2.94	3.34E-03	9.31E-02	14-3-3-like protein GF14-C
Ma06_g06810	ITC1587_Bchr6_P15242	1383.96	0.7	0.2	3.43	6.03E-04	4.00E-02	heme oxygenase 1
Ma11_g23550	ITC1587_Bchr11_P34263	4137.81	0.7	0.17	4.05	5.15E-05	9.80E-03	uncharacterized protein At4g15545
Ma07_g28530	ITC1587_Bchr4_P08676*	886.2	0.7	0.24	2.9	3.78E-03	9.84E-02	uncharacterized LOC103993105
Ma02_g15760	ITC1587_Bchr2_P04321	1159.74	0.7	0.16	4.27	1.93E-05	5.53E-03	uncharacterized LOC103975593
Ma07_g24980	ITC1587_Bchr7_P21109	233.55	0.7	0.21	3.25	1.16E-03	5.38E-02	uncharacterized LOC103992643
Ma03_g16620	ITC1587_Bchr3_P06921	744.54	0.69	0.22	3.12	1.79E-03	6.75E-02	probable serine acetyltransferase 1
Ma06_g36970	ITC1587_Bchr6_P18362	1872.81	0.69	0.19	3.69	2.22E-04	2.35E-02	probable protein phosphatase 2C 55
Ma03_g23700	ITC1587_BchrUn_random_P39831	411.01	0.69	0.22	3.19	1.41E-03	6.01E-02	uncharacterized LOC103974166
Ma07_g23520	ITC1587_Bchr11_P33804*	2034.22	0.69	0.24	2.93	3.44E-03	9.43E-02	vacuolar-sorting receptor 1
Ma06_g32870	ITC1587_Bchr6_P18012	433.7	0.68	0.17	3.93	8.43E-05	1.31E-02	putative oxidoreductase TDA3
Ma11_g01060	ITC1587_Bchr11_P31782	301.06	0.68	0.16	4.32	1.53E-05	4.96E-03	uncharacterized LOC103970118
Ma11_g10060	ITC1587_Bchr9_P27483*	486.79	0.68	0.21	3.23	1.26E-03	5.65E-02	clavaminate synthase-like protein At3g21360
Ma09_g18000	ITC1587_BchrUn_random_P36187	493.98	0.68	0.2	3.34	8.28E-04	4.62E-02	psbP domain-containing protein 3
Ma10_g11570	ITC1587_Bchr10_P29946	676.86	0.67	0.19	3.47	5.25E-04	3.66E-02	U-box domain-containing protein 4-like
Ma06_g29830	ITC1587_Bchr6_P17730	278.64	0.66	0.21	3.15	1.63E-03	6.37E-02	protein FLX-like 4
Ma09_g23180	ITC1587_Bchr9_P27572	236.18	0.66	0.22	2.95	3.22E-03	9.14E-02	uncharacterized LOC103998702
Ma07_g22850	ITC1587_Bchr7_P20917	393.62	0.65	0.2	3.24	1.20E-03	5.47E-02	cytochrome c-type biogenesis ccda-like chloroplastic protein 1
Ma01_g06530	ITC1587_Bchr11_P33897*	1322.9	0.64	0.21	3.06	2.21E-03	7.47E-02	shaggy-related protein kinase alpha-like
Ma03_g07060	ITC1587_Bchr3_P05796	233.73	0.64	0.22	2.95	3.14E-03	9.05E-02	cation transport regulator-like protein 2
Ma05_g20120	ITC1587_Bchr5_P13613	306.36	0.64	0.2	3.16	1.60E-03	6.31E-02	dicarboxylate transporter 2.1
Ma05_g21470	ITC1587_Bchr5_P13774	694.56	0.64	0.19	3.32	9.14E-04	4.86E-02	probable serine acetyltransferase 2
Ma09_g29580	ITC1587_Bchr9_P28175*	451.13	0.64	0.17	3.79	1.51E-04	1.88E-02	uncharacterized LOC103999328
Ma05_g23970	ITC1587_Bchr5_P14042	999.85	0.64	0.19	3.28	1.04E-03	5.11E-02	uncharacterized LOC103985583
Ma03_g20110	ITC1587_BchrUn_random_P38265	577.12	0.63	0.22	2.92	3.54E-03	9.57E-02	uncharacterized LOC103974637

Ma01_g05350	ITC1587_Bchr4_P08453*	359.22	0.63	0.2	3.09	2.01E-03	7.15E-02	uncharacterized LOC103988257
Ma06_g16850	ITC1587_Bchr6_P16149	356.19	0.63	0.18	3.41	6.56E-04	4.16E-02	THO complex subunit 4A-like
Ma07_g12990	ITC1587_Bchr7_P19765	508.4	0.62	0.19	3.36	7.79E-04	4.45E-02	uncharacterized LOC103991554
Ma03_g06520	ITC1587_Bchr9_P27351*	1363.43	0.62	0.19	3.27	1.06E-03	5.14E-02	histone H3.3
Ma11_g13000	ITC1587_Bchr3_P07539*	1398.02	0.62	0.19	3.22	1.26E-03	5.65E-02	uncharacterized LOC103971395
Ma04_g01170	ITC1587_Bchr4_P08485	751.82	0.62	0.19	3.28	1.03E-03	5.11E-02	50S ribosomal protein L31
Ma08_g30240		335.37	0.62	0.21	2.97	2.99E-03	8.83E-02	mediator of RNA polymerase II transcription subunit 7a-like
Ma07_g27060	ITC1587_Bchr7_P21287	651.54	0.61	0.2	3.09	2.00E-03	7.14E-02	putative glucose-6-phosphate 1-epimerase
Ma03_g31400	ITC1587_Bchr3_P08177	388.87	0.61	0.21	2.97	2.98E-03	8.83E-02	uncharacterized LOC103979848
Ma02_g22200	ITC1587_Bchr2_P04910	414.3	0.61	0.2	2.98	2.87E-03	8.69E-02	probable aspartyl aminopeptidase
Ma11_g01360	ITC1587_Bchr11_P31811	689.95	0.61	0.18	3.46	5.47E-04	3.74E-02	protein REVEILLE 1-like
Ma11_g12560	ITC1587_Bchr11_P33220	3227.56	0.61	0.17	3.62	2.95E-04	2.80E-02	dihydrolipoyl dehydrogenase
Ma02_g04790	ITC1587_Bchr2_P03348*	278.04	0.61	0.21	2.94	3.29E-03	9.26E-02	protein ULTRAPETALA 1-like
Ma10_g15890	ITC1587_Bchr10_P30311*	394.61	0.6	0.2	2.96	3.11E-03	8.99E-02	uncharacterized LOC103968373
Ma08_g10710	ITC1587_Bchr8_P22411*	616.31	0.6	0.2	3.07	2.14E-03	7.33E-02	protein trigalactosyldiacylglyceroL 4
Ma08_g31020	ITC1587_Bchr8_P24725	428.26	0.6	0.17	3.5	4.68E-04	3.56E-02	ubiquinone biosynthesis protein COQ9
Ma02_g09700	ITC1587_Bchr2_P03802	558.76	0.6	0.2	2.94	3.28E-03	9.25E-02	uncharacterized LOC103976082
Ma05_g25730	ITC1587_Bchr5_P14210	699.73	0.6	0.2	2.95	3.16E-03	9.08E-02	uncharacterized LOC103985431
Ma08_g08510	ITC1587_Bchr8_P22206	365.48	0.59	0.2	2.95	3.21E-03	9.14E-02	uncharacterized LOC103993487
Ma06_g22070	ITC1587_Bchr6_P16689	722.05	0.59	0.17	3.49	4.91E-04	3.62E-02	SWI/SNF complex component SNF12 homolog
Ma06_g18960	ITC1587_Bchr6_P16350*	748.57	0.59	0.18	3.32	8.96E-04	4.82E-02	psbP domain-containing protein 4
Ma04_g02650	ITC1587_Bchr4_P08632	331.18	-0.62	0.18	-3.48	5.01E-04	3.63E-02	uncharacterized LOC103980235
Ma06_g23180	ITC1587_BchrUn_random_ P37087	222.27	-0.64	0.21	-3	2.71E-03	8.45E-02	uncharacterized LOC103974036
Ma08_g19590	ITC1587_Bchr8_P22968	2287.61	-0.66	0.23	-2.92	3.46E-03	9.45E-02	Hypothetical protein
Ma06_g18480	ITC1587_Bchr6_P16305	440.51	-0.67	0.19	-3.51	4.55E-04	3.53E-02	uncharacterized LOC103988027
Ma08_g04220	ITC1587_Bchr8_P21820	244.39	-0.69	0.24	-2.91	3.58E-03	9.59E-02	protein NLP3-like
Ma04_g00250	ITC1587_Bchr4_P08398	1184.13	-0.7	0.23	-2.99	2.77E-03	8.54E-02	probable sugar phosphate/phosphate translocator At5g25400

Ma01_g16950	ITC1587_Bchr2_P03688*	247.52	-0.72	0.23	-3.11	1.85E-03	6.90E-02	AP-1 complex subunit mu-2-like
Ma03_g00670	ITC1587_Bchr3_P05227	840.87	-0.74	0.22	-3.28	1.04E-03	5.11E-02	REF/SRPP-like protein OsI_017815
Ma05_g28700	ITC1587_Bchr11_P34326*	483.79	-0.76	0.2	-3.75	1.75E-04	2.09E-02	diacylglycerol kinase 5-like
Ma07_g11270	ITC1587_Bchr7_P19596	159.02	-0.76	0.23	-3.33	8.63E-04	4.70E-02	F-box only protein 13-like
Ma10_g29790	ITC1587_Bchr10_P31519	235.86	-0.79	0.25	-3.18	1.50E-03	6.16E-02	probable galacturonosyltransferase 9
Ma08_g14790	ITC1587_Bchr8_P23645	1396.29	-0.82	0.22	-3.69	2.20E-04	2.34E-02	phosphoenolpyruvate carboxykinase [ATP]-like
Ma02_g06960		298.87	-0.82	0.28	-2.91	3.56E-03	9.58E-02	Hypothetical protein
Ma05_g06390	ITC1587_Bchr5_P12257	272.13	-0.83	0.27	-3.04	2.34E-03	7.71E-02	uncharacterized LOC103984114
Ma05_g11810	ITC1587_Bchr5_P12740	840.4	-0.84	0.27	-3.11	1.86E-03	6.91E-02	Zinc finger protein
Ma02_g07090	ITC1587_Bchr2_P03568*	128.04	-0.84	0.28	-2.98	2.84E-03	8.65E-02	uncharacterized LOC103972446
Ma06_g31890	ITC1587_Bchr6_P17918	718.08	-0.84	0.25	-3.4	6.65E-04	4.19E-02	probable xyloglucan glycosyltransferase 9
Ma05_g02670	ITC1587_Bchr4_P10631*	2736.29	-0.85	0.27	-3.18	1.45E-03	6.10E-02	5-methyltetrahydropteroyltriglutamate--homocysteine methyltransferase 2-like
Ma04_g33130	ITC1587_Bchr4_P11082	167.66	-0.86	0.29	-2.97	2.96E-03	8.78E-02	uncharacterized LOC103982647
Ma07_g25770	ITC1587_Bchr7_P21184	682.54	-0.87	0.28	-3.11	1.88E-03	6.94E-02	auxin response factor 18-like
Ma03_g03160	ITC1587_Bchr3_P05448	1080.39	-0.88	0.3	-2.93	3.37E-03	9.33E-02	plasma membrane-associated cation-binding protein 1-like
Ma08_g10690	ITC1587_Bchr8_P22409	494.93	-0.89	0.3	-2.94	3.32E-03	9.28E-02	uncharacterized LOC103993304
Ma05_g30340	ITC1587_Bchr5_P14635	435.08	-0.89	0.2	-4.43	9.56E-06	3.82E-03	uncharacterized LOC103986200
Ma05_g01540	ITC1587_Bchr4_P11574*	748	-0.89	0.18	-4.89	1.00E-06	9.13E-04	heparanase-like protein 1
Ma06_g00610	ITC1587_Bchr11_P32641	117.88	-0.9	0.29	-3.04	2.38E-03	7.78E-02	transcription factor bHLH144-like
Ma10_g24940	ITC1587_Bchr10_P31090	304.94	-0.9	0.27	-3.35	8.23E-04	4.62E-02	glucose-6-phosphate isomerase 1
Ma03_g19230	ITC1587_Bchr3_P07143	777.56	-0.91	0.29	-3.11	1.85E-03	6.90E-02	calcium/calmodulin-dependent serine/threonine-protein kinase 1-like
Ma03_g00840	ITC1587_Bchr3_P05241	306.24	-0.91	0.31	-2.92	3.46E-03	9.45E-02	lysosomal Pro-X carboxypeptidase
Ma02_g04710	ITC1587_Bchr2_P03342	731.94	-0.92	0.3	-3.05	2.26E-03	7.56E-02	GATA transcription factor 4-like
Ma10_g21360	ITC1587_Bchr10_P30776	120.02	-0.95	0.31	-3.05	2.28E-03	7.59E-02	cell wall protein IFF6-like
Ma05_g23510	ITC1587_Bchr5_P13995	856.21	-0.96	0.25	-3.82	1.34E-04	1.73E-02	ocs element-binding factor 1-like
Ma04_g02590	ITC1587_Bchr4_P08626	2049.5	-0.97	0.3	-3.21	1.34E-03	5.87E-02	probable rhamnose biosynthetic enzyme 1
Ma08_g09100	ITC1587_Bchr8_P22262	1281.31	-0.97	0.33	-2.9	3.72E-03	9.75E-02	zinc finger CCCH domain-containing

								protein 53-like
Ma09_g03040	ITC1587_Bchr9_P25338	172.12	-0.98	0.32	-3.05	2.30E-03	7.62E-02	floral homeotic protein APETALA 2-like
Ma10_g11600	ITC1587_Bchr10_P29948	220.57	-1	0.33	-3.03	2.43E-03	7.88E-02	vacuolar-sorting receptor 3-like
Ma06_g33040	ITC1587_Bchr6_P18028	161.33	-1.01	0.3	-3.4	6.83E-04	4.25E-02	NAC domain-containing protein 21/22
Ma10_g17870	ITC1587_Bchr6_P15793*	539.06	-1.01	0.31	-3.23	1.26E-03	5.65E-02	uncharacterized LOC103968538
Ma03_g14070	ITC1587_BchrUn_random_ P35664	177.95	-1.03	0.34	-3.03	2.46E-03	7.94E-02	cellulose synthase-like protein D2
Ma08_g27330	ITC1587_Bchr8_P24384	112.5	-1.04	0.33	-3.17	1.51E-03	6.20E-02	uncharacterized LOC103996176
Ma09_g30080	ITC1587_Bchr9_P28221*	94.05	-1.04	0.35	-2.96	3.03E-03	8.89E-02	pathogenesis-related protein 5-like
Ma02_g23380	ITC1587_Bchr2_P05012	183.12	-1.06	0.33	-3.22	1.26E-03	5.65E-02	uncharacterized LOC103976765
Ma03_g24550	ITC1587_Bchr3_P07606	1067.67	-1.06	0.31	-3.41	6.52E-04	4.14E-02	auxin response factor 17-like
Ma03_g18060	ITC1587_Bchr2_P03625*	185.33	-1.07	0.36	-2.93	3.38E-03	9.33E-02	transcription factor bHLH113-like
Ma08_g13170	ITC1587_Bchr8_P22720	1287.97	-1.07	0.35	-3.07	2.14E-03	7.33E-02	Probable metal-nicotianamine transporter YSL12
Ma06_g29810	ITC1587_Bchr6_P17728	326.39	-1.09	0.36	-3	2.66E-03	8.31E-02	beta-1
Ma04_g13100	ITC1587_Bchr4_P09570	469.19	-1.12	0.37	-2.98	2.89E-03	8.71E-02	phosphatidylinositol/phosphatidylcholine transfer protein SFH1-like
Ma09_g10900	ITC1587_Bchr9_P26005	400.28	-1.13	0.38	-3.01	2.57E-03	8.12E-02	uncharacterized LOC103997625
Ma05_g03960	ITC1587_Bchr5_P12030*	396.4	-1.15	0.38	-2.99	2.79E-03	8.59E-02	GPI-anchored protein LORELEI-like
Ma08_g23940	ITC1587_Bchr8_P24077	128.23	-1.15	0.39	-2.98	2.88E-03	8.69E-02	phosphatidylinositol 4-phosphate 5-kinase 9-like
Ma09_g17040	ITC1587_Bchr9_P26502	96.33	-1.16	0.37	-3.11	1.87E-03	6.94E-02	no apical meristem protein
Ma09_g11100	ITC1587_Bchr9_P26023	1242.87	-1.18	0.22	-5.46	4.81E-08	1.23E-04	putative ethylene-responsive transcription factor 4
Ma02_g16930	ITC1587_Bchr2_P04433	84.76	-1.18	0.35	-3.39	7.07E-04	4.30E-02	ribonuclease 3-like protein 2
Ma10_g24160	ITC1587_Bchr10_P31023	51.53	-1.19	0.38	-3.1	1.94E-03	7.02E-02	uncharacterized LOC103969110
Ma05_g09560	ITC1587_Bchr5_P12533	642.56	-1.2	0.41	-2.91	3.62E-03	9.65E-02	probable inactive receptor kinase At2g26730
Ma07_g23800	ITC1587_Bchr7_P21000*	603.35	-1.23	0.4	-3.05	2.30E-03	7.62E-02	PRA1 family protein B4-like
Ma02_g17950	ITC1587_Bchr2_P04518	155.89	-1.24	0.39	-3.16	1.60E-03	6.31E-02	myb-related protein MYBAS2
Ma08_g03470	ITC1587_Bchr8_P21762	747.47	-1.24	0.26	-4.76	1.89E-06	1.38E-03	putative ethylene-responsive transcription factor 4
Ma07_g28080	ITC1587_Bchr4_P08691*	1156.47	-1.25	0.29	-4.35	1.37E-05	4.61E-03	UDP-glucose 6-dehydrogenase 4-like

Ma07_g28090	ITC1587_Bchr7_P21373*	458.19	-1.26	0.34	-3.72	2.01E-04	2.22E-02	UDP-glucose 6-dehydrogenase 1-like
Ma10_g21340	ITC1587_Bchr10_P30774	269.8	-1.27	0.38	-3.35	8.16E-04	4.60E-02	NADP-dependent malic enzyme
Ma06_g09840	ITC1587_Bchr6_P15516	78.62	-1.27	0.35	-3.63	2.84E-04	2.75E-02	uncharacterized protein At5g41620-like
Ma06_g08180	ITC1587_Bchr6_P15366	150.82	-1.28	0.41	-3.16	1.60E-03	6.31E-02	U-box domain-containing protein 9-like
Ma07_g14220	ITC1587_Bchr7_P19882	102.1	-1.29	0.42	-3.07	2.11E-03	7.28E-02	WRKY transcription factor 22-like
Ma11_g18680	ITC1587_Bchr11_P33839	278.74	-1.3	0.42	-3.07	2.13E-03	7.32E-02	regulatory protein NPR6-like
Ma05_g17880	ITC1587_Bchr5_P13390*	46.26	-1.31	0.4	-3.29	1.01E-03	5.09E-02	uncharacterized LOC103985228
Ma01_g15280	ITC1587_BchrUn_random_ P37575*	59.2	-1.32	0.41	-3.19	1.40E-03	5.99E-02	uncharacterized LOC103991192
Ma08_g09370	ITC1587_Bchr8_P22281	45.52	-1.32	0.44	-2.98	2.93E-03	8.73E-02	U-box domain-containing protein 4-like
Ma03_g24970	ITC1587_Bchr3_P07643	744.66	-1.32	0.42	-3.16	1.60E-03	6.31E-02	putative ethylene-responsive transcription factor 11
Ma04_g15890	ITC1587_Bchr4_P10027	162.12	-1.34	0.31	-4.31	1.64E-05	5.13E-03	F-box protein At5g39450-like
Ma06_g06710	ITC1587_Bchr9_P28124*	952.69	-1.34	0.41	-3.24	1.19E-03	5.46E-02	UDP-glucuronic acid decarboxylase 6-like
Ma03_g10820	ITC1587_Bchr3_P06160	88.67	-1.34	0.46	-2.9	3.72E-03	9.75E-02	polygalacturonase ADPG2
Ma03_g31250	ITC1587_Bchr3_P08161	178.49	-1.35	0.45	-2.99	2.80E-03	8.61E-02	cysteine-rich and transmembrane domain-containing protein A-like
Ma04_g28130	ITC1587_Bchr4_P10636	220.07	-1.35	0.45	-3.01	2.60E-03	8.15E-02	uncharacterized LOC103983075
Ma07_g18480	ITC1587_BchrUn_random_ P37067	727.77	-1.35	0.42	-3.21	1.33E-03	5.84E-02	uncharacterized LOC103974030
Ma02_g12520	ITC1587_Bchr2_P04053	277.02	-1.36	0.45	-3.02	2.52E-03	8.04E-02	putative GEM-like protein 8
Ma06_g15450	ITC1587_Bchr6_P16022	31.32	-1.37	0.44	-3.07	2.13E-03	7.32E-02	Whole genome shotgun sequence of line PN40024
Ma11_g04210	ITC1587_Bchr11_P32105*	143.86	-1.37	0.46	-2.97	2.96E-03	8.78E-02	S-norcoclaurine synthase 1-like
Ma06_g33550	ITC1587_Bchr6_P18079	289.98	-1.38	0.2	-6.79	1.09E-11	1.39E-07	respiratory burst oxidase homolog protein A-like
Ma10_g25040	ITC1587_Bchr10_P31099	117.22	-1.38	0.35	-3.91	9.19E-05	1.37E-02	probable protein phosphatase 2C 12
Ma08_g20600	ITC1587_Bchr8_P23801	162.08	-1.39	0.45	-3.09	1.98E-03	7.11E-02	uncharacterized LOC103995103
Ma06_g02690	ITC1587_Bchr6_P14865	144.85	-1.4	0.45	-3.08	2.09E-03	7.28E-02	regulatory protein NPR1-like
Ma08_g02110	ITC1587_Bchr8_P21667	560.6	-1.4	0.4	-3.47	5.17E-04	3.66E-02	auxin transporter-like protein 4
Ma08_g32840	ITC1587_Bchr8_P24872	1203.75	-1.4	0.46	-3.07	2.15E-03	7.33E-02	probable methyltransferase PMT24
Ma08_g02670	ITC1587_Bchr9_P27947*	162.31	-1.41	0.4	-3.5	4.66E-04	3.56E-02	wall-associated receptor kinase-like 14

Ma06_g16010	ITC1587_Bchr6_P16081	212.75	-1.41	0.39	-3.6	3.13E-04	2.84E-02	xylem cysteine proteinase 1-like
Ma01_g17310	ITC1587_Bchr1_P02260	127.76	-1.43	0.49	-2.93	3.36E-03	9.31E-02	ABC transporter B family member 4-like
Ma06_g38470	ITC1587_Bchr6_P18502*	891.15	-1.43	0.44	-3.22	1.26E-03	5.65E-02	probable methyltransferase PMT3
Ma03_g24170	ITC1587_BchrUn_random_ P38237*	35.53	-1.43	0.49	-2.94	3.31E-03	9.28E-02	G-type lectin S-receptor-like serine/threonine-protein kinase At4g03230
Ma03_g23850	ITC1587_Bchr3_P07550	1286.91	-1.43	0.46	-3.08	2.04E-03	7.18E-02	zinc finger A20 and AN1 domain-containing stress-associated protein 5-like
Ma03_g27330	ITC1587_Bchr3_P07852	235.53	-1.44	0.39	-3.7	2.13E-04	2.31E-02	probable LRR receptor-like serine/threonine-protein kinase At2g16250
Ma07_g03740	ITC1587_Bchr7_P18878	1485.15	-1.44	0.36	-4.01	6.19E-05	1.11E-02	putative uncharacterized protein
Ma05_g04430	ITC1587_Bchr5_P12077	946.98	-1.45	0.41	-3.49	4.75E-04	3.57E-02	UDP-glucuronate 4-epimerase 1
Ma10_g24690	ITC1587_Bchr10_P31070	157.71	-1.45	0.43	-3.36	7.80E-04	4.45E-02	uncharacterized LOC103969146
Ma06_g27740	ITC1587_Bchr6_P17536	150.25	-1.45	0.47	-3.11	1.85E-03	6.90E-02	probable LRR receptor-like serine/threonine-protein kinase At1g53440
Ma08_g04820	ITC1587_Bchr8_P21876	51.77	-1.45	0.47	-3.07	2.14E-03	7.33E-02	protein PHLOEM PROTEIN 2-LIKE A10-like
Ma09_g01030	ITC1587_Bchr9_P25154	169.86	-1.45	0.35	-4.11	3.89E-05	8.42E-03	serine carboxypeptidase-like 45
Ma06_g20750	ITC1587_Bchr6_P16539	1131.75	-1.46	0.44	-3.29	9.96E-04	5.05E-02	probable CCR4-associated factor 1 homolog 11
Ma11_g23640	ITC1587_Bchr5_P14560*	61.83	-1.48	0.45	-3.25	1.14E-03	5.30E-02	dual specificity protein kinase shkD-like
Ma06_g00820	ITC1587_Bchr11_P32662	225.38	-1.48	0.51	-2.9	3.70E-03	9.73E-02	protein YLS9-like
Ma07_g28030	ITC1587_Bchr7_P21366	514.02	-1.5	0.42	-3.6	3.17E-04	2.85E-02	uncharacterized LOC103992910
Ma10_g10050	ITC1587_Bchr10_P29744	328.64	-1.5	0.46	-3.28	1.03E-03	5.11E-02	probable protein NAP1
Ma08_g08170	ITC1587_Bchr8_P22174	62.9	-1.51	0.47	-3.2	1.38E-03	5.98E-02	RNA recognition motif containing protein
Ma03_g16090	ITC1587_Bchr3_P06760	212.27	-1.51	0.46	-3.26	1.13E-03	5.27E-02	homeobox-leucine zipper protein HOX19
Ma10_g18290	ITC1587_Bchr10_P30520	442.03	-1.52	0.42	-3.62	2.94E-04	2.80E-02	UDP-glucose 6-dehydrogenase 4-like
Ma10_g20300	ITC1587_Bchr10_P30687	157.04	-1.55	0.47	-3.29	9.89E-04	5.05E-02	proline-rich receptor-like protein kinase PERK8
Ma03_g08390	ITC1587_Bchr3_P05933	1125.9	-1.55	0.37	-4.14	3.44E-05	7.86E-03	S-adenosylmethionine synthase
Ma10_g10150	ITC1587_Bchr10_P29751	172.29	-1.56	0.47	-3.29	1.02E-03	5.09E-02	putative AP2 domain containing protein
Ma03_g22750	ITC1587_Bchr3_P07464	112.25	-1.57	0.5	-3.14	1.68E-03	6.55E-02	chloride channel protein CLC-c

Ma05_g28740	ITC1587_Bchr5_P14480	179.07	-1.58	0.43	-3.69	2.26E-04	2.36E-02	calcium-binding protein KIC-like
Ma06_g35670	ITC1587_Bchr6_P18244	52.13	-1.59	0.55	-2.92	3.54E-03	9.56E-02	probable LRR receptor-like serine/threonine-protein kinase At3g47570
Ma01_g14250	ITC1587_Bchr1_P01834	298.21	-1.59	0.45	-3.52	4.35E-04	3.45E-02	uncharacterized LOC103992216
Ma04_g16660	ITC1587_BchrUn_random_P35012*	377.07	-1.6	0.51	-3.13	1.76E-03	6.69E-02	trihelix transcription factor GT-2
Ma06_g20870	ITC1587_Bchr6_P16551	251.59	-1.6	0.32	-4.94	7.91E-07	8.08E-04	soluble inorganic pyrophosphatase-like
Ma10_g10470	ITC1587_Bchr10_P29779	1524.73	-1.61	0.54	-2.99	2.84E-03	8.65E-02	U-box domain-containing protein 27-like
Ma01_g19220	ITC1587_Bchr1_P02321	321.77	-1.61	0.35	-4.53	5.79E-06	2.64E-03	uncharacterized LOC103998092
Ma06_g10800	ITC1587_Bchr6_P15608*	142.54	-1.62	0.4	-4.04	5.30E-05	9.80E-03	LRR receptor-like serine/threonine-protein kinase GSO2
Ma10_g06510	ITC1587_Bchr10_P28573	200.63	-1.62	0.47	-3.47	5.23E-04	3.66E-02	uncharacterized LOC103999709
Ma06_g16200	ITC1587_Bchr6_P16096	151.7	-1.63	0.55	-2.94	3.28E-03	9.25E-02	forkhead box protein G1-like
Ma05_g15470	ITC1587_Bchr5_P13066	223.38	-1.64	0.36	-4.55	5.41E-06	2.51E-03	probable inorganic phosphate transporter 1-8
Ma02_g19100	ITC1587_Bchr2_P04623	47.92	-1.65	0.57	-2.91	3.63E-03	9.68E-02	peroxisomal membrane protein 13-like
Ma03_g29950	ITC1587_BchrUn_random_P38555*	571.81	-1.65	0.46	-3.57	3.60E-04	3.10E-02	cysteine-rich receptor-like protein kinase 10
Ma05_g13570	ITC1587_Bchr5_P12878	66.44	-1.66	0.48	-3.47	5.18E-04	3.66E-02	uncharacterized LOC103984868
Ma02_g05470	ITC1587_Bchr2_P03417	33.77	-1.66	0.56	-2.97	2.96E-03	8.78E-02	putative UPF0496 protein 2
Ma11_g21200	ITC1587_Bchr11_P34074	196.36	-1.66	0.32	-5.24	1.63E-07	3.13E-04	auxin response factor 18-like
Ma10_g07930	ITC1587_Bchr10_P29554	1444.75	-1.66	0.51	-3.26	1.10E-03	5.26E-02	putative Stem-specific protein TSJT1
Ma02_g23190	ITC1587_Bchr2_P04997	124.85	-1.66	0.4	-4.11	4.03E-05	8.55E-03	probable BOI-related E3 ubiquitin-protein ligase 2
Ma07_g26490	ITC1587_BchrUn_random_P39404	71.34	-1.66	0.52	-3.2	1.40E-03	5.99E-02	putative probable LRR receptor-like serine/threonine-protein kinase At1g51810
Ma07_g08520	ITC1587_Bchr7_P19326	55.46	-1.67	0.56	-3	2.74E-03	8.51E-02	3'-N-debenzoyl-2'-deoxytaxol N-benzoyltransferase-like
Ma11_g22280	ITC1587_Bchr11_P34164*	99.06	-1.68	0.47	-3.6	3.14E-04	2.85E-02	zinc finger protein ZAT11-like
Ma08_g01300	ITC1587_Bchr8_P21590	270.65	-1.69	0.48	-3.49	4.81E-04	3.60E-02	Whole genome shotgun sequence of line PN40024
Ma02_g19020	ITC1587_Bchr2_P04615	1159.95	-1.69	0.57	-2.94	3.24E-03	9.18E-02	probable LRR receptor-like serine/threonine-protein kinase

								At2g16250
Ma02_g08720	ITC1587_Bchr2_P03719	81.89	-1.74	0.57	-3.05	2.27E-03	7.58E-02	transcription factor TGA2-like
Ma03_g02030	ITC1587_Bchr3_P05349	691.47	-1.76	0.49	-3.56	3.69E-04	3.14E-02	protein TIFY 10A-like
Ma08_g09890	ITC1587_Bchr8_P22325	820.12	-1.77	0.54	-3.28	1.04E-03	5.11E-02	ingression protein fic1-like
Ma07_g11470	ITC1587_Bchr7_P19614*	101.28	-1.77	0.59	-2.99	2.82E-03	8.63E-02	uncharacterized glycosyl hydrolase Rv2006/MT2062
Ma02_g14570	ITC1587_Bchr2_P04223*	261.17	-1.78	0.49	-3.61	3.07E-04	2.84E-02	GATA transcription factor 12-like
Ma03_g18040	ITC1587_Bchr3_P06978*	875.28	-1.79	0.41	-4.34	1.43E-05	4.73E-03	calmodulin-like protein 3
Ma11_g19050	ITC1587_Bchr11_P33872	757.71	-1.79	0.4	-4.49	7.21E-06	3.07E-03	uncharacterized LOC103971974
Ma00_g03220	ITC1587_BchrUn_random_P38524	112.36	-1.8	0.59	-3.06	2.19E-03	7.41E-02	vacuolar-sorting receptor 6-like
Ma05_g31300	ITC1587_Bchr5_P14710	2218.47	-1.8	0.53	-3.41	6.50E-04	4.14E-02	nematode resistance protein-like HSPRO2
Ma10_g18880	ITC1587_Bchr6_P15717*	127.12	-1.81	0.61	-2.94	3.29E-03	9.26E-02	uncharacterized LOC103968626
Ma10_g28680	ITC1587_Bchr10_P31419	1051.83	-1.81	0.6	-3.02	2.51E-03	8.04E-02	uncharacterized LOC103969480
Ma11_g16170	ITC1587_Bchr11_P31736*	94.95	-1.82	0.63	-2.9	3.70E-03	9.73E-02	Hydrophobic protein LTI6B
Ma11_g01990	ITC1587_Bchr11_P31872	66.42	-1.84	0.56	-3.32	9.10E-04	4.85E-02	RING-H2 finger protein ATL16-like
Ma08_g21480	ITC1587_Bchr8_P23724	26.67	-1.86	0.57	-3.26	1.12E-03	5.27E-02	protein DA1-related 2-like
Ma05_g16120	ITC1587_BchrUn_random_P35166	2196.1	-1.86	0.53	-3.53	4.10E-04	3.31E-02	E3 ubiquitin-protein ligase RHA1B-like
Ma08_g00910	ITC1587_Bchr8_P21554	598.43	-1.86	0.62	-3.02	2.54E-03	8.08E-02	UDP-glucuronate 4-epimerase 6-like
Ma04_g06770	ITC1587_Bchr4_P08977	82.25	-1.87	0.47	-3.97	7.20E-05	1.20E-02	zinc finger protein 6-like
Ma10_g28280	ITC1587_Bchr10_P31382	530.85	-1.87	0.6	-3.14	1.69E-03	6.57E-02	leucine-rich repeat extensin-like protein 3
Ma09_g07320	ITC1587_Bchr9_P25694	2009.67	-1.87	0.61	-3.08	2.04E-03	7.18E-02	notchless protein homolog 1-like
Ma07_g12510	ITC1587_Bchr7_P19721	500.62	-1.88	0.55	-3.41	6.47E-04	4.14E-02	uncharacterized LOC103991516
Ma03_g20430	ITC1587_Bchr3_P07262	52.78	-1.88	0.58	-3.24	1.20E-03	5.49E-02	cytochrome P450 94B3-like
Ma01_g11850	ITC1587_Bchr1_P01635	163.88	-1.89	0.63	-3.01	2.59E-03	8.14E-02	chaperone protein dnaJ 11
Ma10_g10220	ITC1587_Bchr10_P29758	193.77	-1.89	0.39	-4.83	1.39E-06	1.11E-03	uncharacterized LOC104000324
Ma10_g24530	ITC1587_Bchr10_P31055	811.93	-1.9	0.64	-2.95	3.20E-03	9.14E-02	probable pectin methyltransferase QUA2
Ma06_g09240	ITC1587_Bchr6_P15464	644.4	-1.91	0.51	-3.74	1.82E-04	2.14E-02	mitogen-activated protein kinase kinase kinase NPK1-like
Ma11_g22820	ITC1587_Bchr11_P34203	173.94	-1.91	0.55	-3.46	5.50E-04	3.75E-02	protein UPSTREAM OF FLC-like

Ma10_g08130	ITC1587_Bchr10_P29571	245.84	-1.91	0.55	-3.48	5.04E-04	3.63E-02	NBS-LRR disease resistance protein
Ma08_g25890	ITC1587_Bchr8_P24240	408.34	-1.92	0.6	-3.18	1.46E-03	6.11E-02	U-box domain-containing protein 16-like
Ma03_g10750	ITC1587_Bchr3_P06153	100.83	-1.93	0.58	-3.35	8.20E-04	4.61E-02	pirin-like protein
Ma07_g03780	ITC1587_Bchr7_P18881	398.42	-1.93	0.37	-5.19	2.10E-07	3.35E-04	bifunctional 3-dehydroquinate dehydratase/shikimate dehydrogenase
Ma05_g04300	ITC1587_Bchr5_P12061	339.24	-1.93	0.59	-3.27	1.09E-03	5.25E-02	serine carboxypeptidase 24-like
Ma07_g11070	ITC1587_Bchr7_P19576*	96.05	-1.93	0.56	-3.48	4.99E-04	3.63E-02	purple acid phosphatase 17-like
Ma06_g03700	ITC1587_Bchr6_P14954	115	-1.94	0.52	-3.71	2.08E-04	2.26E-02	uncharacterized LOC103986701
Ma07_g19640	ITC1587_Bchr7_P20619	731.05	-1.95	0.55	-3.54	4.02E-04	3.30E-02	trans-cinnamate 4-monooxygenase-like
Ma05_g01920	ITC1587_Bchr5_P11821	105.76	-1.95	0.52	-3.75	1.76E-04	2.09E-02	uncharacterized LOC103983571
Ma08_g21570	ITC1587_Bchr8_P23856	3436.55	-1.95	0.57	-3.42	6.37E-04	4.09E-02	E3 ubiquitin-protein ligase RING1-like
Ma07_g25430	ITC1587_Bchr7_P21152	3269.02	-1.95	0.59	-3.32	8.98E-04	4.82E-02	expressed protein
Ma11_g16450	ITC1587_Bchr11_P33645	529.74	-1.96	0.61	-3.22	1.29E-03	5.72E-02	probable xyloglucan endotransglucosylase/hydrolase protein 30
Ma10_g07640	ITC1587_Bchr10_P29525	87.33	-1.96	0.5	-3.92	8.71E-05	1.34E-02	uncharacterized LOC104000176
Ma02_g11500	ITC1587_Bchr2_P03969	93.52	-1.96	0.64	-3.07	2.11E-03	7.28E-02	uncharacterized LOC103975929
Ma08_g33410	ITC1587_Bchr8_P24918	49.08	-1.97	0.58	-3.42	6.26E-04	4.06E-02	arogenate dehydratase/prephenate dehydratase 6
Ma08_g26630	ITC1587_Bchr3_P06099*	51.72	-1.97	0.51	-3.88	1.02E-04	1.43E-02	nucleobase-ascorbate transporter 6-like
Ma04_g28940	ITC1587_Bchr4_P10715	69.37	-1.97	0.59	-3.34	8.30E-04	4.62E-02	purple acid phosphatase 2-like
Ma11_g22040	ITC1587_Bchr11_P34145	109.36	-1.98	0.66	-3.01	2.63E-03	8.22E-02	uncharacterized LOC103972236
Ma03_g29510	ITC1587_Bchr3_P08026	175.59	-1.99	0.67	-2.98	2.90E-03	8.71E-02	transcription factor MYB44-like
Ma06_g07960	ITC1587_Bchr6_P15344	29.87	-1.99	0.63	-3.16	1.56E-03	6.27E-02	early nodulin-like protein 1
Ma05_g28110	ITC1587_Bchr5_P14424	340.78	-1.99	0.66	-3.04	2.37E-03	7.77E-02	premnaspirodiene oxygenase-like
Ma01_g14190	ITC1587_Bchr1_P01830	60.41	-2	0.61	-3.3	9.73E-04	4.98E-02	glycosyltransferase protein
Ma09_g30970	ITC1587_Bchr9_P28294	42.33	-2	0.66	-3.03	2.42E-03	7.88E-02	uncharacterized LOC103999521
Ma06_g01240	ITC1587_Bchr11_P32698	29.33	-2	0.68	-2.96	3.04E-03	8.89E-02	dof zinc finger protein DOF5.3-like
Ma11_g24260	ITC1587_Bchr11_P34322	131.6	-2.01	0.69	-2.93	3.44E-03	9.43E-02	calcium-binding protein PBP1-like
Ma07_g15780	ITC1587_Bchr7_P20058	294.96	-2.01	0.65	-3.09	1.99E-03	7.13E-02	transcription factor bHLH35
Ma06_g05980	ITC1587_Bchr6_P15180	338.37	-2.01	0.67	-3.02	2.56E-03	8.10E-02	serine/threonine-protein kinase At5g01020-like

Ma06_g13590	ITC1587_Bchr6_P15858*	241.14	-2.01	0.39	-5.16	2.44E-07	3.47E-04	BTB/POZ and TAZ domain-containing protein 2-like
Ma08_g25980	ITC1587_Bchr8_P24250	38.7	-2.06	0.71	-2.92	3.53E-03	9.56E-02	uncharacterized LOC103996295
Ma06_g10780	ITC1587_Bchr6_P15602	48.54	-2.07	0.68	-3.04	2.40E-03	7.84E-02	probable leucine-rich repeat receptor-like protein kinase At1g35710
Ma02_g22840	ITC1587_Bchr2_P04965	53.22	-2.07	0.67	-3.07	2.15E-03	7.33E-02	ankyrin-3-like
Ma05_g26980	ITC1587_Bchr7_P19678*	21.18	-2.08	0.62	-3.37	7.51E-04	4.38E-02	HIPL1 protein-like
Ma03_g26900	ITC1587_Bchr3_P07811	178.82	-2.09	0.65	-3.23	1.22E-03	5.54E-02	BTB/POZ domain-containing protein At5g66560-like
Ma09_g12720	ITC1587_Bchr10_P31604*	644.45	-2.09	0.37	-5.66	1.53E-08	4.88E-05	vegetative cell wall protein gp1-like
Ma06_g14370	ITC1587_Bchr6_P15926	220.57	-2.1	0.52	-4.07	4.75E-05	9.47E-03	1-aminocyclopropane-1-carboxylate oxidase
Ma08_g09910	ITC1587_Bchr8_P22327	417.49	-2.11	0.62	-3.4	6.65E-04	4.19E-02	pollen-specific leucine-rich repeat extensin-like protein 2
Ma01_g07950	ITC1587_Bchr1_P01282	560.83	-2.11	0.64	-3.3	9.54E-04	4.96E-02	glycine dehydrogenase (decarboxylating)
Ma02_g03950	ITC1587_Bchr2_P03279	108.07	-2.12	0.53	-3.96	7.37E-05	1.22E-02	uncharacterized LOC103969571
Ma10_g25820	ITC1587_Bchr10_P31161*	30.3	-2.12	0.73	-2.92	3.51E-03	9.52E-02	transcription initiation factor TFIID subunit 9-like
Ma04_g34100	ITC1587_Bchr4_P11163	468.87	-2.12	0.73	-2.9	3.77E-03	9.83E-02	probable methyltransferase PMT21
Ma09_g01600	ITC1587_Bchr9_P25210	442.72	-2.13	0.63	-3.39	7.08E-04	4.30E-02	adenosylhomocysteinase
Ma04_g23080	ITC1587_Bchr1_P00304	466.96	-2.13	0.7	-3.04	2.36E-03	7.77E-02	uncharacterized LOC103976658
Ma11_g14440	ITC1587_Bchr11_P33439	669.2	-2.13	0.59	-3.6	3.18E-04	2.85E-02	COBRA-like protein 7
Ma10_g11550	ITC1587_Bchr10_P29944	36.38	-2.14	0.74	-2.9	3.69E-03	9.73E-02	uncharacterized LOC104000785
Ma06_g15250	ITC1587_Bchr6_P16004	109.77	-2.15	0.7	-3.08	2.05E-03	7.20E-02	GDSL esterase/lipase At4g10955-like
Ma01_g04690	ITC1587_Bchr1_P00983	703.97	-2.15	0.7	-3.05	2.25E-03	7.56E-02	putative acid phosphatase 1
Ma05_g31450	ITC1587_Bchr5_P14726	159.23	-2.17	0.73	-2.96	3.04E-03	8.90E-02	fasciclin-like arabinogalactan protein 4
Ma05_g06590	ITC1587_Bchr5_P12271	221.63	-2.17	0.54	-3.99	6.64E-05	1.16E-02	probable inactive receptor kinase At4g23740
Ma06_g16210	ITC1587_Bchr6_P16097	71.62	-2.17	0.69	-3.16	1.56E-03	6.27E-02	probable sugar phosphate/phosphate translocator At1g06470
Ma08_g09300	ITC1587_Bchr8_P22276	966.8	-2.17	0.74	-2.93	3.41E-03	9.39E-02	basic 7S globulin-like
Ma01_g11840	ITC1587_Bchr1_P01634	93.29	-2.18	0.7	-3.13	1.75E-03	6.66E-02	uncharacterized LOC103994308
Ma01_g14820		161.43	-2.18	0.66	-3.33	8.75E-04	4.75E-02	lysine-rich arabinogalactan protein 19-like

Ma01_g03590	ITC1587_Bchr1_P00754	38.08	-2.19	0.63	-3.49	4.91E-04	3.62E-02	putative DNA-binding protein ESCAROLA
Ma02_g12530	ITC1587_Bchr1_P00175*	227.29	-2.19	0.65	-3.34	8.26E-04	4.62E-02	myosin-1-like
Ma10_g24580	ITC1587_Bchr10_P31060	478.77	-2.21	0.73	-3.02	2.53E-03	8.05E-02	uncharacterized LOC103969136
Ma10_g04730	ITC1587_Bchr10_P29337	172.99	-2.21	0.73	-3.02	2.54E-03	8.08E-02	kinesin-5-like
Ma01_g14590	ITC1587_Bchr1_P01870	61.76	-2.22	0.49	-4.55	5.39E-06	2.51E-03	uncharacterized LOC103991867
Ma01_g21800	ITC1587_BchrUn_random_P35520	172.07	-2.23	0.66	-3.39	7.00E-04	4.30E-02	AP2/ERF and B3 domain-containing transcription repressor RAV2-like
Ma10_g18610	ITC1587_Bchr10_P30544	2835.14	-2.23	0.7	-3.18	1.47E-03	6.11E-02	sucrose synthase 2-like
Ma04_g07110	ITC1587_Bchr6_P17412*	15.97	-2.25	0.75	-2.98	2.92E-03	8.73E-02	uncharacterized LOC103980826
Ma03_g29050	ITC1587_Bchr3_P08058*	110.36	-2.26	0.52	-4.3	1.68E-05	5.13E-03	probable leucine-rich repeat receptor-like protein kinase At5g63930
Ma01_g19330	ITC1587_Bchr1_P02334	146.55	-2.26	0.45	-4.98	6.34E-07	7.04E-04	Hypothetical protein
Ma07_g19950	ITC1587_Bchr7_P20655	118.21	-2.27	0.6	-3.77	1.62E-04	1.98E-02	putative clathrin assembly protein At1g25240
Ma07_g06000	ITC1587_Bchr7_P19081	1401.85	-2.29	0.65	-3.51	4.56E-04	3.53E-02	putative dehydration-responsive element-binding protein 1D
Ma10_g23400	ITC1587_Bchr10_P30956	813.43	-2.3	0.61	-3.79	1.49E-04	1.86E-02	probable xyloglucan endotransglucosylase/hydrolase protein 25
Ma04_g29240	ITC1587_Bchr4_P10741	756.26	-2.32	0.78	-2.97	2.95E-03	8.78E-02	5-methyltetrahydropteroyltriglutamate--homocysteine methyltransferase 2
Ma07_g14890	ITC1587_Bchr7_P19937	137.23	-2.34	0.78	-3.02	2.52E-03	8.04E-02	protein NRT1/ PTR FAMILY 5.7-like
Ma08_g08030	ITC1587_Bchr8_P22158	502.1	-2.35	0.81	-2.89	3.80E-03	9.86E-02	amino acid permease 3-like
Ma09_g07210	ITC1587_Bchr9_P25689	1174.24	-2.37	0.68	-3.49	4.84E-04	3.60E-02	uncharacterized LOC103997178
Ma03_g17210	ITC1587_Bchr3_P06858	635.73	-2.38	0.7	-3.42	6.21E-04	4.04E-02	probable WRKY transcription factor 41
Ma07_g19790	ITC1587_Bchr7_P20632	1686.46	-2.38	0.6	-3.98	6.93E-05	1.19E-02	probable galacturonosyltransferase-like 6
Ma08_g11830	ITC1587_Bchr8_P22517	156.3	-2.38	0.59	-4.05	5.15E-05	9.80E-03	transcription factor RF2a-like
Ma10_g26710	ITC1587_Bchr10_P31239	21.42	-2.39	0.78	-3.08	2.08E-03	7.28E-02	caffeoylshikimate esterase-like
Ma04_g04550	ITC1587_Bchr4_P08793	17.92	-2.41	0.64	-3.74	1.84E-04	2.15E-02	probable transcription factor KAN2
Ma07_g28960	ITC1587_Bchr7_P21420	35.86	-2.41	0.79	-3.05	2.27E-03	7.58E-02	calcium-binding protein KIC-like
Ma04_g38270	ITC1587_Bchr4_P11514	63.11	-2.41	0.79	-3.06	2.19E-03	7.42E-02	patatin-like protein 3
Ma04_g22340	ITC1587_Bchr1_P00239	75.41	-2.42	0.71	-3.39	6.89E-04	4.26E-02	uncharacterized LOC103977327

Ma06_g30170	ITC1587_Bchr6_P17763	294.69	-2.42	0.55	-4.4	1.08E-05	4.07E-03	lipoxygenase 6
Ma03_g16010	ITC1587_Bchr3_P07819*	33.66	-2.42	0.64	-3.78	1.57E-04	1.92E-02	phytosulfokines 2
Ma04_g13980	ITC1587_Bchr4_P09651	167.87	-2.45	0.78	-3.16	1.58E-03	6.30E-02	polyol transporter 5-like
Ma08_g01770	ITC1587_Bchr8_P21632	48.95	-2.45	0.71	-3.46	5.42E-04	3.71E-02	protein YIPF5 homolog
Ma10_g05760	ITC1587_Bchr10_P28450	631.13	-2.46	0.72	-3.42	6.16E-04	4.03E-02	nematode resistance protein-like HSPRO2
Ma03_g27360	ITC1587_Bchr3_P07857	74.68	-2.47	0.81	-3.05	2.26E-03	7.56E-02	transcription factor bHLH25-like
Ma03_g17130	ITC1587_Bchr3_P06852	288.84	-2.48	0.84	-2.96	3.12E-03	8.99E-02	allene oxide cyclase 3
Ma03_g23500	ITC1587_Bchr3_P07516	22.01	-2.48	0.84	-2.93	3.36E-03	9.31E-02	filament-like plant protein
Ma05_g00400	ITC1587_Bchr5_P11700	257.95	-2.49	0.79	-3.14	1.72E-03	6.62E-02	CASP-like protein MA4_106O17.50
Ma05_g02360	ITC1587_Bchr5_P11861	78.6	-2.5	0.77	-3.25	1.14E-03	5.31E-02	BTB/POZ domain-containing protein At1g30440-like
Ma02_g02550	ITC1587_BchrUn_random_ P34520	21.43	-2.5	0.63	-3.93	8.39E-05	1.31E-02	protein NLP2-like
Ma07_g18620	ITC1587_Bchr7_P20728*	200.81	-2.5	0.84	-2.98	2.90E-03	8.71E-02	alpha-humulene synthase-like
Ma07_g19650	ITC1587_Bchr7_P20620	21.88	-2.51	0.64	-3.92	8.92E-05	1.35E-02	Hypothetical protein
Ma08_g10980	ITC1587_Bchr8_P22436	56.93	-2.52	0.87	-2.91	3.66E-03	9.70E-02	protein NRT1/ PTR FAMILY 6.3-like
Ma05_g04980	ITC1587_Bchr5_P12122	80.01	-2.52	0.79	-3.18	1.50E-03	6.16E-02	nucleobase-ascorbate transporter 6-like
Ma09_g08840	ITC1587_Bchr9_P25822	157.02	-2.53	0.53	-4.79	1.66E-06	1.28E-03	calmodulin-like protein 7
Ma06_g21300	ITC1587_BchrUn_random_ P39709	39.26	-2.54	0.83	-3.05	2.29E-03	7.61E-02	protein G1-like1
Ma06_g16290	ITC1587_Bchr6_P16104	181.3	-2.54	0.64	-3.95	7.92E-05	1.28E-02	putative dehydration-responsive element-binding protein 3
Ma07_g08040	ITC1587_Bchr7_P19282	461.95	-2.55	0.77	-3.33	8.80E-04	4.76E-02	uncharacterized LOC103990325
Ma09_g23420	ITC1587_Bchr9_P27596	477.27	-2.55	0.78	-3.26	1.12E-03	5.26E-02	probable WRKY transcription factor 17
Ma04_g01510	ITC1587_Bchr4_P08523	29.87	-2.55	0.85	-2.99	2.77E-03	8.54E-02	uncharacterized LOC103980338
Ma08_g16980	ITC1587_BchrUn_random_ P37229	181.59	-2.56	0.76	-3.39	7.11E-04	4.31E-02	probable leucine-rich repeat receptor-like protein kinase At5g49770
Ma07_g02670	ITC1587_Bchr7_P18795	116.24	-2.57	0.67	-3.84	1.21E-04	1.63E-02	indole-3-acetic acid-induced protein ARG7-like
Ma10_g07300	ITC1587_Bchr10_P29473	109.95	-2.57	0.88	-2.92	3.55E-03	9.58E-02	Peroxidase 4
Ma07_g06460	ITC1587_Bchr7_P19125	30.8	-2.58	0.88	-2.95	3.16E-03	9.08E-02	glucan endo-1
Ma08_g23400	ITC1587_Bchr8_P24028	192.19	-2.58	0.84	-3.09	2.00E-03	7.14E-02	putative lipoxygenase 5

Ma04_g32260	ITC1587_Bchr4_P11017	92.64	-2.6	0.62	-4.17	3.03E-05	7.23E-03	proline-rich receptor-like protein kinase PERK3
Ma02_g18610	ITC1587_Bchr2_P04578	32.07	-2.61	0.8	-3.26	1.11E-03	5.26E-02	protein NRT1/ PTR FAMILY 4.6-like
Ma10_g27820	ITC1587_Bchr10_P31338	55.6	-2.64	0.77	-3.41	6.45E-04	4.14E-02	Cationic peroxidase 1
Ma03_g30000	ITC1587_Bchr9_P26062*	87.73	-2.66	0.81	-3.27	1.06E-03	5.15E-02	cysteine-rich repeat secretory protein 57-like
Ma02_g17240	ITC1587_Bchr2_P04460	124.71	-2.66	0.86	-3.09	2.01E-03	7.15E-02	SCAR-like protein 2
Ma08_g21890	ITC1587_Bchr8_P23883	17.88	-2.66	0.84	-3.17	1.52E-03	6.20E-02	putative zinc finger and SCAN domain-containing protein 29
Ma08_g01990	ITC1587_Bchr8_P21656	55.16	-2.67	0.68	-3.9	9.62E-05	1.40E-02	probable serine/threonine-protein kinase NAK
Ma04_g26300	ITC1587_Bchr4_P10473	174.28	-2.67	0.84	-3.17	1.51E-03	6.20E-02	Dehydration-responsive element-binding protein 1D
Ma06_g20060	ITC1587_Bchr6_P16459	181.37	-2.68	0.82	-3.26	1.11E-03	5.26E-02	beta-galactosidase 3
Ma10_g27530	ITC1587_Bchr10_P31316*	199.53	-2.68	0.63	-4.23	2.39E-05	6.22E-03	probable inositol oxygenase
Ma06_g04630	ITC1587_Bchr6_P15043	16.98	-2.68	0.87	-3.06	2.18E-03	7.41E-02	ras-related protein RHN1-like
Ma08_g28380	ITC1587_Bchr8_P24478	16.86	-2.68	0.92	-2.9	3.70E-03	9.73E-02	probable WRKY transcription factor 65
Ma10_g05230	ITC1587_Bchr8_P21629*	19.88	-2.69	0.78	-3.47	5.29E-04	3.68E-02	importin subunit beta-1-like
Ma04_g25460	ITC1587_Bchr1_P00542	103.24	-2.69	0.75	-3.61	3.08E-04	2.84E-02	uncharacterized LOC103999827
Ma10_g22540	ITC1587_Bchr10_P30883	405.28	-2.71	0.89	-3.05	2.32E-03	7.66E-02	uncharacterized LOC103968974
Ma04_g32180	ITC1587_Bchr4_P11011	21.08	-2.72	0.83	-3.26	1.11E-03	5.26E-02	3-ketoacyl-CoA synthase 1
Ma01_g01030	ITC1587_Bchr1_P00942	114.55	-2.72	0.9	-3.02	2.52E-03	8.04E-02	probable xyloglucan endotransglucosylase/hydrolase protein 7
Ma06_g23490	ITC1587_Bchr1_P02617	17.69	-2.73	0.73	-3.75	1.75E-04	2.09E-02	putative expressed protein
Ma11_g20870	ITC1587_Bchr11_P34043	17.64	-2.77	0.96	-2.89	3.84E-03	9.94E-02	actin-depolymerizing factor 5-like
Ma04_g36920	ITC1587_Bchr4_P11121*	225.36	-2.79	0.74	-3.78	1.56E-04	1.91E-02	indole-3-acetic acid-induced protein ARG7-like
Ma07_g27150	ITC1587_Bchr7_P21294	643.24	-2.79	0.87	-3.21	1.35E-03	5.90E-02	putative calcium-transporting ATPase 13
Ma08_g21680	ITC1587_Bchr8_P23864	277.64	-2.8	0.79	-3.52	4.31E-04	3.44E-02	UDP-glucuronic acid decarboxylase 6-like
Ma01_g17910	ITC1587_Bchr1_P02200	25.98	-2.8	0.82	-3.44	5.87E-04	3.90E-02	Protein DROOPING LEAF
Ma10_g05210	ITC1587_Bchr10_P28406	40.9	-2.81	0.72	-3.93	8.63E-05	1.34E-02	abscisic acid receptor PYL4-like
Ma04_g35640	ITC1587_Bchr4_P11298	464.33	-2.81	0.91	-3.1	1.96E-03	7.07E-02	1-aminocyclopropane-1-carboxylate synthase

Ma01_g09530	ITC1587_Bchr1_P01418	71.71	-2.83	0.73	-3.89	1.02E-04	1.43E-02	ARMADILLO BTB ARABIDOPSIS PROTEIN 1-like
Ma04_g11990	ITC1587_Bchr4_P09471	212.88	-2.83	0.9	-3.13	1.75E-03	6.66E-02	probable xyloglucan endotransglucosylase/hydrolase protein 32
Ma08_g00620	ITC1587_Bchr8_P21527	87.61	-2.83	0.82	-3.47	5.23E-04	3.66E-02	glycerophosphodiester phosphodiesterase GDE1-like
Ma10_g20040	ITC1587_Bchr10_P30664	27.96	-2.84	0.79	-3.59	3.36E-04	2.95E-02	uncharacterized LOC103968734
Ma09_g12200	ITC1587_Bchr9_P26119*	50.34	-2.85	0.87	-3.27	1.06E-03	5.15E-02	arabinogalactan peptide 23-like
Ma07_g03310	ITC1587_Bchr7_P18846	1895.2	-2.85	0.87	-3.29	1.00E-03	5.05E-02	E3 ubiquitin-protein ligase PUB23-like
Ma05_g24600	ITC1587_Bchr5_P14115	4196.07	-2.86	0.69	-4.12	3.72E-05	8.34E-03	putative nuclease HARBI1
Ma08_g07230	ITC1587_Bchr8_P22080	42.81	-2.87	0.82	-3.48	4.94E-04	3.63E-02	uncharacterized LOC103993598
Ma01_g17140	ITC1587_Bchr1_P02279	56.1	-2.87	0.84	-3.4	6.66E-04	4.19E-02	proline-rich receptor-like protein kinase PERK8
Ma07_g13700	ITC1587_Bchr7_P19833	27.02	-2.88	0.99	-2.89	3.81E-03	9.89E-02	uncharacterized LOC103991611
Ma06_g09230	ITC1587_Bchr6_P15464*	197.78	-2.88	0.59	-4.89	9.88E-07	9.13E-04	mitogen-activated protein kinase kinase kinase NPK1-like
Ma07_g20050	ITC1587_BchrUn_random_P38638	113.15	-2.89	0.93	-3.1	1.91E-03	6.95E-02	uncharacterized protein At1g66480-like
Ma09_g13070	ITC1587_Bchr9_P26191	83.35	-2.92	0.64	-4.57	4.90E-06	2.45E-03	UDP-glucuronate 4-epimerase 1
Ma03_g23970	ITC1587_Bchr3_P07561	19.4	-2.92	0.99	-2.96	3.11E-03	8.99E-02	nucleobase-ascorbate transporter 12-like
Ma05_g05130	ITC1587_Bchr8_P24098*	47.98	-2.94	0.95	-3.1	1.94E-03	7.02E-02	RNA-binding protein 38-like
Ma03_g03460	ITC1587_Bchr3_P05477	17.08	-2.94	0.84	-3.49	4.82E-04	3.60E-02	gibberellic acid methyltransferase 2-like
Ma10_g11400	ITC1587_Bchr10_P29928	21.6	-2.94	0.92	-3.2	1.37E-03	5.96E-02	uncharacterized LOC104000788
Ma06_g10710	ITC1587_Bchr6_P15596	346.32	-2.98	0.67	-4.43	9.27E-06	3.76E-03	uncharacterized LOC103987361
Ma02_g02850	ITC1587_Bchr10_P29855	210.04	-2.98	0.9	-3.33	8.83E-04	4.77E-02	protein YLS9-like
Ma01_g17600	ITC1587_Bchr1_P02234	387.39	-2.98	0.83	-3.61	3.10E-04	2.84E-02	uncharacterized LOC103996876
Ma03_g29900	ITC1587_Bchr3_P08055*	37.11	-3.01	0.92	-3.26	1.10E-03	5.26E-02	putative cysteine-rich receptor-like protein kinase 41
Ma05_g05700	ITC1587_Bchr5_P12192	389.43	-3.01	0.89	-3.37	7.48E-04	4.38E-02	nudix hydrolase 17
Ma04_g05470	ITC1587_Bchr4_P08872	18.19	-3.01	0.86	-3.5	4.63E-04	3.56E-02	25.3 kDa vesicle transport protein
Ma03_g05090	ITC1587_Bchr3_P05620	1421.11	-3.06	0.93	-3.28	1.02E-03	5.11E-02	probable xyloglucan endotransglucosylase/hydrolase protein 23

Ma05_g07100	ITC1587_Bchr5_P12316	187.93	-3.07	1.01	-3.03	2.44E-03	7.92E-02	probable protein phosphatase 2C 32
Ma10_g27380	ITC1587_Bchr10_P31305	134.23	-3.08	0.8	-3.82	1.32E-04	1.72E-02	uncharacterized LOC103969384
Ma10_g13790	ITC1587_BchrUn_random_ P38212*	63.4	-3.09	1.05	-2.95	3.17E-03	9.08E-02	metallothionein-like protein 2C
Ma04_g28060	ITC1587_Bchr4_P10630	102.87	-3.1	0.7	-4.4	1.07E-05	4.07E-03	Hypothetical protein
Ma03_g30740	ITC1587_Bchr3_P08118	53.01	-3.11	1.05	-2.97	2.99E-03	8.83E-02	LOB domain-containing protein 40
Ma10_g14360	ITC1587_Bchr10_P30176	43.79	-3.18	1.07	-2.98	2.88E-03	8.69E-02	peptide-N4-(N-acetyl-beta-glucosaminyl)asparagine amidase A-like
Ma07_g12500	ITC1587_Bchr7_P19720	531.12	-3.2	0.56	-5.73	1.01E-08	3.84E-05	uncharacterized LOC103991515
Ma07_g15950	ITC1587_Bchr7_P20166	287.15	-3.21	1.02	-3.13	1.74E-03	6.66E-02	probable xyloglucan endotransglucosylase/hydrolase protein 23
Ma02_g05360	ITC1587_Bchr2_P03409*	52.87	-3.21	0.75	-4.29	1.79E-05	5.25E-03	chaperone protein dnaJ 11
Ma01_g17450	ITC1587_Bchr1_P02249	29.99	-3.22	1.02	-3.17	1.53E-03	6.23E-02	myb-related protein 305-like
Ma02_g23730	ITC1587_Bchr2_P05041	4072.35	-3.23	0.8	-4.04	5.41E-05	9.94E-03	sucrose synthase 2-like
Ma01_g17700	ITC1587_Bchr1_P02223	65.86	-3.23	0.85	-3.79	1.51E-04	1.88E-02	uncharacterized LOC103996818
Ma09_g26460	ITC1587_Bchr9_P27894	16.29	-3.24	1.08	-3.01	2.62E-03	8.18E-02	protein TIFY 5A-like
Ma05_g06250	ITC1587_Bchr5_P12241	375.44	-3.24	0.9	-3.61	3.12E-04	2.84E-02	uncharacterized LOC103984099
Ma11_g04680	ITC1587_Bchr11_P32138	464.5	-3.24	0.78	-4.16	3.20E-05	7.50E-03	transcription factor MYB108-like
Ma09_g21650	ITC1587_BchrUn_random_ P35241*	15.52	-3.25	0.75	-4.35	1.36E-05	4.61E-03	uncharacterized LOC103973198
Ma04_g01250	ITC1587_Bchr4_P08494	105.32	-3.27	0.99	-3.31	9.19E-04	4.87E-02	serine carboxypeptidase-like 12
Ma02_g19010	ITC1587_Bchr2_P04614	517.18	-3.27	1.03	-3.19	1.43E-03	6.04E-02	Tubulin alpha-1 chain
Ma06_g02570	ITC1587_Bchr6_P14855*	1480.39	-3.28	0.81	-4.04	5.28E-05	9.80E-03	putative nuclease HARBI1
Ma03_g21680	ITC1587_Bchr3_P07364	15.07	-3.28	1.05	-3.11	1.85E-03	6.90E-02	1-deoxy-D-xylulose 5-phosphate reductoisomerase
Ma03_g18560	ITC1587_Bchr3_P07057	100.68	-3.29	1.01	-3.26	1.10E-03	5.26E-02	annexin D4
Ma09_g12240	ITC1587_Bchr9_P26123	235.8	-3.29	0.94	-3.5	4.61E-04	3.55E-02	uncharacterized LOC103997737
Ma01_g21640	ITC1587_Bchr1_P02753	588.76	-3.31	0.73	-4.52	6.32E-06	2.80E-03	probable calcium-binding protein CML31
Ma01_g17590	ITC1587_Bchr1_P02234*	424.49	-3.32	0.7	-4.73	2.28E-06	1.53E-03	uncharacterized LOC103996881
Ma06_g35550	ITC1587_Bchr6_P18231	43.89	-3.32	1.14	-2.92	3.49E-03	9.51E-02	probable anion transporter 2
Ma04_g29120	ITC1587_Bchr4_P10730	40.95	-3.34	0.86	-3.9	9.52E-05	1.40E-02	calmodulin-like

Ma06_g23440	ITC1587_Bchr6_P16951	164.5	-3.34	1	-3.35	8.06E-04	4.57E-02	fasciclin-like arabinogalactan protein 12
Ma06_g33880	ITC1587_Bchr5_P14610*	18.1	-3.37	1.07	-3.16	1.58E-03	6.30E-02	putative uncharacterized protein Sb03g037890
Ma10_g25140	ITC1587_Bchr10_P31112	22	-3.39	1.16	-2.92	3.48E-03	9.47E-02	putative uncharacterized protein Sb04g034290
Ma04_g32550	ITC1587_Bchr2_P04053*	15.35	-3.42	0.9	-3.81	1.37E-04	1.75E-02	putative GEM-like protein 8
Ma05_g05400	ITC1587_Bchr5_P12160	82.84	-3.42	1.08	-3.16	1.55E-03	6.26E-02	missing_product
Ma08_g19170	ITC1587_Bchr8_P23014	13.12	-3.42	0.95	-3.59	3.34E-04	2.94E-02	putative ethylene-responsive transcription factor ERF021
Ma09_g25480	ITC1587_Bchr9_P27807	112.67	-3.44	1.08	-3.2	1.39E-03	5.99E-02	L-ascorbate oxidase homolog
Ma04_g00530	ITC1587_Bchr4_P08428	45.46	-3.45	1.13	-3.06	2.18E-03	7.41E-02	homeobox-leucine zipper protein ATHB-13-like
Ma09_g18960	ITC1587_BchrUn_random_P36066	384.14	-3.48	0.85	-4.08	4.59E-05	9.30E-03	nudix hydrolase 17
Ma06_g14410	ITC1587_BchrUn_random_P39500	72.52	-3.5	0.83	-4.23	2.33E-05	6.22E-03	1-aminocyclopropane-1-carboxylate oxidase-like
Ma10_g31200	ITC1587_Bchr10_P31652	6.33	-3.5	1.06	-3.3	9.69E-04	4.98E-02	F-box/kelch-repeat protein At1g57790-like
Ma05_g00260	ITC1587_Bchr5_P11689	13.13	-3.51	1.05	-3.34	8.36E-04	4.62E-02	uncharacterized LOC103983773
Ma05_g23650	ITC1587_Bchr5_P14009	34.52	-3.52	0.95	-3.72	1.99E-04	2.21E-02	uncharacterized LOC103985606
Ma03_g10710	ITC1587_Bchr3_P06150	284.45	-3.52	1.16	-3.02	2.52E-03	8.04E-02	fasciclin-like arabinogalactan protein 7
Ma03_g05680	ITC1587_Bchr3_P05672	20.63	-3.52	0.94	-3.74	1.81E-04	2.14E-02	pectinesterase-like
Ma09_g13640	ITC1587_Bchr9_P26245	213.22	-3.54	1.11	-3.2	1.38E-03	5.96E-02	leucine-rich repeat extensin-like protein 4
Ma09_g17640	ITC1587_Bchr9_P26625	9.21	-3.56	1.2	-2.97	2.99E-03	8.83E-02	pollen-specific protein SF3-like
Ma06_g16090	ITC1587_Bchr6_P16085*	28.8	-3.56	0.95	-3.74	1.87E-04	2.16E-02	Peroxidase 70
Ma03_g15640	ITC1587_Bchr3_P06620	9.55	-3.6	1.15	-3.13	1.75E-03	6.66E-02	uncharacterized LOC103978282
Ma01_g13890	ITC1587_Bchr1_P01806	30	-3.6	0.67	-5.34	9.48E-08	2.02E-04	dof zinc finger protein DOF1.7-like
Ma07_g20260	ITC1587_Bchr7_P20688	102.56	-3.62	0.89	-4.06	5.01E-05	9.68E-03	lipase
Ma08_g04630	ITC1587_Bchr8_P21858	22.9	-3.63	1.16	-3.12	1.80E-03	6.77E-02	Protein YABBY 2
Ma06_g14420	ITC1587_BchrUn_random_P39500*	34.33	-3.63	1.01	-3.59	3.33E-04	2.94E-02	1-aminocyclopropane-1-carboxylate oxidase-like
Ma08_g23110		33.08	-3.66	1.09	-3.37	7.64E-04	4.42E-02	extensin-1-like
Ma05_g03690	ITC1587_Bchr5_P11998	169.45	-3.67	1.12	-3.28	1.05E-03	5.11E-02	myb-related protein Myb4-like

Ma10_g15930	ITC1587_Bchr10_P30315	61.62	-3.71	1.2	-3.1	1.94E-03	7.02E-02	uncharacterized LOC103968378
Ma05_g13330	ITC1587_Bchr5_P12858	107.87	-3.76	0.91	-4.15	3.35E-05	7.77E-03	GATA transcription factor 4-like
Ma07_g03320	ITC1587_Bchr7_P18846*	35.57	-3.77	0.85	-4.44	9.15E-06	3.76E-03	E3 ubiquitin-protein ligase PUB22
Ma05_g24190	ITC1587_Bchr5_P14071	21.31	-3.77	1.05	-3.59	3.31E-04	2.93E-02	uncharacterized LOC103985562
Ma07_g19970	ITC1587_Bchr7_P20657	304.94	-3.78	1.12	-3.39	7.08E-04	4.30E-02	U-box domain-containing protein 21-like
Ma09_g19260	ITC1587_Bchr1_P00394*	6.46	-3.81	1.25	-3.05	2.31E-03	7.63E-02	Probable aquaporin NIP5-1
Ma06_g34860	ITC1587_Bchr6_P18193	24.47	-3.83	1.11	-3.45	5.70E-04	3.81E-02	zinc finger protein ZAT12-like
Ma03_g12680	ITC1587_Bchr3_P06323	228.12	-3.84	1.04	-3.7	2.19E-04	2.34E-02	RPM1-interacting protein 4-like
Ma07_g11230	ITC1587_Bchr7_P19593	114.27	-3.93	0.83	-4.71	2.46E-06	1.55E-03	ethylene-responsive transcription factor ERF017-like
Ma03_g10560	ITC1587_Bchr9_P28080*	41.27	-3.93	1.29	-3.06	2.23E-03	7.51E-02	uncharacterized LOC103977867
Ma06_g15750	ITC1587_Bchr6_P16056	183.15	-3.94	1.34	-2.95	3.22E-03	9.14E-02	probable polygalacturonase
Ma05_g29880	ITC1587_Bchr5_P14591	80.44	-3.95	1.22	-3.24	1.21E-03	5.50E-02	putative probable glutathione S-transferase
Ma04_g01520	ITC1587_Bchr4_P08524	13.96	-3.96	1.35	-2.93	3.41E-03	9.39E-02	probable protein phosphatase 2C 66
Ma01_g21650	ITC1587_Bchr1_P02753*	86.17	-3.96	0.9	-4.4	1.08E-05	4.07E-03	probable calcium-binding protein CML31
Ma07_g26900	ITC1587_Bchr7_P21276	116.92	-3.98	1.1	-3.6	3.17E-04	2.85E-02	putative OsWRKY11 - Superfamily of TFs having WRKY and zinc finger domains
Ma10_g06250	ITC1587_Bchr11_P34149*	58.74	-3.98	1.37	-2.9	3.76E-03	9.83E-02	protein trichome birefringence-like 33
Ma07_g28510	ITC1587_Bchr7_P21463	11.39	-4.01	1.36	-2.94	3.23E-03	9.16E-02	leucine-rich repeat receptor-like serine/threonine-protein kinase BAM1
Ma03_g11590	ITC1587_Bchr3_P06226	21.79	-4.04	1.02	-3.95	7.89E-05	1.28E-02	protein TRANSPARENT TESTA 1-like
Ma03_g21690	ITC1587_Bchr3_P07365	160.22	-4.05	1.01	-4.01	6.05E-05	1.10E-02	putative Transcription factor bHLH36
Ma03_g09530	ITC1587_Bchr3_P06046	72.65	-4.07	1.29	-3.15	1.62E-03	6.34E-02	receptor-like protein kinase FERONIA
Ma03_g03430	ITC1587_Bchr3_P05474	24.56	-4.07	1.32	-3.08	2.10E-03	7.28E-02	protein YLS9-like
Ma08_g14000	ITC1587_Bchr8_P22643*	62.72	-4.08	0.93	-4.39	1.11E-05	4.11E-03	uncharacterized LOC103994530
Ma09_g02840	ITC1587_Bchr9_P25325	41.92	-4.1	1.33	-3.09	1.99E-03	7.13E-02	receptor-like protein kinase THESEUS 1
Ma10_g22190	ITC1587_Bchr10_P30849	457.78	-4.1	1.39	-2.96	3.07E-03	8.94E-02	Probable aquaporin TIP2-2
Ma08_g32530	ITC1587_Bchr8_P24850	12.42	-4.11	1.42	-2.9	3.77E-03	9.83E-02	protein IQ-DOMAIN 14-like
Ma00_g00150	ITC1587_Bchr3_P06819	60.51	-4.12	0.77	-5.36	8.53E-08	1.98E-04	monothiol glutaredoxin-S9-like
Ma08_g33860	ITC1587_Bchr2_P03229*	36.42	-4.15	1.39	-2.99	2.83E-03	8.65E-02	extensin-like

Ma06_g04440	ITC1587_Bchr6_P15024	156.24	-4.16	1.4	-2.97	2.93E-03	8.74E-02	beta-xylosidase/alpha-L-arabinofuranosidase 2-like
Ma05_g27030	ITC1587_Bchr6_P15206*	8.26	-4.19	1.26	-3.34	8.43E-04	4.65E-02	uncharacterized LOC103986041
Ma10_g15560	ITC1587_Bchr10_P30287	28.7	-4.2	1.42	-2.96	3.11E-03	8.99E-02	AP2/ERF domain-containing transcription factor
Ma06_g03990	ITC1587_Bchr6_P14983	69.27	-4.22	1.21	-3.5	4.73E-04	3.57E-02	Ethylene-responsive transcription factor ERF071
Ma04_g03470	ITC1587_Bchr4_P08697	19.64	-4.23	1.36	-3.1	1.90E-03	6.94E-02	probable indole-3-acetic acid-amido synthetase GH3.8
Ma02_g22650	ITC1587_Bchr2_P04949*	17.81	-4.25	1.14	-3.73	1.91E-04	2.16E-02	cell wall / vacuolar inhibitor of fructosidase 2-like
Ma06_g32030	ITC1587_BchrUn_random_P37212*	119.22	-4.26	1.12	-3.81	1.39E-04	1.75E-02	uncharacterized glycosyl hydrolase Rv2006/MT2062
Ma05_g29230	ITC1587_Bchr5_P14531*	30.77	-4.27	1.41	-3.04	2.39E-03	7.81E-02	putative AP2/ERF domain-containing transcription factor
Ma10_g12070	ITC1587_Bchr10_P29985	102.72	-4.28	1.39	-3.08	2.04E-03	7.19E-02	uncharacterized LOC104000673
Ma06_g07600	ITC1587_Bchr6_P15313	16.58	-4.3	1.31	-3.29	9.98E-04	5.05E-02	65-kDa microtubule-associated protein 7-like
Ma04_g22570	ITC1587_Bchr1_P00256	47.38	-4.3	1.48	-2.91	3.59E-03	9.61E-02	uncharacterized LOC103977141
Ma03_g19660		76.81	-4.31	1.31	-3.3	9.64E-04	4.98E-02	lysine-rich arabinogalactan protein 18-like
Ma07_g20310	ITC1587_Bchr7_P20692	229.11	-4.32	0.92	-4.68	2.90E-06	1.66E-03	AP2 domain containing protein
Ma03_g06360	ITC1587_Bchr3_P05737	146.42	-4.4	1.08	-4.09	4.35E-05	9.00E-03	uncharacterized LOC103977492
Ma11_g17220	ITC1587_Bchr11_P33711	18.77	-4.41	1.33	-3.3	9.54E-04	4.96E-02	uncharacterized LOC103971817
Ma10_g26320	ITC1587_Bchr10_P31203	146.83	-4.47	1.11	-4.01	6.18E-05	1.11E-02	uncharacterized LOC103969282
Ma11_g17680	ITC1587_Bchr11_P33750	87.35	-4.47	1.45	-3.08	2.09E-03	7.28E-02	fructokinase-1-like
Ma11_g19990	ITC1587_Bchr11_P33961	81.78	-4.47	1.09	-4.1	4.05E-05	8.55E-03	Dehydration-responsive element-binding protein 1E
Ma03_g06600	ITC1587_Bchr3_P05755*	55.82	-4.48	1.29	-3.48	5.04E-04	3.63E-02	uncharacterized LOC103977510
Ma06_g06480	ITC1587_Bchr6_P15218	1322.84	-4.52	1.29	-3.51	4.55E-04	3.53E-02	putative nuclease HARBI1
Ma05_g21280	ITC1587_Bchr5_P13755	12.88	-4.53	1.38	-3.27	1.07E-03	5.17E-02	actin-depolymerizing factor 5-like
Ma06_g23720	ITC1587_Bchr6_P16915	45.08	-4.57	1.3	-3.52	4.34E-04	3.45E-02	gibberellin 2-beta-dioxygenase 8
Ma09_g26920	ITC1587_Bchr9_P27938	151.99	-4.58	1.26	-3.64	2.69E-04	2.63E-02	probable galacturonosyltransferase-like 1
Ma02_g17280	ITC1587_Bchr2_P04464	71.47	-4.58	1.39	-3.29	9.97E-04	5.05E-02	Peroxidase 52

Ma07_g08160	ITC1587_Bchr7_P19295	17.78	-4.59	1.58	-2.9	3.73E-03	9.76E-02	uncharacterized LOC103990315
Ma02_g08860	ITC1587_Bchr2_P03734	14.24	-4.6	1.46	-3.16	1.58E-03	6.30E-02	uncharacterized LOC103976145
Ma03_g07220	ITC1587_Bchr3_P05812	19.43	-4.6	1.37	-3.37	7.53E-04	4.38E-02	thaumatin-like protein
Ma03_g29290	ITC1587_Bchr3_P08006	50.19	-4.61	1.55	-2.98	2.87E-03	8.69E-02	probable xyloglucan glycosyltransferase 5
Ma10_g30050	ITC1587_Bchr10_P31546	173.81	-4.62	1	-4.63	3.68E-06	2.00E-03	Ethylene-responsive transcription factor ERF071
Ma09_g21660	ITC1587_BchrUn_random_P35241	13.67	-4.62	1.11	-4.18	2.93E-05	7.06E-03	uncharacterized LOC103973196
Ma03_g14690	ITC1587_Bchr3_P06593	1987.98	-4.63	1.28	-3.62	2.94E-04	2.80E-02	catalase isozyme A-like
Ma09_g27440	ITC1587_Bchr9_P27976	48.08	-4.66	1.55	-3.01	2.58E-03	8.12E-02	uncharacterized LOC103999144
Ma04_g21110	ITC1587_Bchr1_P00056	67.55	-4.73	1.6	-2.95	3.18E-03	9.10E-02	phospholipase A1-Ibeta2
Ma10_g04340	ITC1587_Bchr10_P29296	15.24	-4.74	1.61	-2.94	3.27E-03	9.24E-02	putative UDP-glucuronate:xylan alpha-glucuronosyltransferase 3
Ma06_g14620	ITC1587_Bchr6_P15942	332.9	-4.74	0.92	-5.16	2.43E-07	3.47E-04	UPF0496 protein 4-like
Ma09_g13490	ITC1587_Bchr9_P26231	20.64	-4.82	1.36	-3.55	3.82E-04	3.20E-02	uncharacterized LOC103997893
Ma04_g21130	ITC1587_Bchr1_P00054	14.94	-4.84	1.62	-3	2.74E-03	8.51E-02	cyclin-P4-1-like
Ma08_g11260	ITC1587_Bchr8_P22467	18.04	-4.89	1.38	-3.53	4.10E-04	3.31E-02	uncharacterized LOC103993249
Ma09_g13700	ITC1587_Bchr9_P26249	266.3	-4.94	1.67	-2.97	3.02E-03	8.88E-02	probable pectinesterase/pectinesterase inhibitor 16
Ma03_g06210	ITC1587_Bchr3_P05720	19.08	-5	1.68	-2.98	2.92E-03	8.73E-02	protein NSP-INTERACTING KINASE 1-like
Ma03_g13420	ITC1587_BchrUn_random_P35146*	33.95	-5	1.09	-4.59	4.43E-06	2.26E-03	plant intracellular Ras-group-related LRR protein 1-like
Ma02_g02510	ITC1587_BchrUn_random_P34524	23.78	-5.01	1.54	-3.26	1.13E-03	5.27E-02	putative ferric-chelate reductase 1
Ma04_g09430	ITC1587_Bchr4_P09224	12	-5.02	1.73	-2.9	3.67E-03	9.73E-02	transcription factor MYB44-like
Ma11_g02980	ITC1587_Bchr11_P31969	346.45	-5.06	1.73	-2.93	3.37E-03	9.33E-02	fasciclin-like arabinogalactan protein 8
Ma11_g12510	ITC1587_Bchr11_P33216	669.66	-5.1	1.6	-3.18	1.45E-03	6.10E-02	probable beta-D-xylosidase 2
Ma10_g19040	ITC1587_Bchr10_P30579	41.78	-5.14	1.57	-3.27	1.07E-03	5.17E-02	putative trehalose-phosphate phosphatase
Ma09_g25750	ITC1587_Bchr9_P27833	8.99	-5.19	1.6	-3.25	1.16E-03	5.38E-02	probable isoaspartyl peptidase/L-asparaginase 2
Ma11_g05570	ITC1587_Bchr11_P32217	11.49	-5.19	1.63	-3.18	1.47E-03	6.11E-02	classical arabinogalactan protein 26-like
Ma05_g21260	ITC1587_Bchr5_P13753	83.8	-5.2	1.44	-3.62	3.00E-04	2.82E-02	butyrate--CoA ligase AAE11

Ma10_g15000	ITC1587_Bchr6_P16022*	6.9	-5.22	1.58	-3.3	9.54E-04	4.96E-02	uncharacterized LOC104000795
Ma06_g02740	ITC1587_Bchr6_P14869	10.17	-5.28	1.25	-4.23	2.35E-05	6.22E-03	probable inositol oxygenase
Ma03_g30090	ITC1587_Bchr3_P08065	140.15	-5.31	0.93	-5.72	1.05E-08	3.84E-05	mitogen-activated protein kinase kinase kinase A-like
Ma08_g11720	ITC1587_Bchr8_P22508	41.54	-5.32	1.34	-3.96	7.38E-05	1.22E-02	dehydration-responsive element-binding protein 1G
Ma09_g01250	ITC1587_Bchr9_P25174	68.71	-5.33	1.71	-3.11	1.85E-03	6.90E-02	neurofilament heavy polypeptide
Ma10_g14940	ITC1587_Bchr2_P04868*	13.94	-5.39	1.56	-3.46	5.33E-04	3.69E-02	dynein light chain LC6
Ma03_g07410	ITC1587_Bchr3_P05828	12.65	-5.39	1.54	-3.51	4.56E-04	3.53E-02	nudix hydrolase 17
Ma09_g01630	ITC1587_Bchr9_P25213	20.76	-5.4	1.84	-2.93	3.34E-03	9.31E-02	protein RALF-like 33
Ma07_g08690	ITC1587_Bchr7_P19375	12.68	-5.49	1.48	-3.72	2.02E-04	2.23E-02	probable WRKY transcription factor 72
Ma04_g15370	ITC1587_Bchr4_P09793	10.64	-5.52	1.56	-3.54	4.02E-04	3.30E-02	zinc finger CCCH domain-containing protein 2-like
Ma04_g06600	ITC1587_Bchr4_P08963	33.83	-5.58	1.91	-2.91	3.56E-03	9.58E-02	calmodulin-binding protein
Ma01_g14600	ITC1587_Bchr1_P01871	110.88	-5.67	1.21	-4.68	2.92E-06	1.66E-03	AP2/ERF and B3 domain-containing protein Os05g0549800-like
Ma03_g30570	ITC1587_Bchr3_P08106	8.74	-5.76	1.93	-2.99	2.80E-03	8.61E-02	AP2/ERF and B3 domain-containing protein Os05g0549800-like
Ma05_g16710	ITC1587_BchrUn_random_P36267	15.88	-5.77	1.93	-2.99	2.81E-03	8.63E-02	glucan endo-1
Ma10_g27780	ITC1587_Bchr7_P19431*	123.7	-5.92	1.91	-3.11	1.90E-03	6.94E-02	protodermal factor 1-like
Ma09_g06370	ITC1587_Bchr6_P17615*	26.91	-5.94	1.97	-3.01	2.57E-03	8.12E-02	transcription factor RF2b-like
Ma03_g16350	ITC1587_BchrUn_random_P34776	7.68	-5.95	2.01	-2.96	3.09E-03	8.97E-02	leucine-rich repeat extensin-like protein 3
Ma04_g14470	ITC1587_Bchr4_P09706	7.64	-5.97	1.9	-3.14	1.72E-03	6.62E-02	flavonol sulfotransferase-like
Ma03_g05830	ITC1587_Bchr3_P05688	18.18	-6.02	1.77	-3.39	6.88E-04	4.26E-02	ethylene-responsive transcription factor ERF016-like
Ma03_g17260	ITC1587_Bchr3_P07769*	38.12	-6.03	1.76	-3.42	6.33E-04	4.08E-02	splicing factor 3A subunit 2-like
Ma02_g23830	ITC1587_Bchr2_P05049	40.46	-6.05	1.71	-3.54	4.07E-04	3.31E-02	expansin-A10-like
Ma05_g18620	ITC1587_Bchr5_P13351	12.8	-6.06	2.01	-3.01	2.58E-03	8.12E-02	putative cytochrome P450 94A1
Ma06_g04640	ITC1587_Bchr6_P15045	89.43	-6.24	1.23	-5.07	3.88E-07	4.50E-04	36.4 kDa proline-rich protein-like
Ma04_g33260	ITC1587_Bchr4_P11092	17.08	-6.26	1.9	-3.29	9.96E-04	5.05E-02	probable pectate lyase 8
Ma11_g04170	ITC1587_Bchr11_P32098	45.73	-6.36	1.74	-3.66	2.56E-04	2.54E-02	GATA transcription factor 4-like

Ma04_g34330	ITC1587_Bchr4_P11181	11.09	-6.48	2.23	-2.9	3.69E-03	9.73E-02	glucan endo-1
Ma09_g11440	ITC1587_Bchr9_P26051	299.4	-6.52	1.81	-3.59	3.26E-04	2.91E-02	isocitrate lyase
Ma06_g34460	ITC1587_Bchr6_P18155*	21.01	-6.54	1.95	-3.35	8.16E-04	4.60E-02	cytosolic endo-beta-N-acetylglucosaminidase-like
Ma08_g34320	ITC1587_Bchr8_P25009	325	-6.58	2.04	-3.23	1.24E-03	5.59E-02	Aquaporin PIP-type
Ma03_g28380	ITC1587_Bchr3_P07948	6.71	-6.6	2.13	-3.09	1.99E-03	7.13E-02	cytokinin hydroxylase-like
Ma05_g13640	ITC1587_Bchr5_P12886	9.21	-6.76	1.6	-4.23	2.38E-05	6.22E-03	IQ calmodulin-binding motif family protein
Ma03_g33490	ITC1587_Bchr3_P08352*	10.57	-6.76	2	-3.38	7.27E-04	4.35E-02	uncharacterized LOC103980029
Ma06_g02610	ITC1587_Bchr6_P14858	112.62	-6.79	2.11	-3.21	1.32E-03	5.82E-02	beta-galactosidase-like
Ma02_g17270	ITC1587_Bchr2_P04463	38.2	-6.95	1.74	-4	6.46E-05	1.15E-02	Peroxidase 52
Ma02_g17570	ITC1587_Bchr2_P04487	10.06	-6.95	2.25	-3.09	2.02E-03	7.17E-02	uncharacterized LOC103975432
Ma01_g18460	ITC1587_Bchr1_P02150	79.43	-7.03	2.08	-3.38	7.35E-04	4.37E-02	WEB family protein At5g16730
Ma09_g20470	ITC1587_Bchr9_P27291	27.21	-7.47	1.76	-4.24	2.23E-05	6.09E-03	probable xyloglucan endotransglucosylase/hydrolase protein 32
Ma02_g10570	ITC1587_Bchr2_P03886*	8.84	-7.49	2.34	-3.19	1.41E-03	5.99E-02	L-gulonolactone oxidase-like
Ma01_g03800	ITC1587_Bchr1_P00772	63.22	-7.59	2.5	-3.03	2.45E-03	7.92E-02	probable xyloglucan endotransglucosylase/hydrolase protein 8
Ma05_g26050	ITC1587_Bchr5_P14241	39.76	-7.77	2.06	-3.77	1.64E-04	1.98E-02	transcription factor BEE 1-like
Ma08_g20500	ITC1587_Bchr8_P23819	15.13	-7.79	2.47	-3.16	1.60E-03	6.31E-02	leucine-rich repeat extensin-like protein 6
Ma01_g11980	ITC1587_Bchr1_P01650	46.28	-7.86	2	-3.93	8.34E-05	1.31E-02	uncharacterized LOC103994162
Ma11_g13300	ITC1587_Bchr11_P33322	8.72	-7.88	2.7	-2.92	3.46E-03	9.45E-02	early nodulin-like protein 3
Ma06_g25310	ITC1587_Bchr6_P17305	138.35	-7.94	2.39	-3.32	8.90E-04	4.79E-02	uncharacterized LOC103988981
Ma09_g22130	ITC1587_Bchr9_P27465*	29.44	-7.99	2.47	-3.24	1.20E-03	5.48E-02	protein YLS3-like
Ma09_g16290	ITC1587_Bchr9_P27181*	83.73	-8.01	1.71	-4.7	2.66E-06	1.58E-03	alpha-dioxygenase 1-like
Ma11_g03390	ITC1587_Bchr11_P32009	7.02	-8.24	2.82	-2.92	3.50E-03	9.51E-02	probable receptor-like protein kinase At4g10390
Ma03_g12690	ITC1587_Bchr3_P06324	13.3	-8.43	2.36	-3.58	3.49E-04	3.03E-02	GDSL esterase/lipase At4g26790-like
Ma03_g21640	ITC1587_Bchr3_P07361	154.82	-8.99	2.42	-3.72	1.97E-04	2.19E-02	Peroxidase 72
Ma01_g13710	ITC1587_Bchr1_P01790	92.46	-9.73	2.86	-3.4	6.82E-04	4.25E-02	protein ECERIFERUM 1-like
Ma08_g00630	ITC1587_Bchr8_P21529	11.16	-25.5	5.98	-4.26	2.01E-05	5.70E-03	bidirectional sugar transporter SWEET14-like

Table S6: Complete list of genes differentially expressed due to genotypes interaction with *Xanthomonas campestris* pv. *musacearum* at 48 hpi.

Gene ID from *Musa acuminata* (DH Pahang)	Gene ID from *Musa balbisiana* (Pisang Klutuk Wulung)	BaseMean	Log2 Fold Change	lfcSE	Stat	Pvalue	Padj	Description
Ma02_g09860	ITC1587_Bchr2_P03815	122.44	7.85	2.22	3.53	4.11E-04	0.07	uncharacterized protein C24B11.05-like
Ma01_g13710	ITC1587_Bchr1_P01790	12.23	7.07	1.93	3.67	2.47E-04	0.053	protein ECERIFERUM 1-like
Ma03_g16020	ITC1587_Bchr3_P06748	6.03	6.85	1.84	3.71	2.04E-04	0.05	uncharacterized LOC103978341
Ma08_g16700	ITC1587_Bchr6_P15616*	19.05	6.44	1.89	3.41	6.51E-04	0.086	protein MOTHER of FT and TF 1-like
Ma08_g04050	ITC1587_Bchr8_P21808	42.02	6.05	1.32	4.58	4.74E-06	0.006	putative probable 3-ketoacyl-CoA synthase 2
Ma02_g22610	ITC1587_Bchr2_P04944	3.77	5.75	1.71	3.37	7.51E-04	0.09	amino acid permease 4-like
Ma07_g15500	ITC1587_Bchr7_P20008	32.89	5.67	1.6	3.54	3.99E-04	0.069	putative cell wall protein
Ma07_g12820	ITC1587_Bchr10_P31102	15.14	5.67	1.39	4.07	4.62E-05	0.027	EF hand family protein
Ma07_g22430		9.6	5.53	1.49	3.71	2.08E-04	0.05	Hypothetical protein
Ma03_g14260	ITC1587_BchrUn_random_P35644	27.47	5.52	1.48	3.73	1.92E-04	0.048	BAHD acyltransferase DCR
Ma04_g37530	ITC1587_Bchr4_P11456	36.07	5.45	1.25	4.36	1.31E-05	0.012	heat stress transcription factor C-2a-like
Ma02_g09430	ITC1587_Bchr2_P03779	10.4	5.29	1.48	3.57	3.62E-04	0.064	low affinity sulfate transporter 3-like
Ma04_g02930	ITC1587_Bchr4_P08659	93.82	4.93	0.96	5.11	3.25E-07	0.002	glucose-1-phosphate adenylyltransferase large subunit 1-like
Ma06_g25850	ITC1587_Bchr6_P17218	19.99	4.65	1.17	3.98	6.81E-05	0.029	3-ketoacyl-CoA synthase 11
Ma11_g12530	ITC1587_Bchr11_P33218	7.62	4.65	1.34	3.48	5.02E-04	0.076	stachyose synthase
Ma10_g30110	ITC1587_Bchr6_P15772*	5.86	4.63	1.37	3.39	7.02E-04	0.089	peamaclein-like
Ma05_g22570	ITC1587_Bchr5_P13888	12.64	4.52	1.12	4.04	5.45E-05	0.028	subtilisin-like protease
Ma05_g03150	ITC1587_Bchr5_P11943	10.47	4.5	1.35	3.34	8.33E-04	0.094	protein HOTHEAD-like
Ma07_g08490	ITC1587_Bchr7_P19324	20.87	4.34	1.29	3.37	7.55E-04	0.09	pleiotropic drug resistance protein 6
Ma05_g29440	ITC1587_Bchr11_P31698*	53.86	4.32	1.08	3.99	6.51E-05	0.029	uncharacterized LOC103986283
Ma01_g14490	ITC1587_Bchr1_P01860	755.31	4.3	0.87	4.96	7.14E-07	0.003	18.5 kDa class I heat shock protein
Ma05_g20490	ITC1587_Bchr5_P13663	12.52	4.16	1.16	3.59	3.32E-04	0.063	putative invertase inhibitor

Ma02_g24240	ITC1587_Bchr2_P05084	10.11	4.13	1.07	3.86	1.13E-04	0.035	UDP-glucuronate:xylan alpha-glucuronosyltransferase 2-like
Ma05_g11190	ITC1587_Bchr5_P12674	20.85	4.01	0.82	4.91	9.24E-07	0.003	subtilisin-like protease
Ma10_g29680	ITC1587_Bchr10_P31511*	54.21	3.66	1.06	3.46	5.30E-04	0.076	ATP-dependent zinc metalloprotease FTSH 6
Ma08_g07110	ITC1587_Bchr8_P22071	29.29	3.63	0.76	4.76	1.90E-06	0.004	cytochrome P450 77A3-like
Ma03_g31600	ITC1587_Bchr3_P08196	26.38	3.59	1.06	3.38	7.26E-04	0.09	ninja-family protein 6-like
Ma02_g13100	ITC1587_Bchr2_P04098	30.46	3.58	1.02	3.53	4.17E-04	0.07	subtilisin-like protease
Ma10_g27880	ITC1587_Bchr10_P31344	44.46	3.47	0.74	4.67	2.98E-06	0.004	zinc finger CCCH domain-containing protein 2-like
Ma01_g18330	ITC1587_Bchr1_P02164	256.26	3.41	0.66	5.16	2.49E-07	0.002	trihelix transcription factor GTL1-like
Ma03_g04050	ITC1587_Bchr3_P05519	74.01	3.39	0.91	3.71	2.04E-04	0.05	BTB/POZ domain-containing protein At1g55760-like
Ma07_g00050	ITC1587_Bchr7_P18552	9.91	3.35	0.91	3.67	2.46E-04	0.053	uncharacterized LOC103991041
Ma09_g28620	ITC1587_Bchr9_P28084	22.86	3.21	0.96	3.36	7.79E-04	0.091	RING-H2 finger protein ATL43-like
Ma05_g20500	ITC1587_Bchr5_P13664	16.86	3.2	0.9	3.57	3.56E-04	0.064	pectinesterase inhibitor-like
Ma03_g24790	ITC1587_Bchr3_P07628	29.15	3.16	0.93	3.38	7.19E-04	0.09	putative E3 ubiquitin-protein ligase LIN-1 {ECO:0000250|UniProtKB:D1FP53}
Ma04_g36440	ITC1587_Bchr4_P11370	22.4	3.16	0.84	3.76	1.69E-04	0.045	cytosolic sulfotransferase 6-like
Ma07_g25910	ITC1587_Bchr7_P21196	12.13	3.1	0.93	3.32	8.97E-04	0.097	TPR repeat-containing thioredoxin TTL1-like
Ma11_g16060	ITC1587_Bchr11_P33612	38.57	3.06	0.63	4.88	1.07E-06	0.003	transcription factor EGL1-like
Ma01_g10360	ITC1587_Bchr1_P01491	23.09	2.93	0.74	3.94	8.29E-05	0.03	putative lipid-transfer protein DIR1
Ma04_g27500	ITC1587_Bchr7_P18722*	21.87	2.78	0.81	3.44	5.73E-04	0.079	uncharacterized LOC103983137
Ma06_g26940	ITC1587_Bchr6_P17458	17.71	2.74	0.81	3.40	6.75E-04	0.088	probable inactive receptor kinase At5g58300
Ma03_g25130	ITC1587_Bchr3_P07656	25.2	2.74	0.7	3.9	9.46E-05	0.032	CASP-like protein 5
Ma05_g01050	ITC1587_Bchr5_P11745	20.37	2.72	0.79	3.44	5.84E-04	0.079	uncharacterized LOC103983649
Ma08_g11730	ITC1587_Bchr8_P22509	18.24	2.67	0.78	3.41	6.44E-04	0.085	post-GPI attachment to proteins factor 3-like
Ma10_g17570	ITC1587_Bchr10_P30463	30.05	2.66	0.7	3.8	1.42E-04	0.04	subtilisin-like protease
Ma02_g10140	ITC1587_Bchr2_P03841*	61.33	2.66	0.59	4.48	7.32E-06	0.008	zinc finger Ran-binding domain-containing protein 2-like
Ma06_g15030	ITC1587_Bchr6_P15980	172.49	2.65	0.45	5.85	4.89E-09	0	allene oxide synthase
Ma09_g10290	ITC1587_Bchr9_P25949	59.14	2.52	0.61	4.15	3.33E-05	0.022	uncharacterized LOC103997570
Ma11_g15640	ITC1587_Bchr11_P33565	51.73	2.52	0.62	4.1	4.17E-05	0.025	ras-related protein RABC2a-like

Ma05_g02500	ITC1587_Bchr5_P11875	51.82	2.49	0.71	3.51	4.47E-04	0.071	9-cis-epoxycarotenoid dioxygenase 1
Ma03_g16030	ITC1587_Bchr3_P06749	66.33	2.47	0.71	3.48	5.10E-04	0.076	growth-regulating factor 6
Ma11_g24920	ITC1587_Bchr11_P34375	233.47	2.44	0.56	4.38	1.18E-05	0.012	premnaspirodiene oxygenase-like
Ma04_g23650	ITC1587_Bchr1_P00366	15.22	2.44	0.71	3.41	6.40E-04	0.085	uncharacterized LOC103976091
Ma05_g01810	ITC1587_Bchr5_P11814*	88.79	2.39	0.65	3.66	2.56E-04	0.054	magnesium transporter MRS2-3
Ma08_g09130	ITC1587_Bchr8_P22265	20.43	2.38	0.66	3.62	2.91E-04	0.059	protein SCARECROW 2-like
Ma03_g18730	ITC1587_BchrUn_random_ P38210	86.93	2.37	0.57	4.13	3.71E-05	0.024	probable protein phosphatase 2C 68
Ma09_g11790	ITC1587_Bchr9_P26085	20.17	2.37	0.65	3.64	2.75E-04	0.057	wound induced protein
Ma05_g12170	ITC1587_Bchr1_P00039*	579.01	2.3	0.61	3.79	1.51E-04	0.041	Probable aquaporin PIP1-2
Ma10_g27770	ITC1587_Bchr10_P31333	215.87	2.29	0.57	4.03	5.47E-05	0.028	heat stress transcription factor C-2b-like
Ma07_g21520	ITC1587_Bchr1_P00880*	27.36	2.23	0.61	3.67	2.40E-04	0.052	potassium transporter 6-like
Ma01_g20890	ITC1587_Bchr1_P02200*	34.6	2.21	0.64	3.45	5.59E-04	0.078	YABBY transcription factor
Ma09_g09340	ITC1587_Bchr9_P25868	20.12	2.21	0.63	3.5	4.72E-04	0.073	RING-H2 finger protein ATL65
Ma05_g05020	ITC1587_Bchr5_P12125	85.73	2.2	0.56	3.97	7.28E-05	0.029	homeobox-leucine zipper protein HOX16-like
Ma07_g25610	ITC1587_Bchr7_P21170	39.37	2.19	0.59	3.71	2.05E-04	0.05	zinc finger protein MAGPIE-like
Ma08_g23410	ITC1587_Bchr8_P24029	40.13	2.19	0.59	3.68	2.30E-04	0.052	protein Brevis radix-like 1
Ma08_g33470	ITC1587_Bchr8_P24925	32.65	2.13	0.63	3.37	7.39E-04	0.09	G-type lectin S-receptor-like serine/threonine-protein kinase At2g19130
Ma10_g13960	ITC1587_Bchr10_P30136	101.82	2.11	0.45	4.72	2.34E-06	0.004	glycerol-3-phosphate 2-O-acyltransferase 6-like
Ma10_g00390	ITC1587_BchrUn_random_ P35062	16.55	2.11	0.63	3.36	7.72E-04	0.091	zinc finger CCCH domain-containing protein 35-like
Ma06_g01860	ITC1587_Bchr6_P14797*	166.88	2.11	0.57	3.7	2.12E-04	0.05	uncharacterized LOC103986549
Ma03_g19080	ITC1587_Bchr3_P07100	27.18	2.09	0.53	3.93	8.58E-05	0.031	Superoxide dismutase [Mn] 3.1
Ma07_g19350	ITC1587_Bchr7_P20593	85.31	2	0.58	3.43	6.12E-04	0.082	Myb-related protein 306
Ma05_g10880	ITC1587_Bchr5_P12649	98.93	1.88	0.49	3.88	1.05E-04	0.034	uncharacterized LOC103984583
Ma06_g16710	ITC1587_Bchr6_P16136	136.44	1.85	0.56	3.34	8.52E-04	0.095	probable protein phosphatase 2C 49
Ma07_g04810	ITC1587_Bchr7_P18970	67.65	1.85	0.55	3.38	7.28E-04	0.09	transcription factor MYC4-like
Ma11_g24720	ITC1587_Bchr11_P34360	72.56	1.82	0.44	4.1	4.15E-05	0.025	probable receptor-like protein kinase At1g11050
Ma08_g04840	ITC1587_Bchr8_P21878	143.85	1.81	0.51	3.53	4.19E-04	0.07	probable zinc metallopeptidase EGY3
Ma02_g01590	ITC1587_Bchr2_P03138	79.15	1.77	0.45	3.94	7.99E-05	0.03	receptor-like serine/threonine-protein kinase

								At2g45590
Ma05_g05440	ITC1587_Bchr5_P12164	158.32	1.69	0.43	3.97	7.28E-05	0.029	putative abscisic acid-insensitive 5-like protein 5
Ma06_g13850	ITC1587_Bchr6_P15877	340.76	1.69	0.49	3.46	5.32E-04	0.076	Stromal 70 kDa heat shock-related protein
Ma01_g10350	ITC1587_Bchr1_P01490	50.13	1.69	0.49	3.44	5.73E-04	0.079	putative small heat shock protein
Ma02_g24350	ITC1587_Bchr2_P05093	139.8	1.68	0.45	3.73	1.91E-04	0.048	DELLA protein SLR1-like
Ma02_g22370	ITC1587_Bchr2_P04925	82.72	1.68	0.36	4.63	3.59E-06	0.005	uncharacterized LOC103976537
Ma08_g33170	ITC1587_Bchr8_P24900	594.21	1.65	0.43	3.8	1.46E-04	0.041	SKP1-interacting partner 15-like
Ma04_g15450	ITC1587_Bchr4_P09803	135.17	1.65	0.38	4.3	1.69E-05	0.015	bifunctional pinoresinol-lariciresinol reductase 2-like
Ma04_g14350	ITC1587_Bchr4_P09695	196.48	1.65	0.49	3.39	6.99E-04	0.089	ninja-family protein 6-like
Ma03_g11520	ITC1587_Bchr3_P06219	3070.78	1.64	0.46	3.57	3.53E-04	0.064	linoleate 13S-lipoxygenase 2-1
Ma04_g22560	ITC1587_BchrUn_random_P38009*	53.07	1.64	0.4	4.06	4.86E-05	0.028	serine/threonine-protein kinase PBS1-like
Ma11_g01700	ITC1587_Bchr11_P31841	70.45	1.62	0.45	3.58	3.43E-04	0.064	transcription factor TCP21-like
Ma04_g01770	ITC1587_Bchr4_P08549	129.59	1.59	0.41	3.86	1.14E-04	0.035	heavy metal-associated isoprenylated plant protein 26-like
Ma04_g28700	ITC1587_Bchr4_P10691	117.96	1.58	0.33	4.72	2.38E-06	0.004	uncharacterized LOC103983020
Ma07_g04700	ITC1587_Bchr7_P18961	373.86	1.53	0.36	4.23	2.32E-05	0.017	trihelix transcription factor GTL1-like
Ma02_g14650	ITC1587_Bchr2_P04229	191.59	1.52	0.44	3.5	4.73E-04	0.073	3-oxo-5-alpha-steroid 4-dehydrogenase 2-like
Ma03_g01580	ITC1587_Bchr3_P05308	53.29	1.49	0.43	3.47	5.12E-04	0.076	ras-related protein Rab11D-like
Ma07_g19450	ITC1587_Bchr7_P20601	334.8	1.46	0.4	3.69	2.23E-04	0.052	E3 ubiquitin-protein ligase XB3-like
Ma09_g07630	ITC1587_Bchr9_P25718	1041.77	1.39	0.41	3.38	7.29E-04	0.09	ribonuclease 3-like
Ma11_g07930	ITC1587_Bchr11_P32457	144.78	1.38	0.36	3.87	1.09E-04	0.035	saposin-like type B
Ma02_g08090	ITC1587_Bchr2_P03653	142.42	1.38	0.41	3.35	8.14E-04	0.094	squamosa promoter-binding-like protein 16
Ma02_g24140	ITC1587_Bchr2_P05074	707.64	1.34	0.37	3.63	2.81E-04	0.058	uncharacterized LOC103976698
Ma09_g24360	ITC1587_Bchr9_P27692	96.63	1.3	0.32	4.04	5.33E-05	0.028	glutamate receptor 3.3-like
Ma09_g07620	ITC1587_Bchr9_P25718*	590.13	1.28	0.34	3.8	1.43E-04	0.04	extracellular ribonuclease LE-like
Ma05_g17960	ITC1587_BchrUn_random_P37051	131.06	1.27	0.36	3.54	4.03E-04	0.069	auxin response factor 24-like
Ma10_g11950	ITC1587_Bchr10_P29975	131.81	1.25	0.32	3.86	1.11E-04	0.035	non-specific phospholipase C6-like
Ma05_g20310	ITC1587_Bchr5_P13639	822.89	1.18	0.33	3.55	3.78E-04	0.066	alpha

Ma11_g02180	ITC1587_Bchr11_P31893	234.68	1.18	0.33	3.55	3.82E-04	0.066	uncharacterized LOC103970212
Ma09_g00910	ITC1587_Bchr9_P25142	122.53	1.15	0.29	3.96	7.48E-05	0.029	uncharacterized LOC103996620
Ma11_g00900	ITC1587_Bchr11_P31769	60.21	1.15	0.34	3.34	8.36E-04	0.094	uncharacterized LOC103970102
Ma11_g00480	ITC1587_Bchr11_P31725	107.84	1.14	0.33	3.47	5.22E-04	0.076	uncharacterized LOC103970820
Ma00_g02720	ITC1587_Bchr9_P27274*	1878.38	1.06	0.26	4.03	5.57E-05	0.028	translationally-controlled tumor protein homolog
Ma10_g00340	ITC1587_BchrUn_random_P35065	152.07	1.01	0.27	3.75	1.79E-04	0.046	zinc finger protein NUTCRACKER-like
Ma06_g09630	ITC1587_Bchr6_P15499	209.66	0.91	0.23	3.85	1.16E-04	0.035	transport inhibitor response 1-like protein Os04g0395600
Ma01_g07230	ITC1587_Bchr1_P01211	885.68	0.84	0.25	3.33	8.58E-04	0.095	chaperone protein ClpB1
Ma10_g28350	ITC1587_Bchr10_P31390	382.89	0.8	0.24	3.32	8.96E-04	0.097	ADP
Ma10_g15700	ITC1587_Bchr10_P30299	590.17	-0.62	0.15	-4.16	3.23E-05	0.022	uncharacterized protein At5g03900
Ma09_g09320	ITC1587_Bchr9_P25865	609.55	-0.63	0.19	-3.31	9.29E-04	0.1	thioredoxin-like protein HCF164
Ma05_g00120	ITC1587_Bchr11_P33865*	1803.39	-0.63	0.17	-3.78	1.56E-04	0.042	ethylene receptor-like
Ma09_g02360	ITC1587_Bchr9_P25281	2209.12	-0.64	0.19	-3.37	7.61E-04	0.09	cysteine synthase-like
Ma04_g21440	ITC1587_Bchr3_P08301*	956.05	-0.64	0.19	-3.34	8.34E-04	0.094	NAD-dependent malic enzyme 59 kDa isoform
Ma06_g27370	ITC1587_Bchr6_P17504*	1039.22	-0.65	0.2	-3.32	9.01E-04	0.097	chlorophyllide a oxygenase
Ma08_g03140	ITC1587_Bchr8_P21741	481	-0.66	0.17	-3.97	7.28E-05	0.029	magnesium transporter MRS2-11
Ma03_g14310	ITC1587_BchrUn_random_P35638	2822.32	-0.66	0.17	-3.94	8.04E-05	0.03	malate dehydrogenase [NADP]
Ma08_g09410	ITC1587_Bchr8_P22284	762.06	-0.68	0.21	-3.33	8.75E-04	0.096	uncharacterized LOC103993412
Ma04_g36370	ITC1587_Bchr4_P11363	872.41	-0.73	0.18	-3.98	6.97E-05	0.029	serine carboxypeptidase-like 34
Ma06_g27790	ITC1587_Bchr6_P17541	327.34	-0.75	0.23	-3.33	8.54E-04	0.095	seven transmembrane domain-containing tyrosine-protein kinase 1-like
Ma05_g03790	ITC1587_Bchr5_P12009	373.42	-0.76	0.22	-3.45	5.65E-04	0.079	peptidyl-tRNA hydrolase ICT1
Ma02_g08630	ITC1587_Bchr8_P24756*	347.95	-0.77	0.21	-3.59	3.36E-04	0.063	phosphoglucomutase
Ma09_g20320	ITC1587_BchrUn_random_P36341	358.14	-0.77	0.23	-3.35	8.05E-04	0.093	uncharacterized LOC103973687
Ma09_g20720	ITC1587_Bchr6_P16800	3176.66	-0.79	0.21	-3.84	1.25E-04	0.037	salt tolerance protein-like
Ma04_g02270	ITC1587_Bchr4_P08596	781.29	-0.8	0.23	-3.52	4.33E-04	0.07	apoptosis-inducing factor homolog B-like
Ma05_g05820	ITC1587_Bchr10_P31265*	295.78	-0.84	0.23	-3.61	3.10E-04	0.061	transcription factor HY5-like

Ma05_g27870	ITC1587_Bchr5_P14402	328.78	-0.84	0.19	-4.38	1.17E-05	0.012	4-coumarate--CoA ligase-like 5
Ma08_g33640	ITC1587_Bchr11_P34301*	324.52	-0.86	0.25	-3.47	5.21E-04	0.076	probable UDP-arabinose 4-epimerase 2
Ma07_g23550	ITC1587_Bchr7_P20975	261.66	-0.88	0.23	-3.89	9.86E-05	0.033	probable serine/threonine-protein kinase NAK
Ma10_g11820	ITC1587_Bchr10_P29965	247.81	-0.89	0.25	-3.52	4.33E-04	0.07	probable WRKY transcription factor 4
Ma04_g11640	ITC1587_Bchr4_P09437	151.97	-0.91	0.27	-3.31	9.18E-04	0.099	vacuolar protein-sorting-associated protein 37 homolog 1-like
Ma04_g13320	ITC1587_Bchr4_P09589	615.69	-0.92	0.23	-3.99	6.49E-05	0.029	protein kinase and PP2C-like domain-containing protein
Ma06_g10070	ITC1587_Bchr6_P15537	278.46	-0.94	0.26	-3.6	3.14E-04	0.061	probable peptide/nitrate transporter At3g43790
Ma08_g22120	ITC1587_Bchr8_P23908	877.71	-0.95	0.28	-3.35	8.23E-04	0.094	uncharacterized aarF domain-containing protein kinase At1g79600
Ma07_g26010	ITC1587_Bchr7_P21209	553.58	-0.95	0.28	-3.39	6.90E-04	0.089	RNA polymerase sigma factor sigE
Ma04_g16550	ITC1587_BchrUn_random_ P36402	420.33	-0.98	0.25	-3.96	7.48E-05	0.029	malate dehydrogenase
Ma10_g31030	ITC1587_Bchr10_P31636*	479.88	-0.99	0.29	-3.42	6.33E-04	0.084	stAR-related lipid transfer protein 7
Ma07_g26920	ITC1587_Bchr3_P08271*	765.2	-1.02	0.3	-3.38	7.36E-04	0.09	cytochrome P450 72A15-like
Ma05_g31110	ITC1587_Bchr5_P14697	91.38	-1.03	0.3	-3.37	7.52E-04	0.09	aldo-keto reductase family 4 member C9-like
Ma08_g27550	ITC1587_Bchr8_P24399	391.2	-1.06	0.3	-3.52	4.35E-04	0.07	uncharacterized LOC103996158
Ma06_g19140	ITC1587_Bchr10_P29806*	177.88	-1.07	0.29	-3.69	2.25E-04	0.052	nifU-like protein 2
Ma03_g17790	ITC1587_Bchr3_P07009	488.13	-1.07	0.29	-3.68	2.31E-04	0.052	quinolinate synthase
Ma05_g14290	ITC1587_Bchr5_P12942	418.35	-1.07	0.31	-3.47	5.15E-04	0.076	TOM1-like protein 1
Ma01_g13980	ITC1587_Bchr1_P01811	216.92	-1.12	0.31	-3.61	3.06E-04	0.061	uncharacterized LOC103995168
Ma06_g36860	ITC1587_Bchr6_P18350	367.81	-1.12	0.32	-3.57	3.61E-04	0.064	probable glycerophosphoryl diester phosphodiesterase 3
Ma01_g18190	ITC1587_Bchr1_P02179	313.97	-1.13	0.33	-3.47	5.11E-04	0.076	U-box domain-containing protein 12-like
Ma02_g06570	ITC1587_Bchr2_P03523	351.94	-1.15	0.29	-3.98	6.86E-05	0.029	uncharacterized LOC103972089
Ma09_g27120	ITC1587_Bchr9_P27954*	272.96	-1.15	0.25	-4.68	2.80E-06	0.004	thylakoidal processing peptidase 1
Ma08_g21800	ITC1587_Bchr10_P29069*	1286.49	-1.16	0.34	-3.44	5.92E-04	0.08	protein IN2-1 homolog B-like
Ma10_g27080	ITC1587_Bchr10_P31270	183.77	-1.16	0.35	-3.37	7.39E-04	0.09	uncharacterized LOC103969357
Ma06_g04950	ITC1587_Bchr6_P15079	597.7	-1.17	0.34	-3.4	6.66E-04	0.087	sulfate transporter 1.3-like
Ma11_g22350	ITC1587_Bchr5_P14718*	226.79	-1.18	0.35	-3.34	8.35E-04	0.094	ultraviolet-B receptor UVR8-like
Ma03_g26080	ITC1587_Bchr2_P03269*	282.74	-1.19	0.33	-3.57	3.51E-04	0.064	pyruvate

Ma10_g13670	ITC1587_Bchr6_P16151*	294.46	-1.22	0.34	-3.63	2.79E-04	0.058	soluble inorganic pyrophosphatase-like
Ma07_g08190	ITC1587_Bchr7_P19297	848.13	-1.22	0.35	-3.47	5.19E-04	0.076	thylakoidal processing peptidase 1
Ma10_g11130	ITC1587_Bchr10_P29903	4164.01	-1.24	0.29	-4.26	2.04E-05	0.016	GDP-L-galactose phosphorylase 1-like
Ma04_g09390	ITC1587_Bchr1_P00892*	222.86	-1.25	0.35	-3.52	4.39E-04	0.07	protein RER1A-like
Ma03_g32490	ITC1587_Bchr3_P08270*	339.42	-1.25	0.36	-3.5	4.58E-04	0.072	cytochrome P450 CYP72A219-like
Ma03_g26960	ITC1587_Bchr3_P07815	248.35	-1.26	0.31	-4.01	6.00E-05	0.029	serine carboxypeptidase-like 2
Ma09_g16370	ITC1587_Bchr9_P26574	269.22	-1.27	0.36	-3.58	3.49E-04	0.064	probable calcium-transporting ATPase 5
Ma00_g02240	ITC1587_BchrUn_random_ P37437	476.31	-1.27	0.3	-4.26	2.05E-05	0.016	zeaxanthin epoxidase
Ma11_g08170	ITC1587_Bchr8_P22086*	54.97	-1.28	0.37	-3.44	5.72E-04	0.079	G-type lectin S-receptor-like serine/threonine-protein kinase B120
Ma08_g10850	ITC1587_Bchr8_P22421	564.02	-1.34	0.31	-4.38	1.21E-05	0.012	transcription factor UNE10
Ma03_g32450	ITC1587_Bchr3_P08271*	4823.91	-1.34	0.32	-4.18	2.97E-05	0.022	cytochrome P450 CYP72A219-like
Ma03_g09090	ITC1587_Bchr3_P06004	207.34	-1.34	0.35	-3.78	1.57E-04	0.042	probable mediator of RNA polymerase II transcription subunit 26b
Ma02_g14750	ITC1587_Bchr2_P04237	83.51	-1.34	0.37	-3.67	2.40E-04	0.052	nuclear transcription factor Y subunit A-10-like
Ma03_g32460	ITC1587_Bchr3_P08271*	2272.19	-1.38	0.39	-3.53	4.23E-04	0.07	cytochrome P450 CYP72A219-like
Ma08_g15940	ITC1587_Bchr8_P22791	418.48	-1.42	0.36	-3.98	6.75E-05	0.029	DNA-binding protein SMUBP-2
Ma03_g32500	ITC1587_Bchr3_P08271	2897.68	-1.45	0.31	-4.64	3.44E-06	0.005	cytochrome P450 CYP72A219-like
Ma09_g11540	ITC1587_Bchr9_P26059	383.07	-1.46	0.43	-3.37	7.45E-04	0.09	uncharacterized LOC103997793
Ma09_g07230	ITC1587_Bchr9_P25690	228.48	-1.46	0.34	-4.34	1.40E-05	0.013	uncharacterized LOC103997302
Ma07_g25830	ITC1587_Bchr7_P21189	158.78	-1.47	0.38	-3.85	1.20E-04	0.036	uncharacterized LOC103992727
Ma07_g24330	ITC1587_Bchr7_P21046	88.87	-1.47	0.44	-3.33	8.62E-04	0.095	uncharacterized LOC103992585
Ma05_g07350	ITC1587_Bchr5_P12340	187.04	-1.48	0.4	-3.69	2.28E-04	0.052	NAC domain-containing protein 68
Ma04_g03710	ITC1587_Bchr4_P08718	1173.15	-1.49	0.43	-3.5	4.59E-04	0.072	sugar transport protein 13-like
Ma06_g07010	ITC1587_Bchr6_P15256	415.96	-1.53	0.39	-3.95	7.72E-05	0.03	cyanidin 3-O-rutinoside 5-O-glucosyltransferase-like
Ma04_g36790	ITC1587_Bchr4_P11398	413.69	-1.54	0.33	-4.72	2.40E-06	0.004	probable inorganic phosphate transporter 1-8
Ma06_g07000	ITC1587_Bchr6_P15254*	221.69	-1.55	0.39	-3.97	7.16E-05	0.029	crocetin glucosyltransferase
Ma07_g25310	ITC1587_Bchr7_P21138	1600.58	-1.56	0.42	-3.75	1.78E-04	0.046	ABC transporter A family member 7-like
Ma03_g14360	ITC1587_BchrUn_random_ P35631	261.31	-1.59	0.47	-3.37	7.60E-04	0.09	thebaine 6-O-demethylase-like

Ma10_g28480	ITC1587_Bchr10_P31403	657.96	-1.6	0.46	-3.46	5.36E-04	0.076	ABC transporter C family member 3-like
Ma08_g11100	ITC1587_Bchr8_P22450	118.47	-1.65	0.48	-3.46	5.37E-04	0.076	MLO-like protein 6
Ma06_g14710	ITC1587_Bchr6_P15949	94.7	-1.66	0.44	-3.75	1.80E-04	0.046	Beta-fructofuranosidase
Ma07_g01650	ITC1587_Bchr7_P18698	93.06	-1.66	0.45	-3.68	2.33E-04	0.052	purple acid phosphatase 2-like
Ma01_g23640	ITC1587_BchrUn_random_ P34919	350.45	-1.72	0.47	-3.62	2.93E-04	0.059	probable serine acetyltransferase 4
Ma06_g02210	ITC1587_Bchr6_P14826	215.11	-1.82	0.48	-3.83	1.30E-04	0.038	uncharacterized LOC103986574
Ma09_g10270	ITC1587_Bchr9_P25948	1160.42	-1.83	0.53	-3.44	5.78E-04	0.079	perakine reductase-like
Ma09_g11880	ITC1587_Bchr9_P26091	177.48	-1.85	0.55	-3.39	7.02E-04	0.089	uncharacterized LOC103997701
Ma02_g23000	ITC1587_Bchr2_P04978	431.42	-1.87	0.48	-3.91	9.09E-05	0.031	NAC transcription factor NAM-B1-like
Ma04_g06400	ITC1587_Bchr4_P08952	95.67	-1.92	0.55	-3.52	4.29E-04	0.07	high-affinity nitrate transporter 2.3-like
Ma06_g37470	ITC1587_Bchr9_P25803*	105.99	-1.93	0.47	-4.09	4.25E-05	0.025	cationic amino acid transporter 7
Ma06_g24400	ITC1587_Bchr6_P17231	69.13	-1.94	0.57	-3.39	6.87E-04	0.089	phosphatidylinositol transfer protein 1-like
Ma09_g30300	ITC1587_Bchr9_P28242	727.44	-1.99	0.59	-3.36	7.79E-04	0.091	probable CCR4-associated factor 1 homolog 11
Ma08_g26460	ITC1587_Bchr8_P24305	52.28	-1.99	0.58	-3.45	5.58E-04	0.078	uncharacterized LOC103996250
Ma05_g30790	ITC1587_Bchr5_P14670	213.02	-2.02	0.44	-4.65	3.38E-06	0.005	chr5
Ma08_g31320	ITC1587_Bchr8_P24755	404.2	-2.05	0.56	-3.68	2.30E-04	0.052	glutamine synthetase cytosolic isozyme 2-like
Ma03_g25040	ITC1587_Bchr3_P07650	98.74	-2.06	0.57	-3.58	3.39E-04	0.063	squamosa promoter-binding-like protein 9
Ma10_g24780	ITC1587_Bchr10_P31078	116.65	-2.08	0.53	-3.92	8.85E-05	0.031	E3 ubiquitin-protein ligase ATL6-like
Ma05_g17620	ITC1587_BchrUn_random_ P36044	53.39	-2.08	0.62	-3.35	8.21E-04	0.094	expressed protein
Ma06_g01150	ITC1587_Bchr11_P32690	448.45	-2.08	0.49	-4.28	1.88E-05	0.016	probable WRKY transcription factor 26
Ma05_g08890	ITC1587_Bchr5_P12481	101.3	-2.14	0.63	-3.38	7.14E-04	0.09	uncharacterized LOC103984321
Ma11_g03400	ITC1587_Bchr11_P32010	312.71	-2.14	0.52	-4.16	3.22E-05	0.022	cytochrome P450 89A2-like
Ma02_g12920	ITC1587_Bchr2_P04083	21.27	-2.19	0.6	-3.62	2.90E-04	0.059	probable L-type lectin-domain containing receptor kinase S.5
Ma09_g30270	ITC1587_Bchr9_P28239	267.46	-2.23	0.56	-3.95	7.78E-05	0.03	protein YLS9
Ma03_g16390	ITC1587_BchrUn_random_ P37550	22.51	-2.27	0.59	-3.86	1.12E-04	0.035	probable strigolactone esterase D14
Ma04_g02820	ITC1587_Bchr4_P08649	437.77	-2.37	0.42	-5.7	1.23E-08	0	cytochrome P450 CYP72A219-like
Ma04_g03780	ITC1587_Bchr4_P08726	115.75	-2.4	0.6	-3.99	6.63E-05	0.029	AP2/ERF and B3 domain-containing transcription factor RAV1-like

Ma01_g21550	ITC1587_BchrUn_random_ P37779	627.99	-2.4	0.69	-3.46	5.39E-04	0.076	3-hydroxy-3-methylglutaryl-coenzyme A reductase 3-like
Ma03_g32480	ITC1587_Bchr3_P08271*	32.5	-2.4	0.6	-4.03	5.59E-05	0.028	Secologanin synthase
Ma05_g08420	ITC1587_Bchr5_P12435	196.71	-2.43	0.67	-3.6	3.21E-04	0.061	zinc finger protein ZAT6-like
Ma05_g09420	ITC1587_Bchr5_P12523	103.85	-2.46	0.6	-4.1	4.08E-05	0.025	probable WRKY transcription factor 61
Ma07_g18480	ITC1587_BchrUn_random_ P37067	76.96	-2.52	0.64	-3.92	8.70E-05	0.031	uncharacterized LOC103974030
Ma10_g08130	ITC1587_Bchr10_P29571	315.75	-2.59	0.69	-3.74	1.84E-04	0.047	NBS-LRR disease resistance protein
Ma06_g02890	ITC1587_Bchr6_P14885	464.12	-2.65	0.53	-4.99	6.18E-07	0.003	ubiquinol oxidase 2
Ma04_g12590	ITC1587_Bchr4_P09526	86.38	-2.66	0.7	-3.79	1.49E-04	0.041	CASP-like protein 2C1
Ma10_g08180	ITC1587_Bchr10_P29571*	78.06	-2.76	0.78	-3.55	3.79E-04	0.066	NBS-LRR disease resistance protein
Ma01_g17660	ITC1587_Bchr1_P02228	67.65	-2.87	0.8	-3.6	3.15E-04	0.061	60S ribosomal protein L19-2-like
Ma01_g03350	ITC1587_Bchr1_P00730*	1004.27	-2.94	0.61	-4.79	1.69E-06	0.004	ABC transporter G family member 39-like
Ma01_g21430	ITC1587_Bchr7_P21273*	61.16	-3	0.76	-3.92	8.77E-05	0.031	Hydroxyacylglutathione hydrolase 3
Ma03_g07490	ITC1587_Bchr3_P05835*	11.92	-3.05	0.83	-3.66	2.50E-04	0.053	MACPF domain-containing protein At1g14780-like
Ma04_g32760	ITC1587_Bchr4_P11054	17.25	-3.19	0.89	-3.57	3.60E-04	0.064	receptor-like protein 12
Ma10_g12750	ITC1587_Bchr5_P14061*	110.8	-3.22	0.83	-3.89	9.96E-05	0.033	uncharacterized LOC104000771
Ma09_g17280	ITC1587_BchrUn_random_ P36874	14.81	-3.26	0.98	-3.33	8.78E-04	0.096	protein NRT1/ PTR FAMILY 5.1-like
Ma04_g31000	ITC1587_Bchr4_P10904	24.72	-3.72	1.06	-3.52	4.35E-04	0.07	caffeic acid 3-O-methyltransferase-like
Ma06_g11140	ITC1587_Bchr6_P15633	22.39	-3.75	0.98	-3.82	1.35E-04	0.039	Myb-related protein 308
Ma06_g32410	ITC1587_Bchr6_P17971	29.12	-3.8	1.09	-3.47	5.13E-04	0.076	premnaspirodiene oxygenase-like
Ma06_g32370	ITC1587_Bchr6_P17968	14.46	-3.81	1.13	-3.39	7.07E-04	0.089	premnaspirodiene oxygenase-like
Ma11_g03310	ITC1587_Bchr11_P32002	23.74	-3.84	1.1	-3.49	4.81E-04	0.074	Cytochrome b5 isoform 1
Ma10_g28190	ITC1587_Bchr10_P31374	23.63	-3.9	0.91	-4.29	1.77E-05	0.015	probable WRKY transcription factor 72
Ma02_g10360	ITC1587_Bchr2_P03864	31.91	-3.93	0.97	-4.03	5.53E-05	0.028	uncharacterized LOC103976380
Ma05_g26410	ITC1587_Bchr5_P14270	17.1	-4.12	1.14	-3.61	3.08E-04	0.061	pectinesterase/pectinesterase inhibitor PPE8B-like
Ma01_g21700	ITC1587_Bchr1_P02746	15.32	-4.24	1.15	-3.68	2.36E-04	0.052	uncharacterized LOC104000306
Ma05_g04060	ITC1587_Bchr5_P12038	29.77	-4.59	0.94	-4.87	1.13E-06	0.003	U-box domain-containing protein 35
Ma07_g07170	ITC1587_Bchr7_P19196	179.94	-4.65	0.97	-4.78	1.71E-06	0.004	8-hydroxygeraniol dehydrogenase-like

Ma10_g27290	ITC1587_Bchr10_P31295	1471.62	-4.83	1.07	-4.52	6.18E-06	0.007	cytochrome P450 86B1-like
Ma11_g09840	ITC1587_Bchr11_P32947	9.02	-5.62	1.62	-3.48	5.11E-04	0.076	E3 ubiquitin-protein ligase PUB23-like

Note: Gene IDs from *Musa balbisiana* are based on the results of the reciprocal best BLAST search. The best BLAST hit is reported for the genes where the reciprocal best BLAST search hit was not available and it is denoted with *.

Table S7: GO-terms for the BXW-resistant genotype *Musa balbisiana* and the BXW-susceptible genotype Pisang Awak in response to *Xanthomonas campestris* pv. *musacearum* at 12 hpi and 48 hpi.

GOBPID	Pvalue	OddsRatio	ExpCount	Count	Size	Term
***Musa balbisiana* at 12 hpi**						
GO:0002684	0.00023	2.43609	10.9922	24	179	positive regulation of immune system process
GO:0006952	0.0007	1.71848	31.59509	50	515	defense response
GO:0048524	0.00223	5.39993	1.4238	6	23	positive regulation of viral process
GO:0023056	0.00338	3.32693	3.11809	9	52	positive regulation of signaling
GO:0051447	0.0038	Inf	0.12337	2	2	negative regulation of meiotic cell cycle
GO:0010647	0.00387	3.25086	3.17806	9	53	positive regulation of cell communication
GO:0050793	0.00426	5.68707	1.13271	5	19	regulation of developmental process
GO:0050867	0.0067	1.99855	10.26226	19	166	positive regulation of cell activation
GO:0048146	0.00694	2.73999	4.08568	10	66	positive regulation of fibroblast proliferation
GO:0051173	0.00978	1.36117	64.11234	82	1036	positive regulation of nitrogen compound metabolic process
GO:0060252	0.011	30.4227	0.18571	2	3	positive regulation of glial cell proliferation
GO:0060501	0.011	30.4227	0.18571	2	3	positive regulation of epithelial cell proliferation involved in lung morphogenesis
GO:0002694	0.01212	1.73385	14.7332	24	238	regulation of leukocyte activation
GO:0043207	0.01282	1.40113	46.05672	61	744	response to external biotic stimulus
GO:0033555	0.01339	2.60142	3.83806	9	62	multicellular organismal response to stress
GO:1905954	0.01454	5.08317	0.99047	4	16	positive regulation of lipid localization
GO:0001974	0.01496	7.61569	0.55714	3	9	blood vessel remodeling
GO:0031069	0.01496	7.61569	0.55714	3	9	hair follicle morphogenesis
GO:0048583	0.02009	2.02337	6.91091	13	116	regulation of response to stimulus
GO:0048534	0.02027	1.58125	18.68445	28	304	hematopoietic or lymphoid organ development

GO:0060463	0.02111	15.20939	0.24762	2	4	lung lobe morphogenesis
GO:0010883	0.02217	4.35588	1.11428	4	18	regulation of lipid storage
GO:0008218	0.02436	2.6748	2.9095	7	47	bioluminescence
GO:0023014	0.02449	1.4109	33.49017	45	541	signal transduction by protein phosphorylation
GO:0045785	0.02617	1.65894	13.3713	21	216	positive regulation of cell adhesion
GO:0001662	0.02679	5.71029	0.68095	3	11	behavioral fear response
GO:0008356	0.02679	5.71029	0.68095	3	11	asymmetric cell division
GO:1903706	0.02703	1.79459	9.47134	16	153	regulation of hemopoiesis
GO:0048661	0.02782	2.86317	2.35236	6	38	positive regulation of smooth muscle cell proliferation
GO:0043901	0.03065	3.18287	1.79291	5	29	negative regulation of multi-organism process
GO:1903046	0.03184	3.81041	1.23808	4	20	meiotic cell cycle process
GO:0001892	0.03399	5.08251	0.74185	3	12	embryonic placenta development
GO:0017145	0.03411	5.07516	0.74285	3	12	stem cell division
GO:0002118	0.03411	5.07516	0.74285	3	12	aggressive behavior
GO:0031295	0.03881	2.61674	2.53807	6	41	T cell costimulation
GO:0048870	0.04012	4.67355	0.78788	3	13	cell motility
GO:0045596	0.04284	1.65176	10.83506	17	176	negative regulation of cell differentiation
GO:0007275	0.04579	1.49269	17.56727	25	295	multicellular organism development
GO:0051461	0.04857	7.60274	0.37143	2	6	positive regulation of corticotropin secretion
***Musa balbisiana* at 48 hpi**						
GO:0006450	0.03988	29.91273	0.04055	1	6	regulation of translational fidelity
**Pisang Awak at 48 hpi**						
GO:0010942	0.00362	7.52415	0.65802	4	287	positive regulation of cell death
GO:0031323	0.009	8.37433	0.44708	3	207	regulation of cellular metabolic process
GO:0006979	0.0129	6.98958	0.50211	3	219	response to oxidative stress
GO:1901723	0.01368	91.81111	0.01376	1	6	negative regulation of cell proliferation involved in kidney development
GO:0035802	0.01368	91.81111	0.01376	1	6	adrenal cortex formation
GO:0061032	0.01594	76.5	0.01605	1	7	visceral serous pericardium development
GO:0032836	0.01594	76.5	0.01605	1	7	glomerular basement membrane development

GO:0033327	0.01594	76.5	0.01605	1	7	Leydig cell differentiation
GO:0006910	0.0182	65.56349	0.01834	1	8	phagocytosis, recognition
GO:0046620	0.0182	65.56349	0.01834	1	8	regulation of organ growth
GO:0071774	0.02321	9.41869	0.23845	2	104	response to fibroblast growth factor
GO:0060009	0.02495	45.87778	0.02522	1	11	Sertoli cell development
GO:0060430	0.02719	41.70202	0.02751	1	12	lung saccule development
GO:0061138	0.02844	8.41486	0.26596	2	116	morphogenesis of a branching epithelium
GO:0010719	0.02942	38.22222	0.02981	1	13	negative regulation of epithelial to mesenchymal transition
GO:2000242	0.02942	38.22222	0.02981	1	13	negative regulation of reproductive process
GO:0051173	0.03136	3.03297	2.51744	6	1098	positive regulation of nitrogen compound metabolic process
GO:1905939	0.03165	35.27778	0.0321	1	14	regulation of gonad development
GO:0072203	0.03165	35.27778	0.0321	1	14	cell proliferation involved in metanephros development
GO:0007530	0.03831	28.65278	0.03898	1	17	sex determination
GO:0042753	0.04052	26.96405	0.04127	1	18	positive regulation of circadian rhythm
GO:2000243	0.04052	26.96405	0.04127	1	18	positive regulation of reproductive process
GO:0003002	0.04069	6.88024	0.32328	2	141	regionalization
GO:0001505	0.04496	6.4994	0.34162	2	149	regulation of neurotransmitter levels
GO:0009798	0.04931	21.81746	0.05044	1	22	axis specification
GO:0043446	0.04931	21.81746	0.05044	1	22	cellular alkane metabolic process

Table S8: Details of primers used for qRT-PCR analysis for validation of RNA-Seq fold changes in Pisang Awak and *Musa balbisiana* in response to artificial inoculation with *Xanthomonas campestris* pv. *musacearum.*

**Gene Target**	**Forward**	**Reverse**
Ma02_g20530	GCTTCAGATCACCGCATTT	CGAGAACTTTCTTGAGCTTCTT
Ma05_g25630	GGCAGAGGAAGAAGAAGAAG	GTTGACCGAGTTGAGGATG
Ma02_g12480	GAGCAGACCATCAGAGAAAC	TGCCCAGCAGTCATCTAT
Ma06_g26670	GAAGTGGTGGCGTGTTAT	GTGAACTGGAAGGTGAAGAG
Ma03_g31180	TCAGGGCGAGTCGATAATA	CTCTTCTTGGCTTCCTCTTC

Ma09_g22650	ACTTGGATCGGTGAAGATTG	TCGTCTGTGTAGGAGTAAGG
Ma09_g28690	AGCTTCTCTCCCTCACTATC	GCTGCCCATCCAATACAA
Ma04_g38470	TCGAGTGCCAAGCCTAC	CGAGGGAGATGTCGATGAA
Ma09_g05410	ATCTCGTCAACAAGCTCTTC	CTCCATCTCCCATGTCTTTATG
Ma09_g08260	CGACCTCGATCTCTCCATAA	TACCGGTGGATGGTGTT
ITC1587_Bchr4_T08866	CAAGAGCCCACGAGTTG	TCTCTGGCAGGGATGTC
Ma02_g12690	CCTACTCGTAGCCTCTTCTT	GTGGCTGAAATAGTCCTCATC
Ma02_g12710	GGTTCAACAGGAGCAGAAA	GAAAGCATCCGCTACCTATAC
Ma04_g18790	GATGAAGGTAACGGAGAAGTG	CGAAGACAGCGATAGAATGAG
Ma04_g19490	CTCCCTTCCTCACTTATTTCTG	TAGCGTATCGTTCTGTGATTG
Ma06_g14430	GTTCGTCCTCCAAGATGATAAG	TACTCTCTCGGCTAACTTCC
Ma02_g08740	GGGACTTCTATGATGTTGTCTT	CGGATCTACCGCTGATATTG
ITC1587_Bchr1_T01647	AGTACGGCTTCGACCAC	GGTCGATGATGCTCTGGA
Ma04_g33020	CCAACCTGGGAAATCTCTTG	CTGGTGCTGAAGACTGTAAC
Ma07_g03320	ATTGCTGCAGGAGATGATG	TCTCCCTGGTCTTAGCTTT
Ma07_g08690	GGCAATCTCAAGACCATCTC	TTCTCTCACTTCACCCATTTC
Ma08_g00630	GTCTCTTCACCAAGGACATATAC	CCACGACGTTCTTCTTCTTC
Ma11_g13300	CGTCTACCCACCCGATAA	AAAGTGACCACGGTGTTG
Ma04_g02930	GCATTAACAGCGGCATAAAC	CGCTAGAACCTCGACAAATC
Ma08_g16700	CCCACCGTCTGTCTATATCT	CCTCATCGTGGGATCACTA
Ma08_g29360	TGCGGTAAAGGAGGATGA	GCAGGAGTAACAGGACTAGA
Ma10_g12750	GTAGGTGGTCATGGAAGATTAG	GGTTGCTGAAGAGGAATGT
Ma01_g17260	AGTACAGTGATGCTGTTTACTC	CTGCTGTTCTGCATCTCTAC
Ma03_g11520	CTGATCACCGAGGAACTTATC	GTGTCTTCCAGCTCTCTAATC
Ma02_g09860	GTGAGTGAAGCCAAGTATGAG	AGATGGTGCAGCATGTAATC
